# Umpolung Activation of Bicyclobutanes via N‐Heterocyclic Carbene Catalysis

**DOI:** 10.1002/anie.202513913

**Published:** 2025-08-27

**Authors:** Yu‐Che Chang, Renyu Guo, Thomas Fessard, Quentin Lefebvre, Christophe Salome, M. Kevin Brown

**Affiliations:** ^1^ Department of Chemistry Indiana University 800 E. Kirkwood Ave. Bloomington IN 47401 USA; ^2^ SpiroChem AG Rosental area, WRO‐1047‐3, Mattenstrasse 22 Basel 4058 Switzerland; ^3^ Present address: Bristol Myers Squibb Route 206 & Province Line Road, Princeton New Jersey 08543 USA

**Keywords:** Bicyclobutane, Cycloaddition, N–Heterocyclic carbene, Strained ring, Umpolung

## Abstract

Bicyclo[1.1.0]butane (BCB) has predominantly been explored as an electrophile or radical acceptor owing to its inherent polarity. However, its reactivity as a nucleophile remains largely unexplored. Herein, we report an umpolung strategy that reverses the innate polarity of bicyclobutane aldehydes via N‐heterocyclic carbene (NHC) catalysis. This transformation enables the construction of novel rigidified lactones and lactams via coupling with diverse electrophilic partners, including aldehydes, ketones, and imines. The synthetic versatility of this method highlights its potential for applications in medicinal chemistry by providing new building blocks with defined exit vectors.

## Graphical abstract

NHC‐catalyzed umpolung of bicyclo[1.1.0]butane (BCB) aldehydes enables nucleophilic cycloaddition with carbonyls and imines to form bicyclic lactones and lactams. The resulting oxa‐ and aza‐bicyclo[3.1.1]heptane products can be further transformed into valuable rigid 3D scaffolds, offering a simple and versatile approach to access new chemical space with potential applications in medicinal chemistry.

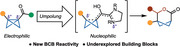

Umpolung is defined as the reversal of the innate polarity of functional groups.^[^ ^1^ ^]^ This reactivity paradigm enables a wide range of valuable chemical transformations to be carried out that would otherwise be inaccessible due to polarity mismatch.^[^ ^2^, ^3^, ^4^, ^5^, ^6^ ^]^ A well‐known example is the polarity inversion of carbonyl compounds, which are traditionally viewed as electrophiles.^[^ ^7^, ^8^, ^9^, ^10^ ^]^ This was first developed with the benzoin condensation^[^ ^11^, ^12^ ^]^ and the Stetter reaction,^[^ ^13^ ^]^ and more recently extended through enal cyclizations reported by Bode,^[^ ^14^, ^15^ ^]^ Glorius,^[^ ^16^ ^]^ Rovis,^[^ ^17^ ^]^ Scheidt,^[^ ^18^ ^]^ and Nair.^[^ ^19^, ^20^ ^]^ These methods have been utilized in a variety of contexts to access a diverse range of complex molecules.^[^ ^21^, ^22^ ^]^

However, one particular class of carbonyl‐containing compounds, bicyclo[1.1.0]butanes (BCBs), has not yet exhibited analogous umpolung‐type reactivity. Since independent studies from our group/SpiroChem,^[^ ^23^ ^]^ the Glorius group,^[^ ^24^ ^]^ and the Procter group^[^ ^25^ ^]^ have demonstrated intermolecular strain‐release cycloadditions of BCBs with alkenes, a wide range of BCB reaction modes have been revealed. For example, Lewis acid catalysis,^[^ ^26^, ^27^, ^28^, ^29^, ^30^, ^31^, ^32^ ^]^ transition metal catalysis,^[^ ^33^, ^34^ ^]^ energy transfer,^[^ ^35^, ^36^, ^37^ ^]^ and single‐electron pathways^[^ ^38^, ^39^, ^40^, ^41^ ^]^ have all been developed in recent years (Scheme 1a). Due to the electrophilic nature of the BCB, capture with nucleophiles or radical acceptors is most common.^[^ ^42^, ^43^ ^]^

**Scheme 1:**
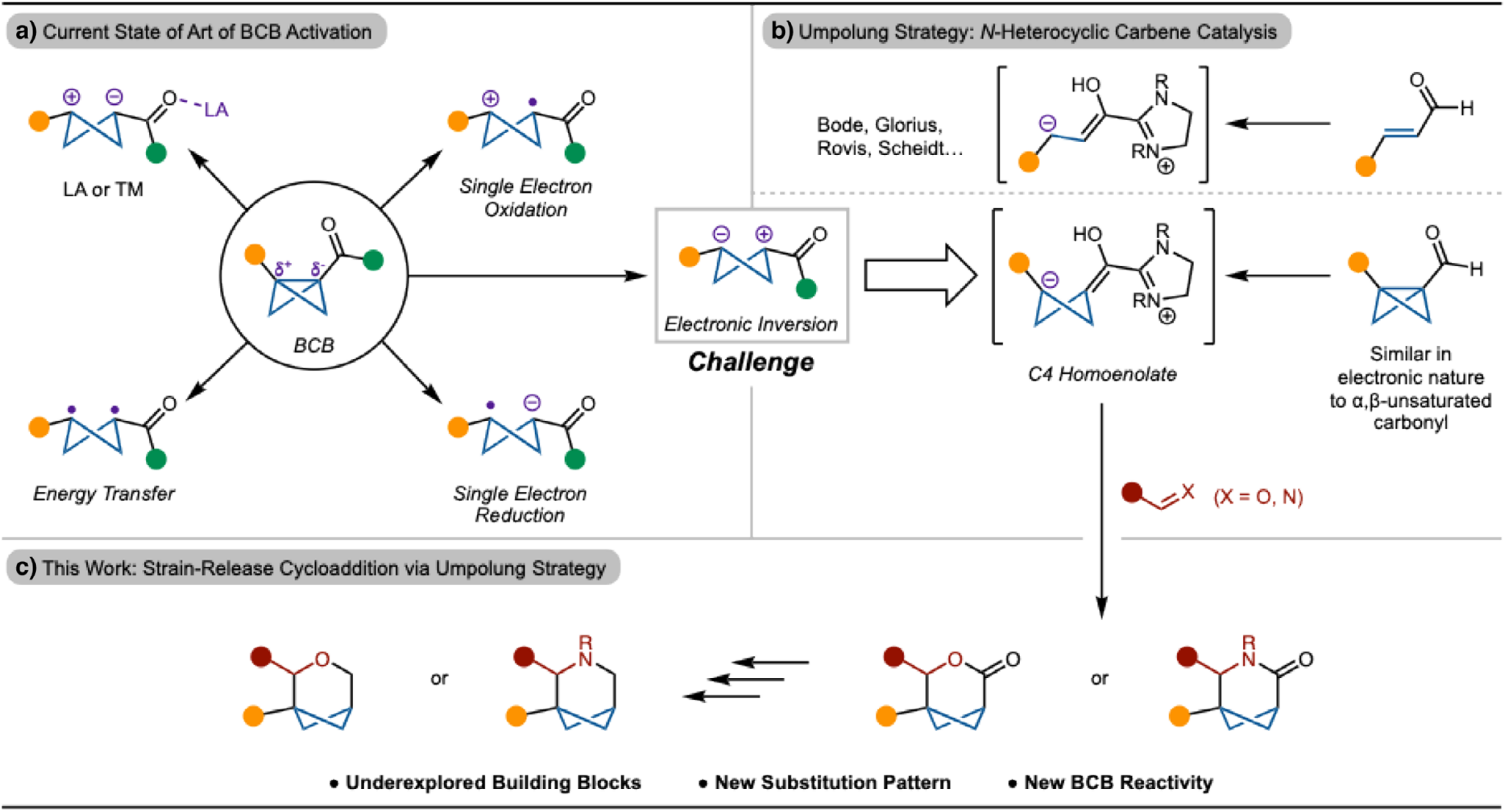
Summary of BCB activation modes

Recognizing this inherent polarity bias, we envisioned that umpolung activation could unlock a complementary reactivity mode. Reversing the innate electrophilicity of the BCB moiety to generate a nucleophilic cycloaddition partner would allow for the synthesis of a variety of new scaffolds. Few reports have disclosed the umpolung activation of BCB through cobalt catalysis and Alder‐ene reaction.^[^ ^44^, ^45^ ^]^ Along these lines, we proposed that the BCB aldehyde can be activated by an N‐heterocyclic carbene (NHC) to generate a Breslow intermediate, which may lead to the formation of a C4‐homoenolate.^[^ ^46^, ^47^, ^48^ ^]^ Either direct reaction of the Breslow intermediate or C4‐homoenolate with different electrophilic partners, such as carbonyls or imines, can lead to the formation of bicyclic lactones or lactams (Scheme 1b). This strategy would allow for the development of a new class of 3D building blocks, for which related structures have attracted attention in medicinal chemistry. Certain rigidified bicyclic scaffolds have been reported to be candidates for benzene isosteres (Scheme 1c).^[^ ^49^, ^50^ ^]^ Considering the novel reactivity proposed and potential synthetic utility, we initiated our investigations on the development of umpolung strain‐release reaction of BCBs. During the final stages of the preparation of this manuscript, Zhou and co‐workers reported a related method on umpolung activation of sulfonylBCBs, which required NHC and Bronsted acid catalysis.^[^ ^51^ ^]^ While this is a significant advance, our studies make complementary products with arylBCBs activation using a single NHC catalyst system. In addition, we demonstrate downstream derivatizations to access complex oxa‐ and aza‐bicyclo[3.1.1]heptanes.

We commenced our studies by treating the bench‐stable BCB aldehyde **1** with NHC catalyst, base, and benzaldehyde (Table 1). An initial hit was observed with the use of NHC precursor IMes•HCl (Table 1, entry 1). Sterically more demanding NHC precursor, IPr•HCl, performed similarly to the smaller catalyst (Table 1, entry 2). A significant increase in yield was observed with NHC precursor SIMes•HCl (Table 1, entry 4). Alkyl NHC precursors were also evaluated but did not demonstrate any reactivity (Table 1, entries 5–7). Several chiral NHC precursors were tested, but only the racemic product was detected (see Supporting Information for details). Evaluation of other bases and solvents did not lead to improved yields (Table 1, entries 8–12).

**Table 1:** Reaction optimization. All reactions are performed on 0.1 mmol scale. Yield was determined by ^1^H NMR analysis of the unpurified reaction mixture using CH_2_Br_2_ as internal standard.

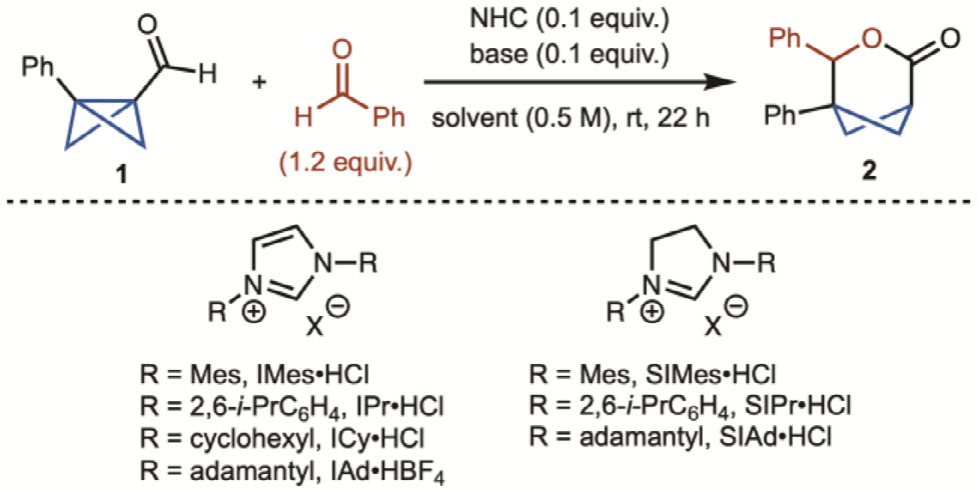				
Entry	NHC	Base	Solvent	Yield
1	IMes•HCl	DBU	THF	30%
2	IPr•HCl	DBU	THF	38%
**3**	**SIMes•HCl**	**DBU**	**THF**	**82%**
4	SIPr•HCl	DBU	THF	62%
5	ICy•HCl	DBU	THF	<5%
6	IAd•HBF_4_	DBU	THF	<5%
7	SIAd•HCl	DBU	THF	<5%
8	SIMes•HCl	Et_3_N	THF	<5%
9	SIMes•HCl	Cs_2_CO_3_	THF	<5%
10	SIMes•HCl	DBU	dioxane	78%
11	SIMes•HCl	DBU	toluene	66%
12	SIMes•HCl	DBU	*t*BuOH	52%

With a set of optimized conditions in hand, various carbonyls were explored (Scheme 2). Electron‐donating groups, such as methoxy and benzodioxole, led to slightly lower yields (products **3** and **7**), perhaps due to the weakened electrophilicity relative to benzaldehyde. On the other hand, electron‐withdrawing groups, including methyl ester and Bpin groups, performed better (products **4** and **6**). Sterically demanding mesityl aldehyde could also be used; however, a lower yield was observed (product **8**). Heteroaryl‐derived aldehydes, including 2‐pyridine, 3‐pyridine, quinoline, thiophene, pyrazole, and pyrrole, all gave rise to product formation (products **9**–**14**). In addition, while acrolein did not lead to product formation (see Supporting Information for details), the gem‐dimethyl‐substituted variant worked well (product **15**). While primary aldehydes gave rise to complex mixtures (see Supporting Information for details), cyclopropyl‐derived aldehyde showed moderate reactivity (product **16**). Ketones such as acetophenone did not lead to product formation. However, activated ketones such as α‐keto, α‐ester, α‐CF_3_, and isatin all generated the desired products **17**–**19** and **21** in moderate to good yields. Chalcone has the potential to engage in Stetter‐type reactivity; however, reaction with the carbonyl was observed to generate product **20**. Finally, various substituted BCB aldehydes were evaluated, and it was found that trifluoromethyl and methoxy substitution was tolerated (products **22** and **23**). An alkyl‐substituted BCB produced desired lactone **24**, albeit in low yield. It was observed that without any external aldehyde partners, the BCB aldehyde undergoes homodimerization to generate **25**. This supported the mechanism for reaction of the NHC with the BCB as opposed to benzaldehyde.

**Scheme 2:**
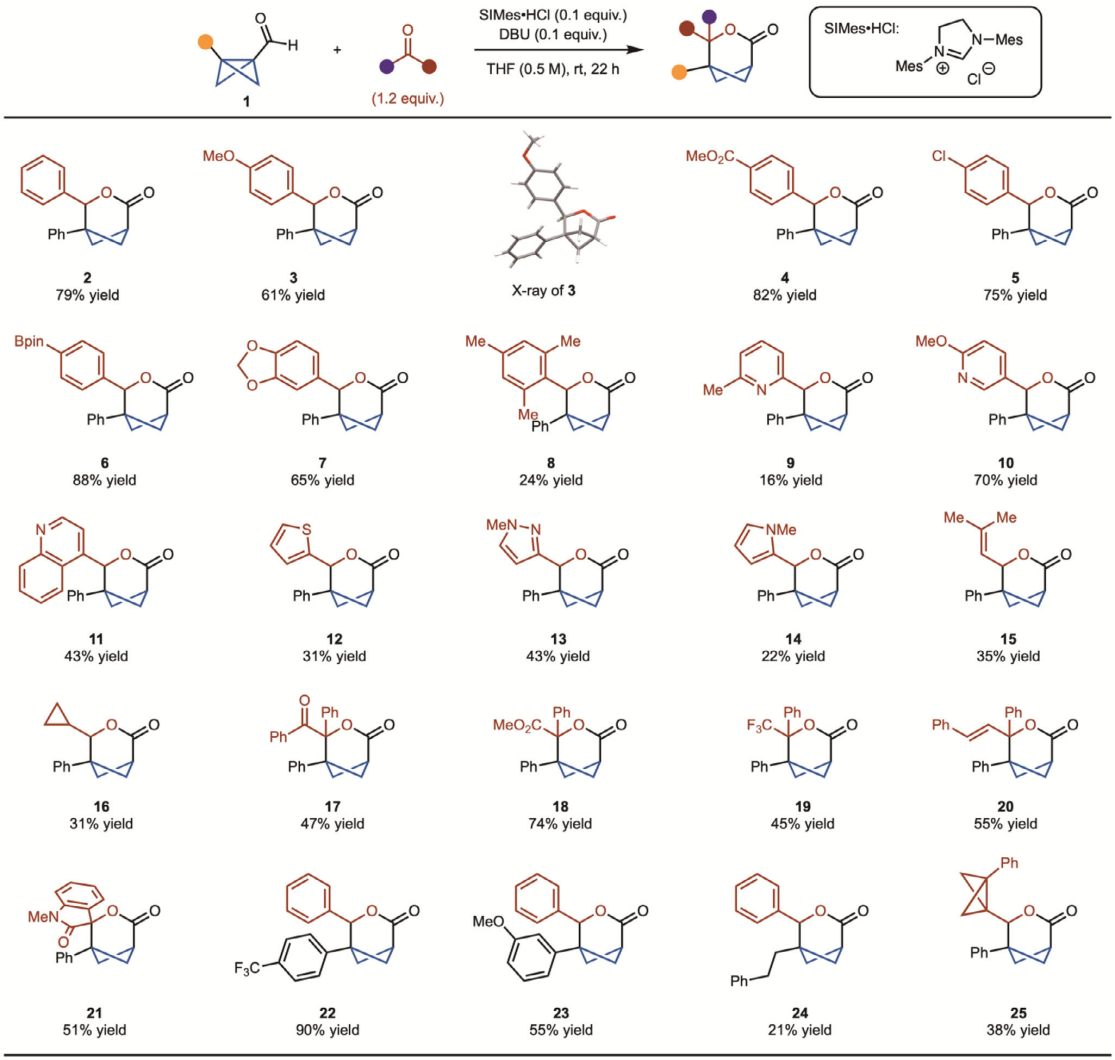
Substrate scope with aldehydes and ketones. Reaction run on 0.2 mmol scale. Yield represents the average of two separate experiments.

After investigating the reactivity with carbonyls, we turned our attention to imines. These are typically more reluctant to engage in Stetter‐ or benzoin‐type reactivity due to reduced electrophilicity relative to aldehydes. To increase reactivity, hydroxy substitution^[^ ^52^ ^]^ or the addition of Lewis acid additives^[^ ^18^, ^53^ ^]^ are often employed. Unfortunately, all imines tested in the presence or absence of various additives did not reveal any promising outcomes (see Supporting Information for details). Ultimately, it was found that isatin‐based *N*‐Boc imine allowed for product formation in 41% yield. Based on this initial hit, reaction optimization revealed that the use of 1.0 equivalents of DBU led to improved yields (68% yield) (see Supporting Information for details). Under the optimized conditions, we examined a variety of imines (Scheme 3). First, the 5‐methoxy group was tolerated in the reaction with a similar yield (product **27**). 4‐Methyl isatin imine led to lower yields, possibly due to the steric hindrance (product **28**). Substituted BCBs also performed well with imines. The trifluoromethylbenzene group exhibited a higher yield (product **29**), while the *p*‐anisole showed a lower yield (product **30**). Finally, the alkyl‐substituted BCB was tolerated with the imine as well (product **31**).

**Scheme 3:**
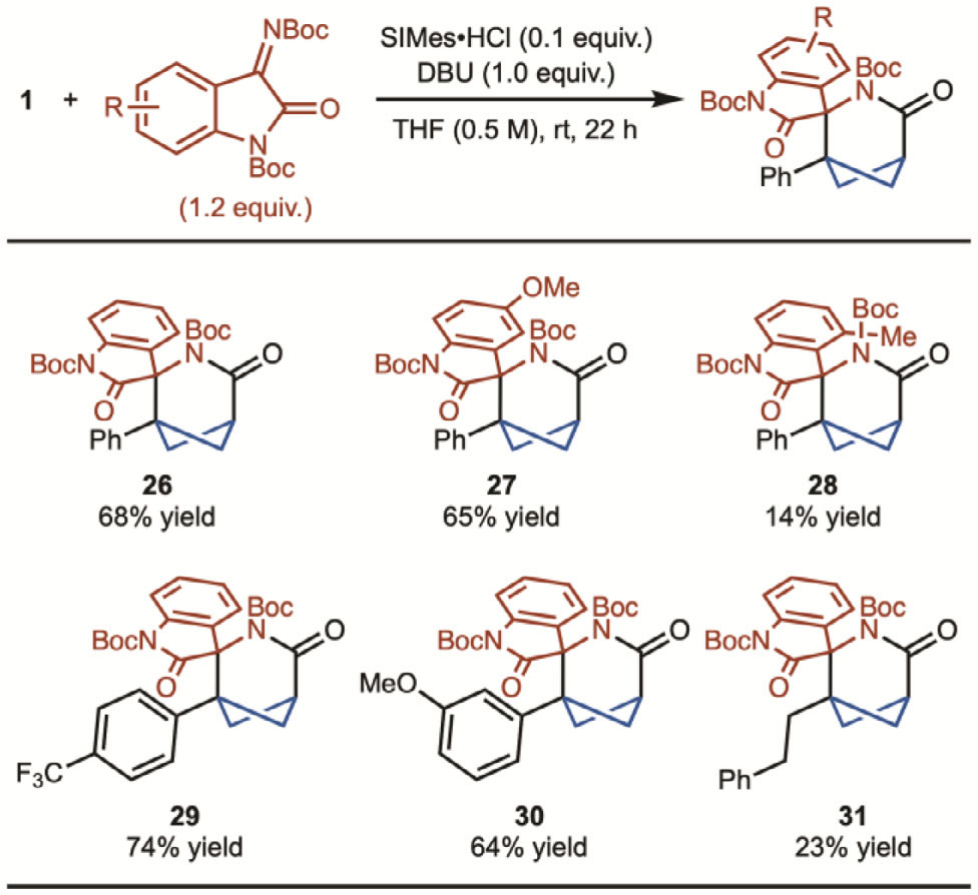
Substrate scope with isatin imine. Reaction run on 0.2 mmol scale. Yield represents the average of two separate experiments.

To demonstrate synthetic utility, a gram‐scale reaction was conducted with little change in yield observed compared to the smaller‐scale reactions (Scheme 4). Lactone **2** could be reduced to pyran **32** via a sequence of DIBAL‐H reduction and silane reduction.^[^ ^54^ ^]^ Polysubstituted pyran **33** was synthesized by a similar route to **32**, but substituting DIBAL‐H with a pre‐generated enolate.^[^ ^55^ ^]^ Finally, lactam **34** was prepared by a three‐step sequence involving lactone ring‐opening with O‐Bn hydroxylamine, mesylation, and intramolecular substitution.^[^ ^56^ ^]^

**Scheme 4:**
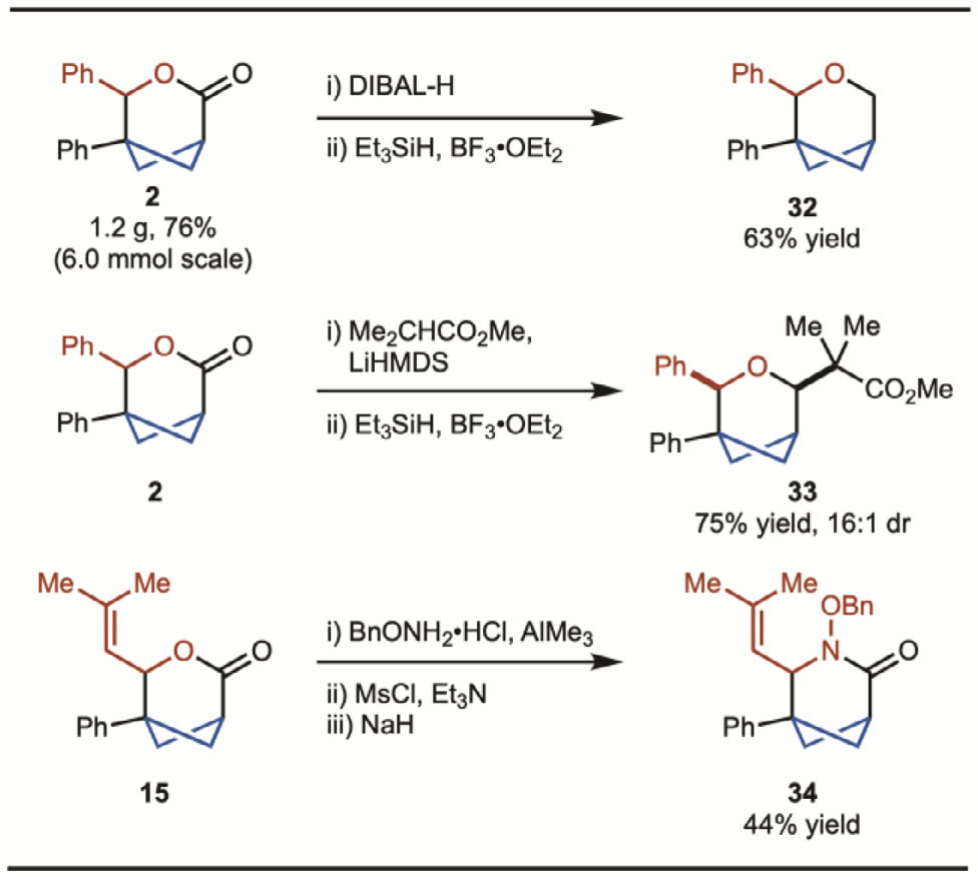
Functional group transformations.

Based on literature reports,^[^ ^44^, ^45^ ^]^ a plausible mechanism is illustrated in Scheme 5. First, SIMes•HCl was converted to the NHC **I** by deprotonation with DBU. Nucleophilic addition of the NHC to BCB aldehyde **1**, followed by a 1,2 proton shift, leads to the formation of Breslow intermediate **II**. Subsequent nucleophilic addition to benzaldehyde generates **III**. Upon tautomerization (**IV**) and lactone formation, the NHC catalyst is regenerated.

**Scheme 5:**
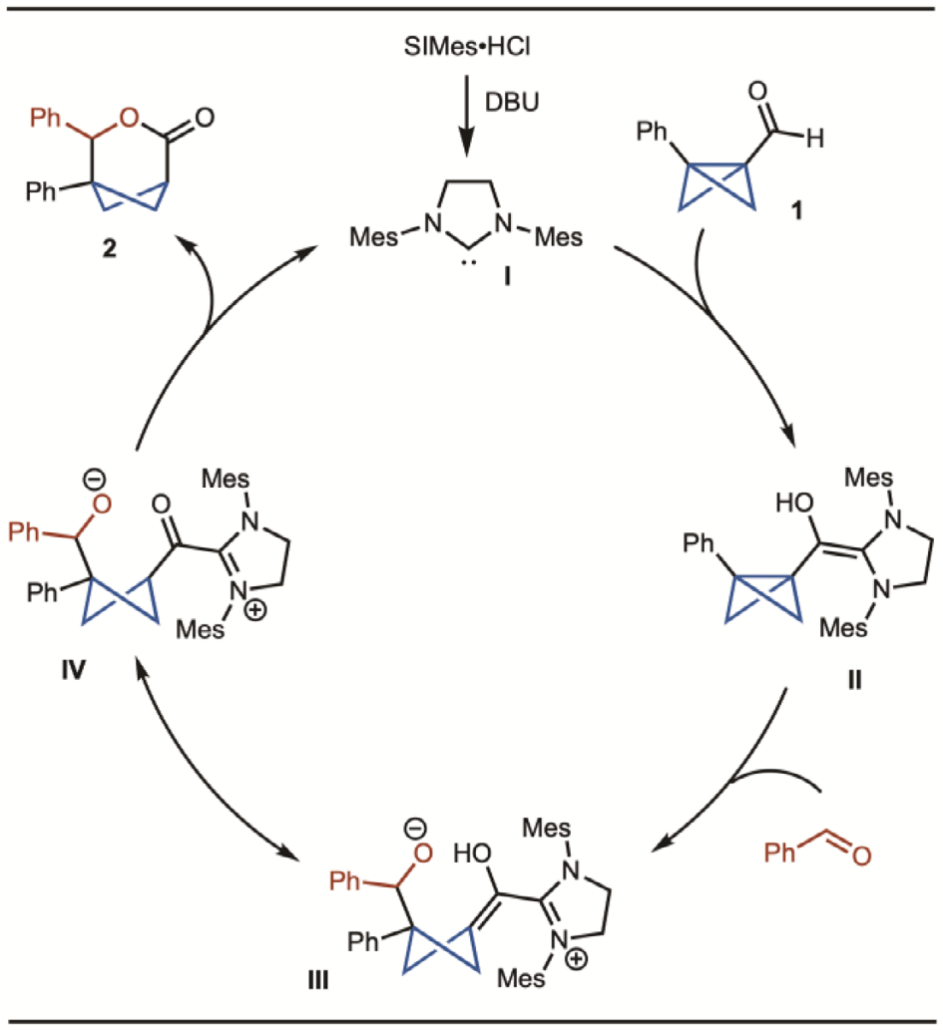
Plausible catalytic cycle.

In conclusion, a new activation mode of BCB is described, which allows for the synthesis of valuable oxa‐ and aza‐bicyclo[3.1.1]heptanes. The reaction conditions are simple and scalable. Moreover, the products are readily amenable to further transformation. This method allows for the synthesis of a new class of 3D building blocks, which will be valuable to access new chemical space.

## Conflict of Interests

The authors declare no conflict of interest.

## Supporting information



Supporting Information

Supporting Information

## Data Availability

The data that support the findings of this study are available in the Supporting Information of this article.
