# Supplementary material for: Umpolung Activation of Bicyclobutanes via N‐Heterocyclic Carbene Catalysis
Source: Angew Chem Int Ed Engl. 2025 Aug 27;64(41):e202513913. doi: 10.1002/anie.202513913 (PMC12501659; doi:10.1002/anie.202513913)
Supplement: Supplementary file 1 — Supporting Information [file ANIE-64-e202513913-s002.pdf]

Umpolung Activation of Bicyclobutanes via N-Heterocyclic Carbene Catalysis

Yu-Che Chang[a], Renyu Guo[a], Thomas Fessard[b], Quentin Lefebvre[b], Christophe Salome[b]*, M. Kevin Brown[a]*

[a]Indiana University, Department of Chemistry, 800 E. Kirkwood Ave., Bloomington, IN 47405
[b]SpiroChem AG, Rosental area, WRO-1047-3, Mattenstrasse 22, 4058 Basel, Switzerland

Supplementary Information – Table of Contents

1. General Information: **2**

2. Reagents and Catalysts: **3**

3. Substrate Synthesis: **5**

4. Optimization Studies: **12**

5. Experimental Procedure for Strain-Release Cycloaddition: **15**

6. Unsuccessful examples: **33**

7. Gram Scale Synthesis and Synthetic Transformations: **34**

8. X-ray Structures: **38**

9. NMR Spectra: **41**

10. References: **84**

1. General Information:

NMR: ^{1}H NMR spectra were recorded at room temperature on a Varian I400 (400 MHz), Varian VXR400 (400 MHz), Varian I500 (500 MHz), Bruker 500 (500 MHz) or a Varian I600 (600 MHz) spectrometer. Chemical shifts are reported in ppm from tetramethylsilane with the residual solvent resonance as the internal standard ($CDCl_3$: 7.26 ppm). Data are reported as follows: chemical shift, multiplicity (s = singlet, d = doublet, t = triplet, q = quartet, br = broad, m = multiplet), coupling constants (Hz), and integration. ^{13}C NMR spectra were recorded on a Varian I400 (101 MHz), Varian I500 (126 MHz) or Bruker 500 (126 MHz) spectrometer with complete proton decoupling. Chemical shifts are reported in ppm from tetramethylsilane with the solvent resonance as the internal standard ($CDCl_3$: 77.16 ppm). ^{19}F NMR spectra were recorded on a Bruker 500 (470 MHz) spectrometer.

HRMS: High-Resolution Mass Spectrometry (HRMS) analysis was obtained using Electrospray Ionization (ESI), Atmospheric Pressure Chemical Ionization (APCI), Electron Ionization (EI) and reported as m/z (relative intensity). ESI and APCI were acquired using a Waters/Micromass LCT Classic (ESI-TOF). EI was acquired using an Agilent 7250 Quadrupole-Time-of-Flight (Q-TOF).

IR: Infrared (IR) spectra were recorded on a Bruker Tensor II FT-IR Spectrometer, ν_{max} in cm^{-1}. Bands are characterized as broad (br), strong (s), medium (m), and weak (w).

Solvents: Dichloromethane (DCM), Tetrahydrofuran (THF), Diethyl ether (Et_2O), dioxane, and Dimethylformamide (DMF) were purified under a positive pressure of dry argon by passage through two columns of activated alumina. Toluene (PhMe) was purified under a positive pressure of dry argon by passage through columns of activated alumina and Q5 (Grubbs apparatus). Methanol (MeOH) was purified over 3Å molecular sieves.

Reactions: Unless otherwise noted, all reactions have been carried out with distilled and degassed solvents under an atmosphere of dry N_2 in an oven (150 °C) and flame-dried glassware with standard vacuum-line techniques. All work-up and purification procedures were carried out with reagent-grade solvents in air.

Purification: Standard flash column chromatography (FCC) techniques using ZEOprep 60/40-63 μm silica gel were used for purification. For difficult separations, medium-pressure liquid chromatography (MPLC) was performed using a Teledyne ISCO CombiFlash Rf 150 instrument.

2. Reagents and Catalysts:

1,8-diazabicyclo(5.4.0)undec-7-ene was purchased from Oakwood and distilled over KOH before use.
1-ethyl-3-(3-dimethylaminopropyl)carbodiimide hydrochloride (EDC) was purchased from Oakwood and used as received.
1-methylindoline-2,3-dione was purchased from Sigma-Aldrich and used as received.
1-methyl-1*H*-pyrazole-3-carbaldehyde was purchased from Combi-Blocks and used as received.
2,2,2-trifluoroacetophenone was purchased from Sigma-Aldrich and used as received.
3-bromoanisole was purchased from Ambeed and used as received.
3-methyl-2-butenal was purchased from Combi-Blocks and used as received.
3-oxocyclobutanecarboxylic acid was purchased from Ambeed and used as received.
4-bromobenzotrifluoride was purchased from Oakwood and used as received.
4-chlorobenzaldehyde was purchased from Ambeed and used as received.
4-dimethylamino pyridine (DMAP) was purchased from Oakwood and used as received.
4-methoxybenzaldehyde was purchased from Chem-Inpex and and distilled before use.
4-methylindoline-2,3-dione was synthesized according to the literature.[1]
4-vinylbenzene boronic acid was purchased from Beantown Chemicals and used as received.
(S)-5-benzyl-2-mesityl-6,6-dimethyl-6,8-dihydro-5H-[1,2,4]triazolo[3,4-c][1,4]oxazin-2-ium tetrafluoroborate was purchased from Sigma-Aldrich and used as received.
5-formyl-2-methoxy-pyridine was purchased from Oakwood and used as received.
5-methoxyindoline-2,3-dione was purchased from Alfa Aesar and used as received.
(5R,6S)-2-mesityl-5,6-diphenyl-6,8-dihydro-5H-[1,2,4]triazolo[3,4-c][1,4]oxazin-2-ium tetrafluoroborate was purchased from Sigma-Aldrich and used as received.
6-methoxynicotinaldehyde was purchased from Oakwood and used as received.
6-methylpicolinaldehyde was purchased from Combi-Blocks and used as received.
benzaldehyde was purchased from Sigma-Aldrich and distilled before use.
benzil was purchased from Oakwood and used as received.
***O*-benzylhydroxylamine hydrochloride** was purchased from Ark Pharm and used as received.
bis(cyclopentadienyl)titanium(IV) dichloride was purchased from Sigma-Aldrich and used as received.
***N*-Boc-imino-(triphenyl)phosphorane** was purchased from Ambeed and used as received.
boron trifluoride diethyl etherate was purchased from Sigma-Aldrich and used as received.
bromobenzene was purchased from Sigma-Aldrich and used as received.
***n*-butyllithium solution (2.5 M in hexanes)** was purchased from Sigma-Aldrich and titrated in THF at 0 °C with *s*-butanol and phenanthroline as an indicator prior to use.
(*E*)-chalcone was purchased from Ambeed and used as received.
cyclopropanecarboxaldehyde was purchased from Combi-Blocks and used as received.
***N,N*-diisopropylethylamine (DIPEA)** was purchased from Oakwood and distilled over CaH_2 before use.
***N*,*O*-dimethylhydroxylamine hydrochloride** was purchased from Ambeed and used as received.

diisobutylaluminum hydride solution (1.0 M in toluene) was purchased from Sigma-Aldrich and used as received.
di-*tert*-butyl dicarbonate was purchased from Oakwood and used as received.
hydrochloric acid 36.5% - 38.0% (HCl) was purchased from Macron Fine Chemicals and used as received.
isobutylmagnesium bromide solution (2M in ether) was purchased from Sigma-Aldrich and titrated in THF at 0 °C with *s*-butanol and 1,10-phenanthroline before use.
lithium bis(trimethylsilyl)amide solution (1.0 M in THF) was purchased from Sigma-Aldrich and titrated in THF at 0 °C with iodine and lithium chloride before use.
mesitaldehyde was purchased from Alfa Aesar and distilled before use.
methanesulfonyl chloride was purchased from Sigma-Aldrich and used as received.
methyl isobutyrate was purchased from Sigma-Aldrich and used as received.
methyl 2-oxo-2-phenylacetate was purchased from Ambeed and used as received.
***N*-methyl-2-pyrrolecarboxaldehyde** was purchased from AK Scientific and used as received.
methyl 4-formylbenzoate was purchased from Combi-Blocks and used as received.
phenylethyl magnesium bromide was purchased from Sigma-Aldrich and titrated in THF at 0 °C with *s*-butanol and 1,10-phenanthroline before use.
piperonal was purchased from Sigma-Aldrich and used as received.
pyrazole-3-carboxaldehyde was purchased from Combi-Blocks and used as received.
pyrrole-2-carboxaldehyde was purchased from Oakwood and used as received.
quinoline-4-carbaldehyde was purchased from Combi-Blocks and used as received.
sodium hydride (60% dispersion in mineral oil) was purchased from Sigma-Aldrich and used as received.
thiophene-2-carbaldehyde was purchased from Sigma-Aldrich and used as received.
triethylamine (Et_3N) was purchased from VWR and distilled over CaH_2 before use.
triethylsilane was purchased from Oakwood and used as received.
trimethylaluminum (2.0 M in toluene) was purchased from Sigma-Aldrich and used as received.
NHC precursors are synthesized according to the literature. [2]

3. Substrate Synthesis:

3.1 Bicyclobutane aldehyde synthesis:

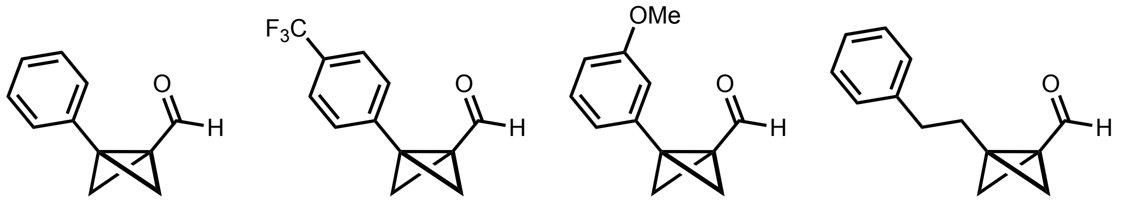

1 **SI-1** **SI-2** **SI-3**

1, **SI-1**, **SI-2**, **SI-3** were prepared using aryl bromides according to the literature procedure.[3]

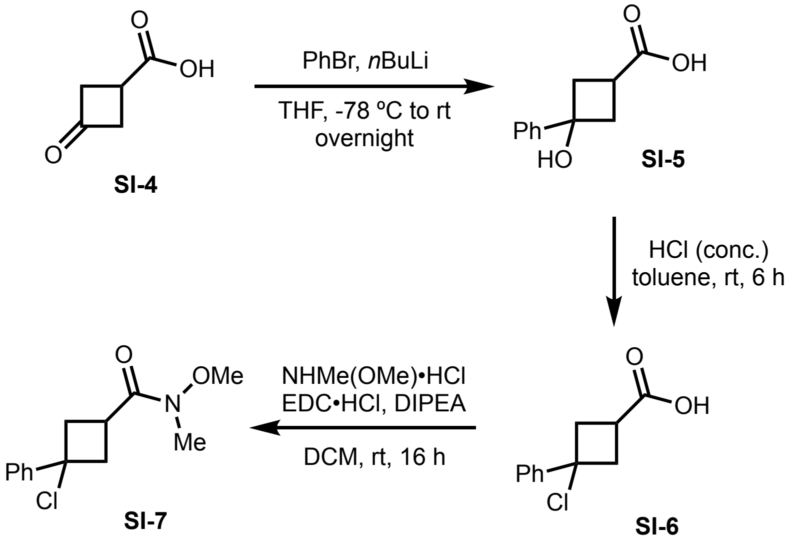

3-chloro-*N*-methoxy-*N*-methyl-3-phenylcyclobutane-1-carboxamide (SI-7):

A flame-dried 100 mL round-bottom flask equipped with a stir bar and septum was cooled under vacuum. After evacuating and backfilling with N_2 three times, bromobenzene (11.5 mL, 110 mmol, 2.20 equiv.) and THF (0.440 M, 114 mL) were added. The solution was cooled to -78 ºC in an acetone/dry ice bath. *n*-Butyllithium (2.50 M in hexane, 44.0 mL, 110 mmol, 2.20 equiv.) was added slowly at –78 °C and stirred for 30 minutes. 3-oxocyclobutanecarboxylic acid **SI-4** (5.71 g, 50.0 mmol, 1.00 equiv.) in THF (10 mL) was added slowly to the solution. The acetone/dry ice bath was then removed and the reaction was stirred overnight at room temperature. Upon completion, the reaction was quenched with saturated NH_4Cl solution (100 mL) and basified with 2M NaOH solution (200 mL). The mixture was washed with Et_2O (200 mL). The aqueous layer was collected and acidified with 6M HCl solution until the pH reached 1. The mixture was extracted with EtOAc three times (200 mL × 3). The combined organic layers were washed with brine (300 mL), dried over anhydrous Na_2SO_4, gravity-filtered, and concentrated via rotary evaporation. The crude **SI-5** was used directly to the next step without further purification.

A 250 mL round bottom flask equipped with a magnetic stir bar was charged with above crude **SI-5** (assuming 50.0 mmol, 1.00 equiv.), concentrated HCl solution (100 mL) and toluene (100 mL). The flask was capped with a septum. The reaction was stirred vigorously for 6 hours at room temperature. Upon completion, the mixture was extracted with Et_2O three times (100 mL × 3). The combined organic layers were washed with water (200 mL) and brine (200 mL), dried over anhydrous Na_2SO_4, gravity-filtered, and concentrated via rotary evaporation. The crude **SI-6** was used directly to the next step without further purification.

A 500 mL round bottom flask equipped with a magnetic stir bar was charged with the above crude **SI-6** (assuming 50.0 mmol, 1.00 equiv.), *N*,*O*-dimethylhydroxylamine hydrochloride (4.88 g, 50.0 mmol, 1.00 equiv.), DCM (0.500 M, 100 mL). The flask was capped with a septum and was cooled to 0 °C in a water/ice bath. *N*,*N*-Diisopropylethylamine (17.4 mL, 100 mmol, 2.00 equiv.) was added slowly and stirred for 5 minutes. EDC•HCl (11.5 g, 60.0 mmol, 1.20 equiv.) was added portion-wise to the solution. The water/ice bath was removed, and the reaction was stirred at room temperature for 16 hours. Upon completion, the solution was diluted with H_2O (50 mL) and extracted with Et_2O three times (100 mL × 3). The combined organic layers were washed with brine (100 mL), dried over anhydrous Na_2SO_4, gravity-filtered, and concentrated via rotary evaporation. The residue was purified by FCC (40% EtOAc in hexanes to afford the desired amide **SI-7** (10 g, 79% yield over 3 steps) as a pale-yellow oil. The characterization is consistent with the literature.[Error! Bookmark not defined.]

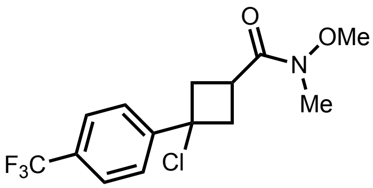

SI-8

3-chloro-*N*-methoxy-*N*-methyl-3-(4-(trifluoromethyl)phenyl)cyclobutane-1-carboxamide (SI-8) was synthesized using 4-bromobenzotrifluoride (3.10 mL, 22.0 mmol, 2.20 equiv.). The crude was purified by FCC (40% EtOAc in hexanes) to afford **SI-8** (1.8 g, 57% yield over 3 steps) as a pale-yellow oil. The characterization is consistent with the literature.[4]

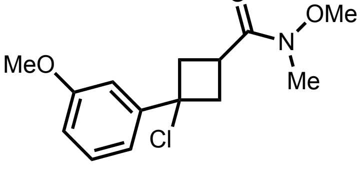

SI-9

3-chloro-*N*-methoxy-3-(3-methoxyphenyl)-*N*-methylcyclobutane-1-carboxamide (SI-9) was synthesized using 3-bromoanisole (2.79 mL, 22.0 mmol, 2.20 equiv.). The crude was purified by

FCC (40% EtOAc in hexanes) to afford **SI-9** (2.0 g, 70% yield over 3 steps) as a pale-yellow oil. The characterization is consistent with the literature.[5]

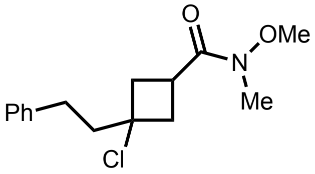

SI-10

3-chloro-*N*-methoxy-*N*-methyl-3-phenethylcyclobutane-1-carboxamide (SI-10) was synthesized using phenylethyl magnesium bromide (0.770 M in THF, 88.6 mL, 68.2 mmol, 2.20 equiv.). The crude was purified by FCC (40% EtOAc in hexanes) to afford **SI-10** (3.5 g, 40% yield, 1.5:1 dr over 3 steps) as a pale-yellow oil.

^{1}H NMR (500 MHz, $CDCl_3$): δ 7.34 – 7.26 (m, 3H), 7.26 – 7.16 (m, 5H), 3.90 – 3.76 (m, 1H, major), 3.66 (s, 3H, major), 3.63 (s, 1.84H, minor), 3.19 (s, 1.87H, minor), 2.93 – 2.83 (m, 3H, major), 2.81 – 2.74 (m, 2.61H), 2.62 – 2.51 (m, 5.33H), 2.28 – 2.20 (m, 1H, minor), 2.20 – 2.13 (m, 2H, major).
^{13}C NMR (126 MHz, $CDCl_3$): δ 174.9 (major), 141.6 (minor), 141.4 (major), 128.7 (major), 128.64 (minor), 128.56 (minor), 128.5 (major), 126.2 (minor), 126.1 (major), 71.1, 65.9, 61.61, 61.57, 45.9, 44.1, 41.32 (minor), 41.26 (major), 32.5, 31.5 (minor), 32.0 (major), 30.6, 30.3.
HRMS (ESI, *m/z*): Calcd for $C_{15}H_{21}O_2NCl^+$ $[M+H]^+$: 282.1255, found: 282.1249.

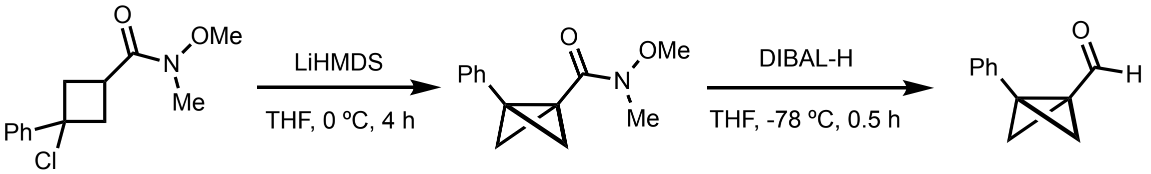

SI-7 **SI-11** **1**

3-phenylbicyclo[1.1.0]butane-1-carbaldehyde (1):

A flame-dried 100 mL round-bottom flask equipped with a stir bar and septum was cooled under vacuum. After evacuating and backfilling three times with N_2, **SI-7** (4.6 g, 18.0 mmol, 1.00 equiv.) and THF (0.33 M, 55 mL) were added via syringes. The solution was cooled to 0 °C in a water/ice bath and LiHMDS (1.0 M in THF, 22 mL, 22 mmol, 1.2 equiv.) was added slowly via syringe. The mixture was stirred for 4 hours at the same temperature. Upon completion, the reaction was quenched with saturated NH_4Cl solution (100 mL). Then the resulting mixture was extracted with EtOAc three times (100 mL × 3). The combined organic layers were washed with brine (200 mL), dried over anhydrous Na_2SO_4, gravity-filtered, and concentrated via rotary evaporation. The crude **SI-10** was used directly in the next step without further purification.

A flame-dried 500 mL round-bottom flask equipped with a magnetic stir bar was sealed with a septum and cooled under vacuum. The crude solid **SI-11** (assuming 18.0 mmol, 1.00 equiv.) was

transferred to the round-bottom flask. After backfilling three times with N_2, THF (0.100 M, 180 mL) was added via syringes. The solution was cooled to -78 °C in an acetone/dry ice bath. DIBAL-H (1.00 M in toluene, 19.8 mL, 19.8 mmol, 1.10 equiv.) was added dropwise via a syringe, and the reaction was stirred at the same temperature for 30 minutes. Upon completion, the reaction is quenched with saturated Rochelle salt solution (50 mL). The mixture was extracted with EtOAc three times (50 mL × 3). The combined organic layers were washed with brine (100 mL), dried over anhydrous Na_2SO_4, gravity-filtered, and concentrated via rotary evaporation. The residue was purified by FCC (20% EtOAc in hexanes) to afford the desired **1** (2.1 g, 74% yield over 2 steps) as a white solid.

^{1}H NMR (500 MHz, CDCl$_3$): δ 9.14 (s, 1H), 7.33 – 7.29 (m, 4H), 7.27 – 7.23 (m, 1H), 3.15 (t, *J* = 1.5 Hz, 2H), 1.78 (t, *J* = 1.5 Hz, 2H).
^{13}C NMR (126 MHz, CDCl$_3$): δ 195.6, 132.2, 128.9, 127.7, 126.1, 39.7, 36.0, 33.8.
HRMS (EI, *m*/*z*): Calcd for $C_{11}H_{10}O^+$ $[M]^+$: 158.1732, found: 158.0726.

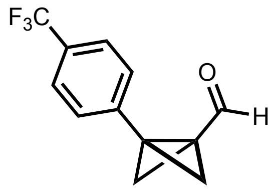

SI-1

3-(4-(trifluoromethyl)phenyl)bicyclo[1.1.0]butane-1-carbaldehyde (SI-1) was synthesized using **SI-8** (1.29 g, 4.00 mmol, 1.00 equiv.). The crude was purified by FCC (20% EtOAc in hexanes) to afford **SI-1** (428 mg, 68% yield over 2 steps) as a pale yellow oil.
^{1}H NMR (500 MHz, CDCl$_3$): δ 9.16 (s, 1H), 7.56 (d, *J* = 8.1 Hz, 2H), 7.40 (d, *J* = 8.1 Hz, 2H), 3.17 (t, *J* = 1.6 Hz, 2H), 1.84 (t, *J* = 1.6 Hz, 2H).
^{13}C NMR (126 MHz, CDCl$_3$): δ 194.9, 136.8, 129.7 (q, *J* = 32.8 Hz, C), 126.3, 125.9 (q, *J* = 3.8 Hz, CH), 124.1 (q, *J* = 272.2 Hz, CF_3), 37.8, 36.2, 34.3.
^{19}F NMR (470 MHz, CDCl$_3$): δ -62.2.
HRMS (EI, *m*/*z*): Calcd for $C_{12}H_9OF_3^+$ $[M]^+$: 226.0605, found: 226.0601.

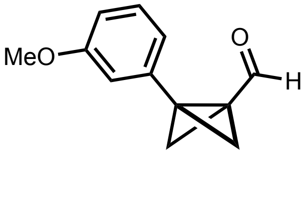

SI-2

3-(3-methoxyphenyl)bicyclo[1.1.0]butane-1-carbaldehyde (SI-2) was synthesized using **SI-9** (1.14 g, 4.00 mmol, 1.00 equiv.). The crude was purified by FCC (20% EtOAc in hexanes) to afford **SI-2** (405 mg, 64% yield over 2 steps) as a pale yellow oil.

^{1}H NMR (500 MHz, CDCl$_3$): δ 9.13 (s, 1H), 7.21 (t, J = 7.9 Hz, 1H), 6.89 (ddd, J = 7.7, 1.7, 0.9 Hz, 1H), 6.83 (t, J = 2.1 Hz, 1H), 6.79 (ddd, J = 8.2, 2.5, 0.9 Hz, 1H), 3.78 (s, 3H), 3.12 (t, J = 1.6 Hz, 2H), 1.76 (t, J = 1.5 Hz, 2H).
^{13}C NMR (126 MHz, CDCl$_3$): δ 195.5, 160.0, 133.8, 129.9, 118.4, 113.1, 112.0, 55.4, 39.4, 36.0, 33.8.
HRMS (EI, *m*/*z*): Calcd for $C_{12}H_{12}O_2^+$ [M]$^+$: 188.0837, found: 188.0831.

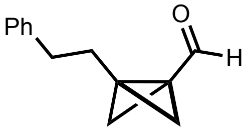

SI-3

3-phenethylbicyclo[1.1.0]butane-1-carbaldehyde (SI-3) was synthesized using **SI-10** (1.41 g, 5.00 mmol, 1.00 equiv.). The crude was purified by FCC (20% EtOAc in hexanes) and further recrystallization with EtOAc/pentane to afford **SI-3** (554 mg, 60% yield over 2 steps) as a pale yellow oil.

^{1}H NMR (500 MHz, CDCl$_3$): δ 9.39 (s, 1H), 7.31 – 7.26 (m, 2H), 7.23 – 7.18 (m, 1H), 7.18 – 7.14 (m, 2H), 2.75 (t, J = 8.0 Hz, 2H), 2.40 (s, 2H), 2.22 (t, J = 8.0 Hz, 2H), 1.37 (t, J = 1.2 Hz, 2H).
^{13}C NMR (126 MHz, CDCl$_3$): δ 196.8, 140.6, 128.6, 128.5, 126.4, 38.9, 37.9, 35.1, 29.5, 25.0.
HRMS (ESI, *m*/*z*): Calcd for $C_{13}H_{14}ONa^+$ [M+Na]$^+$: 209.0937, found: 209.0933.

3.2 Isatin Imine Synthesis:

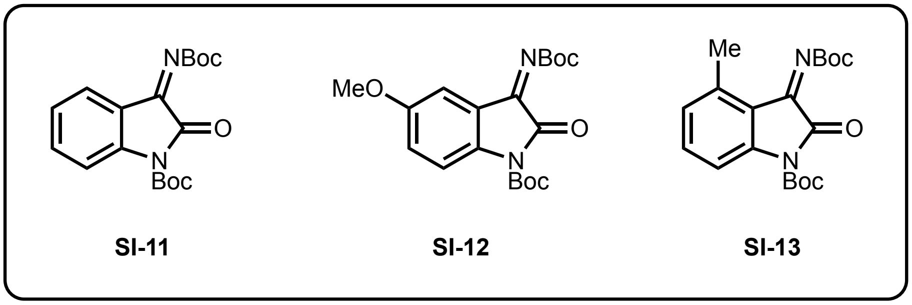

SI-11, SI-12, and **SI-13** were prepared according to a modified literature procedure by Wang and co-workers.[6]

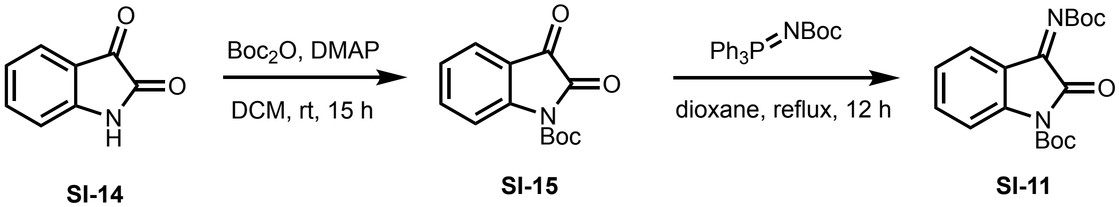

tert-butyl (_Z_)-3-((_tert_-butoxycarbonyl)imino)-2-oxoindoline-1-carboxylate (SI-11):

A 250 mL flask equipped with a stir bar, **SI-14** (2.94 g, 20.0 mmol, 1.00 equiv.) and DMAP (0.12 g, 1.0 mmol, 0.0050 equiv.) was sealed with the septum. After vacuuming and backfilling three times with N_2, THF (0.200 M, 100 mL) was added via a syringe. Boc_2O (4.80 g, 22.0 mmol, 1.10 equiv.) was added portionwise. The mixture was stirred at room temperature for 15 hours. Upon completion, brine (50 mL) was added to the reaction. The resulting mixture was extracted with EtOAc three times (50 mL × 3). The combined organic layers were dried over anhydrous Na_2SO_4, gravity-filtered, and concentrated via rotary evaporation. The crude **SI-15** was used directly in the next step without further purification.

A 250 mL flask equipped with a reflux condenser, stir bar, crude **SI-15** (assuming 20.0 mmol, 1.00 equiv.), and _N_-Boc-imino-(triphenyl)phosphorane (8.30 g, 22.0 mmol, 1.10 equiv.) was sealed with the septum. After vacuuming and backfilling three times with N_2, dioxane (1.0 M, 20 mL) was added via a syringe. The mixture was stirred under reflux for 12 hours. Upon completion, the mixture was concentrated via rotary evaporation. The residue was purified by FCC (20% EtOAc in hexanes) to afford the desired **SI-11** (5.9 g, 86% yield over 2 steps) as a yellow solid. The characterization is consistent with the literature.[6]

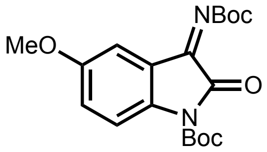

SI-12

tert-butyl (_E_)-3-((_tert_-butoxycarbonyl)imino)-5-methoxy-2-oxoindoline-1-carboxylate (SI-12) was synthesized using 5-methoxyindoline-2,3-dione (886 mg, 5.00 mmol, 1.00 equiv.). The crude was purified by FCC (10% EtOAc in hexanes) to afford **SI-12** (0.99 g, 73% yield over 2 steps) as a yellow solid. The characterization is consistent with the literature.[6]

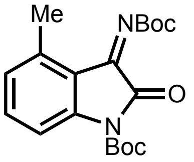

SI-13

tert-butyl (_E_)-3-((_tert_-butoxycarbonyl)imino)-4-methyl-2-oxoindoline-1-carboxylate (SI-12)

was synthesized using 4-methylindoline-2,3-dione (677 mg, 4.20 mmol, 1.00 equiv.). The crude was purified by FCC (10% EtOAc in hexanes) to afford **SI-13** (0.57 g, 55% yield over 2 steps) as a yellow solid. The characterization is consistent with the literature.[6]

4. Optimization Studies:

General procedure for condition optimization: An oven-dried 1-dram vial equipped with a stir bar was cooled under vacuum. **1** (15.8 mg, 0.100 mmol, 1.00 equiv.) and NHC precursor (0.01 mmol, 0.1 equiv.) were added. The vial was then sealed with a septum placed under vacuum. After evacuating and backfilling three times with N_2, the solvent (0.2 mL, 0.5 M) was added to the reaction vial via a syringe, followed by the addition of benzaldehyde via a syringe. Then the base (0.01 mmol, 0.1 equiv.) was then added via the syringe. The septum on the reaction vial was quickly replaced by a screw cap and the reaction was stirred at room temperature for 22 hours. Upon completion, the solvent was removed by rotary evaporation. CH_2Br_2 (7 µL, 0.1 mmol) was added as an internal standard for crude ^{1}H NMR analysis to determine the yield.

If the base was solid, it was weighed out with **1**. For imine, it was also weighed with **1** if it was a solid.

4.1 NHC catalyst screening of aldehyde:

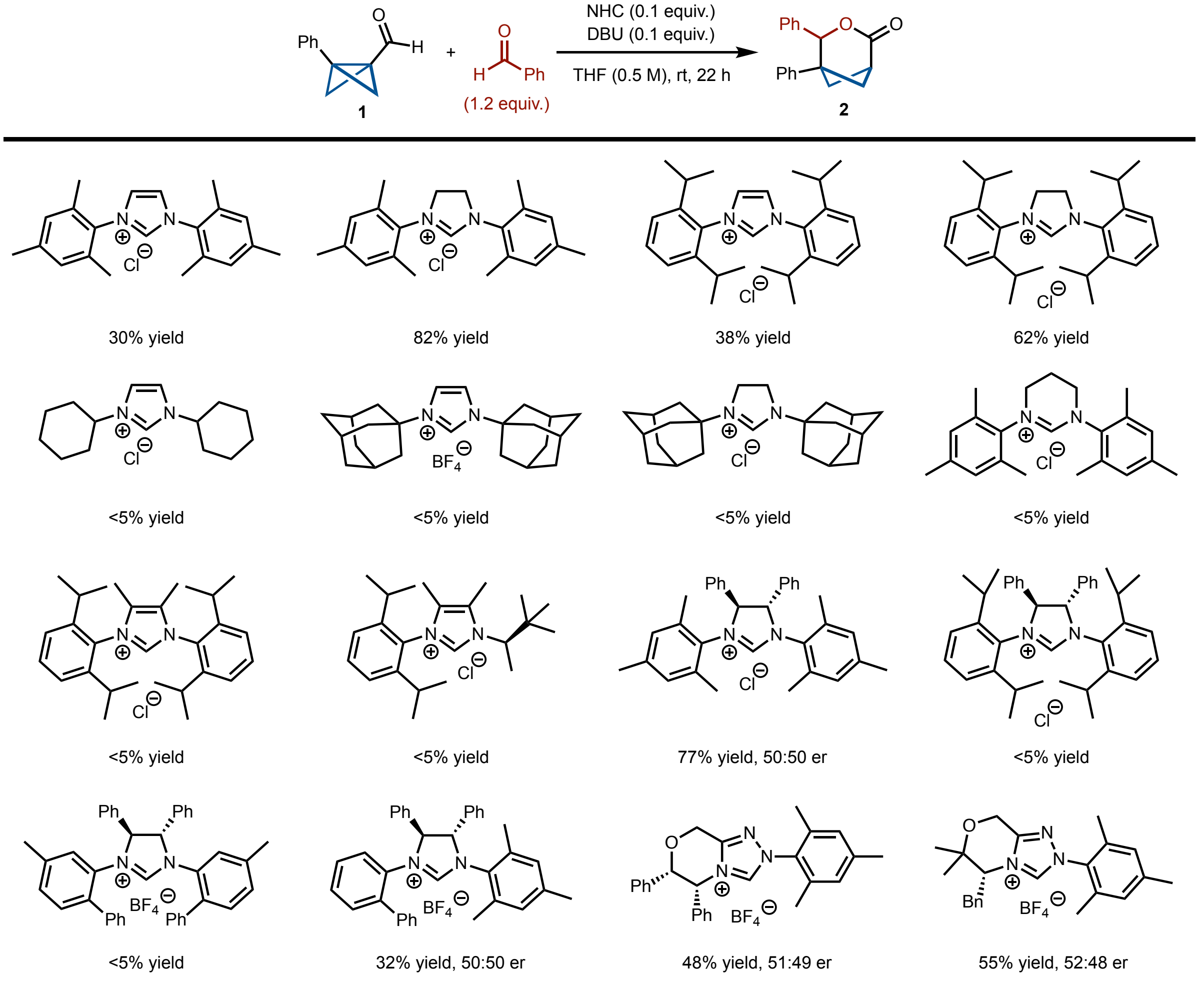

All reactions are performed on 0.1 mmol scale. Yield was determined by ^{1}H NMR analysis of the unpurified reaction mixture using CH_2Br_2 as internal standard.

4.2 Base and solvent screening of aldehyde:

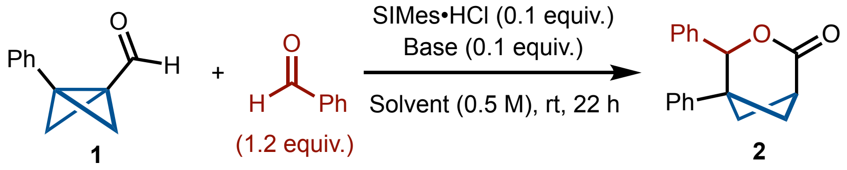

entry	base	solvent	yield (%)[a]
1	DBU	THF	<5%
2	Et_3N	THF	<5%
3	DIPEA	THF	<5%
4	K_2CO_3	THF	<5%
5	KOAc	THF	<5%
6	Cs_2CO_3	THF	<5%
7	DBU	DCM	68%
8	DBU	MeCN	30%
9	DBU	dioxane	78%
10	DBU	toluene	66%
11	DBU	*t*BuOH	52%

[a] All reactions are performed on 0.1 mmol scale. Yield was determined by ^{1}H NMR analysis of the unpurified reaction mixture using CH_2Br_2 as internal standard.

4.3 Equivalence of aldehyde screening:

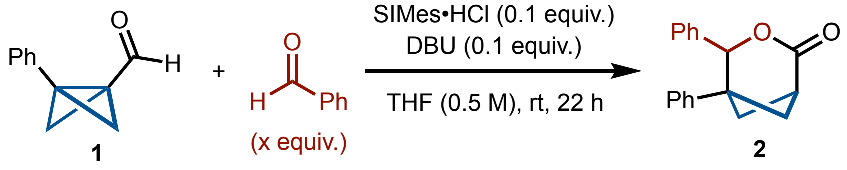

entry	x equiv. of benzaldehyde	yield (%)[a]
1	1.2	82%
2	1.5	86%
3	2	88%

[a] All reactions are performed on 0.1 mmol scale. Yield was determined by ^{1}H NMR analysis of the unpurified reaction mixture using CH_2Br_2 as internal standard.

4.4 Base and solvent screening for imine:

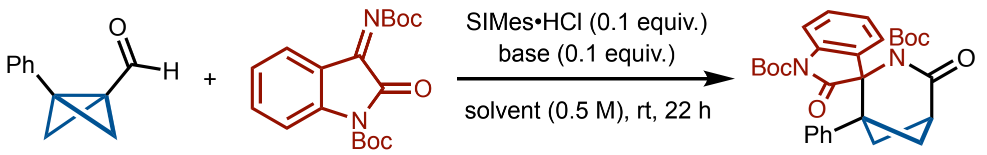

1 (1.2 equiv.) **26**

entry	base	solvent	yield (%)[a]
1	DBU	THF	41%
2	DBU	DCM	6%
3	DBU	MeCN	11%
4	DBU	DME	30%
5	DBU	dioxane	35%
6	DBU	toluene	23%
7	Et_3N	THF	<5%
8	DIPEA	THF	<5%
9	K_2CO_3	THF	<5%
10	KOAc	THF	<5%
11	Cs_2CO_3	THF	<5%

[a] All reactions are performed on 0.1 mmol scale. Yield was determined by ^{1}H NMR analysis of the unpurified reaction mixture using CH_2Br_2 as internal standard.

4.5 Equivalence of DBU screening:

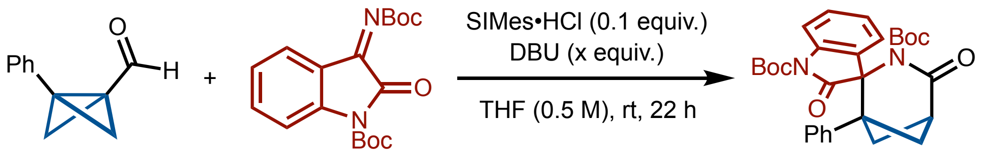

1 (1.2 equiv.) **26**

entry	x equiv. of DBU	yield (%)[a]
1	0.1	41%
2	0.5	45%
3	1.0	68%
4	2.0	66%

[a] All reactions are performed on 0.1 mmol scale. Yield was determined by ^{1}H NMR analysis of the unpurified reaction mixture using CH_2Br_2 as internal standard.

5. Experimental Procedure for Strain-Release Cycloaddition:

5.1 General procedure for cycloaddition with carbonyl-containing partners:

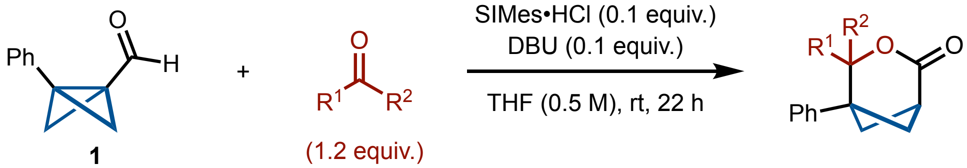

An oven-dried 1-dram vial equipped with a stir bar was cooled under vacuum. **1** (31.6 mg, 0.200 mmol, 1.00 equiv.) and SIMes•HCl (6.9 mg, 0.020 mmol, 0.10 equiv.) were added. The vial was then sealed with a septum placed under vacuum. After evacuating and backfilling three times with N_2, THF (0.4 mL, 0.5 M) was added to the reaction vial via a syringe, followed by the addition of aldehyde or ketone (0.24 mmol, 1.2 equiv.) via a syringe. Then DBU (3 µL, 0.02 mmol, 0.1 equiv.) was added via the syringe. The septum on the reaction vial was quickly replaced by a screw cap and the reaction was stirred at room temperature for 22 hours. Upon completion, the solvent was removed by rotary evaporation. CH_2Br_2 (14 µL, 0.2 mmol) was added as an internal standard for crude ^{1}H NMR analysis to determine the yield. The crude was purified by FCC to give the corresponding cycloadducts.

If the aldehyde or ketone was solid, they were weighed out with **1** and SIMes•HCl.

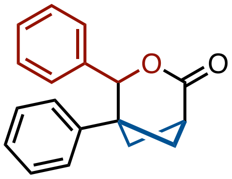

2

4,5-diphenyl-3-oxabicyclo[3.1.1]heptan-2-one (2): The title compound was prepared according to the general procedure using **1** (31.6 mg, 0.200 mmol, 1.00 equiv.) and benzaldehyde (25 µL, 0.24 mmol, 1.2 equiv.). The crude was purified by FCC (5% to 15% EtOAc in hexanes) to afford **2** (42 mg, 79% yield) as a white solid.

^{1}H NMR (500 MHz, CDCl$_3$): δ 7.25 – 7.18 (m, 4H), 7.13 (td, J = 6.7, 1.6 Hz, 2H), 6.78 – 6.69 (m, 4H), 5.64 (s, 1H), 3.19 – 3.11 (m, 1H), 2.65 – 2.57 (m, 2H), 2.54 – 2.48 (m, 1H), 2.35 (dd, J = 9.9, 6.1 Hz, 1H).
^{13}C NMR (126 MHz, CDCl$_3$): δ 174.5, 142.2, 136.3, 128.4, 128.1, 127.6, 127.4, 127.0, 126.2, 87.1, 50.7, 37.6, 36.6, 29.1.
HRMS (ESI, *m/z*): Calcd for $C_{18}H_{16}O_2Na^+$ $[M+Na]^+$: 287.1043, found: 287.1039.
IR: 2951 (w), 1753 (s), 1316 (m), 1189 (m), 1070 (m), 1021 (s), 764 (m), 699 (m).
m.p.: 129 – 131 °C.

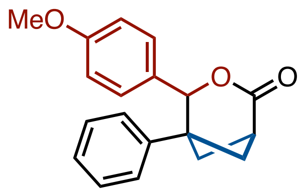

3

4-(4-methoxyphenyl)-5-phenyl-3-oxabicyclo[3.1.1]heptan-2-one (3): The title compound was prepared according to the general procedure using **1** (31.6 mg, 0.200 mmol, 1.00 equiv.) and 4-methoxybenzaldehyde (33 µL, 0.24 mmol, 1.2 equiv.). The crude was purified by FCC (10% to 20% EtOAc in hexanes) to afford **3** (36 mg, 61% yield) as a white solid.

^{1}H NMR (500 MHz, $CDCl_3$): δ 7.22 (dd, J = 5.4, 1.5 Hz, 3H), 6.77 – 6.71 (m, 2H), 6.66 (s, 4H), 5.60 (s, 1H), 3.75 (s, 3H), 3.17 – 3.10 (m, 1H), 2.62 – 2.56 (m, 2H), 2.51 – 2.46 (m, 1H), 2.34 (dd, J = 9.8, 6.0 Hz, 1H).
^{13}C NMR (126 MHz, $CDCl_3$): δ 174.6, 159.4, 142.3, 128.5, 128.4, 128.1, 127.3, 126.2, 113.0, 86.9, 55.3, 50.8, 37.6, 36.5, 29.1.
HRMS (ESI, *m/z*): Calcd for $C_{19}H_{18}O_3Na^+$ $[M+Na]^+$: 317.1148, found: 317.1143.
IR: 2959 (w), 1751 (s), 1612 (m), 1513 (s), 1245 (s), 1177 (s), 1071 (s), 1031 (s), 1020 (s), 762 (m), 701 (m).
m.p.: 102 – 104 °C.

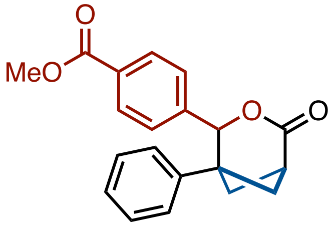

4

methyl 4-(4-oxo-1-phenyl-3-oxabicyclo[3.1.1]heptan-2-yl)benzoate (4): The title compound was prepared according to the general procedure using **1** (31.6 mg, 0.200 mmol, 1.00 equiv.) and methyl 4-formylbenzoate (39 mg, 0.24 mmol, 1.2 equiv.). The crude was purified by FCC (10% to 20% EtOAc in hexanes) to afford **4** (53 mg, 82% yield) as a white solid.

^{1}H NMR (500 MHz, $CDCl_3$): δ 7.80 (d, J = 8.5 Hz, 2H), 7.23 (dd, J = 4.9, 1.8 Hz, 3H), 6.80 (d, J = 8.4 Hz, 2H), 6.74 – 6.70 (m, 2H), 5.69 (s, 1H), 3.88 (s, 3H), 3.19 – 3.14 (m, 1H), 2.67 – 2.60 (m, 2H), 2.48 – 2.43 (m, 1H), 2.37 (dd, J = 10.1, 6.0 Hz, 1H).
^{13}C NMR (126 MHz, $CDCl_3$): δ 174.1, 166.8, 141.7, 141.4, 130.0, 128.9, 128.6, 127.6, 127.0, 126.1, 86.6, 52.3, 50.6, 37.5, 36.6, 29.0.
HRMS (ESI, *m/z*): Calcd for $C_{20}H_{19}O_4^+$ $[M+H]^+$: 323.1278, found: 323.1273.
IR: 2955 (w), 1756 (s), 1719 (s), 1435 (m), 1312 (m), 1278 (s), 1188 (m), 1108 (m), 1071 (m), 1019 (s), 770 (m), 701 (m).

m.p.: 100 – 102 °C.

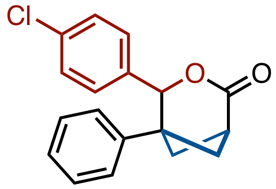

5

4-(4-chlorophenyl)-5-phenyl-3-oxabicyclo[3.1.1]heptan-2-one (5): The title compound was prepared according to the general procedure using **1** (31.6 mg, 0.200 mmol, 1.00 equiv.) and 4-chlorobenzaldehyde (34 mg, 0.24 mmol, 1.2 equiv.). The crude was purified by FCC (5% to 15% EtOAc in hexanes) to afford **5** (45 mg, 75% yield) as a white solid.

^{1}H NMR (500 MHz, CDCl$_3$): δ 7.25 (dd, *J* = 4.9, 1.8 Hz, 3H), 7.11 (d, *J* = 8.5 Hz, 2H), 6.74 (dd, *J* = 6.7, 2.9 Hz, 2H), 6.66 (d, *J* = 8.5 Hz, 2H), 5.62 (s, 1H), 3.17 – 3.13 (m, 1H), 2.64 – 2.58 (m, 2H), 2.45 – 2.40 (m, 1H), 2.36 (dd, *J* = 10.0, 5.9 Hz, 1H).
^{13}C NMR (126 MHz, CDCl$_3$): δ 174.2, 141.8, 134.9, 134.1, 128.6, 128.3, 127.9, 127.6, 126.2, 86.4, 50.6, 37.5, 36.5, 29.0.
HRMS (ESI, *m/z*): Calcd for $C_{18}H_{15}O_2ClNa^+$ [M+Na]$^+$: 321.0653, found: 321.0649.
IR: 2989 (w), 1756 (s), 1491 (m), 1314 (m), 1188 (m), 1108 (m), 1072 (m), 1020 (m), 807 (m), 761 (m), 700 (m).
m.p.: 82 – 84 °C.

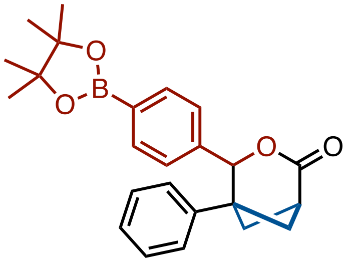

6

5-phenyl-4-(4-(4,4,5,5-tetramethyl-1,3,2-dioxaborolan-2-yl)phenyl)-3-oxabicyclo[3.1.1]heptan-2-one (6): The title compound was prepared according to the general procedure using **1** (31.6 mg, 0.200 mmol, 1.00 equiv.) and 4-(4,4,5,5-tetramethyl-1,3,2-dioxaborolan-2-yl)benzaldehyde (56 mg, 0.24 mmol, 1.2 equiv.). The crude was purified by FCC (5% to 15% EtOAc in hexanes) to afford **6** (69 mg, 88% yield) as a white solid.

^{1}H NMR (500 MHz, CDCl$_3$): δ 7.56 (d, *J* = 8.2 Hz, 2H), 7.22 (dd, *J* = 5.0, 1.8 Hz, 3H), 6.77 – 6.69 (m, 4H), 5.66 (s, 1H), 3.14 (t, *J* = 5.4 Hz, 1H), 2.64 – 2.57 (m, 2H), 2.48 (dd, *J* = 9.9, 7.2 Hz, 1H), 2.34 (dd, *J* = 10.0, 6.0 Hz, 1H), 1.33 (s, 6H), 1.32 (s, 6H).

^{13}C NMR (126 MHz, CDCl$_3$): δ 174.5, 142.0, 139.3, 134.0, 128.5, 127.4, 126.3, 126.2, 87.1, 84.0, 50.6, 37.6, 36.7, 29.0, 25.1, 24.9.
HRMS (ESI, *m/z*): Calcd for $C_{24}H_{27}O_4BNa^+$ [M+Na]$^+$: 413.1895, found: 413.1892.
IR: 2977 (m), 1755 (s), 1618 (m), 1399 (m), 1360 (s), 1320 (m), 1143 (m), 1090 (m), 1072 (m), 1019 (m), 858 (m), 774 (m), 735 (m), 712 (m), 656 (m).
m.p.: 165 – 167 °C.

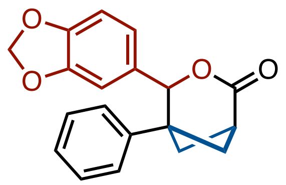

7

4-(benzo[*d*][1,3]dioxol-5-yl)-5-phenyl-3-oxabicyclo[3.1.1]heptan-2-one (7): The title compound was prepared according to the general procedure using **1** (31.6 mg, 0.200 mmol, 1.00 equiv.) and piperonal (36 mg, 0.24 mmol, 1.2 equiv.). The crude was purified by FCC (10% to 20% EtOAc in hexanes) to afford **7** (40 mg, 65% yield) as a white solid.

^{1}H NMR (500 MHz, CDCl$_3$): δ 7.26 – 7.21 (m, 3H), 6.80 – 6.72 (m, 2H), 6.56 (d, *J* = 8.1 Hz, 1H), 6.27 (d, *J* = 1.8 Hz, 1H), 6.18 (ddd, *J* = 8.1, 1.8, 0.6 Hz, 1H), 5.90 (q, *J* = 1.5 Hz, 2H), 5.55 (s, 1H), 3.13 (ddd, *J* = 6.1, 3.8, 2.3 Hz, 1H), 2.62 – 2.54 (m, 2H), 2.53 – 2.46 (m, 1H), 2.36 (dd, *J* = 9.9, 6.1 Hz, 1H).
^{13}C NMR (126 MHz, CDCl$_3$): δ 174.4, 147.4, 147.2, 142.2, 130.2, 128.5, 127.4, 126.2, 120.7, 107.5, 107.5, 101.2, 87.0, 50.8, 37.5, 36.6, 29.1.
HRMS (ESI, *m/z*): Calcd for $C_{19}H_{17}O_4^+$ [M+H]$^+$: 309.1121, found: 309.1115.
IR: 2993 (w), 2877 (w), 1750 (s), 1504 (m), 1489 (m), 1446 (m), 1314 (m), 1240 (m), 1190 (m), 1070 (m), 1035 (s), 1019 (s), 930 (m), 761 (m), 701 (m).
m.p.: 118 – 120 °C.

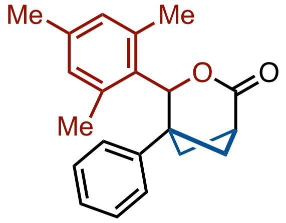

8

4-mesityl-5-phenyl-3-oxabicyclo[3.1.1]heptan-2-one (8): The title compound was prepared according to the general procedure using **1** (31.6 mg, 0.200 mmol, 1.00 equiv.) and mesitaldehyde (35 µL, 0.24 mmol, 1.2 equiv.). The crude was purified by FCC (10% to 20% EtOAc in hexanes) to afford **8** (15 mg, 24% yield) as a white solid.

^{1}H NMR (500 MHz, CDCl$_3$): δ 7.17 (t, J = 7.3 Hz, 1H), 7.12 (t, J = 7.3 Hz, 2H), 6.87 (s, 1H), 6.68 (d, J = 7.1 Hz, 2H), 6.46 (s, 1H), 6.03 (s, 1H), 3.15 (t, J = 5.9 Hz, 1H), 2.85 (dd, J = 10.1, 8.3 Hz, 1H), 2.75 (dd, J = 9.2, 5.8 Hz, 1H), 2.61 (d, J = 8.2 Hz, 4H), 2.26 (dd, J = 10.1, 5.9 Hz, 1H), 2.20 (s, 3H), 1.30 (s, 3H).
^{13}C NMR (126 MHz, CDCl$_3$): δ 174.4, 142.4, 138.3, 137.4, 136.0, 132.3, 129.4, 128.0, 127.2, 126.8, 85.4, 52.4, 38.2, 37.0, 32.5, 23.4, 20.8, 20.6.
HRMS (ESI, *m/z*): Calcd for $C_{21}H_{22}O_2Na^+$ [M+Na]$^+$: 329.1512, found: 329.1507.
IR: 2948 (m), 1750 (s), 1610 (m), 1447 (m), 1314 (m), 1205 (m), 1069 (m), 1036 (m), 1020 (m), 761 (m), 700 (m).
m.p.: 163 – 165 °C.

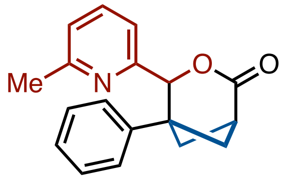

9

4-(6-methylpyridin-2-yl)-5-phenyl-3-oxabicyclo[3.1.1]heptan-2-one (9): The title compound was prepared according to the general procedure using **1** (31.6 mg, 0.200 mmol, 1.00 equiv.) and 6-methylpicolinaldehyde (29 mg, 0.24 mmol, 1.2 equiv.). The crude was purified by FCC (10% to 30% EtOAc in hexanes) to afford **9** (9 mg, 16% yield) as a colorless oil.

^{1}H NMR (500 MHz, CDCl$_3$): δ 7.36 (t, J = 7.7 Hz, 1H), 7.16 (dd, J = 5.4, 1.9 Hz, 3H), 6.97 (d, J = 7.7 Hz, 1H), 6.74 – 6.70 (m, 2H), 6.69 (d, J = 7.7 Hz, 1H), 5.56 (s, 1H), 3.19 – 3.04 (m, 2H), 2.65 – 2.56 (m, 2H), 2.37-2.34 (m, 4H).
^{13}C NMR (126 MHz, CDCl$_3$): δ 174.4, 157.8, 155.1, 142.3, 136.1, 128.2, 127.0, 126.0, 122.7, 119.5, 86.8, 50.4, 37.6, 36.8, 29.7, 24.4.
HRMS (ESI, *m/z*): Calcd for $C_{18}H_{18}O_2N^+$ [M+H]$^+$: 280.1332, found: 280.1326.
IR: 2939 (w), 1751 (s), 1592 (m), 1458 (m), 1314 (m), 1192 (m), 1075 (m), 1038 (m), 1022 (m), 758 (m), 701 (m).
m.p.: 98 – 100 °C.

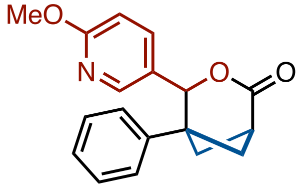

10

4-(6-methoxypyridin-3-yl)-5-phenyl-3-oxabicyclo[3.1.1]heptan-2-one (10): The title compound was prepared according to the general procedure using **1** (31.6 mg, 0.200 mmol, 1.00

equiv.) and 6-methoxynicotinaldehyde (33 mg, 0.24 mmol, 1.2 equiv.). The crude was purified by FCC (10% to 30% EtOAc in hexanes) to afford **10** (42 mg, 70% yield) as a white solid.

^{1}H NMR (500 MHz, CDCl$_3$): δ 7.56 (d, *J* = 2.5 Hz, 1H), 7.24 (dd, *J* = 5.6, 1.7 Hz, 3H), 6.93 (dd, *J* = 8.7, 2.6 Hz, 1H), 6.80 – 6.72 (m, 2H), 6.51 (d, *J* = 8.6 Hz, 1H), 5.60 (s, 1H), 3.85 (s, 3H), 3.19 – 3.09 (m, 1H), 2.60 (dd, *J* = 5.0, 2.5 Hz, 2H), 2.47 – 2.36 (m, 2H).
^{13}C NMR (126 MHz, CDCl$_3$): δ 174.0, 164.0, 145.4, 141.6, 137.2, 128.7, 127.7, 126.1, 125.0, 109.8, 85.1, 53.6, 50.6, 37.4, 36.4, 29.0.
HRMS (ESI, *m/z*): Calcd for $C_{18}H_{18}O_3N^+$ [M+H]$^+$: 296.1281, found: 296.1274.
IR: 2950 (w), 1754 (s), 1606 (m), 1492 (s), 1395 (m), 1313 (m), 1285 (s), 1190 (m), 1072 (m), 1019 (s), 829 (m), 763 (m), 701 (m).
m.p.: 83 – 85 °C.

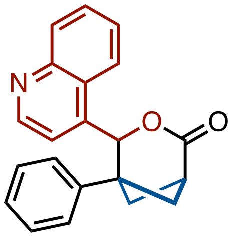

11

5-phenyl-4-(quinolin-4-yl)-3-oxabicyclo[3.1.1]heptan-2-one (11): The title compound was prepared according to the general procedure using **1** (31.6 mg, 0.200 mmol, 1.00 equiv.) and quinoline-4-carbaldehyde (38 mg, 0.24 mmol, 1.2 equiv.). The crude was purified by FCC (30% to 50% EtOAc in hexanes) to afford **11** (27 mg, 43% yield) as a white solid.

^{1}H NMR (500 MHz, CDCl$_3$): δ 8.97 (d, *J* = 4.6 Hz, 1H), 7.97 (d, *J* = 8.4 Hz, 1H), 7.54 (d, *J* = 4.6 Hz, 1H), 7.45 (ddd, *J* = 8.4, 6.6, 1.6 Hz, 1H), 7.02 – 6.87 (m, 5H), 6.55 (d, *J* = 7.2 Hz, 2H), 6.44 (s, 1H), 3.25 (ddd, *J* = 6.0, 3.9, 2.0 Hz, 1H), 2.83 – 2.77 (m, 2H), 2.73 (ddd, *J* = 10.1, 4.6, 2.8 Hz, 1H), 2.37 (dd, *J* = 10.0, 6.0 Hz, 1H).
^{13}C NMR (126 MHz, CDCl$_3$): δ 173.8, 149.6, 147.6, 142.3, 141.2, 129.7, 128.7, 128.3, 127.5, 126.2, 126.1, 125.9, 122.4, 119.2, 81.6, 50.8, 37.7, 36.5, 29.9.
HRMS (ESI, *m/z*): Calcd for $C_{21}H_{18}O_2N^+$ [M+H]$^+$: 316.1332, found: 316.1327.
IR: 2939 (w), 1755 (s), 1509 (m), 1312 (m), 1243 (m), 1186 (m), 1074 (m), 1036 (m), 1020 (m), 754 (m), 700 (m).
m.p.: 180 – 182 °C.

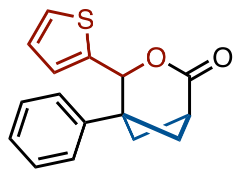

12

5-phenyl-4-(thiophen-2-yl)-3-oxabicyclo[3.1.1]heptan-2-one (12): The title compound was prepared according to the general procedure using **1** (31.6 mg, 0.200 mmol, 1.00 equiv.) and thiophene-2-carbaldehyde (22 µL, 0.24 mmol, 1.2 equiv.). The crude was purified by FCC (5% to 15% EtOAc in hexanes) to afford **12** (17 mg, 31% yield) as a white solid.

^{1}H NMR (500 MHz, CDCl$_3$): δ 7.29 – 7.22 (m, 3H), 7.17 (dd, J = 5.1, 1.2 Hz, 1H), 6.94 – 6.86 (m, 2H), 6.79 (dd, J = 5.1, 3.6 Hz, 1H), 6.40 (d, J = 3.7 Hz, 1H), 5.90 (s, 1H), 3.15 (t, J = 6.1 Hz, 1H), 2.67 – 2.49 (m, 4H).
^{13}C NMR (126 MHz, CDCl$_3$): δ 173.7, 141.7, 139.2, 128.6, 127.5, 126.6, 126.3, 126.0, 125.3, 84.1, 50.8, 37.5, 36.6, 30.0.
HRMS (ESI, *m/z*): Calcd for $C_{16}H_{15}O_2S^+$ [M+H]$^+$: 271.0787, found: 271.0783.
IR: 2976 (w), 1756 (s), 1338 (m), 1314 (m), 1245 (m), 1201 (m), 1153 (m), 1069 (m), 1036 (m), 1019 (m), 758 (m), 700 (m).
m.p.: 116 – 118 °C.

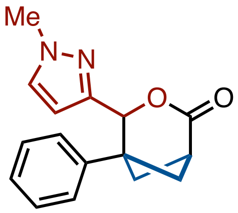

13

4-(1-methyl-1*H*-pyrazol-3-yl)-5-phenyl-3-oxabicyclo[3.1.1]heptan-2-one (13): The title compound was prepared according to the general procedure using **1** (31.6 mg, 0.200 mmol, 1.00 equiv.) and 1-methyl-1*H*-pyrazole-3-carbaldehyde (26 mg, 0.24 mmol, 1.2 equiv.). The crude was purified by FCC (40% to 60% EtOAc in hexanes) to afford **13** (23 mg, 43% yield) as a pale-yellow solid.

^{1}H NMR (500 MHz, CDCl$_3$): δ 7.30 (ddd, J = 14.0, 7.8, 6.2 Hz, 3H), 6.96 – 6.91 (m, 2H), 6.89 (s, 1H), 6.68 (s, 1H), 5.70 (s, 1H), 3.77 (s, 3H), 3.10 (td, J = 5.3, 1.6 Hz, 1H), 2.58 – 2.50 (m, 2H), 2.48 (dd, J = 9.9, 6.1 Hz, 1H), 2.29 (dd, J = 9.8, 7.3 Hz, 1H).
^{13}C NMR (126 MHz, CDCl$_3$): δ 174.3, 141.8, 137.5, 128.7, 128.6, 127.6, 126.2, 118.4, 81.4, 50.1, 39.0, 37.3, 36.3, 29.7.
HRMS (ESI, *m/z*): Calcd for $C_{16}H_{17}O_2N_2^+$ [M+H]$^+$: 269.1285, found: 269.1274.
IR: 2946 (w), 1748 (s), 1653 (m), 1506 (m), 1197 (m), 1074 (m), 1038 (m), 1018 (m), 763 (m),

701 (m).
m.p.: 122 – 124 °C.

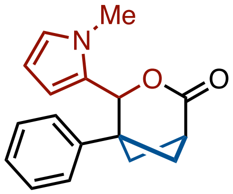

14

4-(1-methyl-1*H*-pyrrol-2-yl)-5-phenyl-3-oxabicyclo[3.1.1]heptan-2-one (14): The title compound was prepared according to the general procedure using **1** (31.6 mg, 0.200 mmol, 1.00 equiv.) and *N*-methyl-2-pyrrolecarboxaldehyde (26 µL, 0.24 mmol, 1.2 equiv.). The crude was purified by FCC (10% to 30% EtOAc in hexanes) to afford **14** (13 mg, 22% yield) as a colorless oil.

^{1}H NMR (500 MHz, CDCl$_3$): δ 7.25 – 7.19 (m, 3H), 6.85 (dd, *J* = 7.7, 1.9 Hz, 2H), 6.37 (t, *J* = 2.2 Hz, 1H), 6.22 (dd, *J* = 3.8, 1.7 Hz, 1H), 6.05 (dd, *J* = 3.8, 2.7 Hz, 1H), 5.63 (s, 1H), 3.12 (t, *J* = 5.9 Hz, 1H), 2.84 – 2.75 (m, 4H), 2.62 (dd, *J* = 9.4, 5.9 Hz, 1H), 2.55 (dd, *J* = 9.4, 7.8 Hz, 1H), 2.48 (dd, *J* = 9.8, 6.1 Hz, 1H).
^{13}C NMR (126 MHz, CDCl$_3$): δ 174.1, 142.1, 128.6, 127.6, 127.5, 126.1, 122.8, 108.7, 107.3, 79.9, 50.3, 37.5, 36.5, 33.4, 30.7.
HRMS (ESI, *m/z*): Calcd for $C_{17}H_{18}O_2N^+$ $[M+H]^+$: 268.1332, found: 268.1326.
IR: 2947 (w), 1747 (s), 1487 (m), 1313 (m), 1192 (m), 1071 (m), 1038 (m), 1016 (m), 759 (m), 701 (m).
m.p.: 117 – 119 °C.

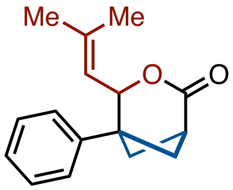

15

4-(2-methylprop-1-en-1-yl)-5-phenyl-3-oxabicyclo[3.1.1]heptan-2-one (15): The title compound was prepared according to the general procedure using **1** (31.6 mg, 0.200 mmol, 1.00 equiv.) and 3-methyl-2-butenal (23 µL, 0.24 mmol, 1.2 equiv.). The crude was purified by FCC (10% to 20% EtOAc in hexanes) to afford **15** (17 mg, 35% yield) as a white solid.

^{1}H NMR (500 MHz, CDCl$_3$): δ 7.32 – 7.27 (m, 2H), 7.24 – 7.20 (m, 1H), 7.06 – 7.00 (m, 2H), 5.22 (s, 2H), 3.03 (t, *J* = 5.8 Hz, 1H), 2.57 – 2.32 (m, 4H), 1.59 (s, 3H), 1.18 (s, 3H).
^{13}C NMR (126 MHz, CDCl$_3$): δ 174.8, 142.4, 140.8, 128.4, 127.1, 126.1, 119.4, 82.4, 49.7, 37.2, 36.3, 30.4, 25.9, 18.1.

HRMS (ESI, *m/z*): Calcd for $C_{16}H_{19}O_2^+$ [M+H]$^+$: 243.1376, found: 243.1380.
IR: 2938 (w), 1746 (s), 1494 (m), 1339 (m), 1314 (m), 1243 (m), 1194 (m), 1067 (m), 1035 (m), 995 (m), 758 (m), 701 (m).
m.p.: 90 – 92 °C.

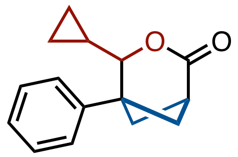

16

4-cyclopropyl-5-phenyl-3-oxabicyclo[3.1.1]heptan-2-one (16): The title compound was prepared according to the general procedure using **1** (31.6 mg, 0.200 mmol, 1.00 equiv.) and cyclopropanecarboxaldehyde (18 µL, 0.24 mmol, 1.2 equiv.). The crude was purified by FCC (5% to 10% EtOAc in hexanes) to afford **16** (14 mg, 31% yield) as a white solid.

^{1}H NMR (500 MHz, CDCl$_3$): δ 7.34 (t, *J* = 7.4 Hz, 2H), 7.27 (d, *J* = 7.5 Hz, 1H), 7.12 – 7.07 (m, 2H), 3.86 (d, *J* = 8.6 Hz, 1H), 3.03 (t, *J* = 5.5 Hz, 1H), 2.56 – 2.49 (m, 2H), 2.46 (dd, *J* = 9.4, 5.9 Hz, 1H), 2.37 (dd, *J* = 9.5, 7.0 Hz, 1H), 0.91 (dtd, *J* = 13.1, 8.2, 4.9 Hz, 1H), 0.61 – 0.51 (m, 1H), 0.47 – 0.39 (m, 1H), 0.13 – 0.05 (m, 1H), -0.37 – -0.48 (m, 1H).
^{13}C NMR (126 MHz, CDCl$_3$): δ 174.9, 143.0, 128.6, 127.2, 125.8, 90.5, 50.1, 37.2, 36.4, 30.4, 12.2, 3.8, 2.8.
HRMS (ESI, *m/z*): Calcd for $C_{15}H_{16}O_2Na^+$ [M+Na]$^+$: 251.1043, found: 251.1033.
IR: 2990 (w), 1746 (s), 1345 (m), 1199 (m), 1072 (m), 1037 (m), 1000 (m), 754 (m), 700 (m).
m.p.: 87 – 89 °C.

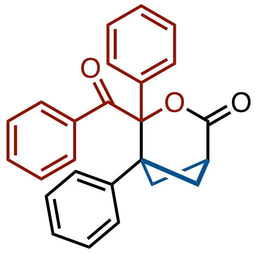

17

4-benzoyl-4,5-diphenyl-3-oxabicyclo[3.1.1]heptan-2-one (17): The title compound was prepared according to the general procedure using **1** (31.6 mg, 0.200 mmol, 1.00 equiv.) and benzil (51 mg, 0.24 mmol, 1.2 equiv.). The crude was purified by FCC (10% to 20% EtOAc in hexanes) to afford **17** (35 mg, 47% yield) as a white solid.

^{1}H NMR (500 MHz, CDCl$_3$): δ 7.79 (d, *J* = 7.1 Hz, 2H), 7.37 (t, *J* = 7.4 Hz, 1H), 7.31 (t, *J* = 7.4 Hz, 1H), 7.23 (t, *J* = 7.9 Hz, 4H), 7.18 – 7.08 (m, 3H), 6.97 (d, *J* = 7.6 Hz, 2H), 6.80 (s, 2H), 3.13

(t, J = 6.0 Hz, 1H), 2.95 (dd, J = 10.3, 6.2 Hz, 1H), 2.77 (dd, J = 10.3, 8.0 Hz, 1H), 2.67 (dd, J = 10.2, 8.0 Hz, 1H), 2.24 (dd, J = 10.2, 6.0 Hz, 1H).
^{13}C NMR (126 MHz, CDCl$_3$): δ 194.3, 173.3, 142.7, 135.8, 134.9, 132.9, 131.0, 129.6, 128.8, 128.1, 128.0, 127.1, 126.8, 126.7, 92.8, 53.1, 38.0, 35.4, 34.7.
HRMS (ESI, *m/z*): Calcd for $C_{25}H_{20}O_3Na^+$ [M+Na]$^+$: 391.1305, found: 391.1299.
IR: 3056 (w), 2923 (w), 1768 (s), 1695 (m), 1512 (m), 1243 (m), 1179 (m), 1032 (m), 1012 (m), 766 (m), 701 (m).
m.p.: 173 – 175 °C.

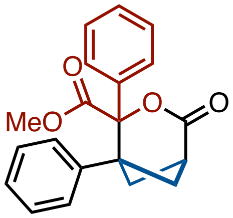

18

methyl 4-oxo-1,2-diphenyl-3-oxabicyclo[3.1.1]heptane-2-carboxylate (18): The title compound was prepared according to the general procedure using **1** (31.6 mg, 0.200 mmol, 1.00 equiv.) and methyl 2-oxo-2-phenylacetate (34 µL, 0.24 mmol, 1.2 equiv.). The crude was purified by FCC (10% to 20% EtOAc in hexanes) to afford **18** (48 mg, 74% yield and 10% inseparable methyl 2-oxo-2-phenylacetate) as a colorless gel.

^{1}H NMR (500 MHz, CDCl$_3$): δ 7.31 (t, J = 7.3 Hz, 2H), 7.21 (dd, J = 16.9, 7.6 Hz, 4H), 7.03 (d, J = 8.3 Hz, 2H), 6.71 (d, J = 7.2 Hz, 2H), 3.74 (s, 3H), 3.14 (t, J = 5.9 Hz, 1H), 2.89 (dd, J = 10.2, 8.0 Hz, 1H), 2.72 (dd, J = 10.2, 6.1 Hz, 1H), 2.44 (dd, J = 10.2, 8.1 Hz, 1H), 2.28 (dd, J = 10.2, 5.9 Hz, 1H).
^{13}C NMR (126 MHz, CDCl$_3$): δ 172.8, 169.3, 141.5, 134.7, 128.9, 128.7, 127.7, 127.3(7), 127.3(5), 127.2, 89.5, 53.0, 52.7, 38.1, 35.1, 33.8.
HRMS (ESI, *m/z*): Calcd for $C_{20}H_{18}O_4Na^+$ [M+Na]$^+$: 345.1097, found: 345.1091.
IR: 2939 (w), 1767 (s), 1741 (s), 1446 (m), 1267 (m), 1245 (m), 1224 (m), 1071 (m), 1036 (m), 775 (m), 701 (m).

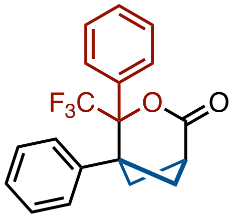

19

4,5-diphenyl-4-(trifluoromethyl)-3-oxabicyclo[3.1.1]heptan-2-one (19): The title compound was prepared according to the general procedure using **1** (31.6 mg, 0.200 mmol, 1.00 equiv.) and

2,2,2-trifluoroacetophenone (34 μL, 0.24 mmol, 1.2 equiv.). The crude was purified by FCC (10% to 20% EtOAc in hexanes) to afford **19** (30 mg, 45% yield) as a white solid.

^{1}H NMR (500 MHz, CDCl$_3$): δ 7.38 (t, *J* = 7.3 Hz, 1H), 7.33 – 7.26 (m, 5H), 7.12 (s, 2H), 6.83 (s, 2H), 3.23 – 3.08 (m, 2H), 2.63 (dd, *J* = 10.2, 6.0 Hz, 1H), 2.35 (dd, *J* = 4.8, 2.2 Hz, 2H).
^{13}C NMR (126 MHz, CDCl$_3$): δ 171.7, 140.8, 132.7, 129.3, 128.8, 127.9, 127.8, 127.8, 127.6, 124.8 (q, *J* = 287.3 Hz, CF$_3$), 87.2 (q, *J* = 27.7 Hz, C), 51.5, 38.3, 35.3, 34.9 (q, *J* = 2.5 Hz, C).
^{19}F NMR (470 MHz, CDCl$_3$): δ -68.8.
HRMS (ESI, *m*/*z*): Calcd for $C_{19}H_{15}O_2F_3Na^+$ [M+Na]$^+$: 355.0911, found: 355.0916.
IR: 3034 (w), 1776 (s), 1447 (m), 1295 (m), 1250 (m), 1169 (s), 1154 (s), 1068 (m), 1033 (m), 1019 (m), 915 (m), 771 (m), 702 (m).
m.p.: 108 – 110 °C.

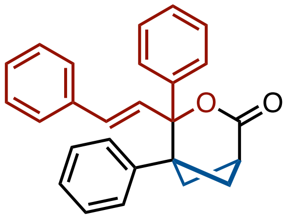

20

(*E*)-4,5-diphenyl-4-styryl-3-oxabicyclo[3.1.1]heptan-2-one (20): The title compound was prepared according to the general procedure using **1** (31.6 mg, 0.200 mmol, 1.00 equiv.) and (*E*)-chalcone (50 mg, 0.24 mmol, 1.2 equiv.). The crude was purified by FCC (10% to 20% EtOAc in hexanes) to afford **20** (43 mg, 55% yield) as a white solid.

^{1}H NMR (500 MHz, CDCl$_3$): δ 7.42 – 7.37 (m, 2H), 7.33 (dd, *J* = 8.3, 6.6 Hz, 2H), 7.31 – 7.26 (m, 4H), 7.26 – 7.18 (m, 3H), 6.99 – 6.89 (m, 3H), 6.74 (d, *J* = 6.1 Hz, 2H), 6.64 (d, *J* = 15.7 Hz, 1H), 3.15 (t, *J* = 5.9 Hz, 1H), 2.71 (dd, *J* = 9.7, 8.0 Hz, 1H), 2.56 – 2.43 (m, 2H), 2.40 – 2.27 (m, 1H).
^{13}C NMR (126 MHz, CDCl$_3$): δ 174.2, 142.3, 138.9, 136.6, 130.9, 128.8, 128.2, 128.1, 127.8, 127.8, 127.7, 127.5, 127.5, 127.3, 126.8, 88.4, 53.4, 38.1, 34.4, 32.5.
HRMS (ESI, *m*/*z*): Calcd for $C_{26}H_{22}O_2Na^+$ [M+Na]$^+$: 339.1512, found: 389.1508.
IR: 3025 (w), 1754 (s), 1494 (m), 1446 (m), 1313 (m), 1256 (m), 1224 (m), 1184 (m), 1039 (m), 1020 (m), 770 (m), 746 (m), 696 (m).
m.p.: 147 – 149 °C.

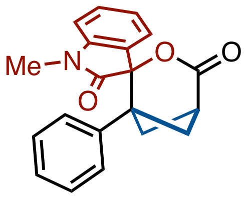

21

1'-methyl-1-phenyl-3-oxaspiro[bicyclo[3.1.1]heptane-2,3'-indoline]-2',4-dione (21): The title compound was prepared according to the general procedure using **1** (31.6 mg, 0.200 mmol, 1.00 equiv.) and 1-methylindoline-2,3-dione (39 mg, 0.24 mmol, 1.2 equiv.). The crude was purified by FCC (10% to 30% EtOAc in hexanes) to afford **21** (33 mg, 51% yield) as a white solid.

^{1}H NMR (500 MHz, CDCl$_3$): δ 7.57 (dd, *J* = 7.5, 1.2 Hz, 1H), 7.26 – 7.21 (m, 1H), 7.10 – 6.99 (m, 4H), 6.72 – 6.65 (m, 2H), 6.43 (d, *J* = 7.8 Hz, 1H), 3.78 (dd, *J* = 9.7, 8.3 Hz, 1H), 3.24 (t, *J* = 6.0 Hz, 1H), 3.01 (dd, *J* = 10.0, 8.3 Hz, 1H), 2.80 (s, 3H), 2.59 (dd, *J* = 10.0, 5.9 Hz, 1H), 2.46 – 2.39 (m, 1H).
^{13}C NMR (126 MHz, CDCl$_3$): δ 173.0, 172.9, 143.3, 139.3, 130.8, 127.6, 127.4, 126.4, 126.2, 124.6, 122.8, 108.3, 85.1, 52.8, 38.4, 33.5, 31.9, 25.9.
HRMS (ESI, *m/z*): Calcd for $C_{20}H_{18}O_3N^+$ [M+H]$^+$: 320.1281, found: 320.1276.
IR: 2955 (w), 1765 (s), 1724 (s), 1614 (m), 1493 (m), 1470 (m), 1390 (m), 1311 (m), 1220 (m), 1098 (m), 1033 (s), 752 (m), 706 (m).
m.p.: 246 – 248 °C.

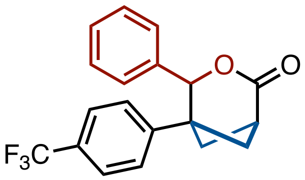

22

4-phenyl-5-(4-(trifluoromethyl)phenyl)-3-oxabicyclo[3.1.1]heptan-2-one (22): The title compound was prepared according to the general procedure using **SI-1** (45.2 mg, 0.200 mmol, 1.00 equiv.) and benzaldehyde (25 µL, 0.24 mmol, 1.2 equiv.). The crude was purified by FCC (5% to 20% EtOAc in hexanes) to afford **22** (60 mg, 90% yield) as a white solid.

^{1}H NMR (500 MHz, CDCl$_3$): δ 7.48 (d, *J* = 8.0 Hz, 2H), 7.26 – 7.21 (m, 1H), 7.20 – 7.13 (m, 2H), 6.84 (d, *J* = 8.0 Hz, 2H), 6.75 (d, *J* = 7.4 Hz, 2H), 5.64 (s, 1H), 3.18 (t, *J* = 5.7 Hz, 1H), 2.69 – 2.54 (m, 3H), 2.35 (dd, *J* = 9.2, 6.1 Hz, 1H).
^{13}C NMR (126 MHz, CDCl$_3$): δ 173.8, 146.3, 135.8, 129.7 (q, *J* = 32.8 Hz, C), 128.5, 127.9, 126.9, 126.7, 125.4 (q, *J* = 3.8 Hz, CH), 124.1 (q, *J* = 272.2 Hz, CF$_3$), 86.7, 50.7, 37.5, 36.5, 29.2.
^{19}F NMR (470 MHz, CDCl$_3$): δ -62.5.
HRMS (ESI, *m/z*): Calcd for $C_{19}H_{15}O_2F_3Na^+$ [M+Na]$^+$: 355.0916, found: 355.0913.
IR: 2950 (w), 1756 (s), 1618 (m), 1324 (s), 1166 (m), 1111 (m), 1060 (s), 1030 (m), 1015 (m), 839

(m), 756 (m), 701 (m),
m.p.: 133 – 135 °C.

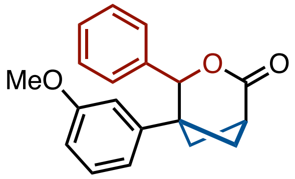

23

5-(3-methoxyphenyl)-4-phenyl-3-oxabicyclo[3.1.1]heptan-2-one (23): The title compound was prepared according to the general procedure using **SI-2** (37.6 mg, 0.200 mmol, 1.00 equiv.) and benzaldehyde (25 μL, 0.24 mmol, 1.2 equiv.). The crude was purified by FCC (5% to 20% EtOAc in hexanes) to afford **23** (33 mg, 55% yield) as a white solid.

^{1}H NMR (500 MHz, $CDCl_3$): δ 7.21 (t, J = 7.3 Hz, 1H), 7.15 (td, J = 7.8, 3.9 Hz, 3H), 6.81 – 6.73 (m, 3H), 6.35 (d, J = 7.5 Hz, 1H), 6.19 (s, 1H), 5.63 (s, 1H), 3.66 (s, 3H), 3.17 – 3.10 (m, 1H), 2.63 – 2.56 (m, 2H), 2.52 – 2.45 (m, 1H), 2.33 (dd, J = 9.9, 6.1 Hz, 1H).
^{13}C NMR (126 MHz, $CDCl_3$): δ 174.5, 159.6, 143.8, 136.4, 129.5, 128.2, 127.7, 127.0, 118.5, 112.8, 112.1, 87.0, 55.3, 50.7, 37.4, 36.6, 29.1.
HRMS (ESI, *m/z*): Calcd for $C_{19}H_{19}O_3^+$ $[M+H]^+$: 295.1329, found: 295.1324.
IR: 2938 (w), 1752 (s), 1602 (m), 1488 (m), 1319 (m), 1252 (m), 1214 (m), 1028 (m), 781 (m), 701 (m).
m.p.: 103 – 105 °C.

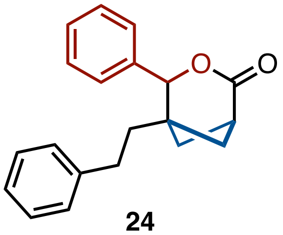

24

5-phenethyl-4-phenyl-3-oxabicyclo[3.1.1]heptan-2-one (24): The title compound was prepared according to the general procedure using **SI-3** (37.3 mg, 0.200 mmol, 1.00 equiv.) and benzaldehyde (25 μL, 0.24 mmol, 1.2 equiv.). The crude was purified by FCC (5% to 10% EtOAc in hexanes) to afford **24** (12 mg, 21% yield) as a colorless oil.

^{1}H NMR (500 MHz, $CDCl_3$): δ 7.42 – 7.36 (m, 3H), 7.35 – 7.32 (m, 2H), 7.28 – 7.25 (m, 2H), 7.18 (t, J = 7.4 Hz, 1H), 7.09 (d, J = 6.9 Hz, 2H), 5.53 (s, 1H), 3.05 (t, J = 6.0 Hz, 1H), 2.72 – 2.65 (m, 1H), 2.49 – 2.42 (m, 1H), 2.38 (dd, J = 9.5, 5.9 Hz, 1H), 2.24 – 2.19 (m, 1H), 2.08 (dd, J = 9.6, 8.1 Hz, 1H), 2.03 (dt, J = 10.4, 4.8 Hz, 1H), 1.71 (ddd, J = 14.2, 11.8, 5.1 Hz, 1H), 1.55 – 1.50 (m, 1H).

^{13}C NMR (126 MHz, CDCl$_3$): δ 175.0, 141.3, 137.2, 128.8, 128.7, 128.5, 128.3, 127.3, 126.3, 86.0, 46.9, 37.6, 36.8, 34.9, 30.4, 29.3.
HRMS (ESI, *m/z*): Calcd for $C_{20}H_{20}O_2Na^+$ [M+Na]$^+$: 315.1356, found: 315.1349.
IR: 2950 (w), 1750 (s), 1558 (m), 1455 (m), 1236 (m), 1043 (m), 752 (m), 702 (m).

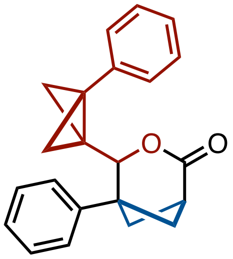

25

5-phenyl-4-(3-phenylbicyclo[1.1.0]butan-1-yl)-3-oxabicyclo[3.1.1]heptan-2-one (25): The title compound was prepared according to the general procedure using **1** (31.6 mg, 0.200 mmol, 1.00 equiv.) and **1** (38 mg, 0.24 mmol, 1.2 equiv.). The crude was purified by FCC (5% to 15% EtOAc in hexanes) to afford **25** (24 mg, 38% yield) as a colorless oil.

^{1}H NMR (500 MHz, CDCl$_3$): δ 7.34 (t, J = 7.4 Hz, 2H), 7.29 – 7.25 (m, 3H), 7.21 – 7.18 (m, 2H), 7.15 (t, J = 7.3 Hz, 1H), 7.11 – 7.08 (m, 2H), 4.54 (s, 1H), 3.02 (t, J = 5.9 Hz, 1H), 2.65 (dd, J = 9.5, 7.8 Hz, 1H), 2.54 (dd, J = 14.8, 6.3 Hz, 2H), 2.48 (dd, J = 9.4, 5.9 Hz, 1H), 2.36 (dd, J = 9.5, 7.8 Hz, 1H), 1.35 (d, J = 6.5 Hz, 1H), 1.15 (s, 1H), 0.70 (s, 1H).
^{13}C NMR (126 MHz, CDCl$_3$): δ 174.1, 142.5, 135.8, 128.5, 128.4, 127.3, 126.4, 126.1, 125.6, 83.2, 50.5, 37.1, 35.7, 33.7, 30.6, 30.3, 22.5, 15.8.
HRMS (ESI, *m/z*): Calcd for $C_{22}H_{20}O_2Na^+$ [M+Na]$^+$: 339.1356, found: 339.1349.
IR: 2938 (w), 1753 (s), 1601 (m), 1489 (m), 1338 (m), 1318 (m), 1194 (m), 1072 (m), 1036 (m), 1013 (m), 757 (m), 697 (m).

5.2 General procedure for cycloaddition with isatin *N*-Boc imine:

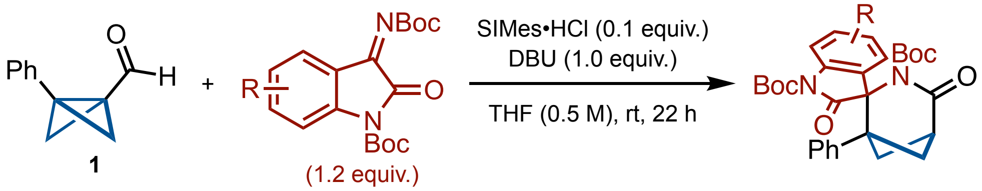

An oven-dried 1-dram vial equipped with a stir bar was cooled under vacuum. **1** (31.6 mg, 0.200 mmol, 1.00 equiv.), isatin *N*-Boc imine (0.24 mmol, 1.2 equiv.), and SIMes•HCl (6.9 mg, 0.020 mmol, 0.10 equiv.) were added. The vial was then sealed with a septum placed under vacuum. After backfilling three times with N_2, THF (0.4 mL, 0.5 M) was added to the reaction vial via a syringe. Then DBU (30 μL, 0.20 mmol, 1.0 equiv.) was added via the syringe. The septum on the reaction vial was quickly replaced by a screw cap and the reaction was stirred at room temperature for 22 hours. Upon completion, the solvent was removed by rotary evaporation. CH_2Br_2 (14 μL,

0.2 mmol) was added as an internal standard for crude ^{1}H NMR analysis to determine the yield. The crude was purified by FCC to give the corresponding cycloadducts.

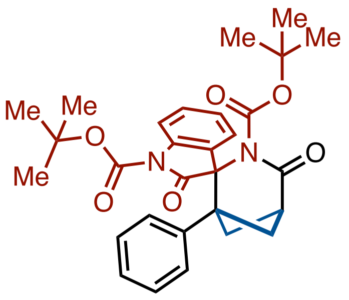

26

di-*tert*-butyl 2',4-dioxo-1-phenyl-3-azaspiro[bicyclo[3.1.1]heptane-2,3'-indoline]-1',3-dicarboxylate (26): The title compound was prepared according to the general procedure using **1** (31.6 mg, 0.200 mmol, 1.00 equiv.) and **SI-11** (83 mg, 0.24 mmol, 1.2 equiv.). The crude was purified by FCC (10% to 30% EtOAc in hexanes) to afford **26** (69 mg, 68% yield) as a white solid.

^{1}H NMR (500 MHz, CDCl$_3$): δ 7.48 (d, *J* = 7.6 Hz, 1H), 7.42 (d, *J* = 8.4 Hz, 1H), 7.24 (d, *J* = 8.1 Hz, 1H), 7.17 (t, *J* = 7.6 Hz, 1H), 7.08 (t, *J* = 7.4 Hz, 1H), 7.00 (t, *J* = 7.7 Hz, 2H), 6.56 (s, 2H), 3.49 – 3.40 (m, 1H), 3.15 (t, *J* = 5.9 Hz, 1H), 2.86 (dd, *J* = 10.2, 8.1 Hz, 1H), 2.36 (dt, *J* = 9.9, 6.5 Hz, 2H), 1.51 (s, 9H), 1.10 (s, 9H).
^{13}C NMR (126 MHz, CDCl$_3$): δ 174.1, 172.7, 149.4, 148.3, 139.6, 139.5, 129.8, 127.9, 127.5, 127.5, 126.6, 124.2, 122.4, 114.9, 84.7, 84.0, 69.7, 53.3, 40.3, 33.1, 31.7, 28.2, 27.3.
HRMS (ESI, *m/z*): Calcd for $C_{29}H_{32}O_6N_2Na^+$ [M+Na]$^+$: 527.2153, found: 527.2145.
IR: 2985 (w), 1779 (s), 1733 (s), 1489 (m), 1369 (m), 1348 (m), 1293 (m), 1242 (m), 1149 (s), 935 (m), 842 (m), 772 (m), 701 (m).
m.p.: 195 – 197 °C.

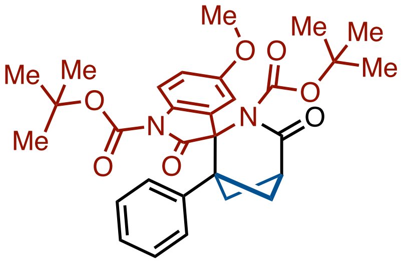

27

di-*tert*-butyl 5'-methoxy-2',4-dioxo-1-phenyl-3-azaspiro[bicyclo[3.1.1]heptane-2,3'-indoline]-1',3-dicarboxylate (27): The title compound was prepared according to the general procedure using **1** (31.6 mg, 0.200 mmol, 1.00 equiv.) and **SI-12** (90 mg, 0.24 mmol, 1.2 equiv.). The crude was purified by FCC (10% to 30% EtOAc in hexanes) to afford **27** (70 mg, 65% yield) as a white solid.

^{1}H NMR (500 MHz, CDCl$_3$): δ 7.33 (d, *J* = 8.9 Hz, 1H), 7.09 (t, *J* = 7.4 Hz, 1H), 7.06 – 6.97 (m, 3H), 6.77 (dd, *J* = 8.9, 2.7 Hz, 1H), 6.60 (s, 2H), 3.82 (s, 3H), 3.45 (dd, *J* = 9.9, 8.2 Hz, 1H), 3.14 (t, *J* = 5.9 Hz, 1H), 2.84 (dd, *J* = 10.2, 8.2 Hz, 1H), 2.43 – 2.30 (m, 2H), 1.50 (s, 9H), 1.13 (s, 9H).
^{13}C NMR (126 MHz, CDCl$_3$): δ 174.1, 172.7, 156.8, 149.5, 148.3, 139.5, 133.0, 129.1, 127.5(2), 127.5(0), 126.6, 115.8, 114.3, 109.0, 84.8, 83.8, 69.9, 56.1, 53.3, 40.3, 33.1, 31.8, 28.2, 27.4.
HRMS (ESI, *m*/*z*): Calcd for $C_{30}H_{34}O_7N_2Na^+$ $[M+Na]^+$: 557.2258, found: 557.2246.
IR: 2983 (w), 1777 (s), 1729 (s), 1489 (m), 1369 (m), 1302 (m), 1272 (m), 1245 (m), 1148 (s), 1020 (m), 912 (m), 729 (m), 700 (m).
m.p.: 180 – 182 °C.

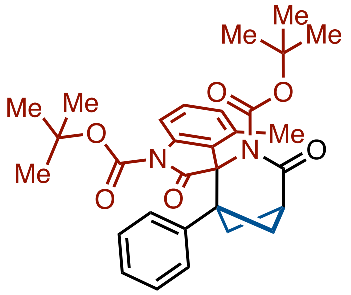

28

di-*tert*-butyl 4'-methyl-2',4-dioxo-1-phenyl-3-azaspiro[bicyclo[3.1.1]heptane-2,3'-indoline]-1',3-dicarboxylate (28): The title compound was prepared according to the general procedure using **1** (31.6 mg, 0.200 mmol, 1.00 equiv.) and **SI-13** (87 mg, 0.24 mmol, 1.2 equiv.). The crude was purified by FCC (10% to 30% EtOAc in hexanes) to afford **28** (15 mg, 14% yield) as a white solid.

^{1}H NMR (500 MHz, CDCl$_3$): δ 7.22 (d, *J* = 8.1 Hz, 1H), 7.09 (q, *J* = 8.9 Hz, 2H), 7.05 – 6.97 (m, 2H), 6.95 (d, *J* = 7.8 Hz, 1H), 6.59 (s, 2H), 3.47 (t, *J* = 9.2 Hz, 1H), 3.13 (t, *J* = 5.8 Hz, 1H), 2.95 (t, *J* = 9.3 Hz, 1H), 2.55 (s, 3H), 2.34 (dt, *J* = 9.8, 6.9 Hz, 2H), 1.51 (s, 9H), 1.12 (s, 9H).
^{13}C NMR (126 MHz, CDCl$_3$): δ 173.8, 172.5, 149.5, 148.2, 140.0, 139.8, 133.2, 128.8, 127.8, 127.5, 127.4, 126.5, 125.3, 112.2, 84.5, 84.0, 71.8, 54.8, 40.7, 34.3, 32.3, 28.2, 27.3, 20.1.
HRMS (ESI, *m*/*z*): Calcd for $C_{30}H_{34}O_6N_2Na^+$ $[M+Na]^+$: 541.2309, found: 541.2301.
IR: 2977 (w), 1773 (s), 1730 (s), 1458 (m), 1369 (m), 1287 (m), 1248 (m), 1149 (s), 912 (m), 731 (m), 701 (m).
m.p.: 185 – 187 °C.

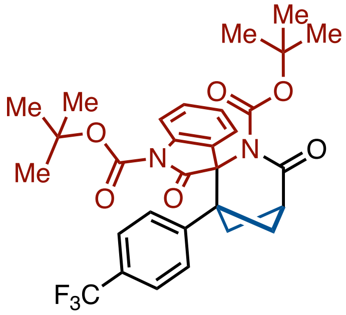

29

di-*tert*-butyl 2',4-dioxo-1-(4-(trifluoromethyl)phenyl)-3-azaspiro[bicyclo[3.1.1]heptane-2,3'-indoline]-1',3-dicarboxylate (29): The title compound was prepared according to the general procedure using **SI-1** (45.2 mg, 0.200 mmol, 1.00 equiv.) and **SI-11** (83 mg, 0.24 mmol, 1.2 equiv.). The crude was purified by FCC (10% to 30% EtOAc in hexanes) to afford **29** (85 mg, 74% yield) as a white solid.

^{1}H NMR (500 MHz, CDCl$_3$): δ 7.50 (dd, J = 7.5, 1.4 Hz, 1H), 7.38 (d, J = 8.2 Hz, 1H), 7.28 (ddd, J = 8.1, 6.5, 1.4 Hz, 3H), 7.20 (td, J = 7.5, 1.1 Hz, 1H), 6.68 (s, 2H), 3.48 (dd, J = 9.9, 8.2 Hz, 1H), 3.18 (t, J = 6.0 Hz, 1H), 2.89 (dd, J = 10.2, 8.2 Hz, 1H), 2.37 (ddd, J = 10.0, 6.0, 2.9 Hz, 2H), 1.49 (s, 9H), 1.11 (s, 9H).
^{13}C NMR (126 MHz, CDCl$_3$): δ 173.5, 172.6, 149.3, 147.9, 143.6, 139.4, 130.2, 129.7 (q, J = 32.8 Hz, C), 127.4, 127.2, 124.4 (q, J = 5.0 Hz, CH), 124.0 (q, J = 272.2 Hz, CF$_3$), 122.5, 115.0, 85.0, 84.5, 69.4, 53.0, 40.2, 33.1, 31.7, 28.0, 27.3.
^{19}F NMR (470 MHz, CDCl$_3$): δ -62.7.
HRMS (ESI, *m*/*z*): Calcd for $C_{30}H_{31}O_6N_2F_3Na^+$ [M+Na]$^+$: 595.2019, found: 595.2026.
IR: 2989 (w), 1781 (s), 1734 (s), 1479 (m), 1370 (m), 1324 (s), 1241 (m), 1148 (s), 1071 (m), 1018 (m), 839 (m), 758 (m), 687 (m).
m.p.: 172 – 174 °C.

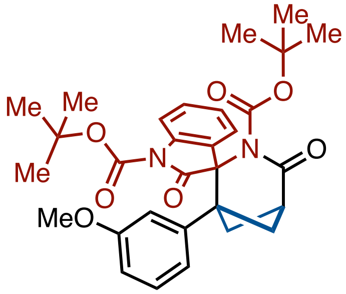

30

di-*tert*-butyl 1-(3-methoxyphenyl)-2',4-dioxo-3-azaspiro[bicyclo[3.1.1]heptane-2,3'-indoline]-1',3-dicarboxylate (30): The title compound was prepared according to the general procedure using **SI-2** (37.6 mg, 0.200 mmol, 1.00 equiv.) and **SI-11** (83 mg, 0.24 mmol, 1.2 equiv.).

The crude was purified by FCC (10% to 30% EtOAc in hexanes) to afford **30** (68 mg, 64% yield) as a white solid.

^{1}H NMR (500 MHz, CDCl$_3$): δ 7.47 (ddd, *J* = 7.7, 5.7, 1.2 Hz, 2H), 7.29 – 7.25 (m, 1H), 7.18 (td, *J* = 7.6, 1.1 Hz, 1H), 6.92 (t, *J* = 8.0 Hz, 1H), 6.63 (ddd, *J* = 8.2, 2.6, 0.9 Hz, 1H), 6.17 (s, 1H), 6.03 (s, 1H), 3.54 (s, 3H), 3.43 (dd, *J* = 9.9, 8.2 Hz, 1H), 3.14 (t, *J* = 5.9 Hz, 1H), 2.84 (dd, *J* = 10.1, 8.2 Hz, 1H), 2.36 (ddd, *J* = 10.0, 6.0, 1.9 Hz, 2H), 1.51 (s, 9H), 1.10 (s, 9H).
^{13}C NMR (126 MHz, CDCl$_3$): δ 174.0, 172.7, 158.8, 149.4, 148.3, 141.0, 139.8, 129.8, 128.5, 128.1, 124.2, 122.4, 119.1, 114.9, 113.8, 111.7, 84.7, 84.1, 69.6, 55.1, 53.2, 40.2, 33.1, 31.8, 28.1, 27.3.
HRMS (ESI, *m/z*): Calcd for $C_{30}H_{34}O_7N_2Na^+$ $[M+Na]^+$: 557.2258, found: 557.2251.
IR: 2984 (w), 1781 (s), 1733 (s), 1603 (m), 1479 (m), 1369 (m), 1348 (m), 1239 (m), 1149 (s), 837 (m), 759 (m), 700 (m).
m.p.: 163 – 165 °C.

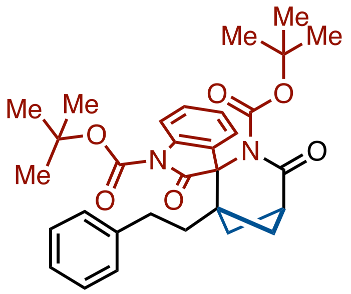

31

di-*tert*-butyl 2',4-dioxo-1-phenethyl-3-azaspiro[bicyclo[3.1.1]heptane-2,3'-indoline]-1',3-dicarboxylate (31): The title compound was prepared according to the general procedure using **SI-3** (37.3 mg, 0.200 mmol, 1.00 equiv.) and **SI-11** (83 mg, 0.24 mmol, 1.2 equiv.). The crude was purified by FCC (10% to 40% EtOAc in hexanes) to afford **31** (24 mg, 23% yield) as a yellow oil.

^{1}H NMR (500 MHz, CDCl$_3$): δ 7.92 (d, *J* = 8.2 Hz, 1H), 7.40 (td, *J* = 7.9, 1.4 Hz, 1H), 7.36 (dd, *J* = 7.6, 1.4 Hz, 1H), 7.21 – 7.16 (m, 3H), 7.13 (t, *J* = 7.3 Hz, 1H), 6.92 (d, *J* = 6.9 Hz, 2H), 3.05 (t, *J* = 6.0 Hz, 1H), 2.97 (dd, *J* = 10.2, 8.3 Hz, 1H), 2.46 (td, *J* = 12.9, 5.1 Hz, 1H), 2.37 (dd, *J* = 10.5, 8.3 Hz, 1H), 2.24 – 2.21 (m, 1H), 2.18 (dd, *J* = 10.4, 6.1 Hz, 1H), 1.60 (s, 9H), 1.54 (d, *J* = 4.7 Hz, 1H), 1.48 (d, *J* = 7.4 Hz, 1H), 1.32 – 1.27 (m, 1H), 1.12 (s, 9H).
^{13}C NMR (126 MHz, CDCl$_3$): δ 174.4, 173.6, 149.5, 149.0, 141.1, 140.4, 130.1, 128.6, 128.3, 128.2, 126.2, 124.9, 123.0, 115.3, 84.9, 84.7, 69.1, 50.5, 39.6, 33.9, 30.9, 30.6, 29.8, 28.2, 27.3.
HRMS (ESI, *m/z*): Calcd for $C_{31}H_{36}O_6N_2Na^+$ $[M+Na]^+$: 555.2466, found: 555.2451.
IR: 2984 (w), 1777 (s), 1733 (s), 1478 (m), 1369 (m), 1344 (m), 1288 (m), 1243 (m), 1148 (s), 910 (m), 840 (m), 727 (m), 700 (m).

6. Unsuccessful examples:

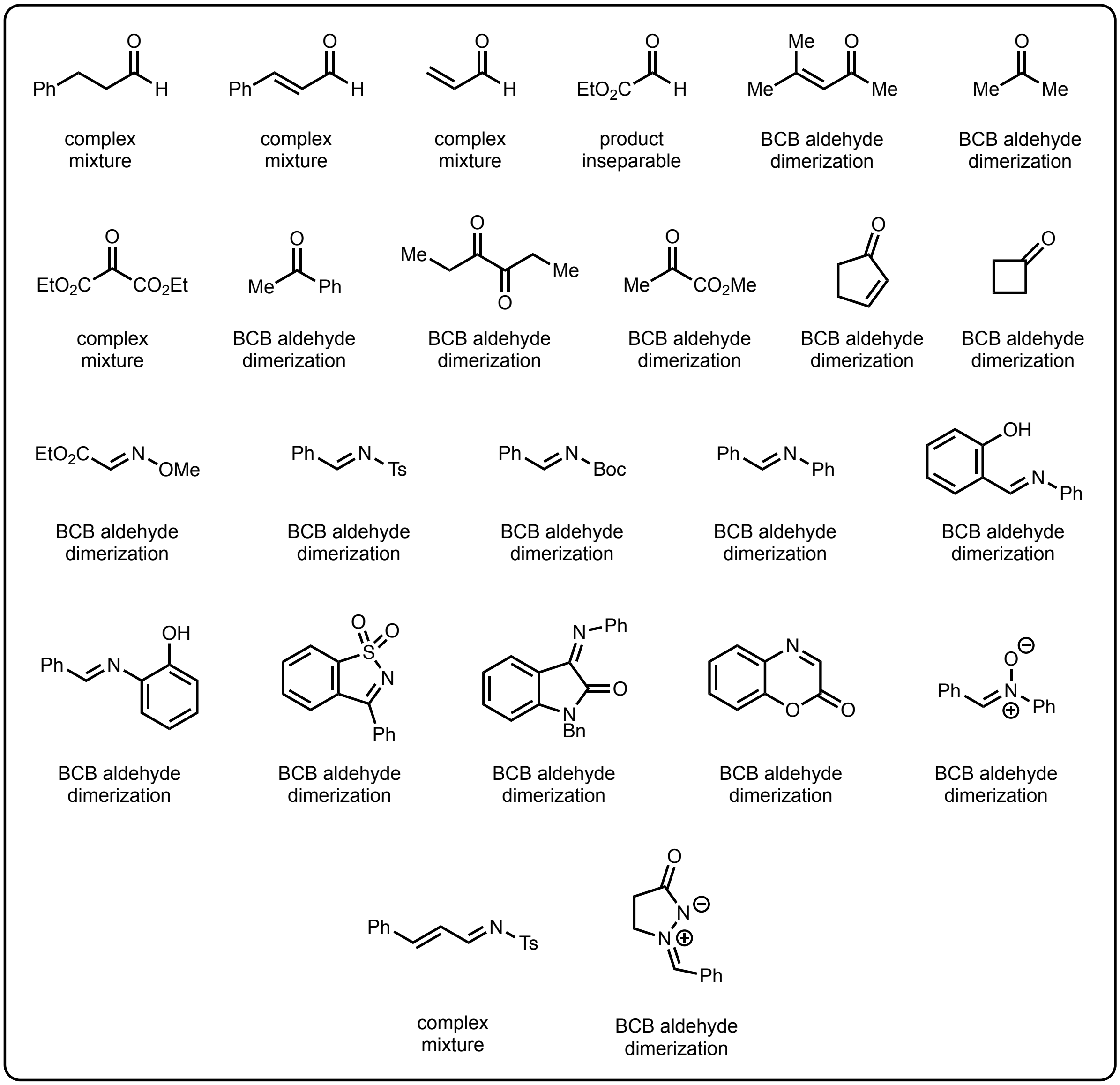

7. Gram Scale Synthesis and Synthetic Transformations:

7.1 Gram scale synthesis of strain-release cycloaddition with benzaldehyde:

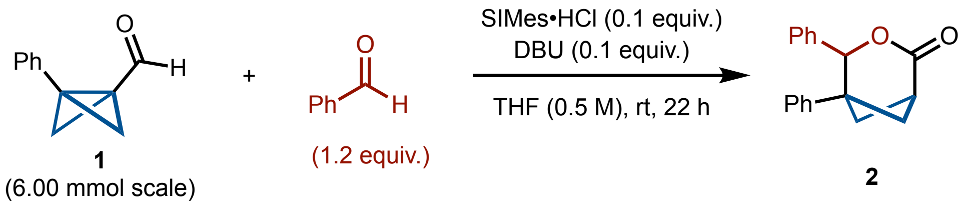

An oven-dried 100 mL round-bottom flask equipped with a stir bar was cooled under vacuum. **1** (949 mg, 6.00 mmol, 1.00 equiv.) and SIMes•HCl (206 mg, 0.600 mmol, 0.100 equiv.) were added. The vial was then sealed with a septum placed under vacuum. After backfilling three times with N_2, THF (12 mL, 0.50 M) was added to the reaction vial via a syringe, followed by the addition of benzaldehyde (0.74 mL, 7.2 mmol, 1.2 equiv.) via a syringe. Then DBU (90 µL, 0.60 mmol, 0.10 equiv.) was added via a syringe. The reaction was stirred at room temperature for 22 hours. Upon completion, the solvent was removed by rotary evaporation. Upon completion, the solvent was removed by rotary evaporation. The residue was purified by FCC (5% to 20% EtOAc in hexanes) to give **2** (1.2 g, 76% yield) as a white solid.

7.2 Synthetic Transformations:

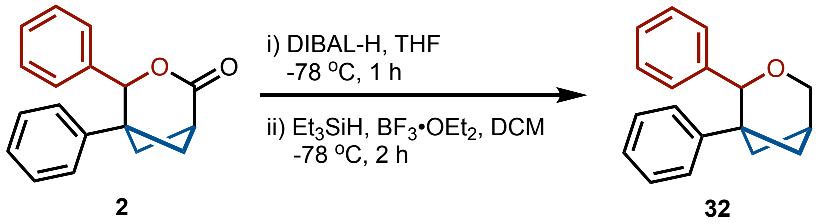

1,2-diphenyl-3-oxabicyclo[3.1.1]heptane (32):

According to the literature[7], an oven-dried 10 mL reaction tube equipped with a stir bar and septum was cooled under vacuum. **2** (26.4 mg, 0.100 mmol, 1.00 equiv.) was added and resealed with a septum. The flask was placed under a vacuum and backfilled three times with N_2. THF (0.10 M, 1.0 mL) was added via syringes. The solution was cooled to -78 °C in an acetone/dry ice bath. DIBAL-H (1.0 M in toluene, 0.30 mL, 0.30 mmol, 3.0 equiv.) was added dropwise down the wall via a syringe, and the reaction was stirred at the same temperature for 1 hour. Upon completion, the reaction is quenched with saturated Rochelle salt solution (2 mL). The mixture was extracted with EtOAc three times (1 mL × 3). The combined organic layers were washed with brine (5 mL), dried over anhydrous Na_2SO_4, gravity-filtered, and concentrated via rotary evaporation. The crude residue was used directly in the next step without further purification.

A 2-dram vial equipped with a stir bar and crude (assuming 0.100 mmol, 1.00 equiv.) was sealed with a septum. The flask was placed under a vacuum and backfilled three times with N_2. DCM (0.10 M, 1.0 mL) was added via syringes. The solution was cooled to -78 °C in an acetone/dry ice bath. Triethylsilane (48 µL, 0.30 mmol, 3.0 equiv.) was added followed by dropwise addition of BF_3•OEt_2 (37 µL, 0.30 mmol, 3.0 equiv.). The reaction was stirred at the same

temperature for 2 hour. Upon completion, the reaction is quenched by slowly adding saturated $NaHCO_3$ solution (1 mL). The mixture was extracted with DCM three times (1 mL × 3). The combined organic layers were washed with brine (5 mL), dried over anhydrous Na_2SO_4, gravity-filtered, and concentrated via rotary evaporation. The residue was purified by FCC (hexanes) to give **32** (16 mg, 63% yield over 2 steps) as a colorless oil.

^{1}H NMR (500 MHz, $CDCl_3$): δ 7.15 – 7.03 (m, 6H), 6.86 (dd, *J* = 8.1, 2.0 Hz, 2H), 6.70 (dd, *J* = 8.0, 1.6 Hz, 2H), 5.12 (s, 1H), 4.38 (ddd, *J* = 9.4, 2.7, 1.5 Hz, 1H), 4.23 (dt, *J* = 9.3, 1.4 Hz, 1H), 2.53 (ddt, *J* = 6.4, 4.4, 2.1 Hz, 1H), 2.39 (ddd, *J* = 8.2, 6.4, 1.5 Hz, 1H), 2.37 – 2.32 (m, 1H), 2.31 – 2.25 (m, 1H), 2.05 (dd, *J* = 9.3, 6.3 Hz, 1H).
^{13}C NMR (126 MHz, $CDCl_3$): δ 145.6, 141.0, 127.8, 127.3, 127.1, 127.0, 126.4, 126.0, 86.6, 71.7, 51.3, 40.0, 31.9, 29.6.
HRMS (ESI, *m/z*): Calcd for $C_{18}H_{18}ONa^+$ $[M+Na]^+$: 273.1250, found: 273.1244.
IR: 2939 (m), 2859 (m), 1496 (m), 1447 (m), 1130 (m), 1040 (m), 1028 (m), 762 (m), 698 (s).

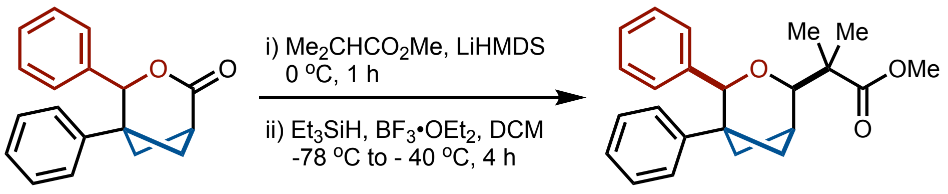

methyl 2-(4,5-diphenyl-3-oxabicyclo[3.1.1]heptan-2-yl)-2-methylpropanoate (33):

According to the literature[8], an oven-dried 10 mL reaction tube equipped with a stir bar and septum was cooled under vacuum. After evacuating backfilling three times with N_2. THF (0.5 M, 0.1 mL) and lithium bis(trimethylsilyl)amide (1.0 M in THF, 0.60 mL, 0.60 mmol, 3.0 equiv.) were added via syringes. The solution was cooled to -78 °C in an acetone/dry ice bath, and methyl isobutyrate (69 µL, 0.600 mmol, 3.00 equiv.) was added slowly via a syringe. After stirring at the same temperature for 30 minutes, the reaction was warmed to 0 °C in a water/ice bath. **2** (52.9 mg, 0.200 mmol, 1.00 equiv.) dissolved in THF (0.2 mL) was added slowly to the reaction via a syringe and stirred at same temperature for 1 hour. Upon completion, the reaction is quenched with saturated NH_4Cl solution (1 mL). The mixture was extracted with EtOAc three times (1 mL × 3). The combined organic layers were washed with brine (5 mL), dried over anhydrous Na_2SO_4, gravity-filtered, and concentrated via rotary evaporation. The crude residue was used directly in the next step without further purification.

A 2-dram vial equipped with a stir bar and crude (assuming 0.200 mmol, 1.00 equiv.) was sealed with a septum. The flask was placed under a vacuum and backfilled three times with N_2. DCM (0.3 M, 0.7 mL) was added via syringes. The solution was cooled to -78 °C in an acetone/dry ice bath. Triethylsilane (96 µL, 0.60 mmol, 3.0 equiv.) was added followed by dropwise addition of BF_3•OEt_2 (74 µL, 0.60 mmol, 3.0 equiv.) via syringes. The reaction was stirred for 4 hours while the temperature naturally warmed to -40 °C. Upon completion, the reaction is quenched by slowly adding saturated $NaHCO_3$ solution (1 mL). The mixture was extracted with DCM three times (1

mL × 3). The combined organic layers were washed with brine (5 mL), dried over anhydrous Na_2SO_4, gravity-filtered, and concentrated via rotary evaporation. The residue was purified by FCC (2% EtOAc in hexanes) to give **33** (53 mg, 75% yield over 2 steps, 16:1 dr) as a colorless oil.

^{1}H NMR (500 MHz, $CDCl_3$): δ 7.07 – 6.99 (m, 6H), 6.96 – 6.91 (m, 2H), 6.61 – 6.54 (m, 2H), 4.97 (s, 1H), 4.18 (s, 1H), 3.71 (s, 3H), 2.54 (dd, J = 8.9, 6.9 Hz, 1H), 2.50 – 2.43 (m, 2H), 2.14 (t, J = 8.4 Hz, 1H), 1.92 (dd, J = 9.2, 5.8 Hz, 1H), 1.37 (s, 3H), 1.35 (s, 3H).
^{13}C NMR (126 MHz, $CDCl_3$): δ 177.1, 145.4, 140.5, 127.6, 127.3, 127.1, 127.0, 126.1, 125.8, 85.7, 83.7, 52.0, 51.3, 47.6, 43.2, 32.3, 27.7, 21.8, 21.4.
HRMS (ESI, *m/z*): Calcd for $C_{23}H_{26}O_3Na^+$ $[M+Na]^+$: 373.1774, found: 373.1766.
IR: 2951 (m), 2868 (w), 1732 (s), 1490 (m), 1470 (m), 1390 (m), 1267 (m), 1123 (m), 1046 (m), 1028 (m), 764 (m), 697 (s).

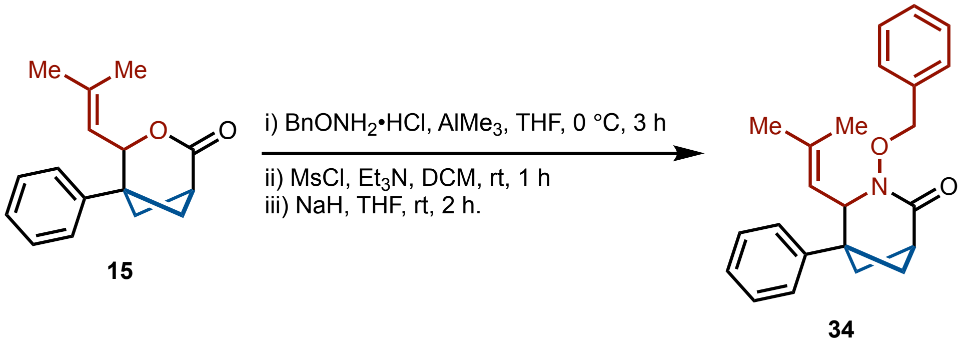

3-(benzyloxy)-4-(2-methylprop-1-en-1-yl)-5-phenyl-3-azabicyclo[3.1.1]heptan-2-one (34):

According to the literature[9], an oven-dried 10 mL reaction tube equipped with a stir bar and septum was cooled under vacuum. *O*-benzylhydroxylamine hydrochloride (48 mg, 0.30 mmol, 3.0 equiv.) was added and resealed with a septum. The flask was placed under a vacuum and backfilled three times with N_2. THF (0.40 M, 0.25 mL) was added via syringes. The solution was cooled to 0 °C in a water/ice bath, and trimethylaluminum (2.0 M in toluene, 0.15 mL, 0.30 mmol, 3.0 equiv.) was added dropwise via a syringe. After stirring the mixture at 0 °C for 30 minutes and at room temperature for 30 minutes, the tube was placed back to 0 °C in a water/ice bath, and **15** (24.2 mg, 0.100 mmol, 1.00 equiv.) dissolved in THF (0.1 mL) was added dropwise to the reaction via a syringe and stirred at the same temperature for 3 hours. Upon completion, the reaction is quenched with saturated $NaHCO_3$ solution (1 mL). The mixture was extracted with EtOAc three times (1 mL × 3). The combined organic layers were washed with brine (5 mL), dried over anhydrous Na_2SO_4, gravity-filtered, and concentrated via rotary evaporation. The crude residue was used directly in the next step without further purification.

A 2-dram vial equipped with a stir bar and crude (assuming 0.200 mmol, 1.00 equiv.) was sealed with a septum. The flask was placed under a vacuum and backfilled three times with N_2. DCM (0.40 M, 0.25 mL) was added via syringes. The solution was cooled to 0 °C in a water/ice bath. Methanesulfonyl chloride (12 µL, 0.15 mmol, 1.5 equiv.) and triethylamine (28 µL, 0.20 mmol, 2.0 equiv.) were added slowly via syringes. The reaction was stirred at the same temperature

for 1 hour. Upon completion, the reaction is quenched with saturated 1M HCl solution (1 mL). The mixture was extracted with DCM three times (1 mL × 3). The combined organic layers were washed with brine (5 mL), dried over anhydrous Na_2SO_4, gravity-filtered, and concentrated via rotary evaporation. The crude residue was used directly in the next step without further purification.

A 2-dram vial equipped with a stir bar and crude (assuming 0.200 mmol, 1.00 equiv.) was sealed with a septum. The flask was placed under a vacuum and backfilled three times with N_2. THF (0.40 M, 0.25 mL) was added via syringes. The solution was cooled to 0 °C in a water/ice bath. Sodium hydride (60 % dispersion in mineral oil, 8 mg, 0.2 mmol, 2 equiv.) was added slowly. The reaction was warmed to room temperature and stirred for 2 hours. Upon completion, the reaction is quenched with water (1 mL). The mixture was extracted with EtOAc three times (1 mL × 3). The combined organic layers were washed with brine (5 mL), dried over anhydrous Na_2SO_4, gravity-filtered, and concentrated via rotary evaporation. The residue was purified by FCC (10% to 20% EtOAc in hexanes) to give **34** (15 mg, 44% yield over 3 steps) as a white solid.

^{1}H NMR (500 MHz, $CDCl_3$): δ 7.42 (d, J = 6.9 Hz, 2H), 7.35 (t, J = 7.5 Hz, 2H), 7.30 – 7.26 (m, 2H), 7.24 (s, 1H), 7.21 – 7.16 (m, 1H), 7.02 – 6.97 (m, 2H), 5.24 (t, J = 9.5 Hz, 1H), 5.18 (d, J = 9.3 Hz, 1H), 5.02 (s, 2H), 2.94 (t, J = 6.0 Hz, 1H), 2.43 (dd, J = 9.1, 6.0 Hz, 1H), 2.38 – 2.31 (m, 2H), 2.28 (dd, J = 9.4, 7.7 Hz, 1H), 1.55 (d, J = 1.3 Hz, 3H), 0.99 (d, J = 1.3 Hz, 3H).
^{13}C NMR (126 MHz, $CDCl_3$): δ 157.7, 143.2, 139.4, 138.6, 128.3, 128.3, 128.1, 127.6, 126.7, 126.3, 119.9, 83.3, 75.9, 50.1, 36.8, 33.1, 31.0, 25.9, 17.9.
HRMS (ESI, *m/z*): Calcd for $C_{23}H_{26}O_2N^+$ $[M+H]^+$: 348.1958, found: 348.1950.
IR: 2982 (w), 1752 (s), 1653 (m), 1496 (m), 1454 (m), 1375 (m), 1199 (m), 1036 (s), 760 (m), 699 (s).
m.p.: 164 – 166 °C.

8. X-ray Structures:

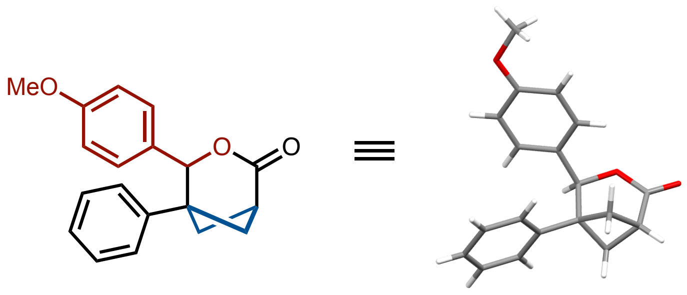

3

A single crystal was obtained by slow evaporation from DCM/pentane solution. A colourless, rod-shaped specimen of $C_{19}H_{18}O_3$, IUMSC 25186, approximate dimensions 0.059×0.084×0.463 mm^3, was placed on a Kapton mount with inert oil for crystal structure determination. The X-ray intensity data were measured on a Bruker D8 Venture KAPPA diffractometer equipped with a microfocus sealed tube (λ = 0.71073 Å) and a multilayer mirror monochromator. The experiment was performed at 153(2) K using an Oxford Cryostream open-nitrogen-flow temperature control unit.

Data collection

The data collection was performed using 0.5° ω and φ scans, frame times of 40 and 1 s, and a detector distance of 40 mm. Overall, 1713 frames were collected with a total exposure time of 15.13 hours. The frames were integrated with the SAINT V8.41 package using a narrow-frame algorithm.[10] The integration of the data using a monoclinic unit cell yielded 99611 reflections to a maximum θ angle of 27.48° (0.77 Å resolution), of which 3412 were independent (average redundancy 27.98, completeness = 99.9%, R_{int} = 5.44%, R_{sig} = 4.56%) and 2854 (83.6%) were greater than $2\sigma(F^2)$. The final cell constants of a = 6.3325(7) Å, b = 14.4177(18) Å, c = 16.336(2) Å, α = 90°, β = 90.879(4)°, γ = 90°, volume = 1491.3(3) $Å^3$, are based upon the refinement of the XYZ-centroids of 9913 reflections above 20 $\sigma(I)$ with 2.50° < 2θ < 27.47°. Data were corrected for absorption effects using the Multi-Scan method in TWINABS Bruker. The calculated minimum and maximum transmission coefficients (based on crystal size) are 0.960 and 0.995.[11] Additional crystal and refinement information can be found in the tables.

Structure solution and refinement

The space group *P*21/*c* (# 14) was determined based on intensity statistics and systematic absences. The structure was solved by SHELXT 2018/2 and refined with full-matrix least squares/difference Fourier cycles using SHELXL-2019/2; Z = 4 for the formula unit $C_{19}H_{18}O_3$.[12,13] Non-hydrogen atoms were refined with anisotropic displacement parameters. The hydrogen atoms were placed in ideal positions and refined as riding atoms with relative isotropic displacement parameters. The final anisotropic full-matrix least-squares refinement on F^2 with 200 variables against 3412 data points and converged at R_1 = 5.12%, for the observed data and wR_2 = 13.16%

for all data. The goodness-of-fit on F^2 was 1.09. The largest peak in the final difference electron density synthesis was 0.48 $e^-/Å^3$ and the deepest hole was −0.30 $e^-/Å^3$ with an RMS deviation of 0.076 $e^-/Å^3$. On the basis of the final model, the calculated density was 1.31 g/cm^3 and *F*(000), 624 e^-. The crystal was non-merohedrally twinned, only the major domain (~80%) was considered in the refinement.

Table S1. Crystal data and structure refinement for 3.

Empirical formula	C19 H18 O3	
Formula weight	294.33	
Crystal color, shape, size	colourless rod, 0.463 × 0.084 × 0.059 mm^3	
Temperature	153(2) K	
Wavelength	0.71073 Å	
Crystal system, space group	Monoclinic, $P2_1/c$	
Unit cell dimensions	a = 6.3325(7) Å	α = 90°.
	b = 14.4177(18) Å	β = 90.879(4)°.
	c = 16.336(2) Å	γ = 90°.
Volume	1491.3(3) Å^3	
Z	4	
Density (calculated)	1.311 Mg/m^3	
Absorption coefficient	0.088 mm^{-1}	
F(000)	624	
**Data collection**		
Diffractometer	Venture D8, Bruker	
Source, detector	Incoatec Iμ3.0, Photon III	
Theta range for data collection	2.494 to 27.482°.	
Index ranges	-8<=h<=7, -18<=k<=18, -21<=l<=21	
Reflections collected	95465	
Independent reflections	3412 [R_{int} = 0.1717]	
Observed Reflections	2854	
Completeness to theta = 25.242°	99.9 %	
**Solution and Refinement**		
Absorption correction	Semi-empirical from equivalents	
Max. and min. transmission	0.7456 and 0.6603	
Solution	Intrinsic methods	
Refinement method	Full-matrix least-squares on F^2	
Weighting scheme	$w = [\sigma^2Fo^2 + AP^2 + BP]^{-1}$, with	
	$P = (Fo^2 + 2\ Fc^2)/3$, A = 0.0520, B = 1.0666	
Data / restraints / parameters	3412 / 0 / 200	
Goodness-of-fit on F^2	1.091	

Final R indices [I>2σ(I)]	R1 = 0.0512, wR2 = 0.1223
R indices (all data)	R1 = 0.0740, wR2 = 0.1316
Extinction coefficient	n/a
Largest diff. peak and hole	0.479 and -0.302 e.$Å^{-3}$

9. NMR Spectra:

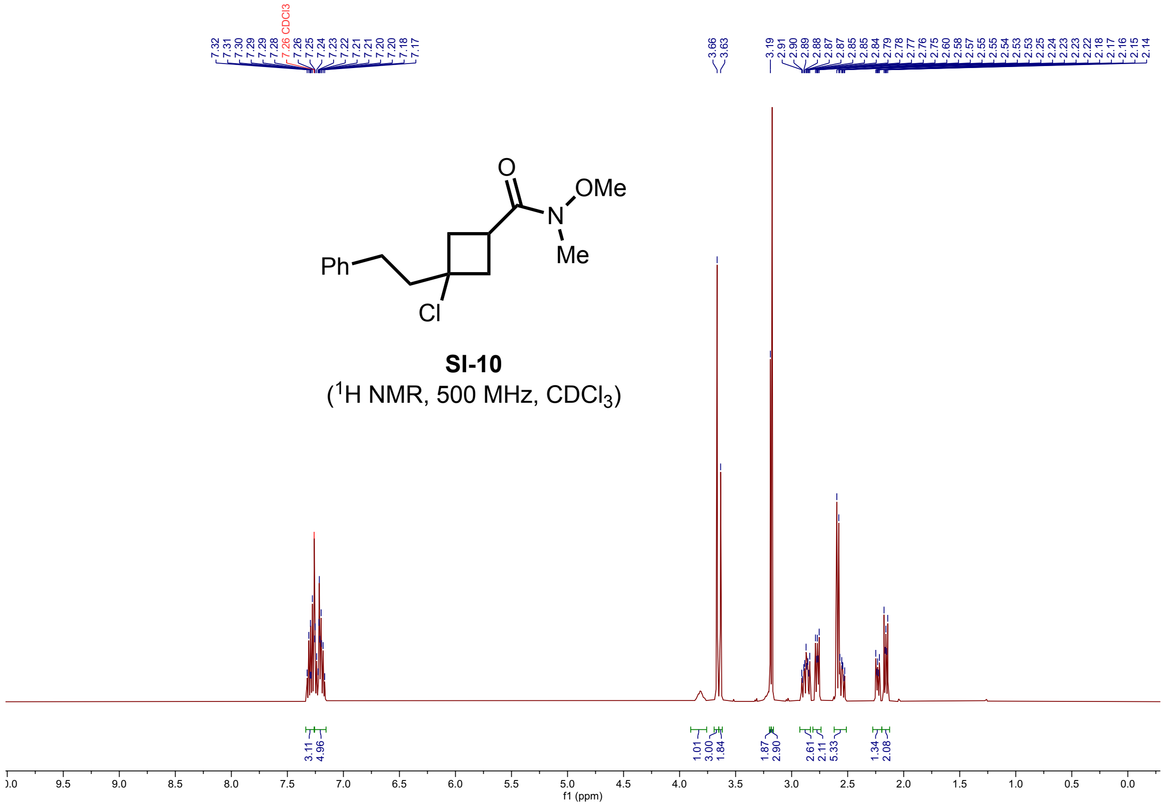

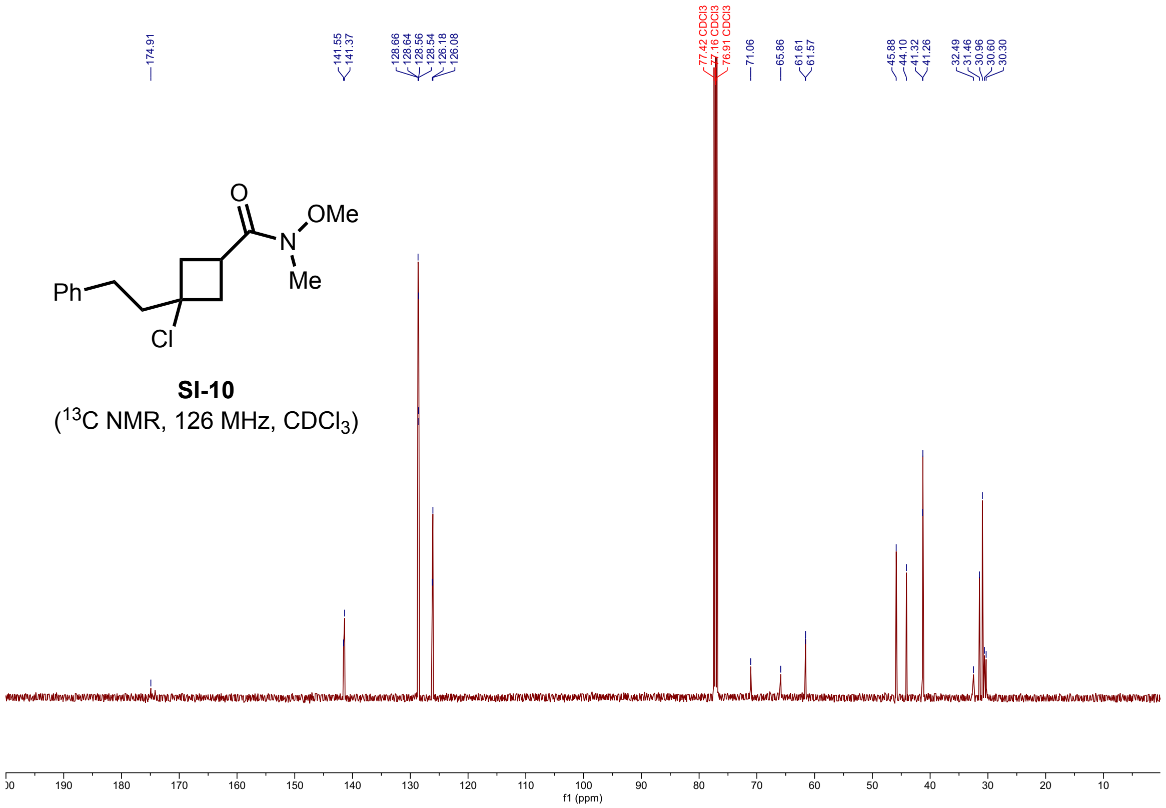

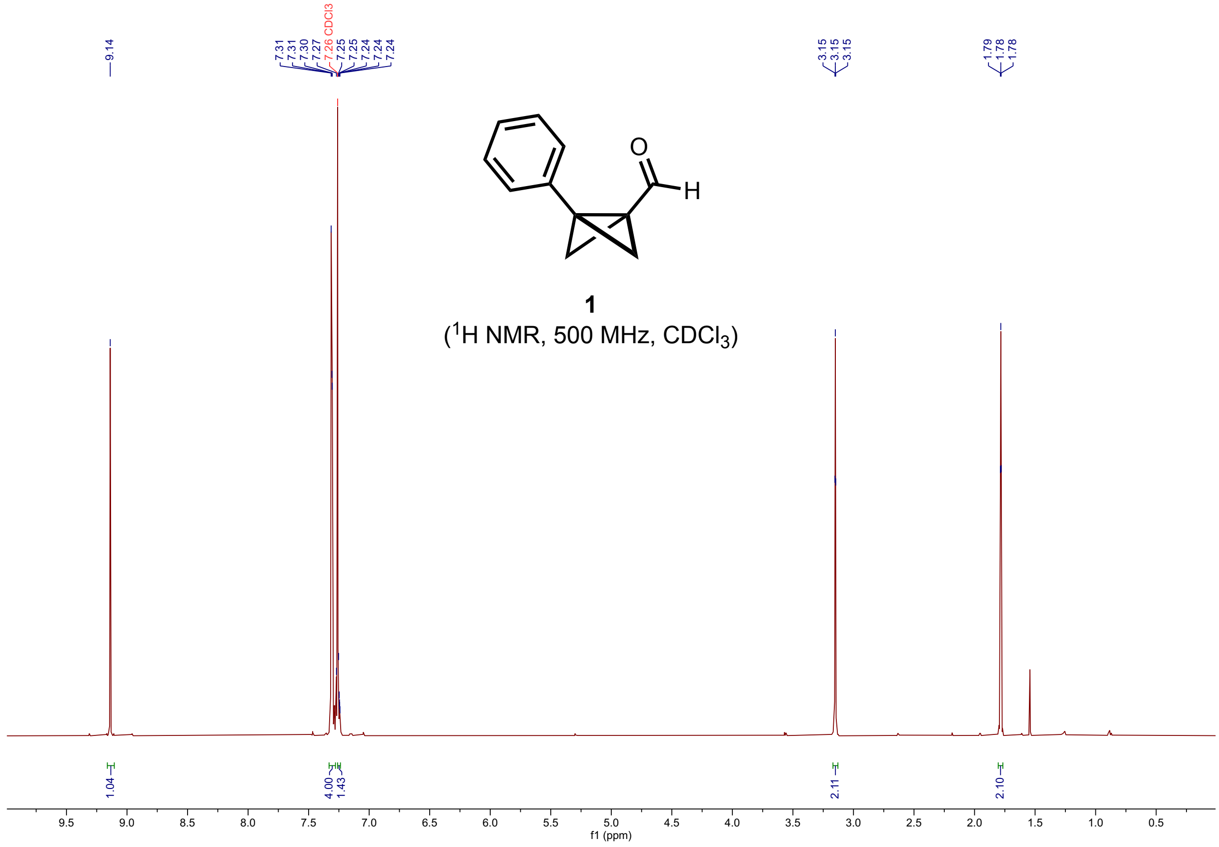
9.14
7.31
7.31
7.30
7.27
7.26 CDCl3
7.25
7.25
7.24
7.24
7.24
3.15
3.15
3.15
1.79
1.78
1.78
O
H
1
(^{1}H NMR, 500 MHz, $CDCl_3$)
1.04
4.00
1.43
2.11
2.10
9.5 9.0 8.5 8.0 7.5 7.0 6.5 6.0 5.5 5.0 4.5 4.0 3.5 3.0 2.5 2.0 1.5 1.0 0.5
f1 (ppm)

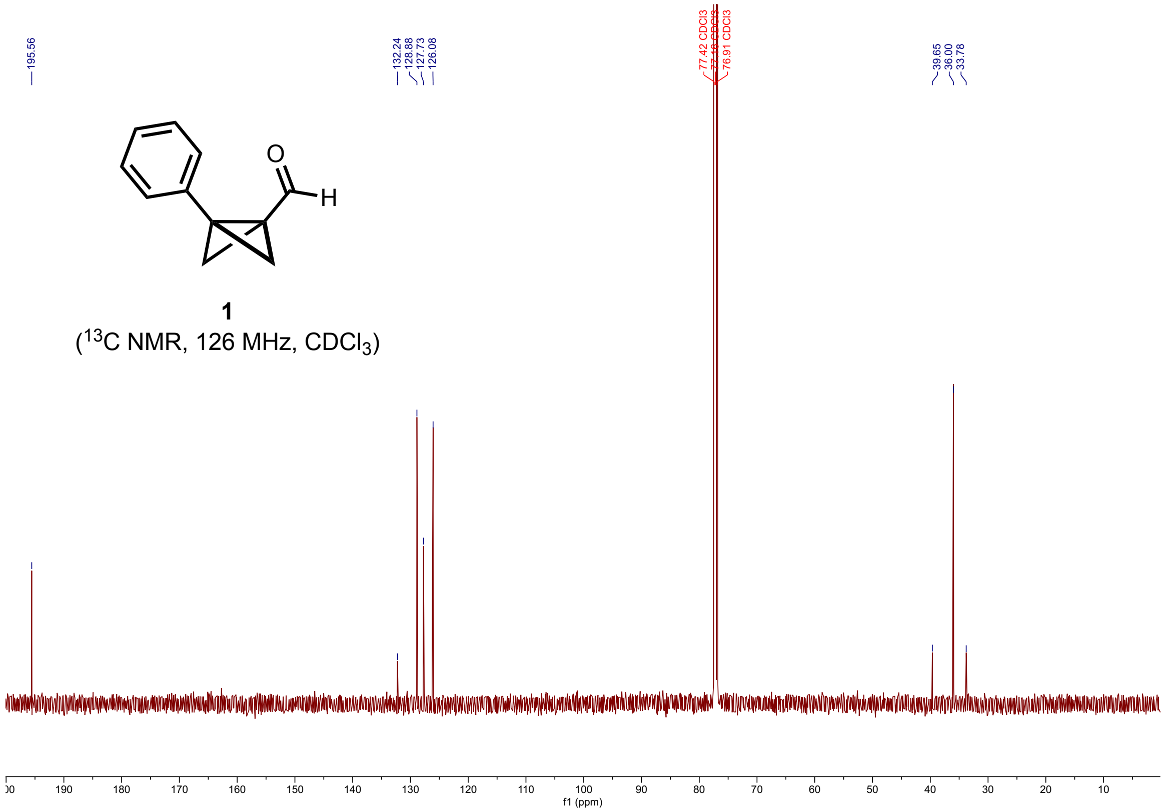
195.56
132.24
128.88
127.73
126.08
77.42 CDCl3
77.16 CDCl3
76.91 CDCl3
39.65
36.00
33.78
O
H
1
(^{13}C NMR, 126 MHz, $CDCl_3$)
190 180 170 160 150 140 130 120 110 100 90 80 70 60 50 40 30 20 10
f1 (ppm)

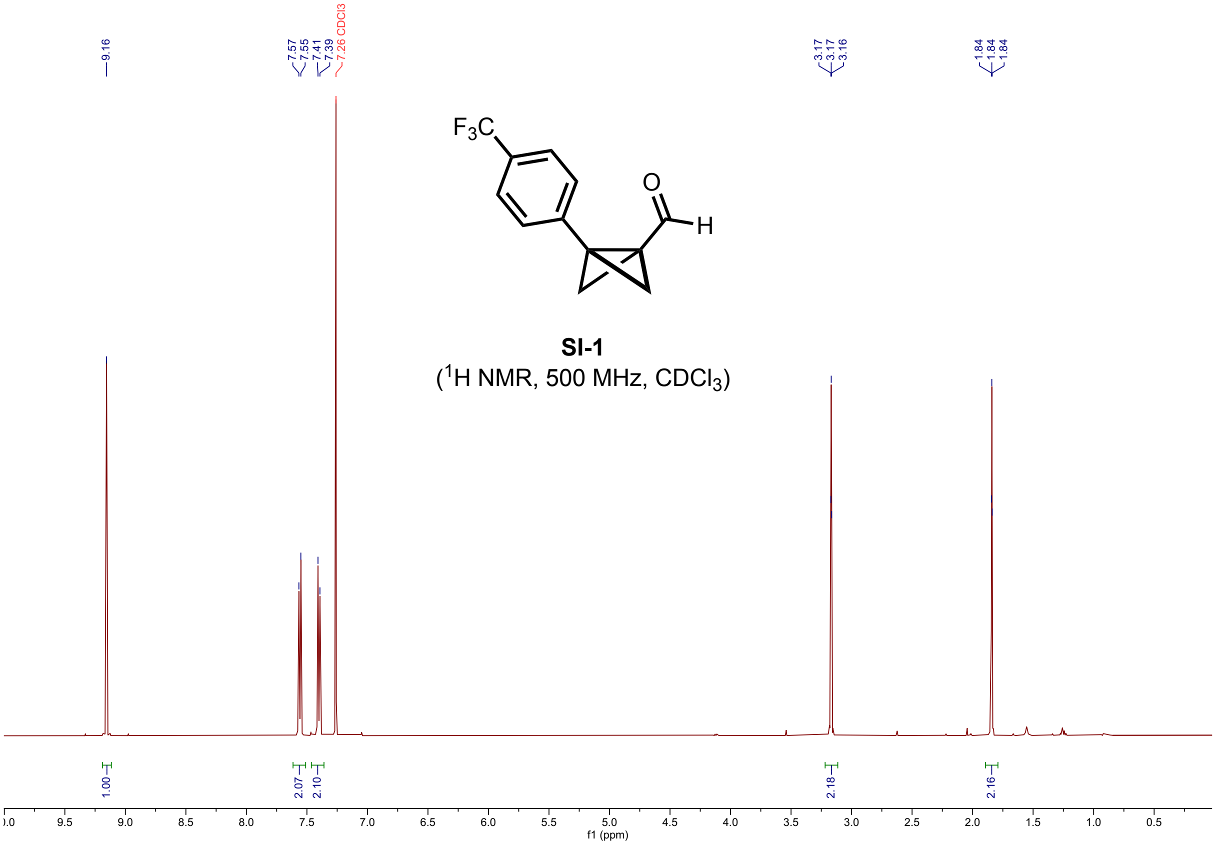

SI-1
(^{1}H NMR, 500 MHz, $CDCl_3$)

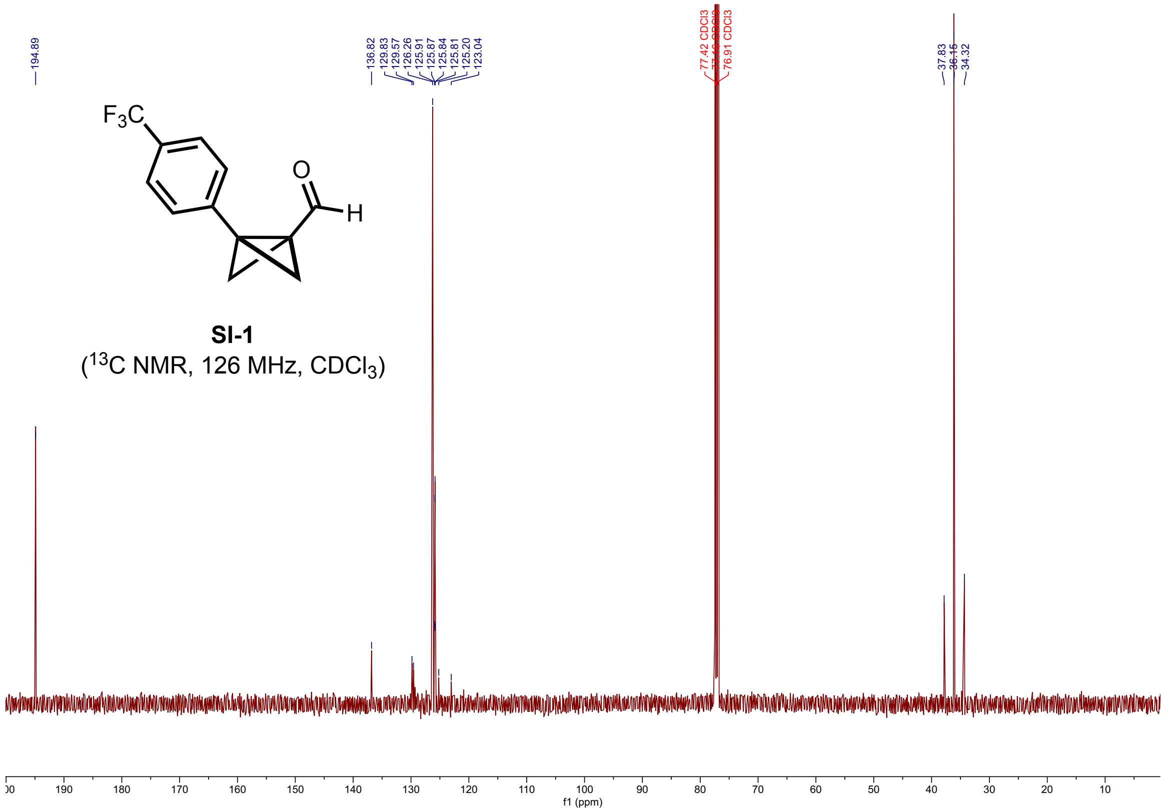

SI-1
(^{13}C NMR, 126 MHz, $CDCl_3$)

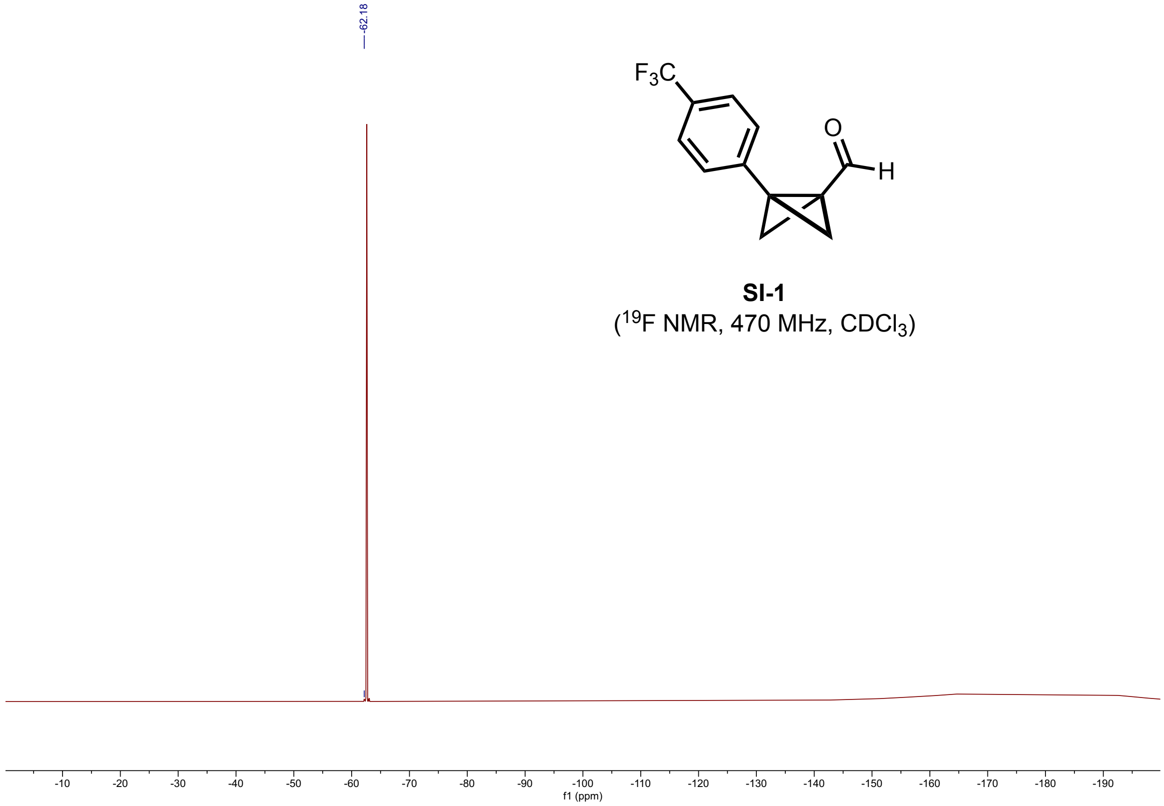

SI-1
(^{19}F NMR, 470 MHz, $CDCl_3$)

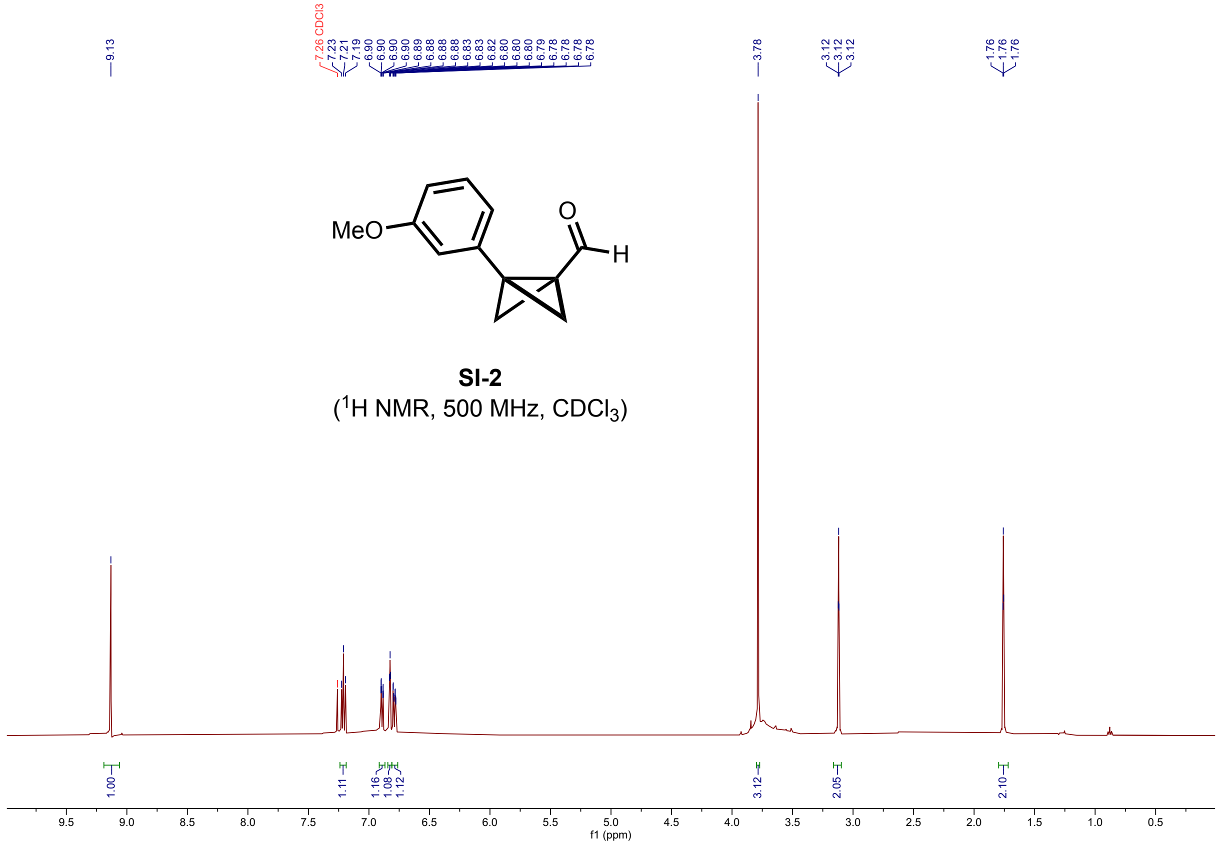

SI-2
(^{1}H NMR, 500 MHz, $CDCl_3$)

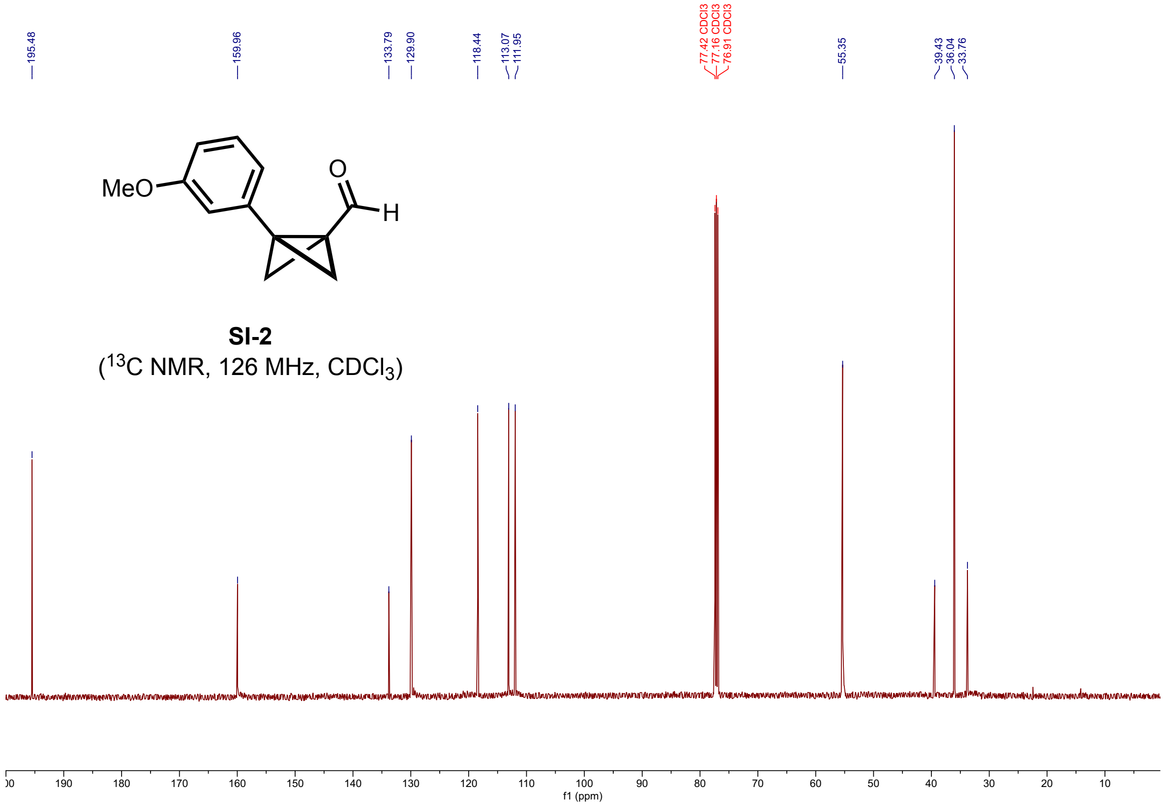

SI-2
(^{13}C NMR, 126 MHz, $CDCl_3$)

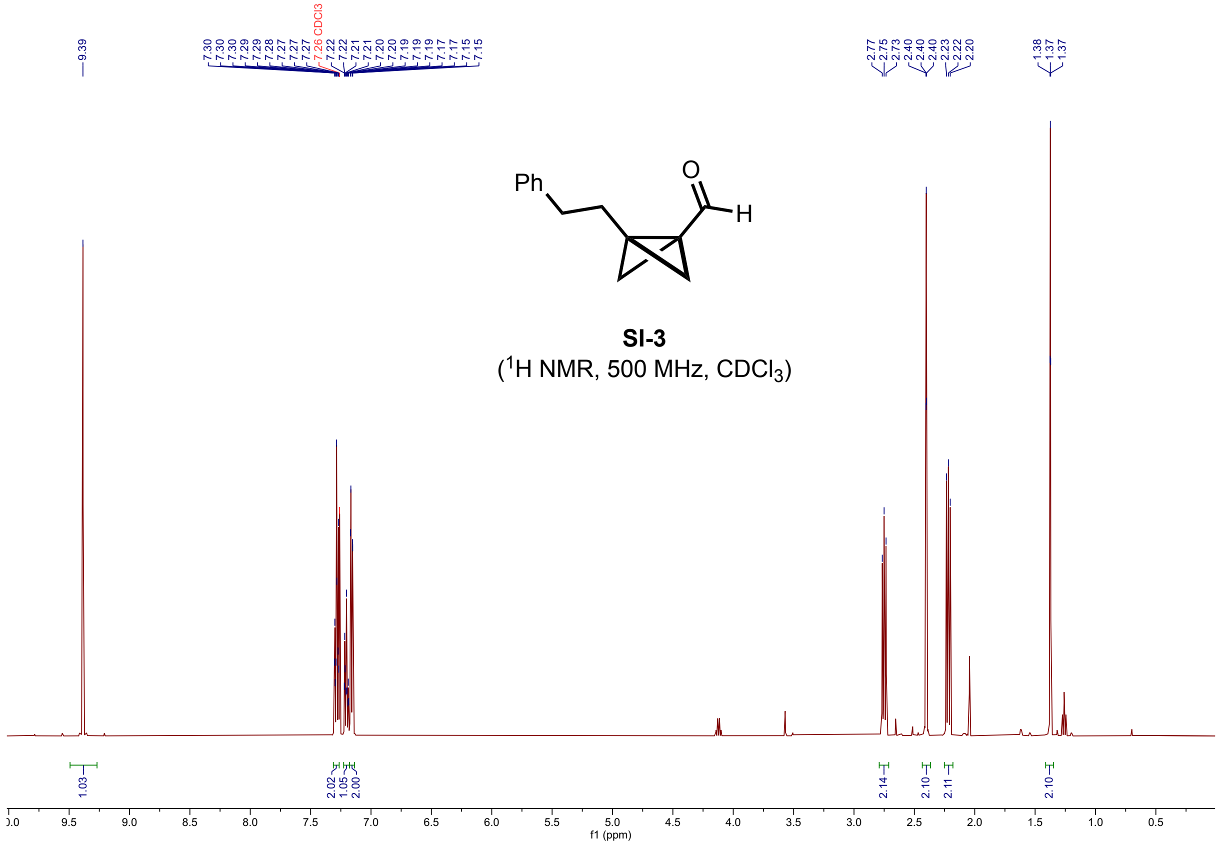
9.39
7.30 7.30 7.29 7.29 7.28 7.27 7.27 7.26 CDCl3 7.22 7.22 7.21 7.21 7.20 7.20 7.19 7.19 7.19 7.17 7.17 7.15 7.15
2.77 2.75 2.73 2.40 2.40 2.40 2.23 2.22 2.20
1.38 1.37 1.37
Ph
O
H
SI-3
(1H NMR, 500 MHz, CDCl3)
1.03
2.02 1.05 2.00
2.14 2.10 2.11 2.10
f1 (ppm)

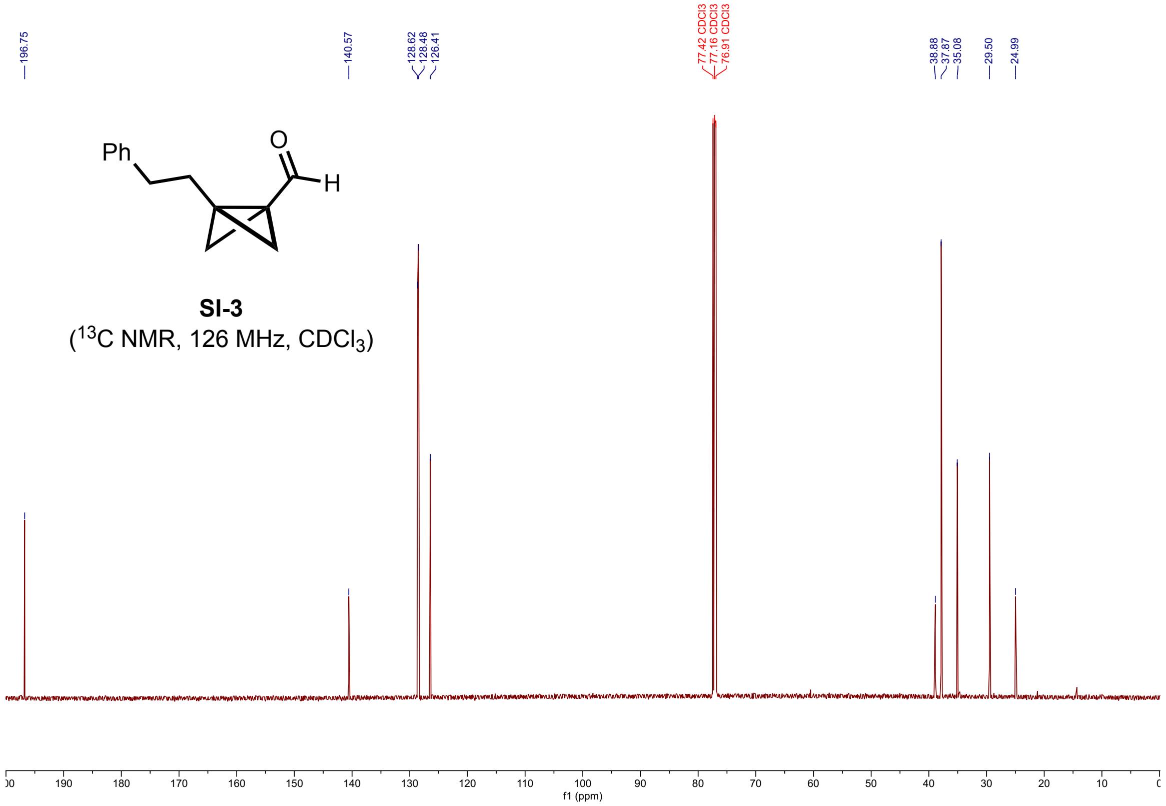
196.75
140.57
128.62 128.48 126.41
77.42 CDCl3 77.16 CDCl3 76.91 CDCl3
38.88 37.87 35.08 29.50 24.99
Ph
O
H
SI-3
(13C NMR, 126 MHz, CDCl3)
f1 (ppm)

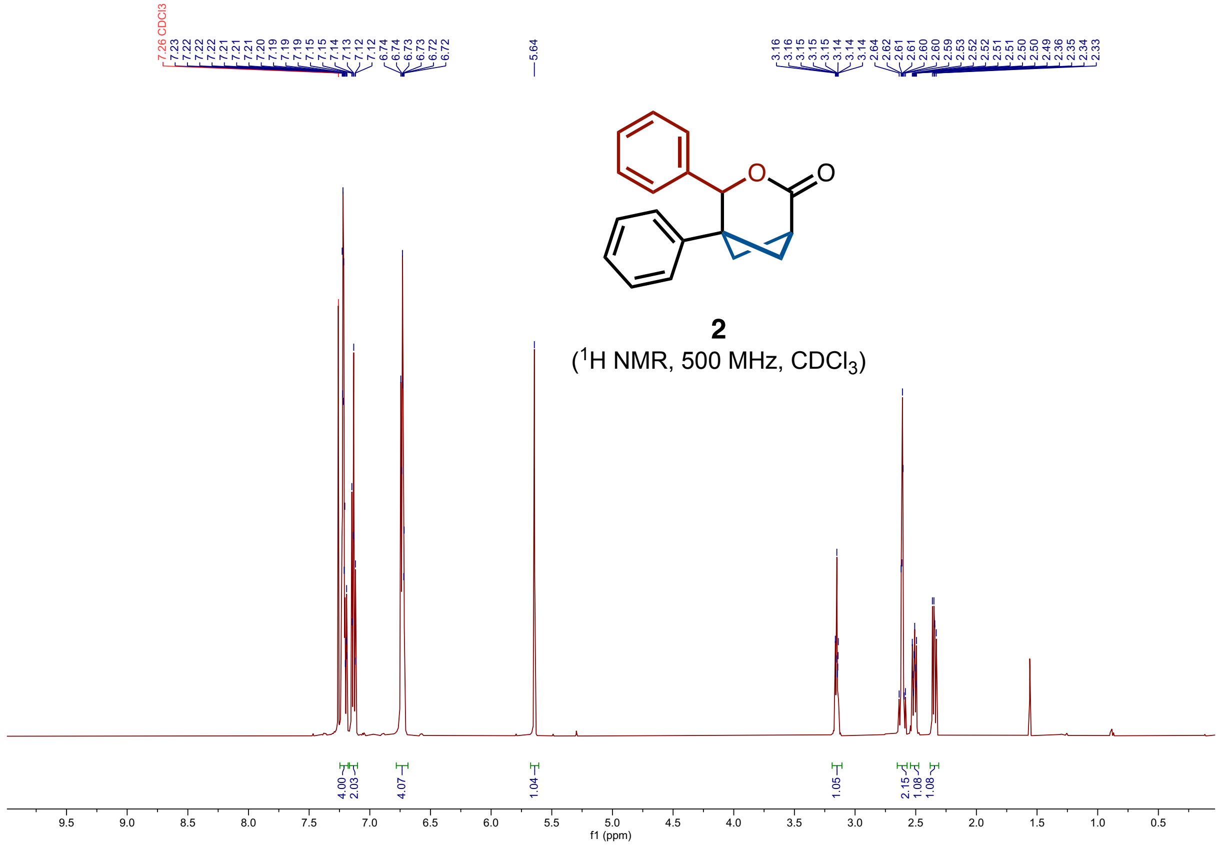

2
(^{1}H NMR, 500 MHz, $CDCl_3$)

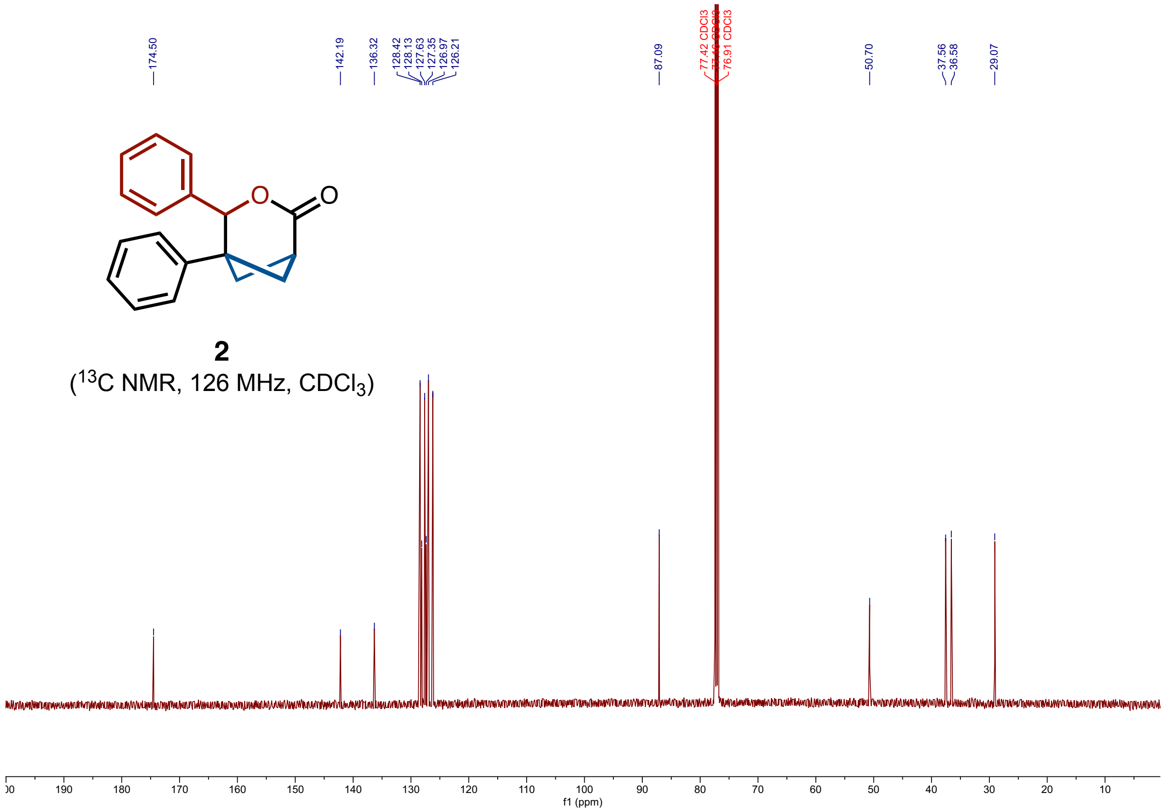

2
(^{13}C NMR, 126 MHz, $CDCl_3$)

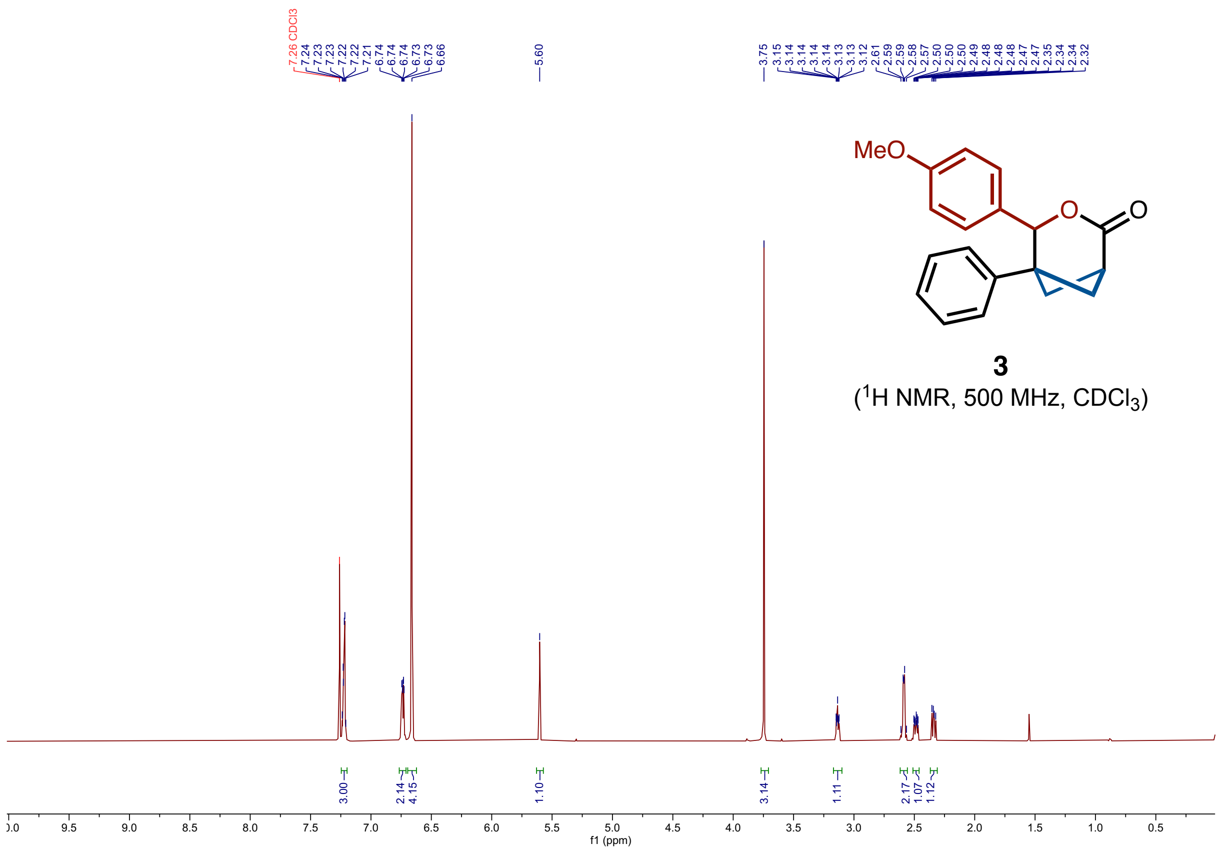
MeO
O
O
3
(¹H NMR, 500 MHz, CDCl₃)
f1 (ppm)

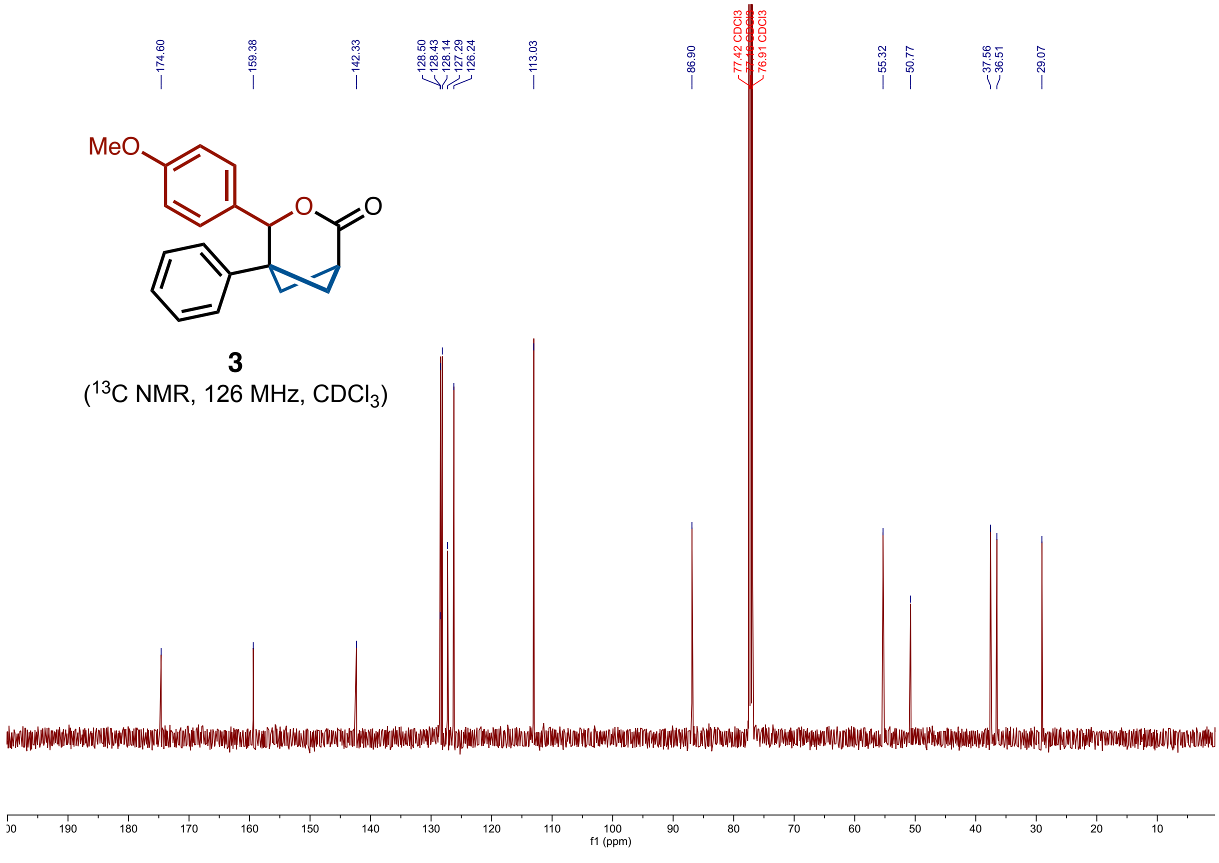
MeO
O
O
3
(¹³C NMR, 126 MHz, CDCl₃)
f1 (ppm)

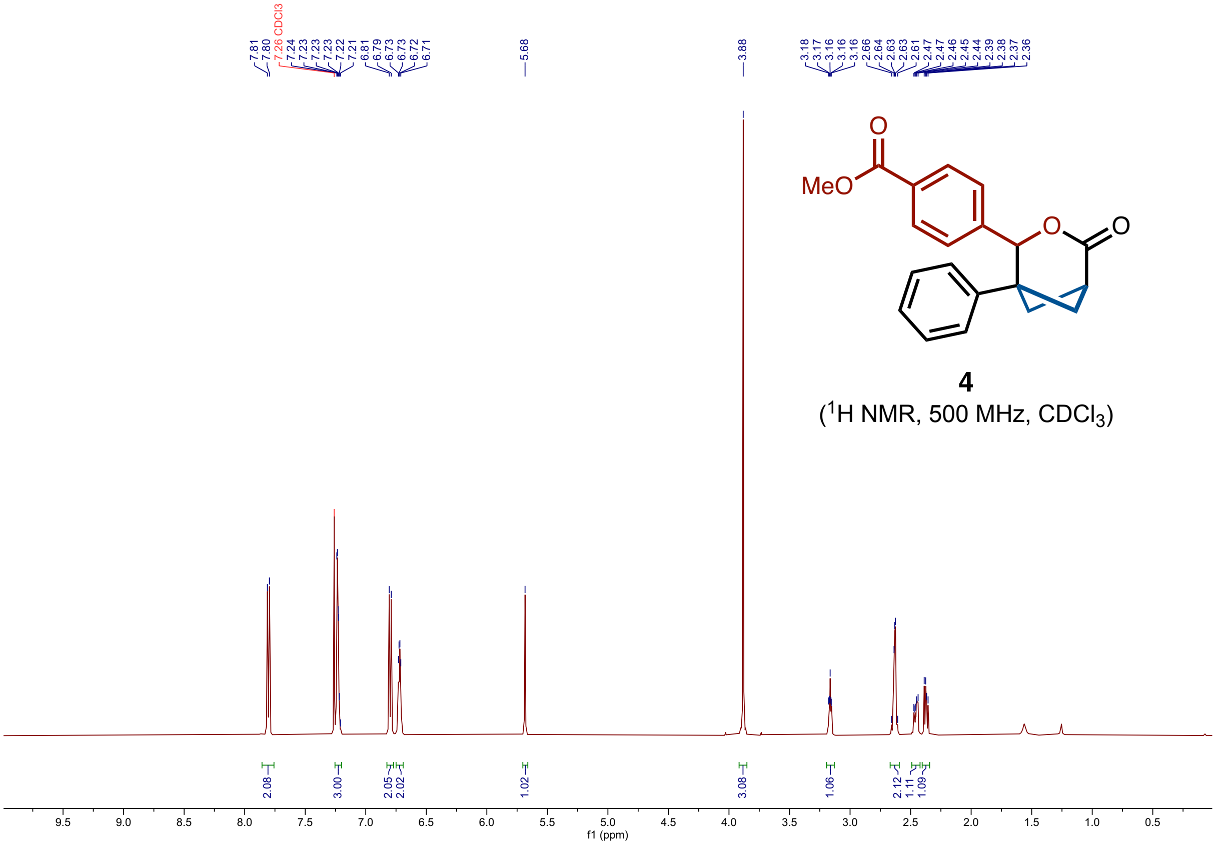

4

(^{1}H NMR, 500 MHz, $CDCl_3$)

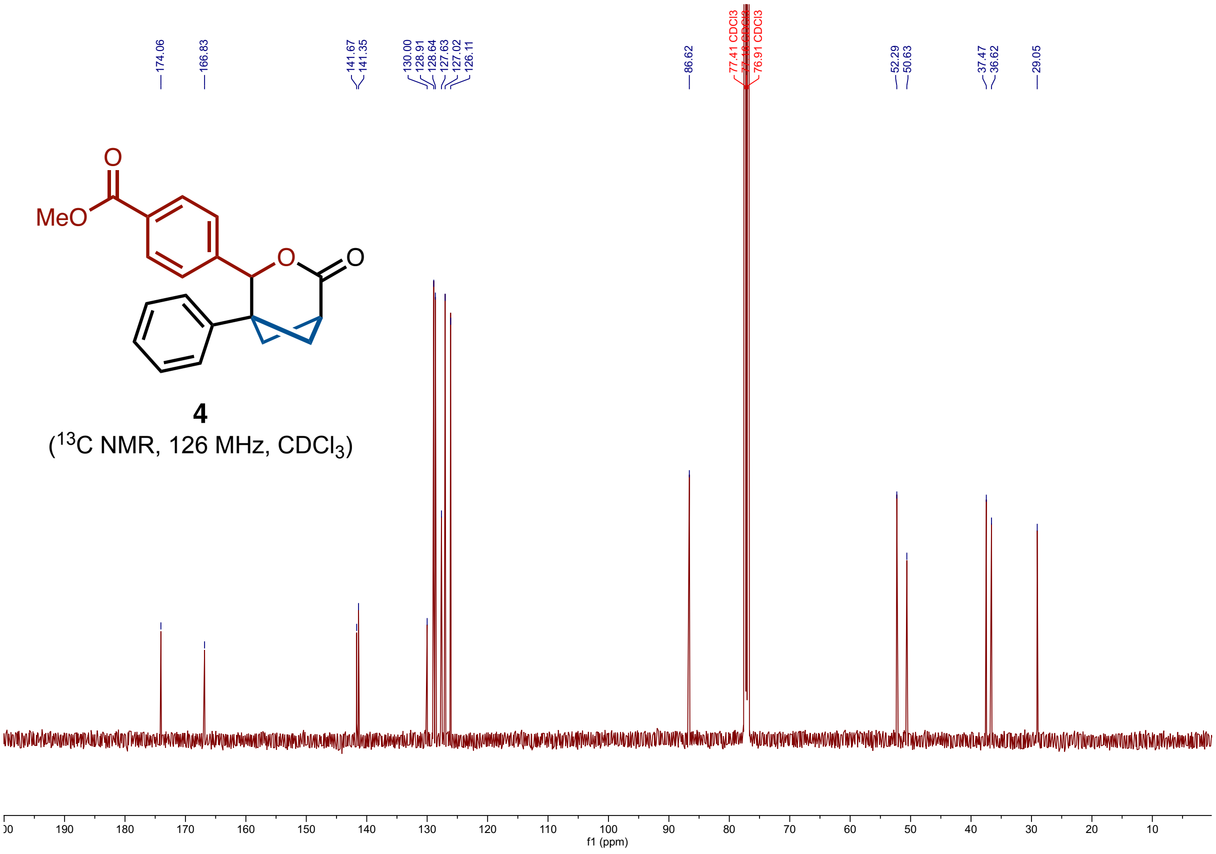

4

(^{13}C NMR, 126 MHz, $CDCl_3$)

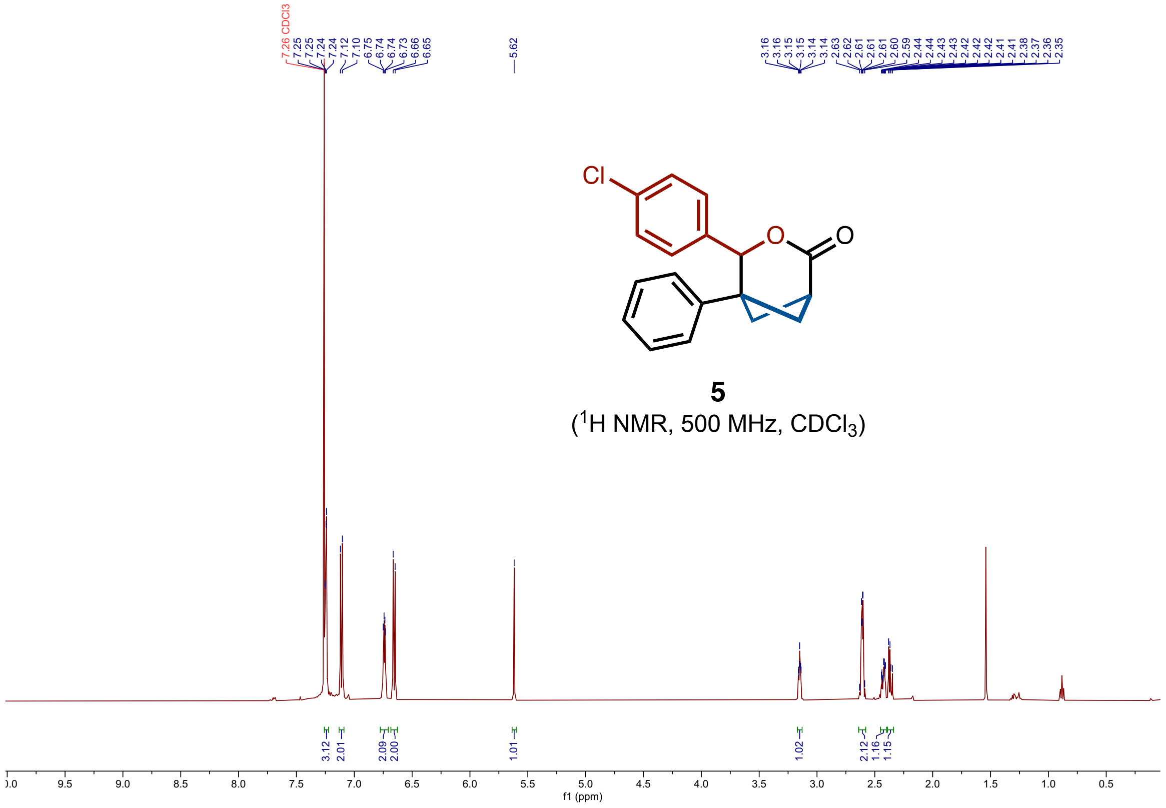
5
(1H NMR, 500 MHz, CDCl3)
f1 (ppm)

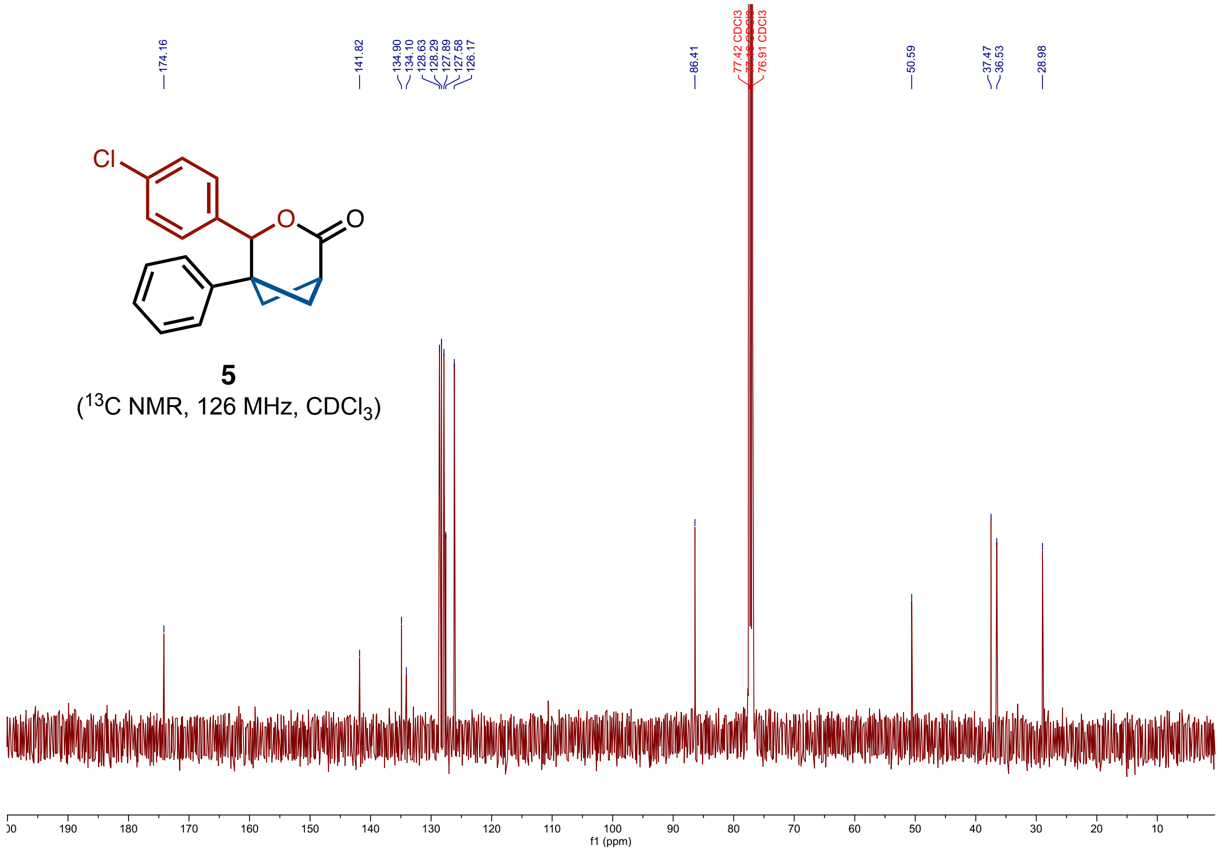
5
(13C NMR, 126 MHz, CDCl3)
f1 (ppm)

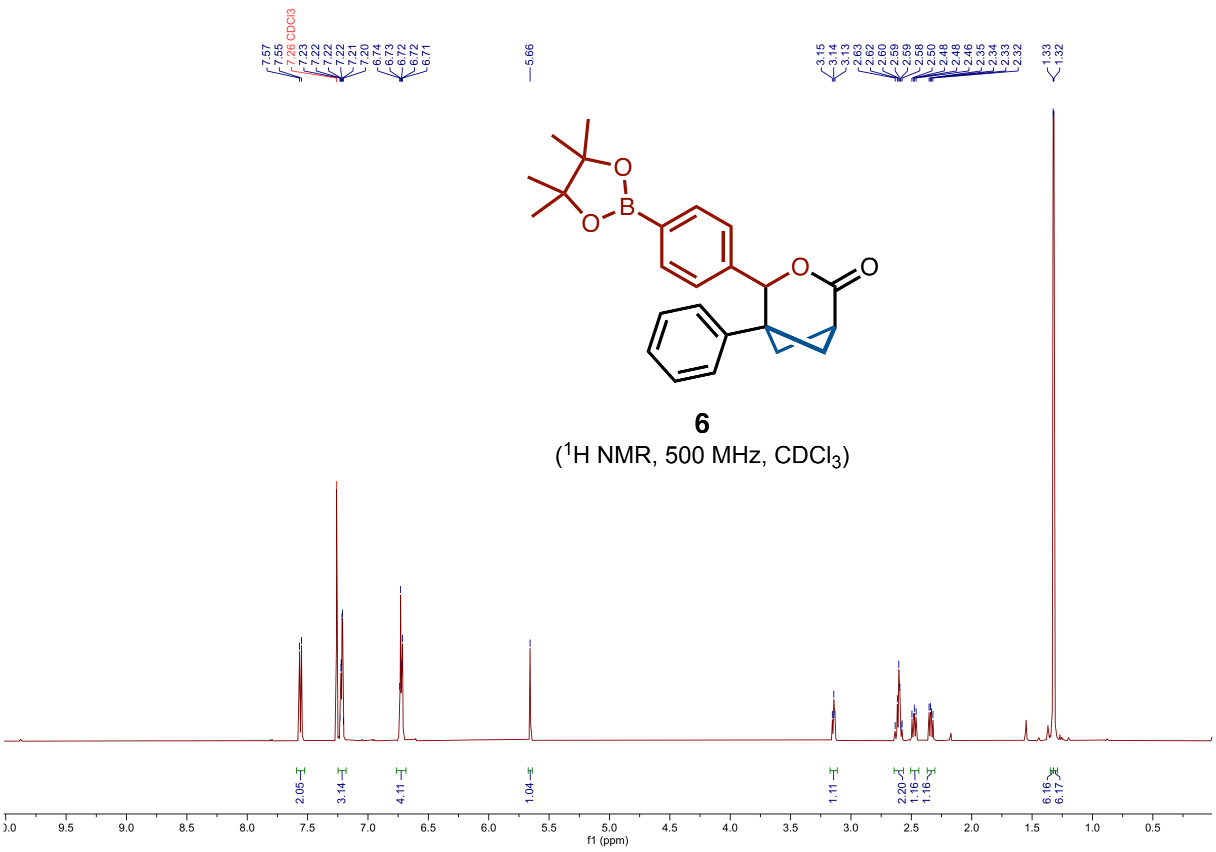
7.57
7.55
7.26 CDCl3
7.23
7.22
7.22
7.22
7.21
7.20
6.74
6.73
6.72
6.72
6.71
5.66
3.15
3.14
3.13
2.63
2.62
2.60
2.59
2.59
2.58
2.50
2.48
2.46
2.35
2.34
2.33
2.32
1.33
1.32
O
B
O
O
O
6
(1H NMR, 500 MHz, CDCl3)
2.05
3.14
4.11
1.04
1.11
2.20
1.16
1.16
6.16
6.17
f1 (ppm)

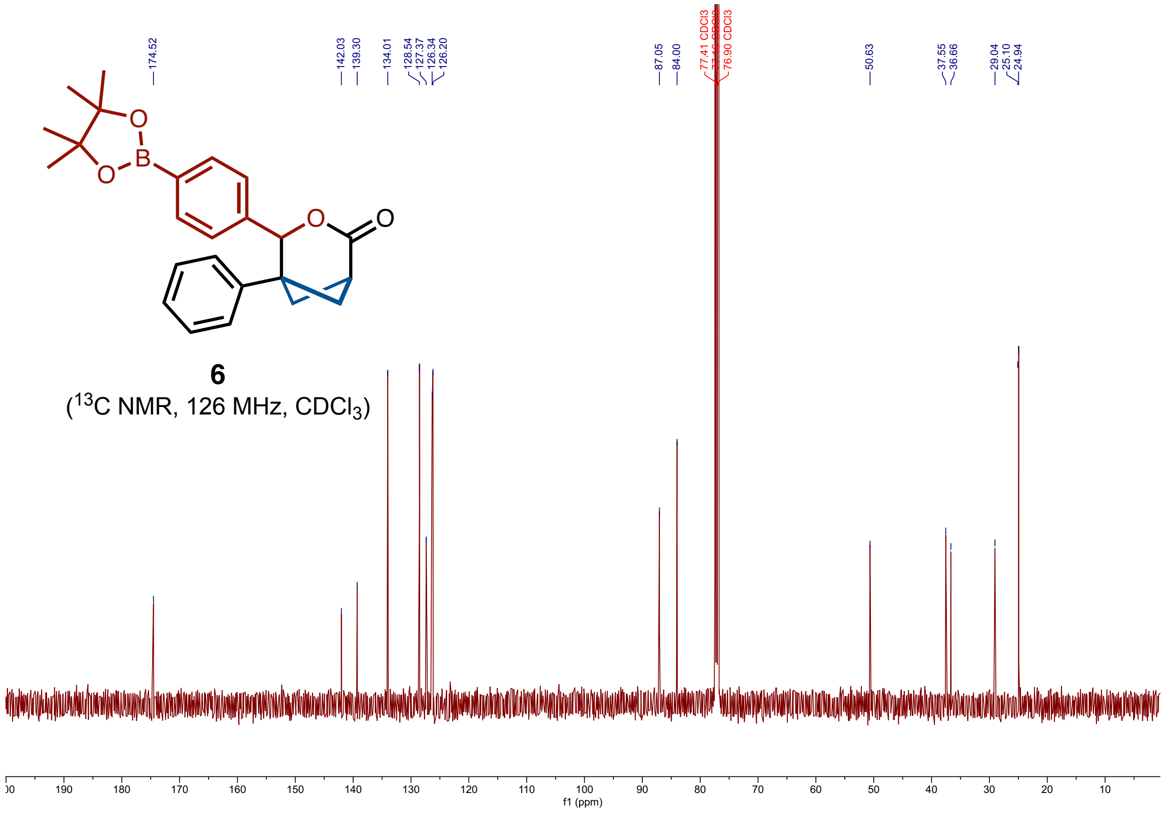
174.52
142.03
139.30
134.01
128.54
127.37
126.34
126.20
87.05
84.00
77.41 CDCl3
76.90 CDCl3
50.63
37.55
36.66
29.04
25.10
24.94
O
B
O
O
O
6
(13C NMR, 126 MHz, CDCl3)
f1 (ppm)

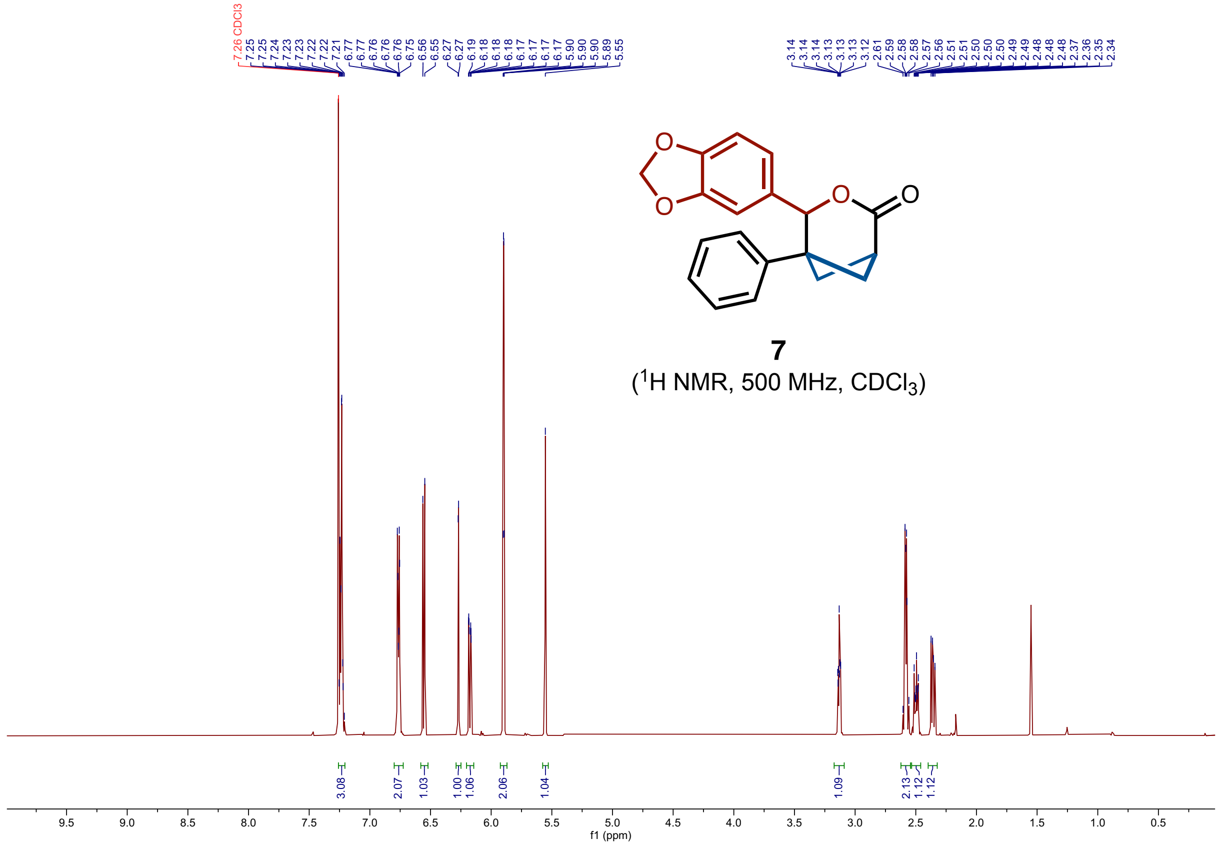

7

(^{1}H NMR, 500 MHz, $CDCl_3$)

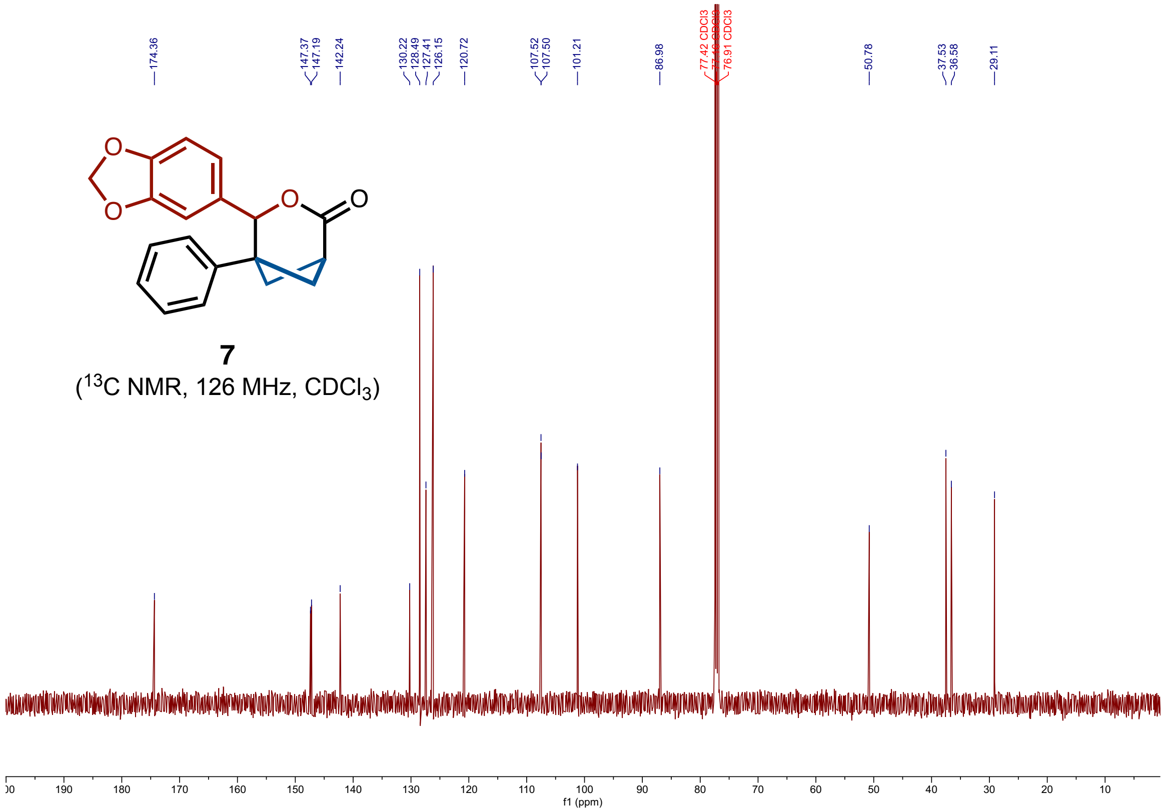

7

(^{13}C NMR, 126 MHz, $CDCl_3$)

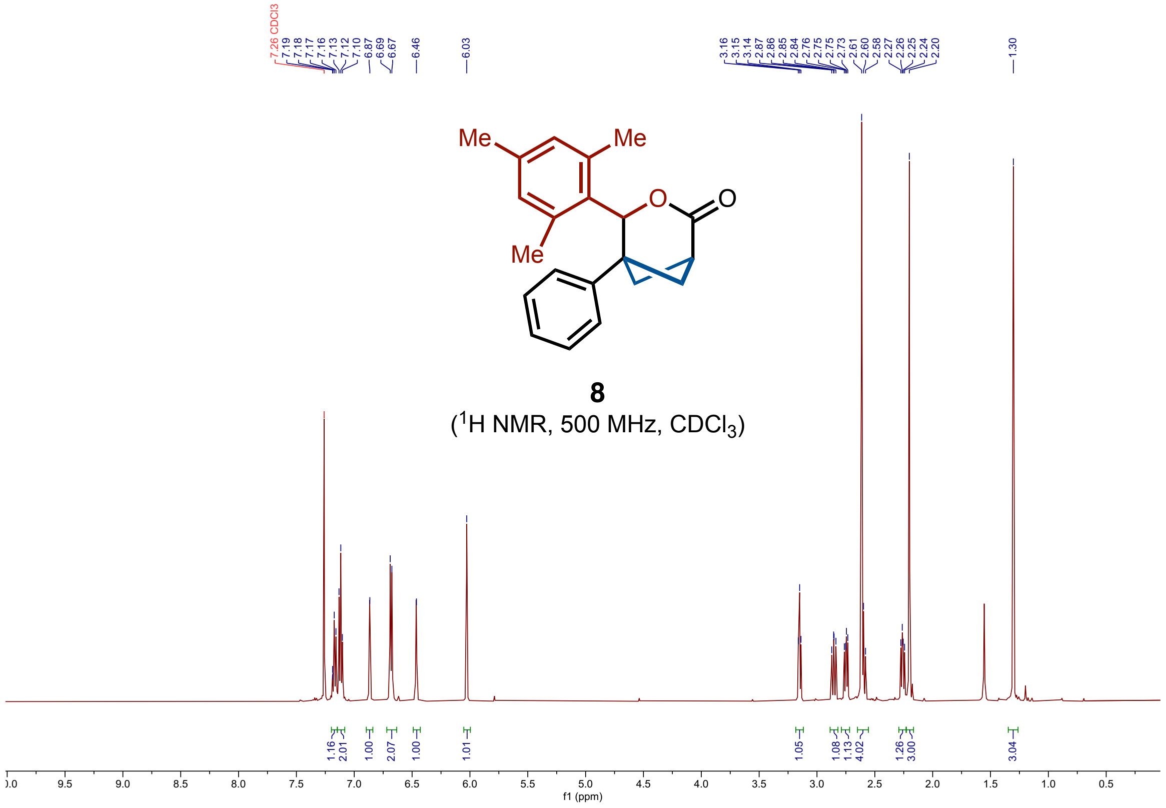

8
(^{1}H NMR, 500 MHz, $CDCl_3$)

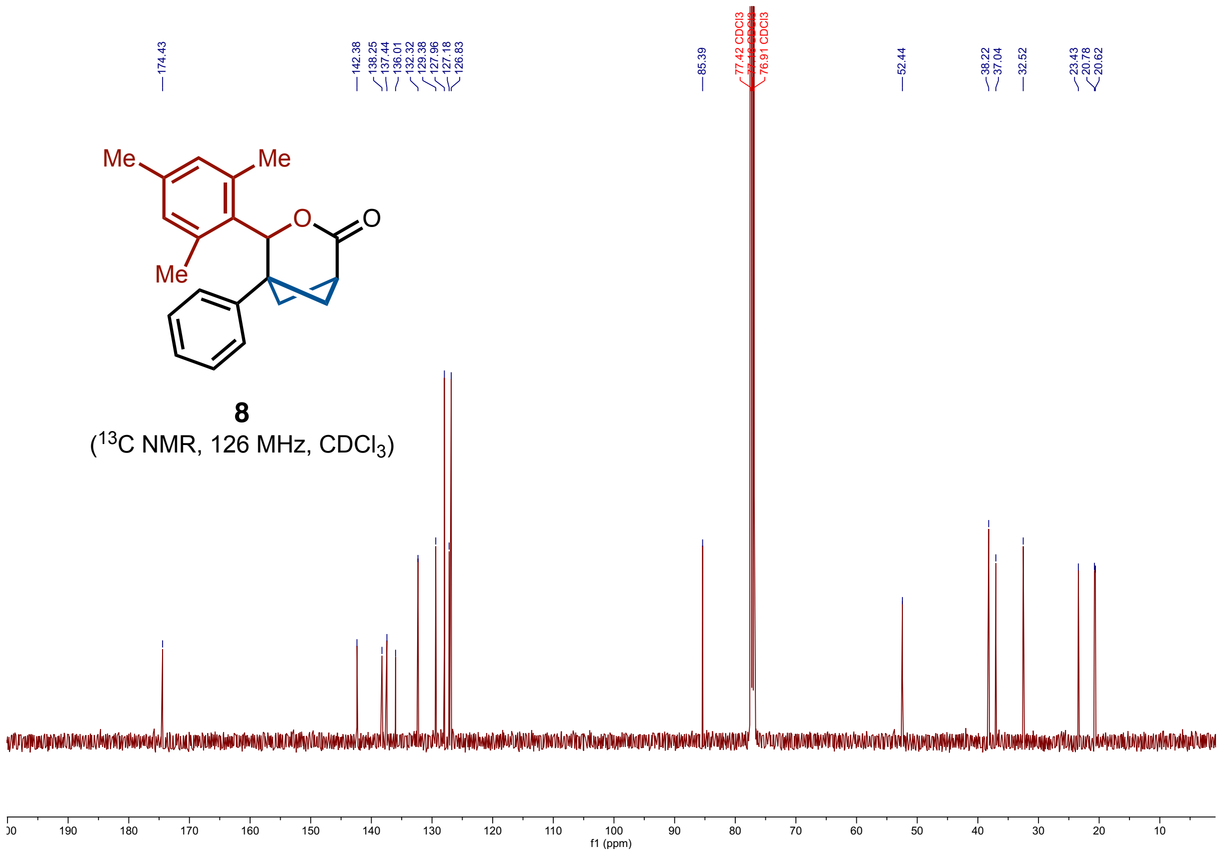

8
(^{13}C NMR, 126 MHz, $CDCl_3$)

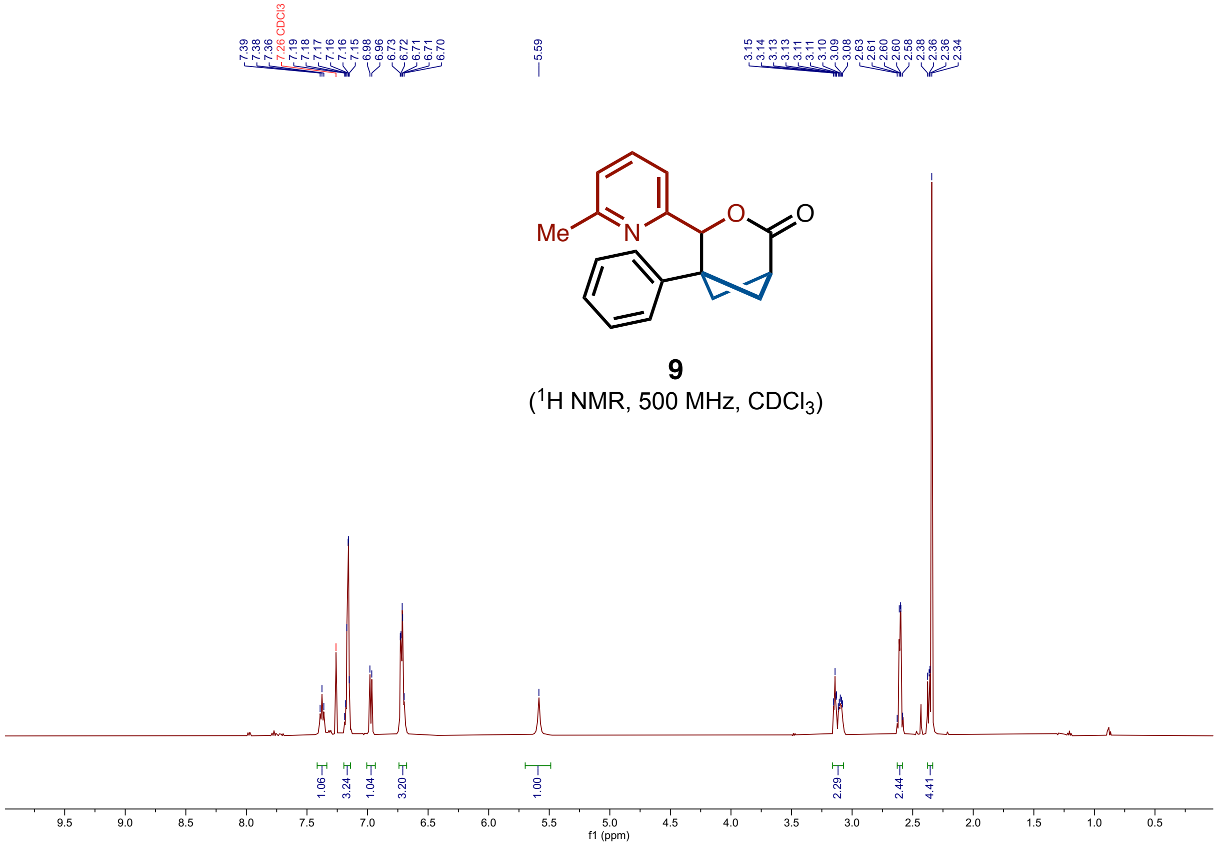

9

(^{1}H NMR, 500 MHz, $CDCl_3$)

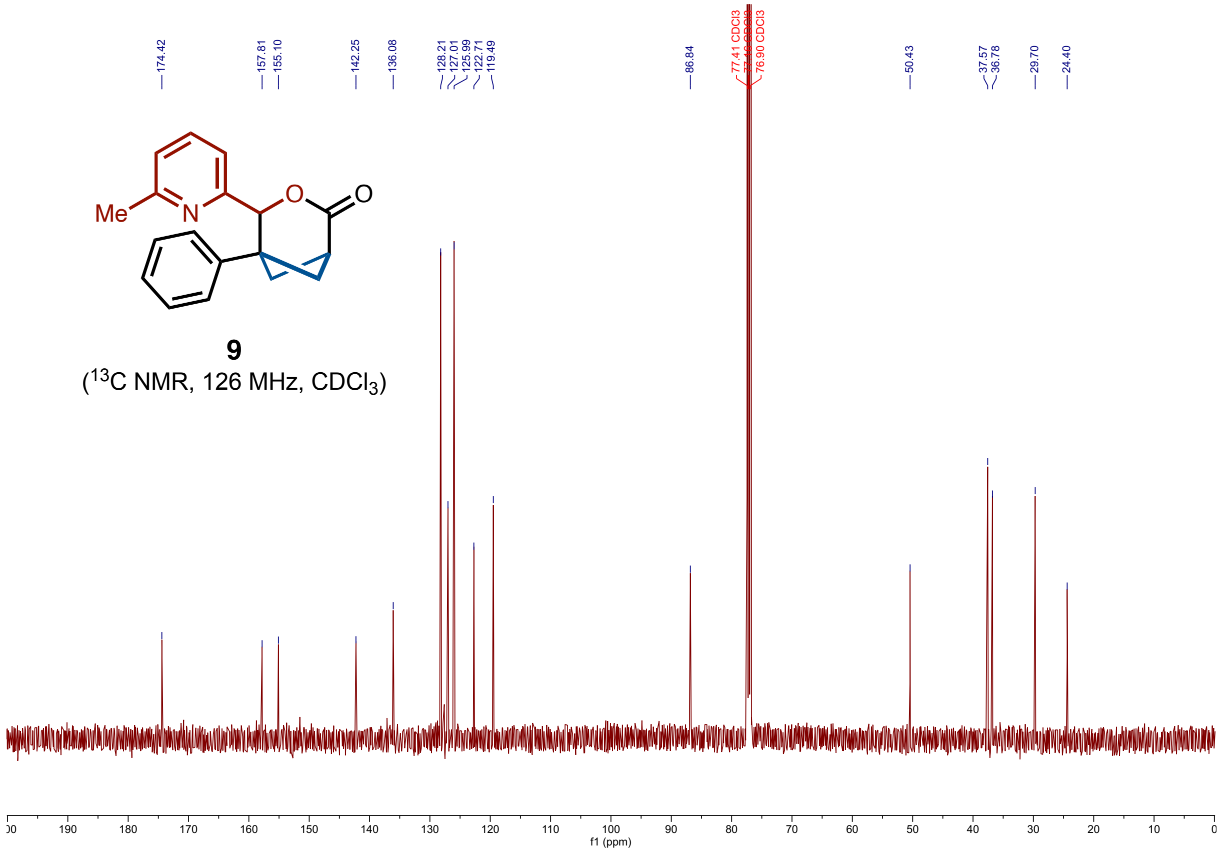

9

(^{13}C NMR, 126 MHz, $CDCl_3$)

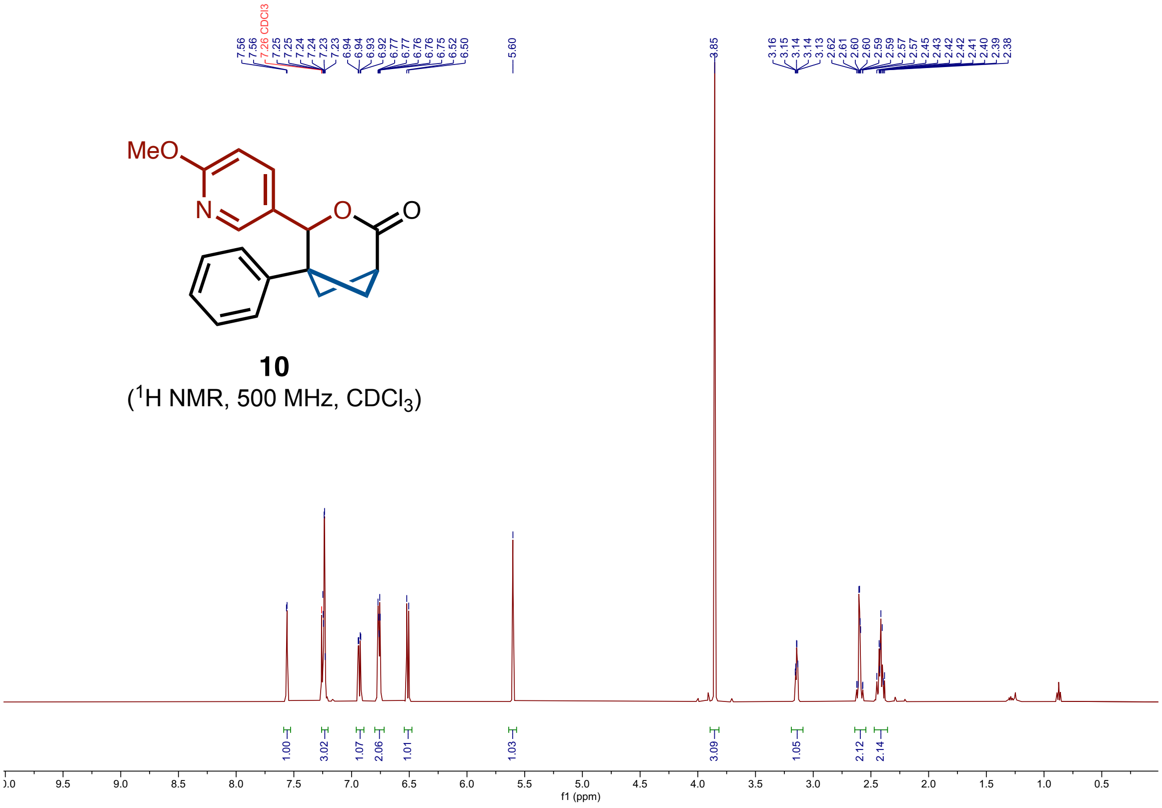

10

(^{1}H NMR, 500 MHz, $CDCl_3$)

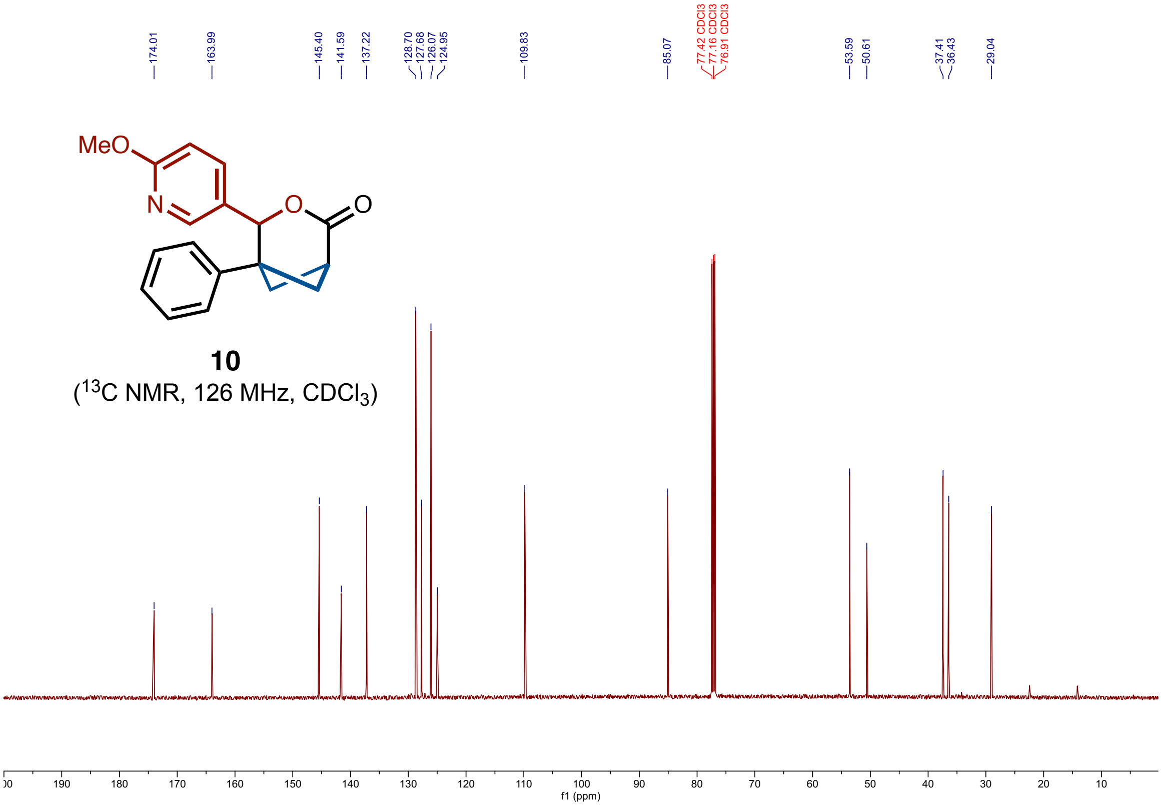

10

(^{13}C NMR, 126 MHz, $CDCl_3$)

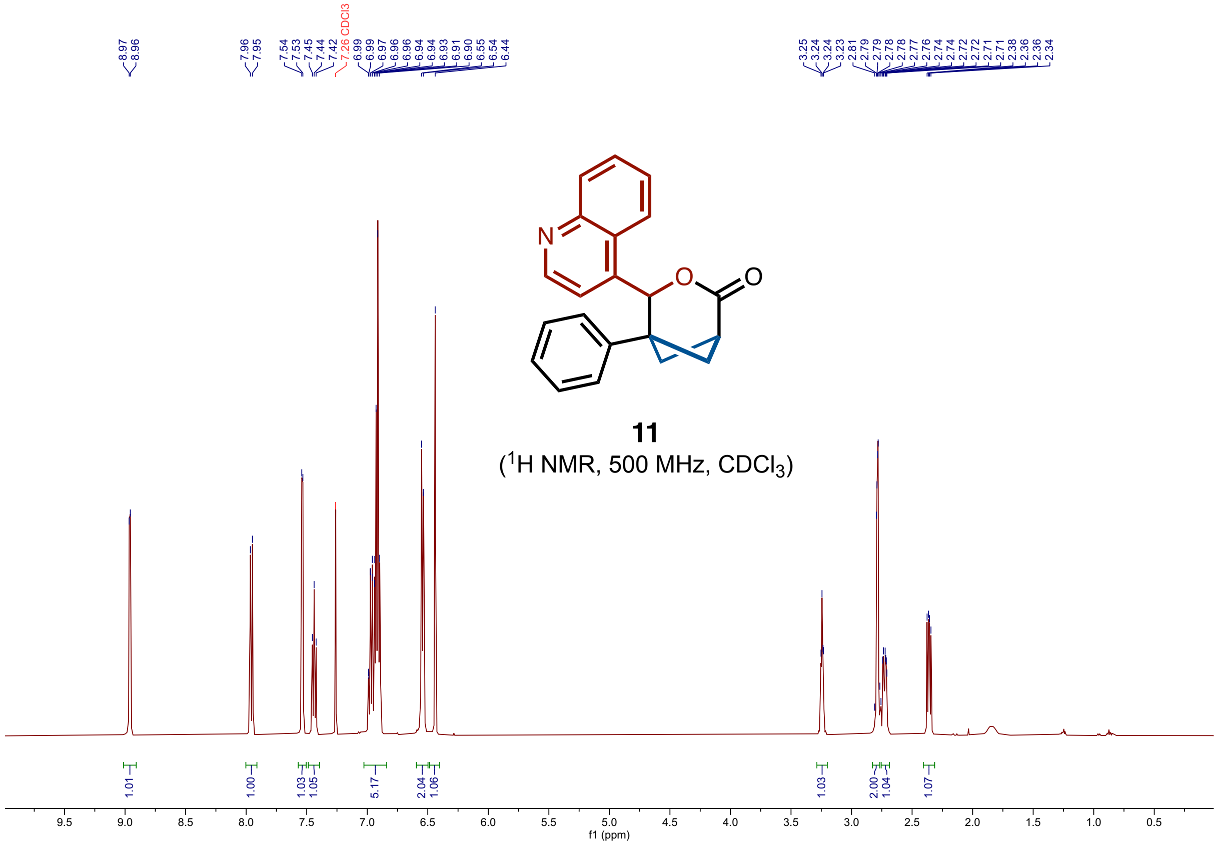
8.97
8.96
7.96
7.95
7.54
7.53
7.45
7.44
7.42
7.26 CDCl3
6.99
6.99
6.97
6.96
6.96
6.94
6.94
6.93
6.91
6.90
6.55
6.54
6.44
3.25
3.24
3.24
3.23
2.81
2.79
2.78
2.78
2.77
2.76
2.74
2.74
2.72
2.72
2.71
2.71
2.38
2.36
2.36
2.34
N
O
O
11
(¹H NMR, 500 MHz, CDCl₃)
1.01
1.00
1.03
1.05
5.17
2.04
1.06
1.03
2.00
1.04
1.07
9.5
9.0
8.5
8.0
7.5
7.0
6.5
6.0
5.5
5.0
4.5
4.0
3.5
3.0
2.5
2.0
1.5
1.0
0.5
f1 (ppm)

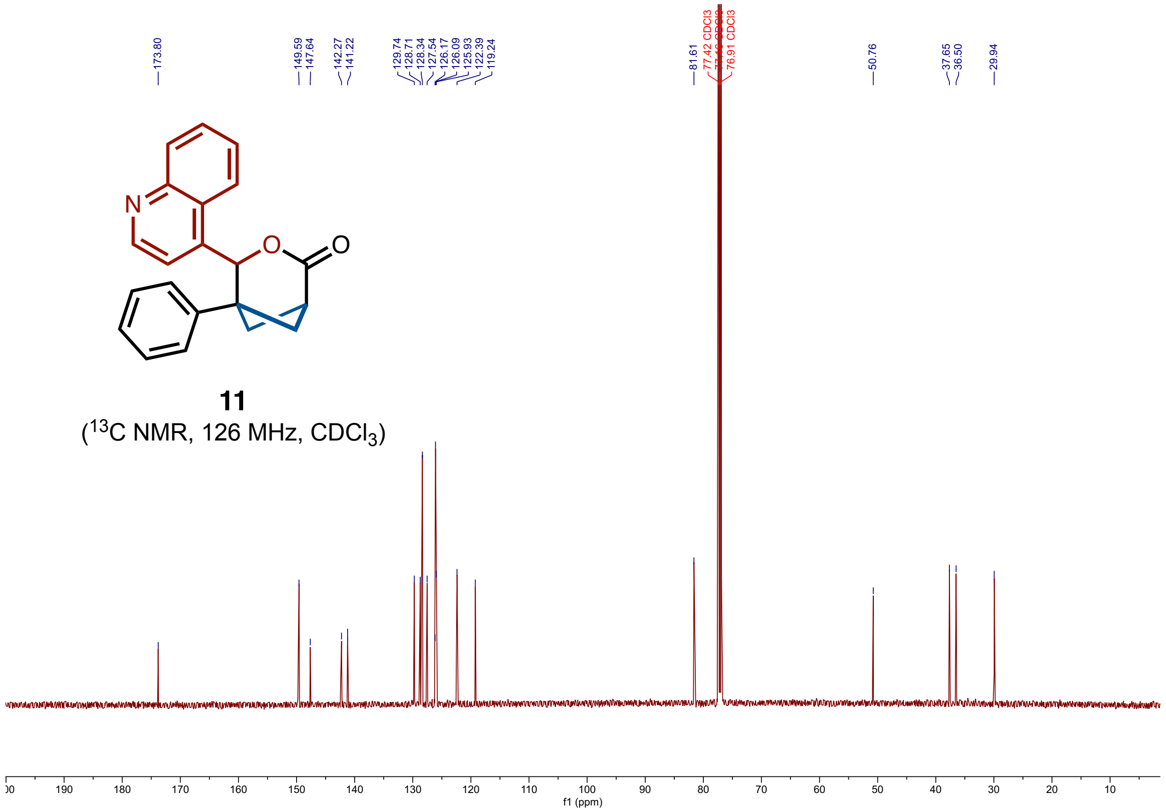
173.80
149.59
147.64
142.27
141.22
129.74
128.71
128.34
127.54
126.17
126.09
125.93
122.39
119.24
81.61
77.42 CDCl3
77.16 CDCl3
76.91 CDCl3
50.76
37.65
36.50
29.94
N
O
O
11
(¹³C NMR, 126 MHz, CDCl₃)
190
180
170
160
150
140
130
120
110
100
90
80
70
60
50
40
30
20
10
f1 (ppm)

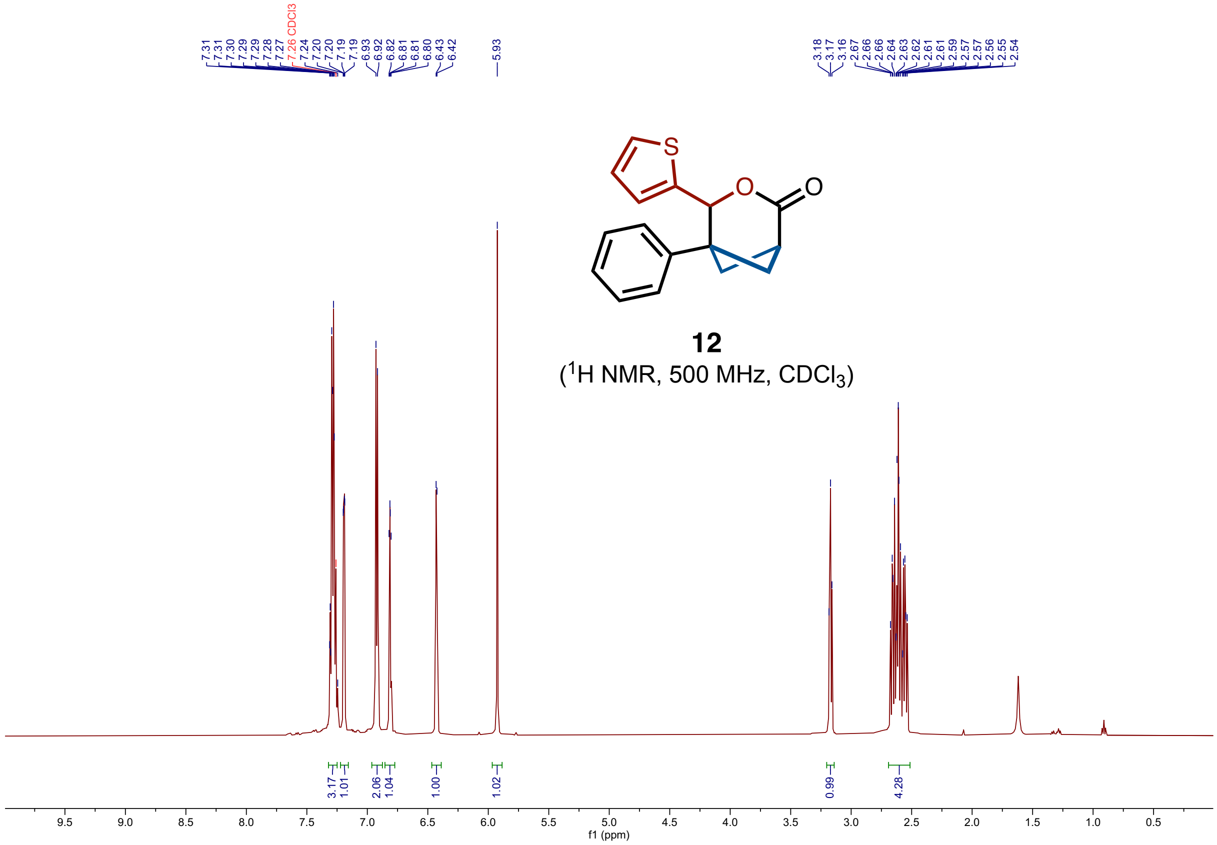
7.31
7.31
7.30
7.29
7.29
7.28
7.27
7.26 CDCl3
7.24
7.20
7.20
7.19
7.19
6.93
6.92
6.82
6.81
6.81
6.80
6.43
6.42
5.93
3.18
3.17
3.16
2.67
2.66
2.66
2.64
2.63
2.62
2.61
2.61
2.59
2.57
2.57
2.56
2.55
2.54
S
O
O
12
(1H NMR, 500 MHz, CDCl3)
3.17
1.01
2.06
1.04
1.00
1.02
0.99
4.28
f1 (ppm)

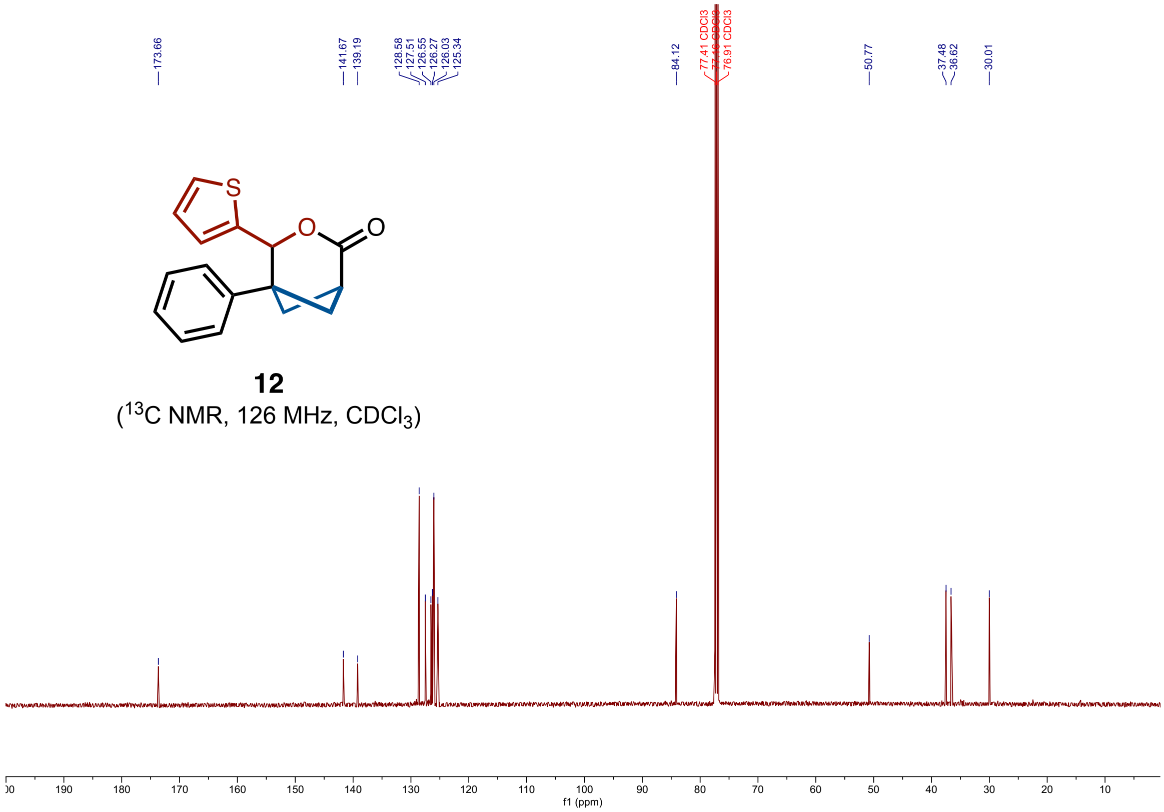
173.66
141.67
139.19
128.58
127.51
126.55
126.27
126.03
125.34
84.12
77.41 CDCl3
77.16 CDCl3
76.91 CDCl3
50.77
37.48
36.62
30.01
S
O
O
12
(13C NMR, 126 MHz, CDCl3)
f1 (ppm)

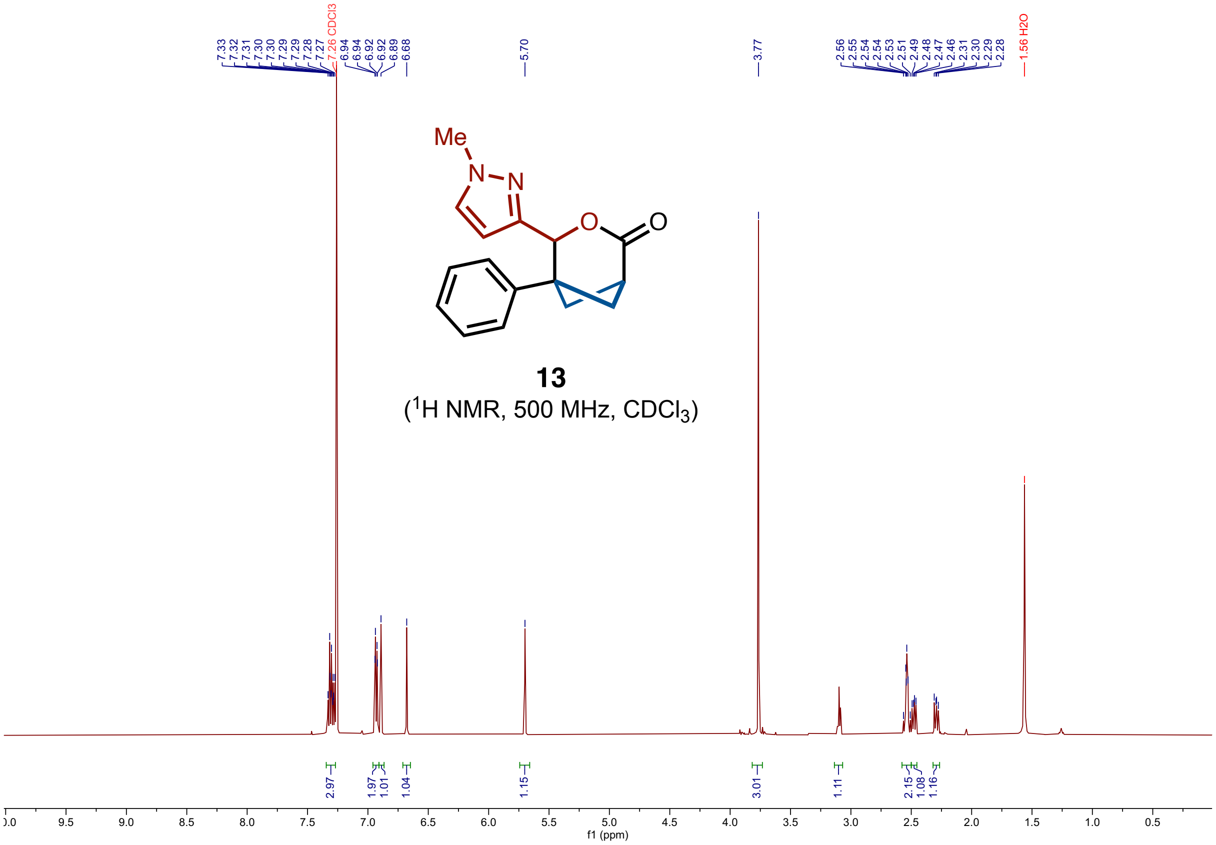
7.33 7.32 7.31 7.30 7.30 7.29 7.29 7.28 7.27
7.26 CDCl3
6.94 6.94 6.92 6.92 6.89 6.68
5.70
3.77
2.56 2.55 2.54 2.54 2.53 2.51 2.49 2.48 2.47 2.46 2.31 2.30 2.29 2.28
1.56 H2O
Me
N
N
O
O
13
(1H NMR, 500 MHz, CDCl3)
2.97 1.97 1.01 1.04 1.15 3.01 1.11 2.15 1.08 1.16
f1 (ppm)

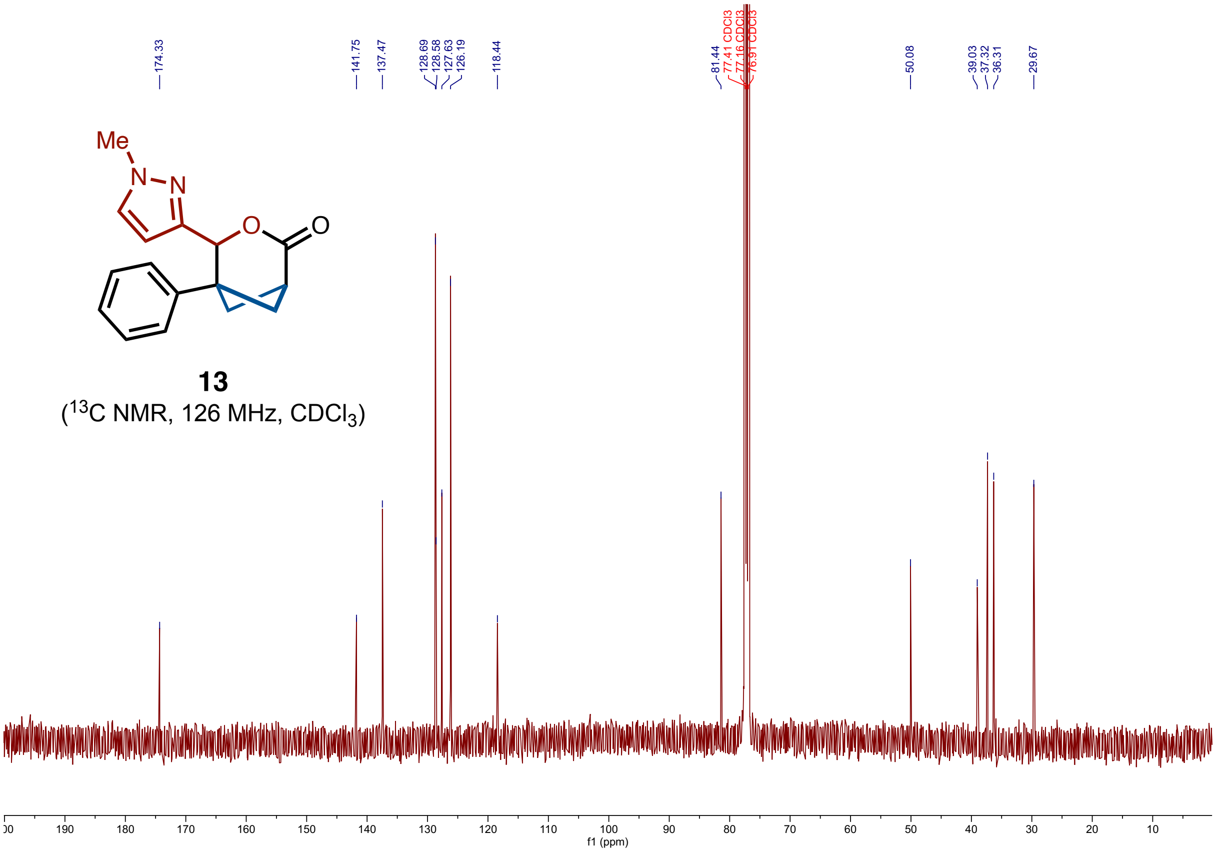
174.33
141.75
137.47
128.69 128.58 127.63 126.19
118.44
81.44
77.41 CDCl3
77.16 CDCl3
76.91 CDCl3
50.08
39.03 37.32 36.31
29.67
Me
N
N
O
O
13
(13C NMR, 126 MHz, CDCl3)
f1 (ppm)

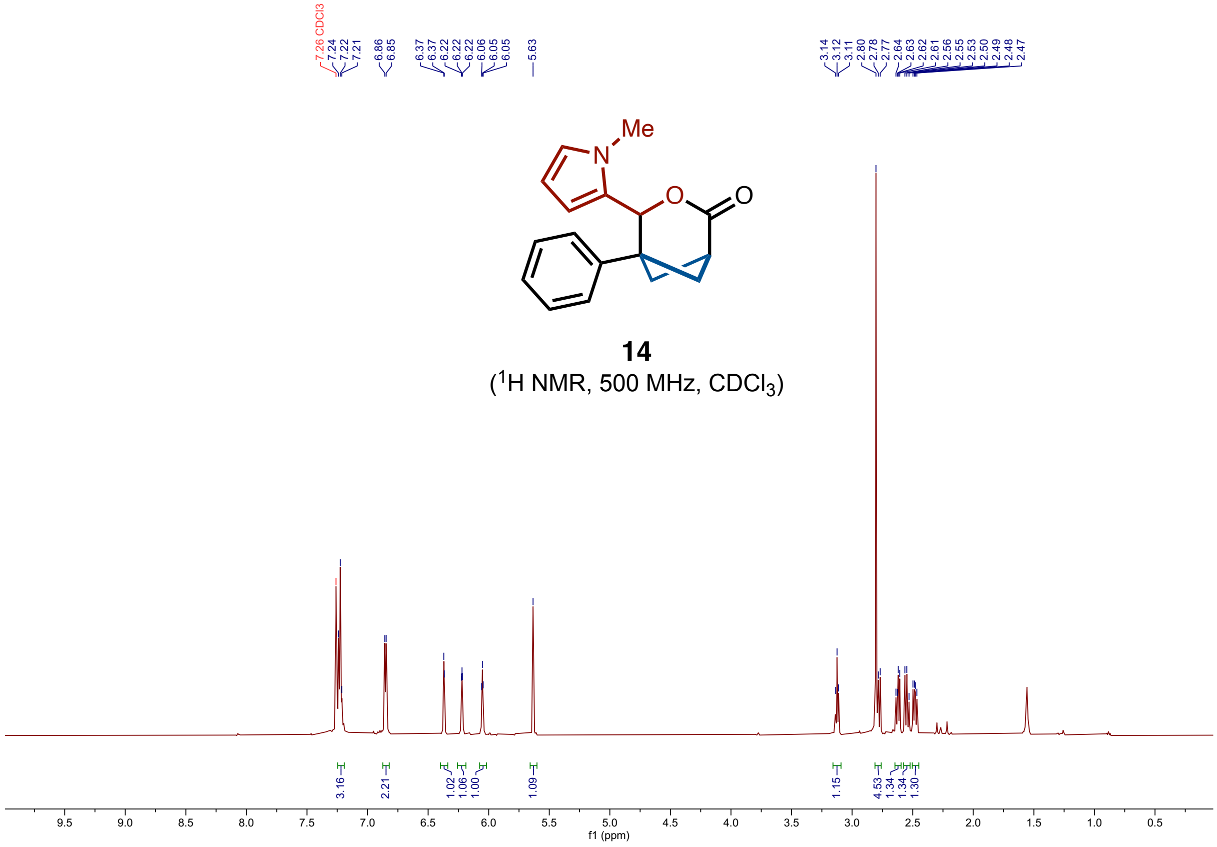
7.26 CDCl3
7.24
7.22
7.21
6.86
6.85
6.37
6.37
6.22
6.22
6.22
6.06
6.05
6.05
5.63
3.14
3.12
3.11
2.80
2.78
2.77
2.64
2.63
2.62
2.61
2.56
2.55
2.53
2.50
2.49
2.48
2.47
Me
N
O
O
14
(1H NMR, 500 MHz, CDCl3)
3.16
2.21
1.02
1.06
1.00
1.09
1.15
4.53
1.34
1.34
1.30
9.5 9.0 8.5 8.0 7.5 7.0 6.5 6.0 5.5 5.0 4.5 4.0 3.5 3.0 2.5 2.0 1.5 1.0 0.5
f1 (ppm)

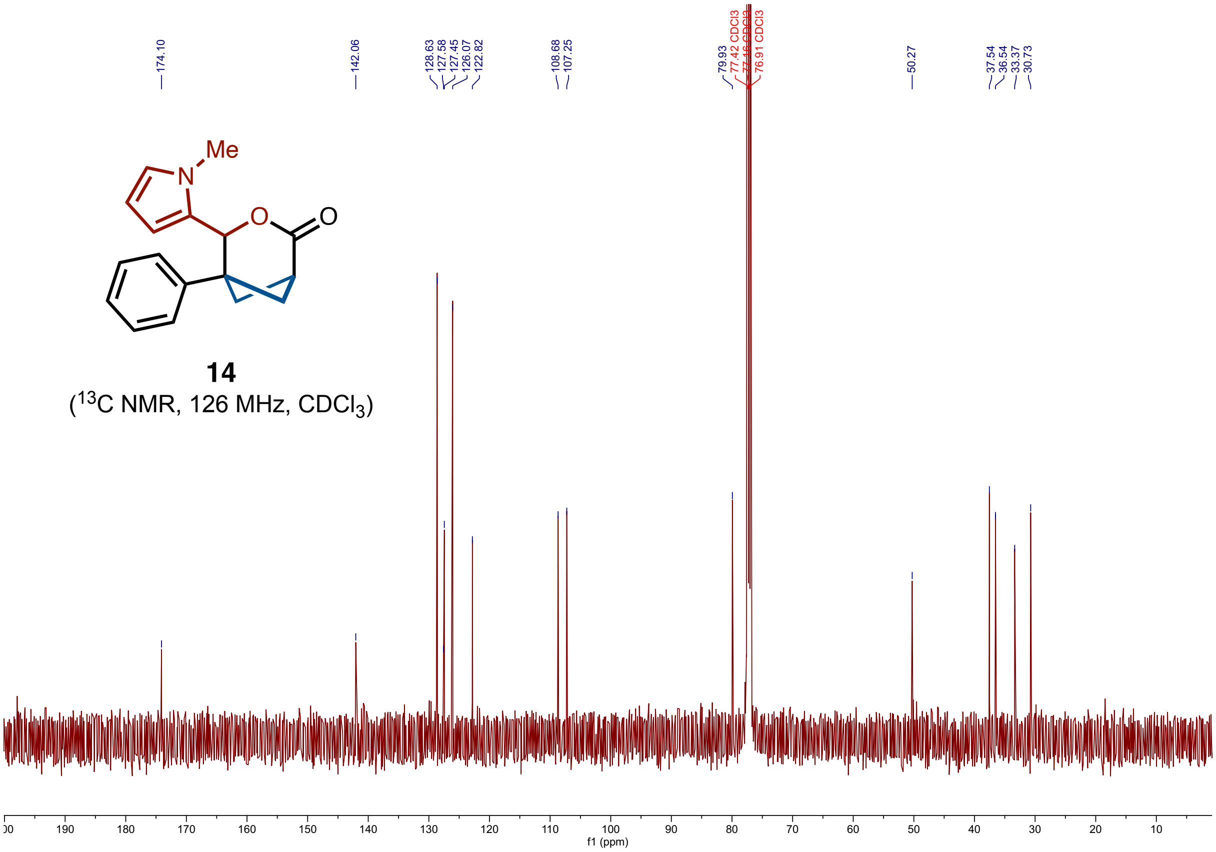
174.10
142.06
128.63
127.58
127.45
126.07
122.82
108.68
107.25
79.93
77.42 CDCl3
77.16 CDCl3
76.91 CDCl3
50.27
37.54
36.54
33.37
30.73
Me
N
O
O
14
(13C NMR, 126 MHz, CDCl3)
190 180 170 160 150 140 130 120 110 100 90 80 70 60 50 40 30 20 10
f1 (ppm)

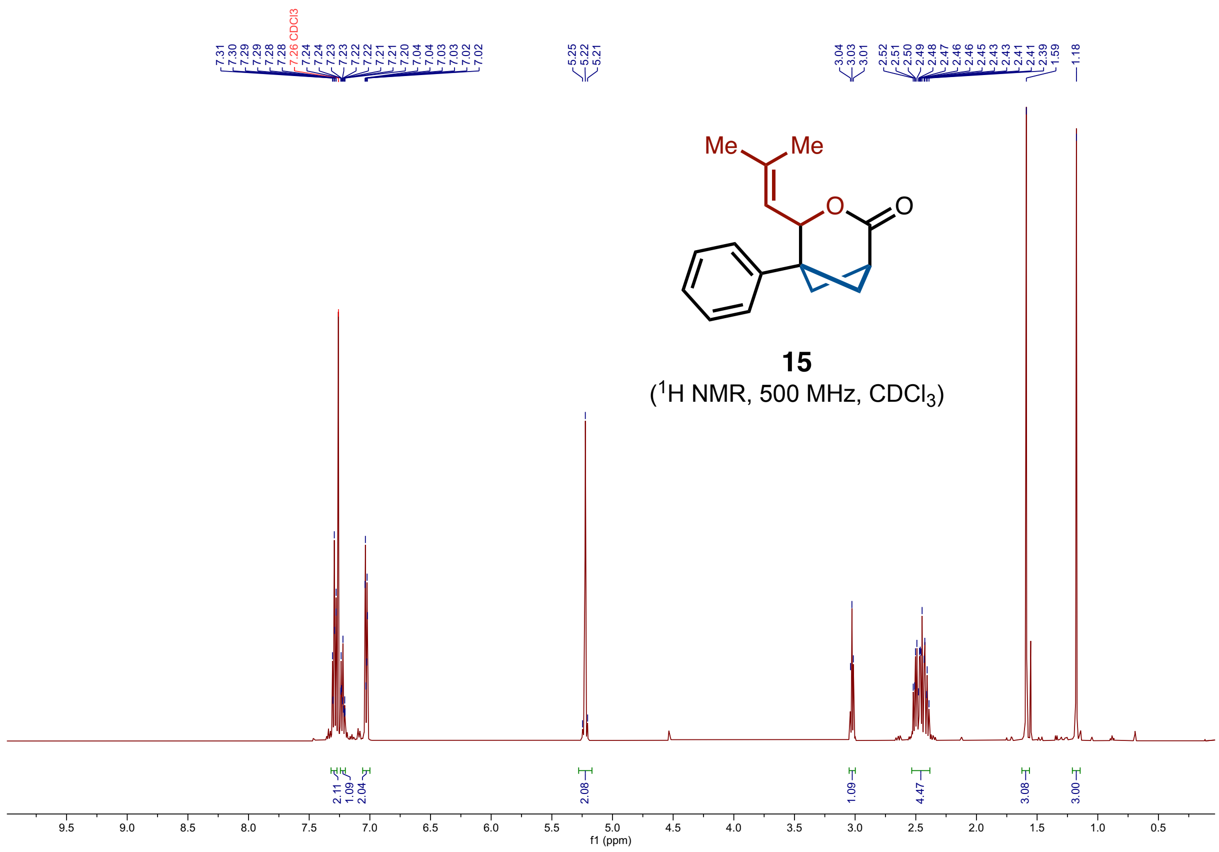
7.31
7.30
7.29
7.29
7.28
7.26 CDCl3
7.24
7.24
7.23
7.23
7.22
7.22
7.21
7.21
7.20
7.04
7.04
7.03
7.03
7.02
7.02
5.25
5.22
5.21
3.04
3.03
3.01
2.52
2.51
2.50
2.49
2.48
2.47
2.46
2.46
2.45
2.43
2.43
2.41
2.41
2.39
1.59
1.18
Me
Me
O
O
15
(1H NMR, 500 MHz, CDCl3)
2.11
1.09
2.04
2.08
1.09
4.47
3.08
3.00
f1 (ppm)

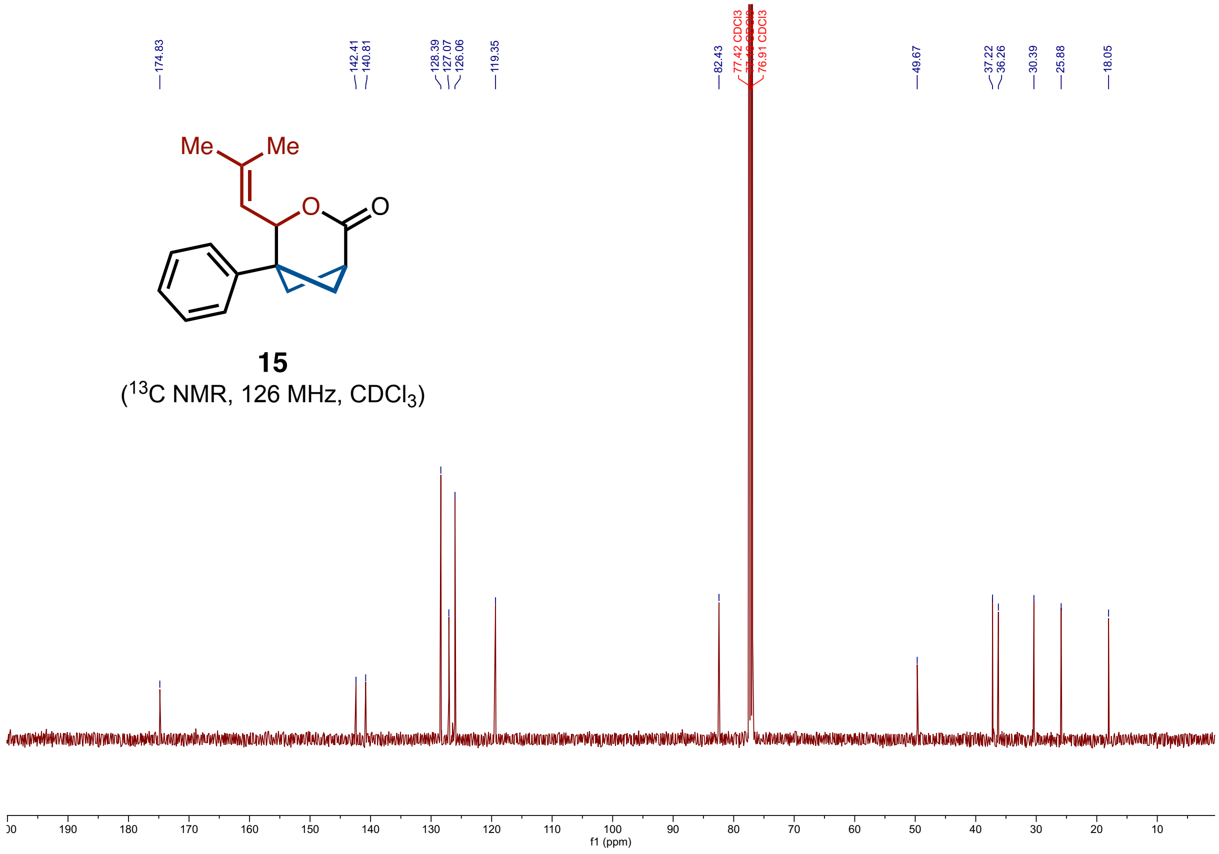
174.83
142.41
140.81
128.39
127.07
126.06
119.35
82.43
77.42 CDCl3
76.91 CDCl3
49.67
37.22
36.26
30.39
25.88
18.05
Me
Me
O
O
15
(13C NMR, 126 MHz, CDCl3)
f1 (ppm)

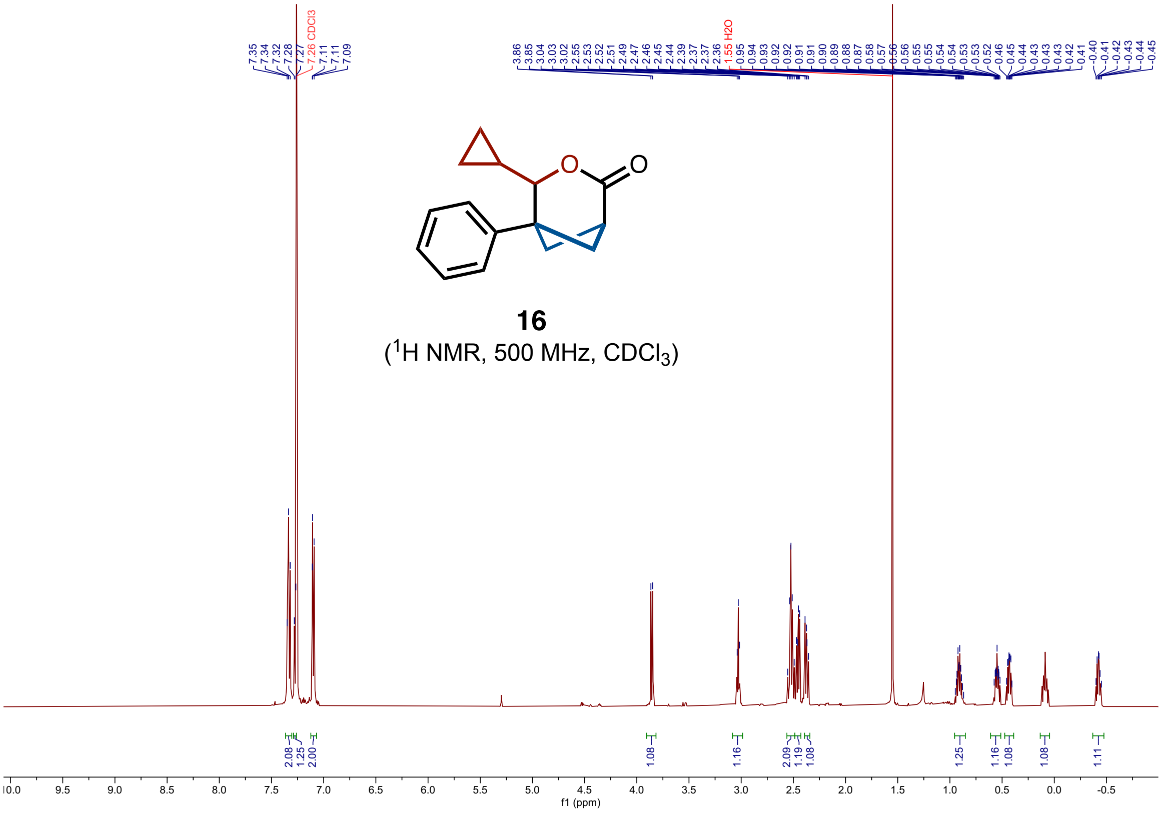
7.35 7.34 7.32 7.28 7.27 7.26 CDCl3 7.11 7.11 7.09
3.86 3.85 3.04 3.03 3.02 2.55 2.53 2.52 2.51 2.49 2.47 2.46 2.45 2.44 2.39 2.37 2.37 2.36 1.55 H2O 0.95 0.94 0.93 0.92 0.92 0.91 0.91 0.90 0.89 0.88 0.87 0.58 0.57 0.56 0.56 0.55 0.55 0.54 0.54 0.53 0.53 0.53 0.52 0.46 0.45 0.44 0.43 0.43 0.43 0.42 0.41 -0.40 -0.41 -0.42 -0.43 -0.44 -0.45
O
O
16
(1H NMR, 500 MHz, CDCl3)
2.08 1.25 2.00 1.08 1.16 2.09 1.19 1.08 1.25 1.16 1.08 1.08 1.11
f1 (ppm)

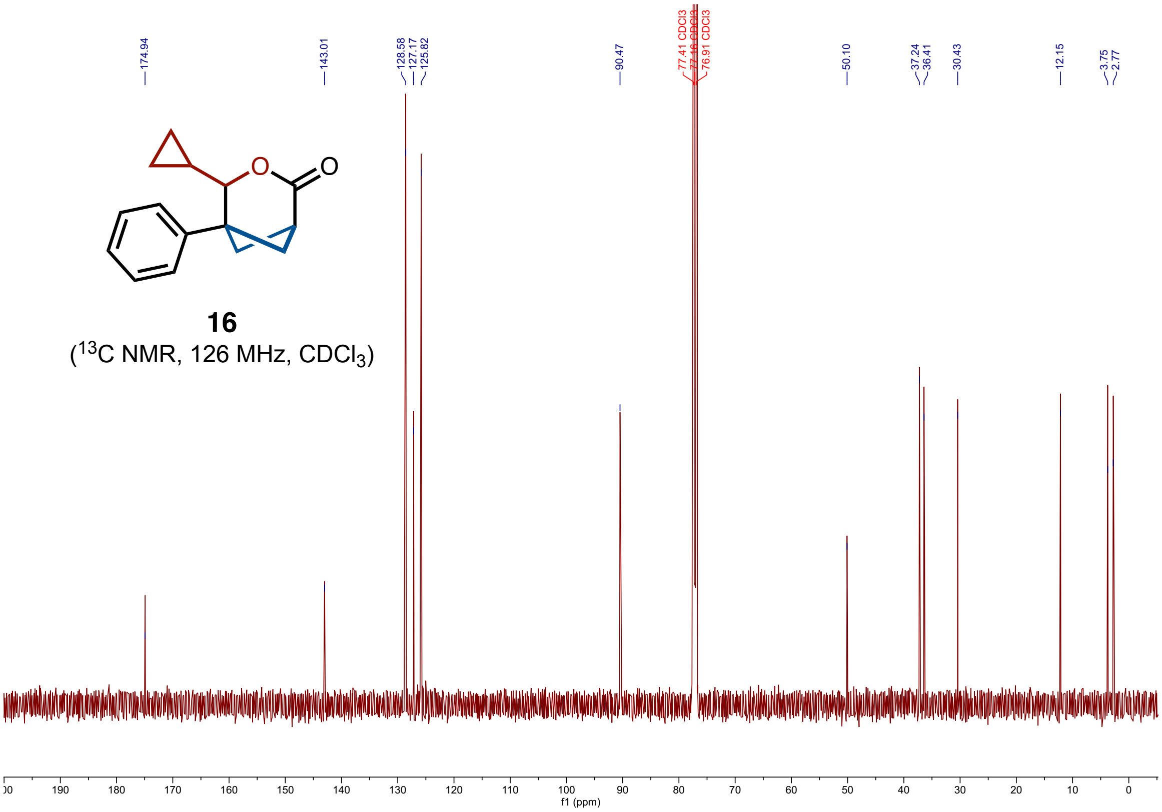
174.94
143.01
128.58 127.17 125.82
90.47
77.41 CDCl3 77.16 CDCl3 76.91 CDCl3
50.10
37.24 36.41
30.43
12.15
3.75 2.77
O
O
16
(13C NMR, 126 MHz, CDCl3)
f1 (ppm)

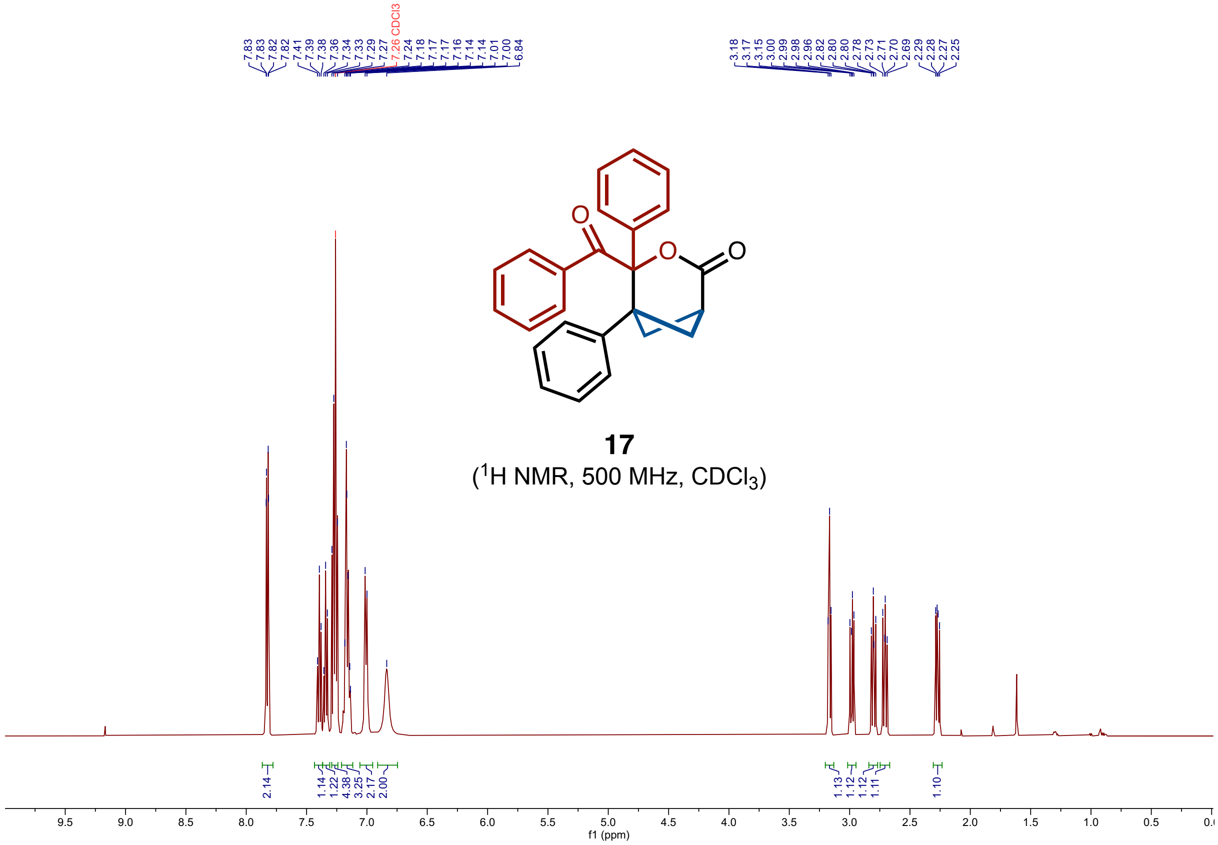
7.83
7.83
7.82
7.41
7.39
7.38
7.36
7.34
7.33
7.29
7.27
7.26 CDCl3
7.24
7.18
7.17
7.17
7.16
7.14
7.14
7.01
7.00
6.84
3.18
3.17
3.15
3.00
2.99
2.98
2.96
2.82
2.80
2.80
2.78
2.73
2.71
2.70
2.69
2.29
2.28
2.27
2.25
17
(1H NMR, 500 MHz, CDCl3)
2.14
1.14
1.22
4.38
3.25
2.17
2.00
1.13
1.12
1.12
1.11
1.10
f1 (ppm)

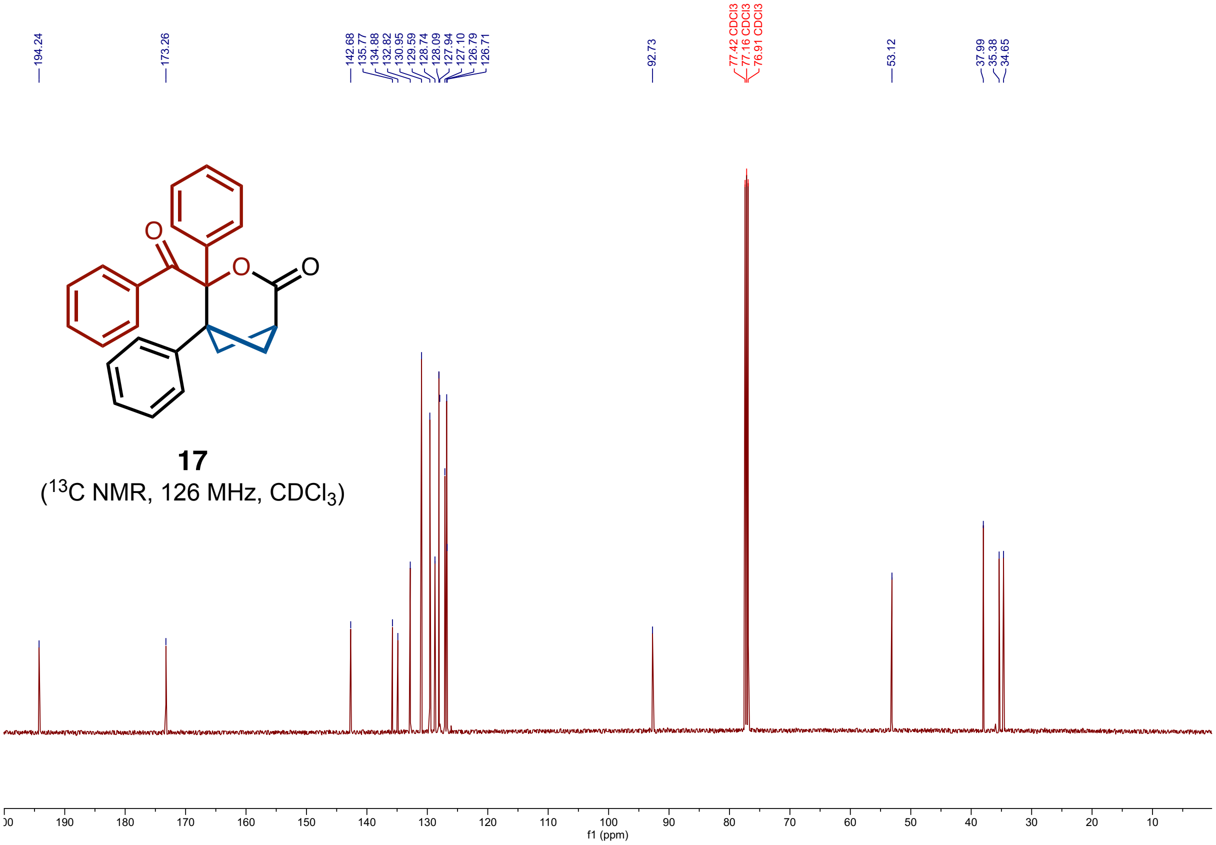
194.24
173.26
142.68
135.77
134.88
132.82
130.95
129.59
128.74
128.09
127.94
127.10
126.79
126.71
92.73
77.42 CDCl3
77.16 CDCl3
76.91 CDCl3
53.12
37.99
35.38
34.65
17
(13C NMR, 126 MHz, CDCl3)
f1 (ppm)

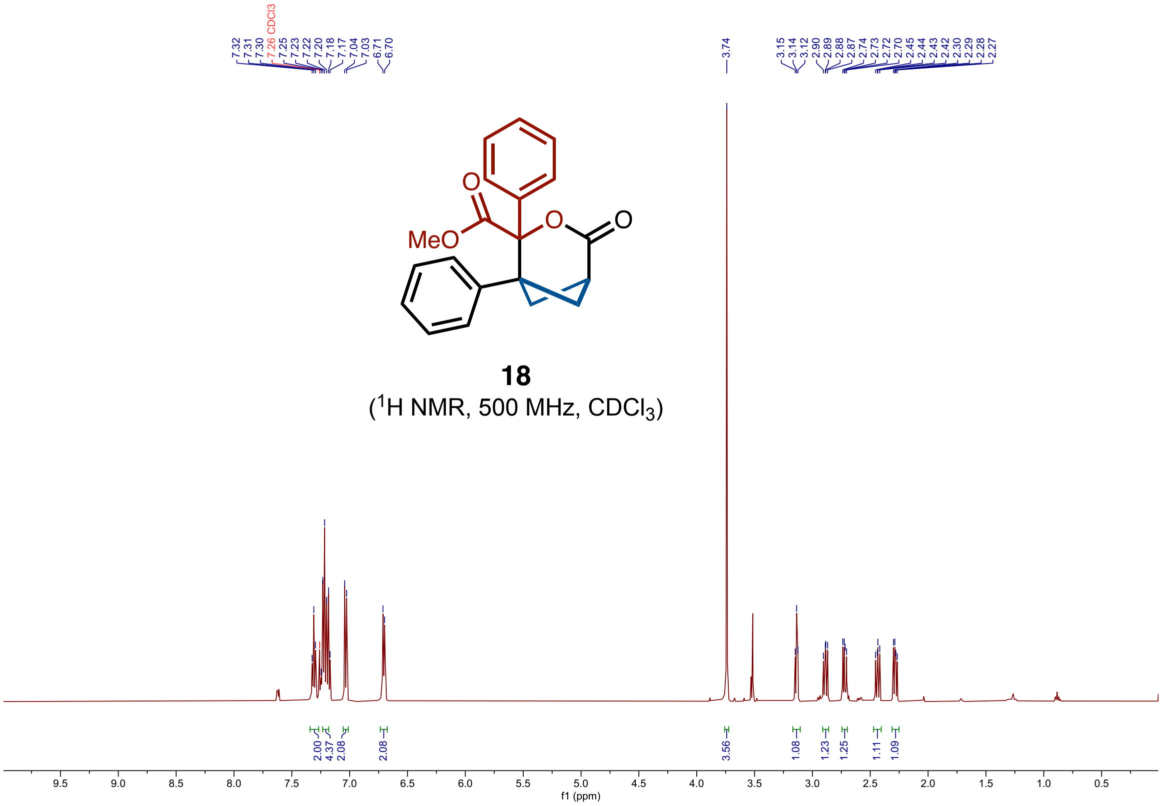

18

(^{1}H NMR, 500 MHz, CDCl$_3$)

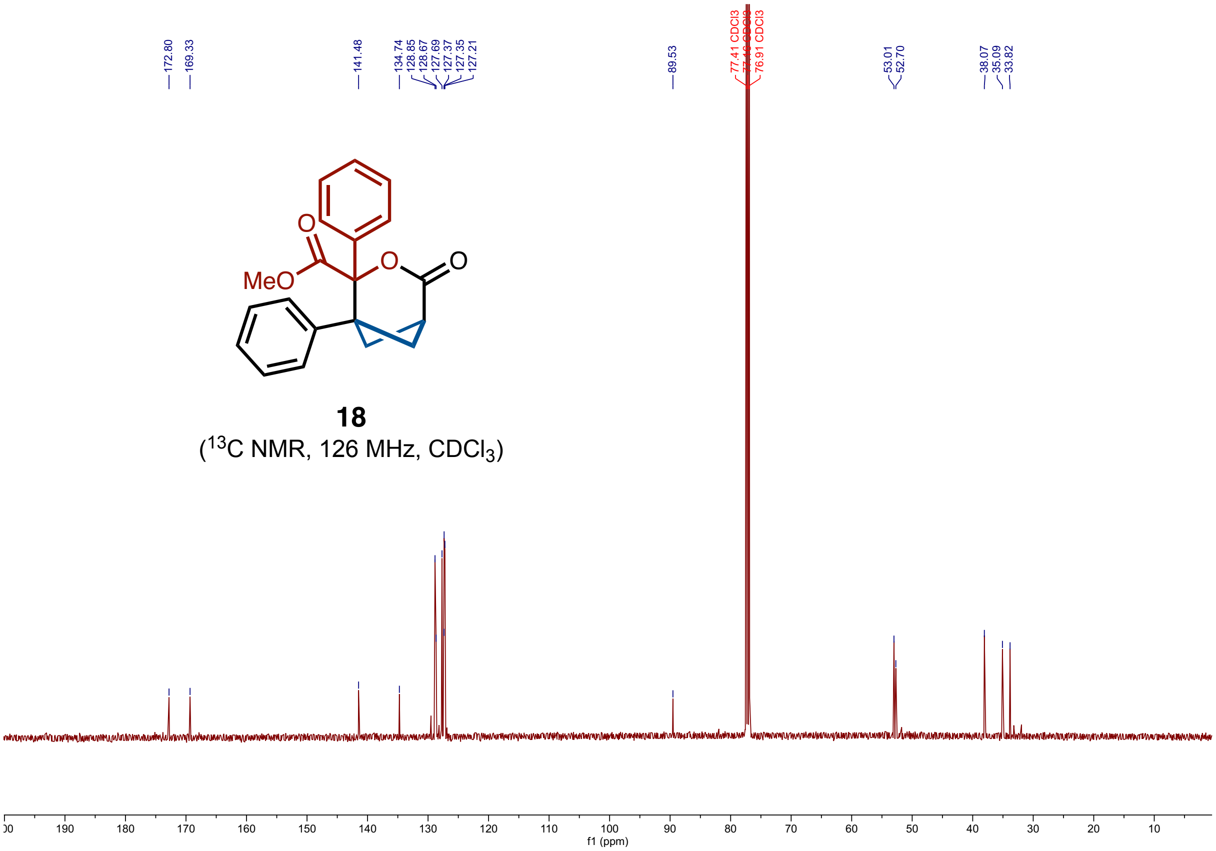

18

(^{13}C NMR, 126 MHz, CDCl$_3$)

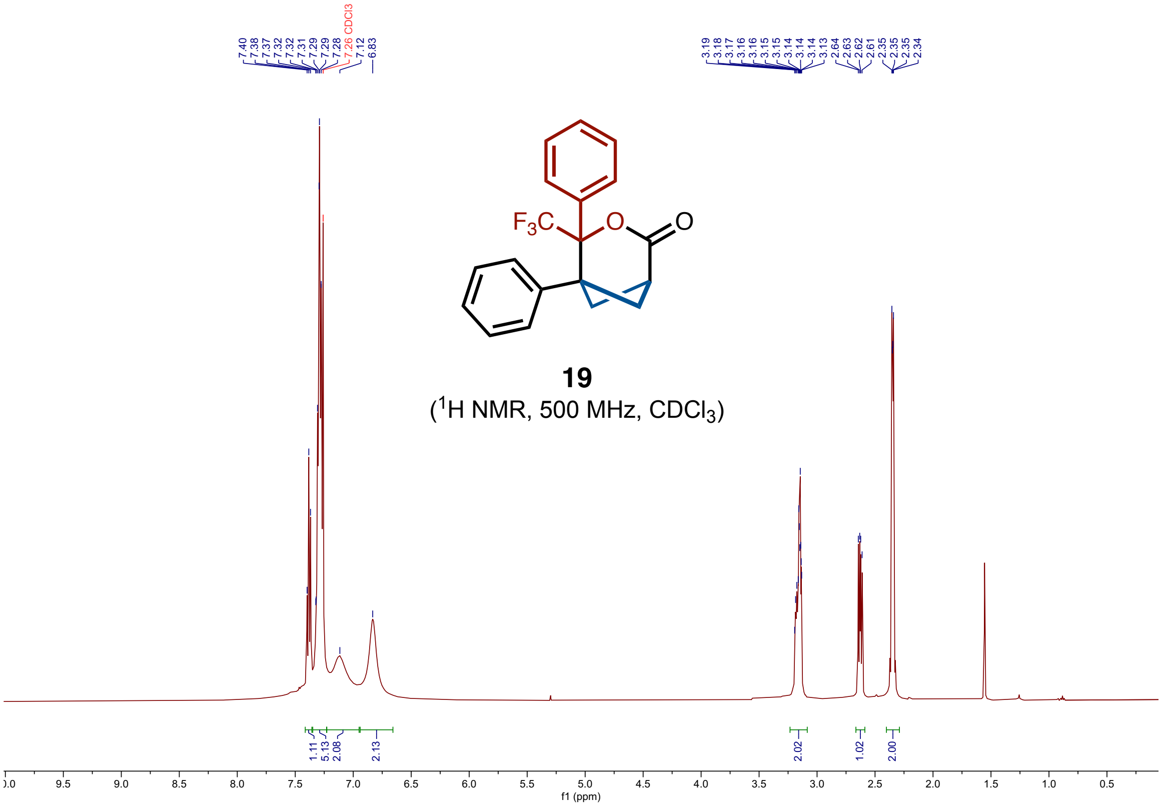
7.40
7.38
7.37
7.32
7.32
7.31
7.29
7.28
7.26 CDCl3
7.12
6.83
3.19
3.18
3.17
3.16
3.16
3.15
3.15
3.14
3.14
3.13
2.64
2.63
2.62
2.61
2.35
2.35
2.34
F3C
O
O
19
(1H NMR, 500 MHz, CDCl3)
1.11
5.13
2.08
2.13
2.02
1.02
2.00
f1 (ppm)

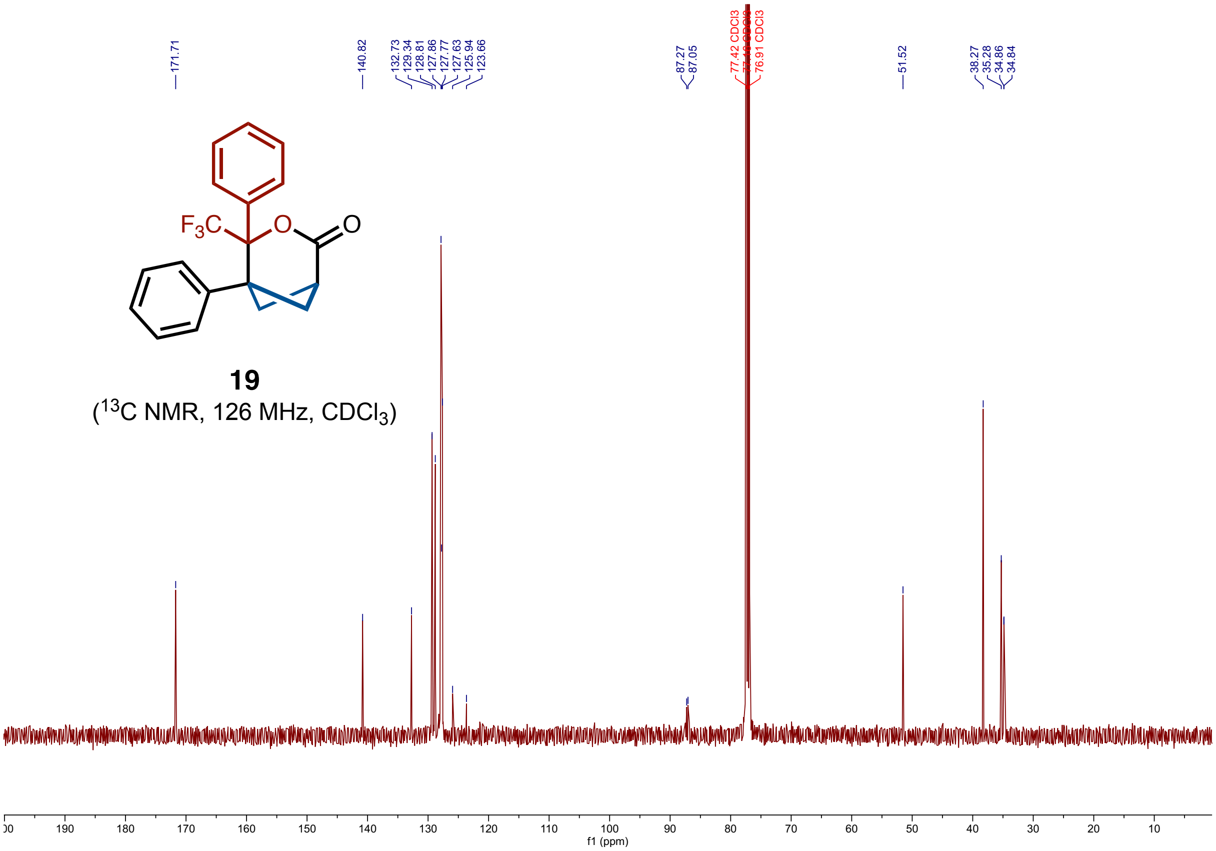
171.71
140.82
132.73
129.34
128.81
127.86
127.77
127.63
125.94
123.66
87.27
87.05
77.42 CDCl3
76.91 CDCl3
51.52
38.27
35.28
34.86
34.84
F3C
O
O
19
(13C NMR, 126 MHz, CDCl3)
f1 (ppm)

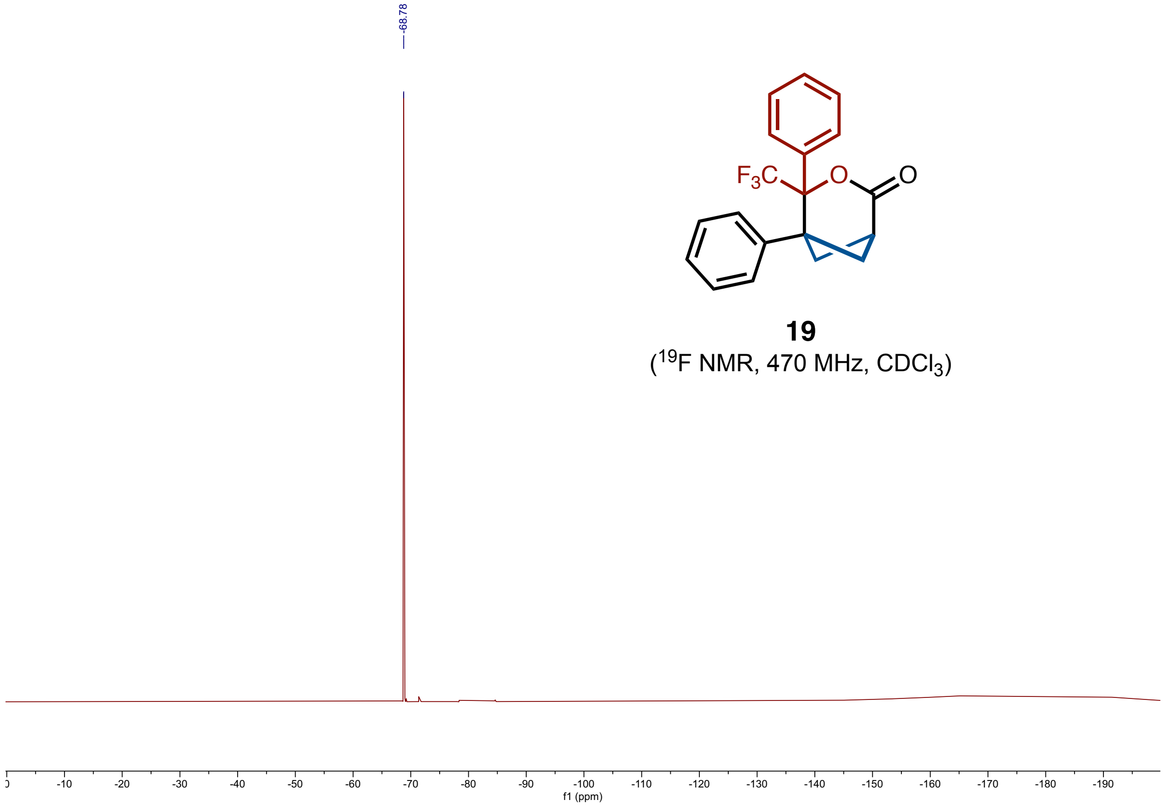

19

(^{19}F NMR, 470 MHz, $CDCl_3$)

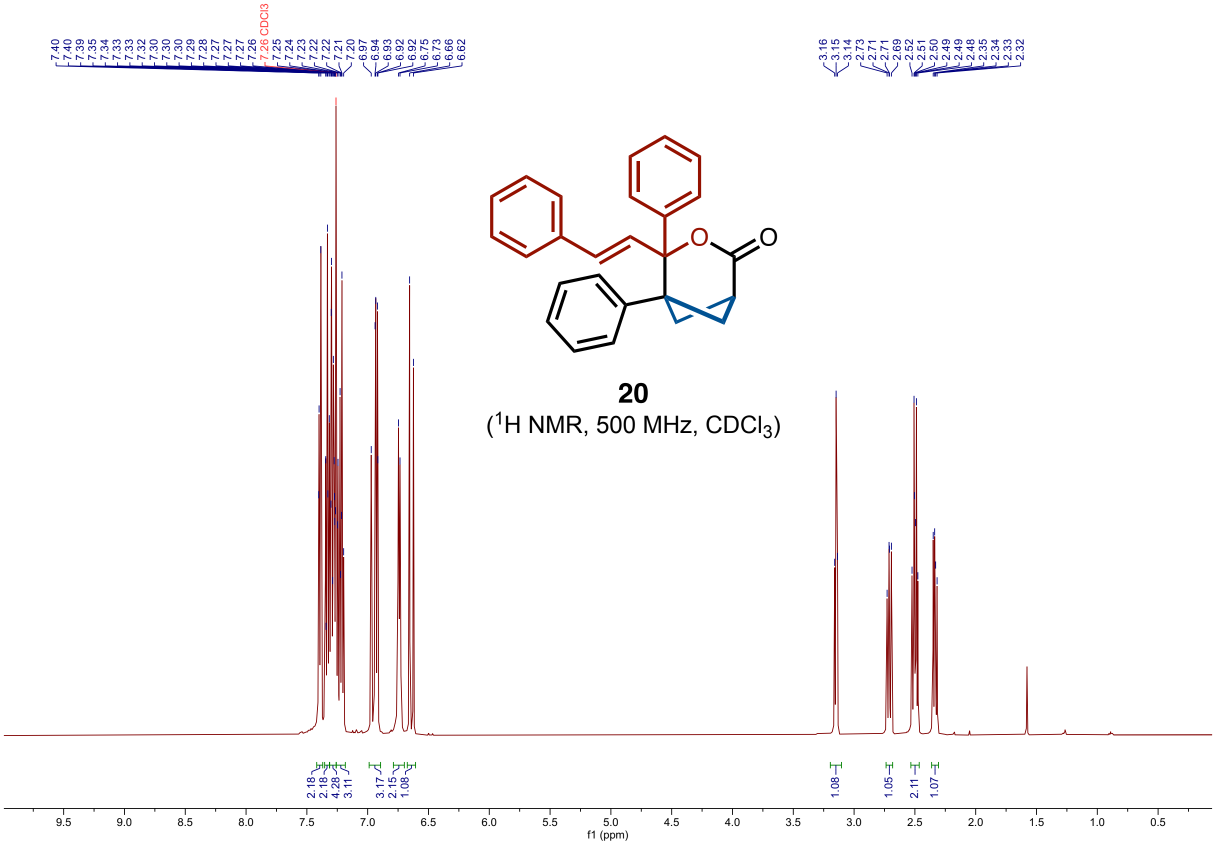
7.40 7.40 7.39 7.35 7.34 7.33 7.33 7.32 7.30 7.30 7.29 7.28 7.27 7.27 7.26 7.26 CDCl3 7.25 7.24 7.23 7.22 7.22 7.21 7.20 6.97 6.94 6.93 6.92 6.92 6.75 6.73 6.66 6.62
3.16 3.15 3.14 2.73 2.71 2.71 2.69 2.52 2.51 2.50 2.49 2.48 2.35 2.34 2.33 2.32
O
O
20
(^{1}H NMR, 500 MHz, $CDCl_3$)
2.18 2.18 4.28 3.11 3.17 2.15 1.08 1.08 1.05 2.11 1.07
f1 (ppm)

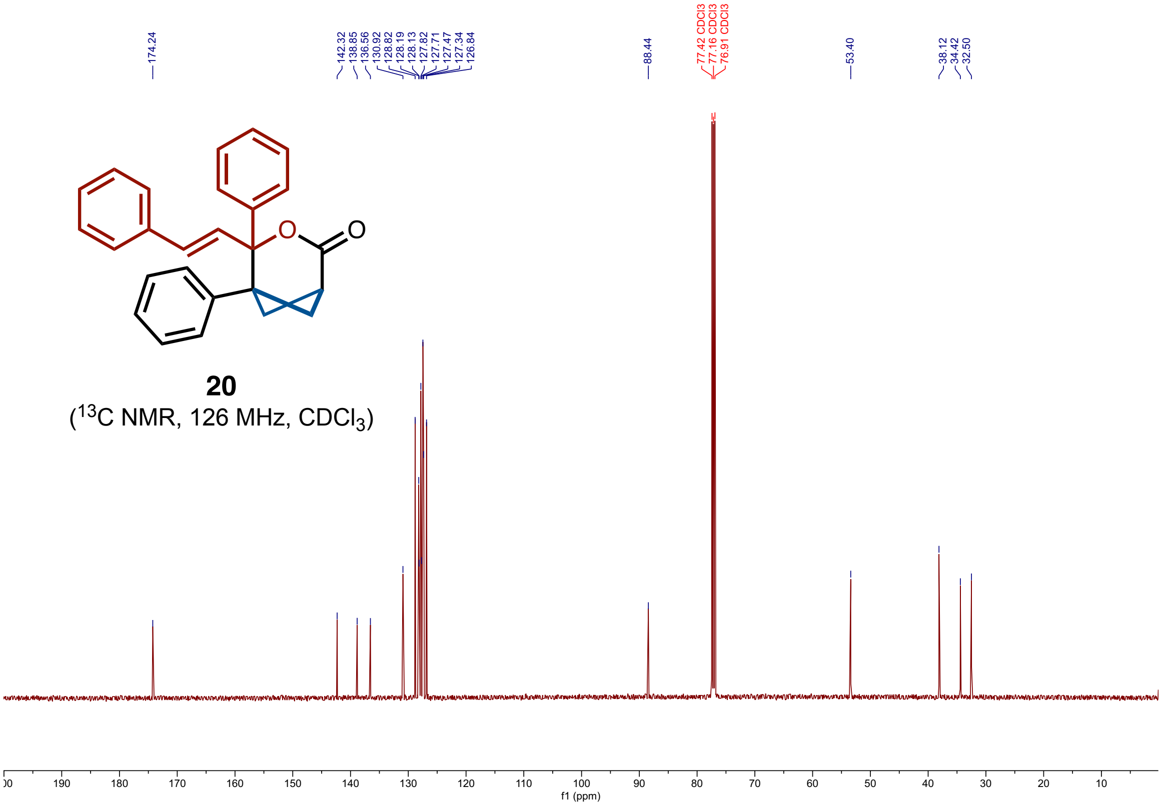
174.24
142.32 138.85 136.56 130.92 128.82 128.19 128.13 127.82 127.71 127.47 127.34 126.84
88.44
77.42 CDCl3 77.16 CDCl3 76.91 CDCl3
53.40
38.12 34.42 32.50
O
O
20
(^{13}C NMR, 126 MHz, $CDCl_3$)
f1 (ppm)

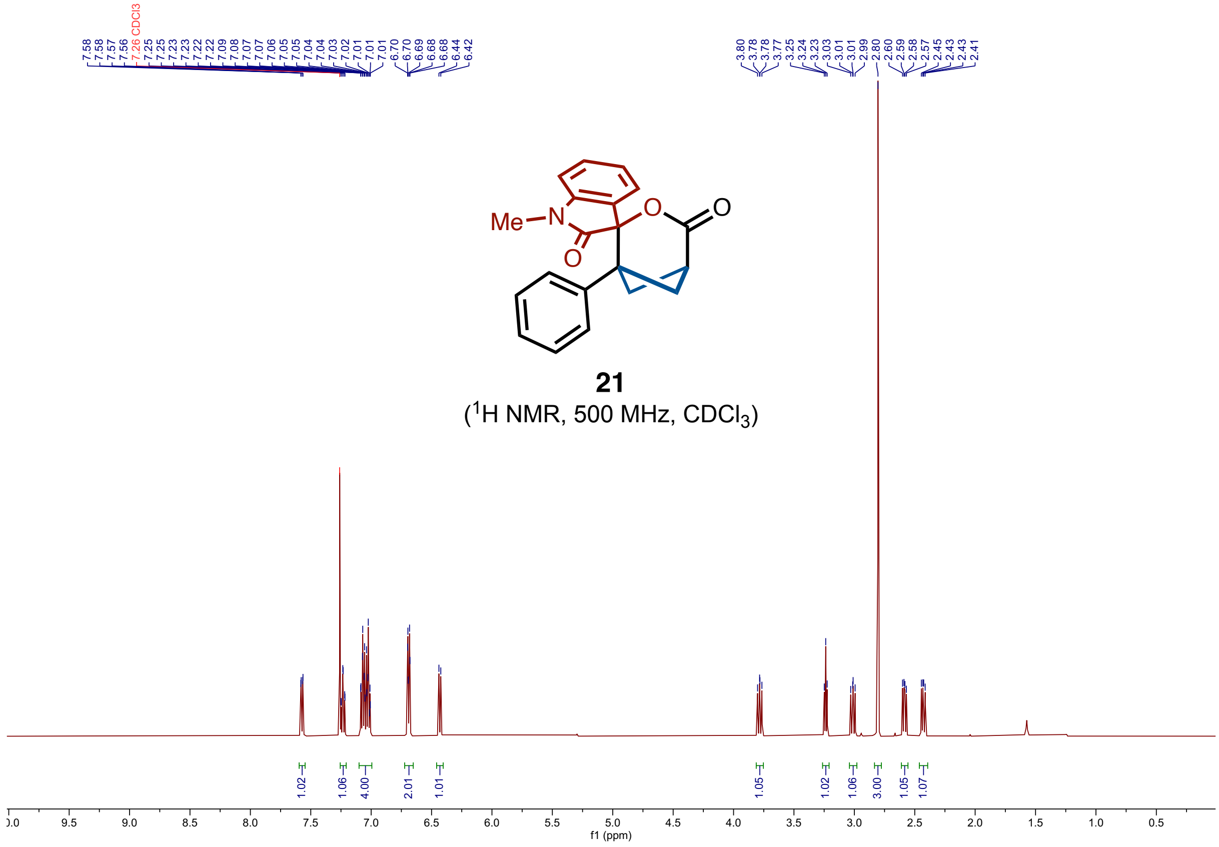

7.58
7.58
7.57
7.56
7.26 CDCl3
7.25
7.25
7.23
7.23
7.22
7.09
7.08
7.07
7.07
7.06
7.05
7.05
7.04
7.04
7.03
7.02
7.01
7.01
7.01
6.70
6.70
6.69
6.68
6.68
6.44
6.42
3.80
3.78
3.78
3.77
3.25
3.24
3.23
3.03
3.01
3.01
2.99
2.80
2.60
2.59
2.58
2.57
2.45
2.43
2.43
2.41
Me
N
O
O
O
21
(1H NMR, 500 MHz, CDCl3)
1.02
1.06
4.00
2.01
1.01
1.05
1.02
1.06
3.00
1.05
1.07
f1 (ppm)

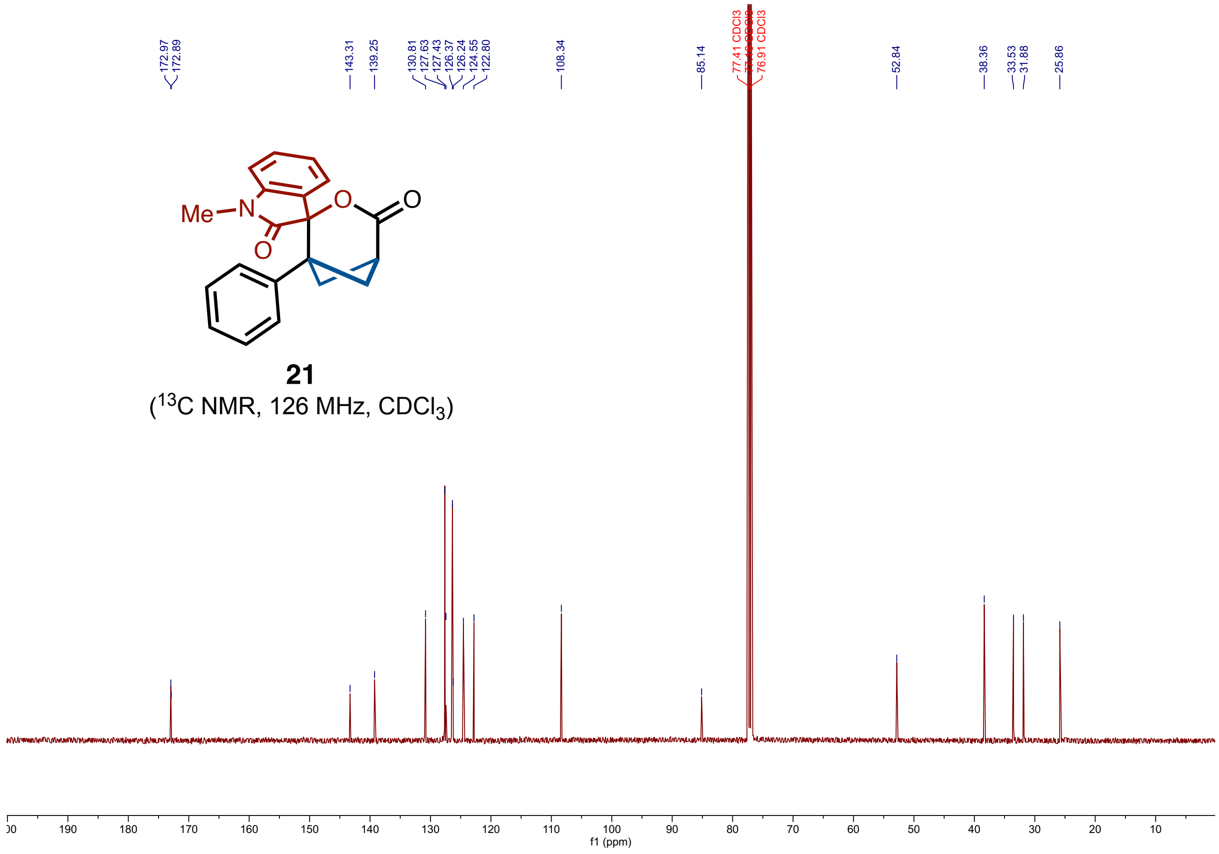

172.97
172.89
143.31
139.25
130.81
127.63
127.43
126.37
126.24
124.55
122.80
108.34
85.14
77.41 CDCl3
76.91 CDCl3
52.84
38.36
33.53
31.88
25.86
Me
N
O
O
O
21
(13C NMR, 126 MHz, CDCl3)
f1 (ppm)

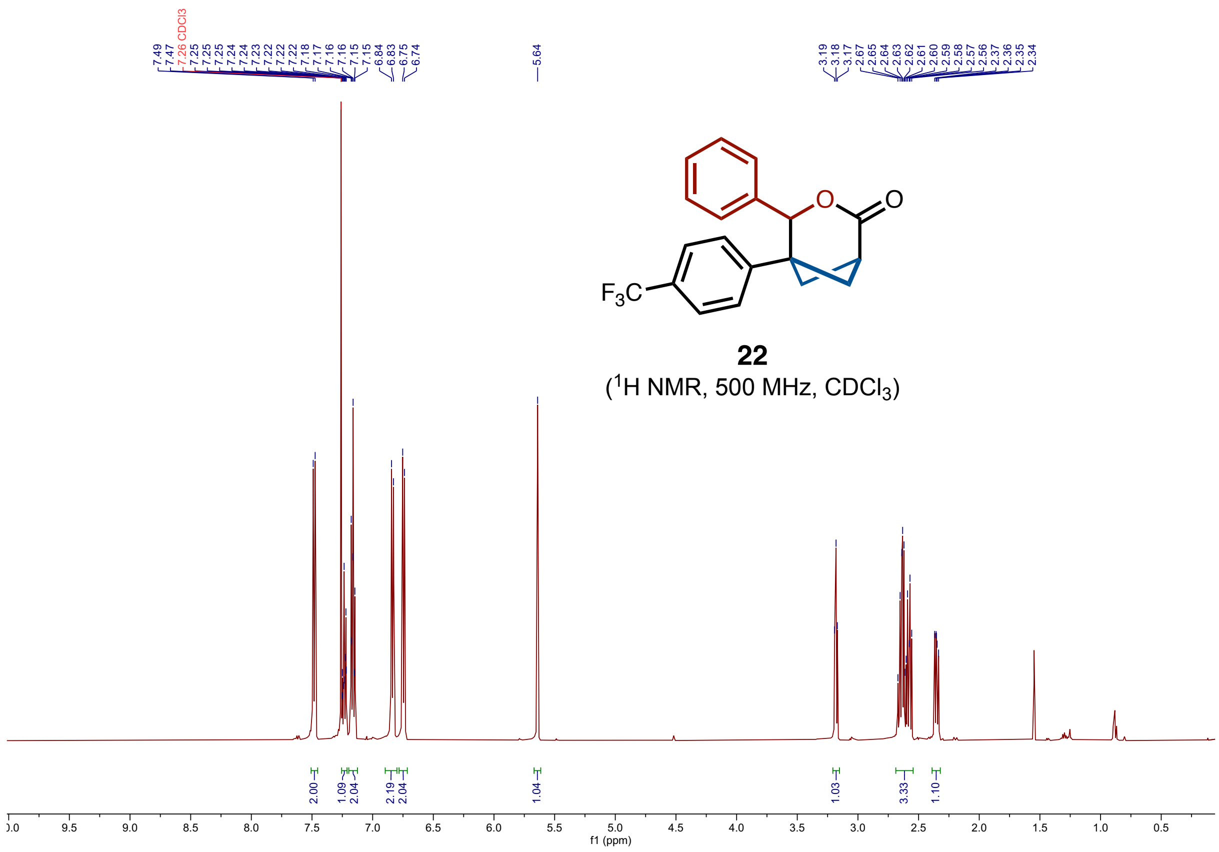
22
(¹H NMR, 500 MHz, CDCl3)
f1 (ppm)

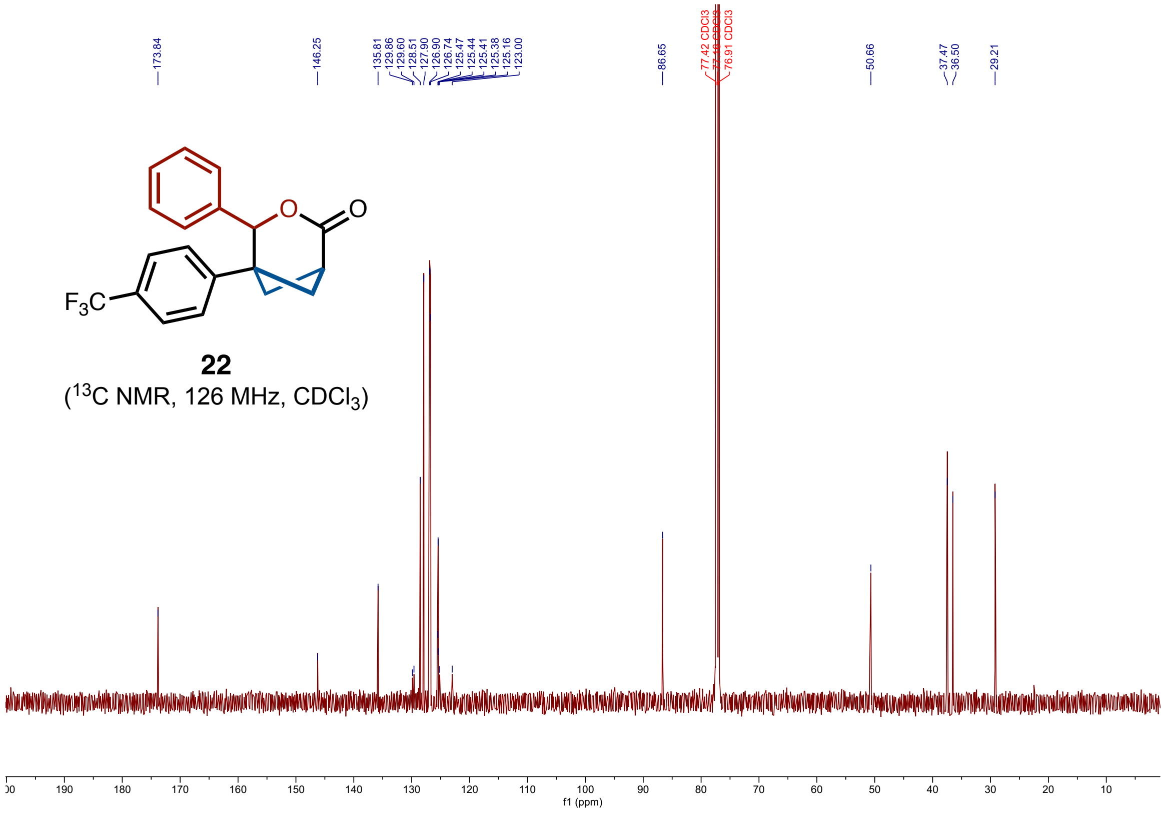
22
(¹³C NMR, 126 MHz, CDCl3)
f1 (ppm)

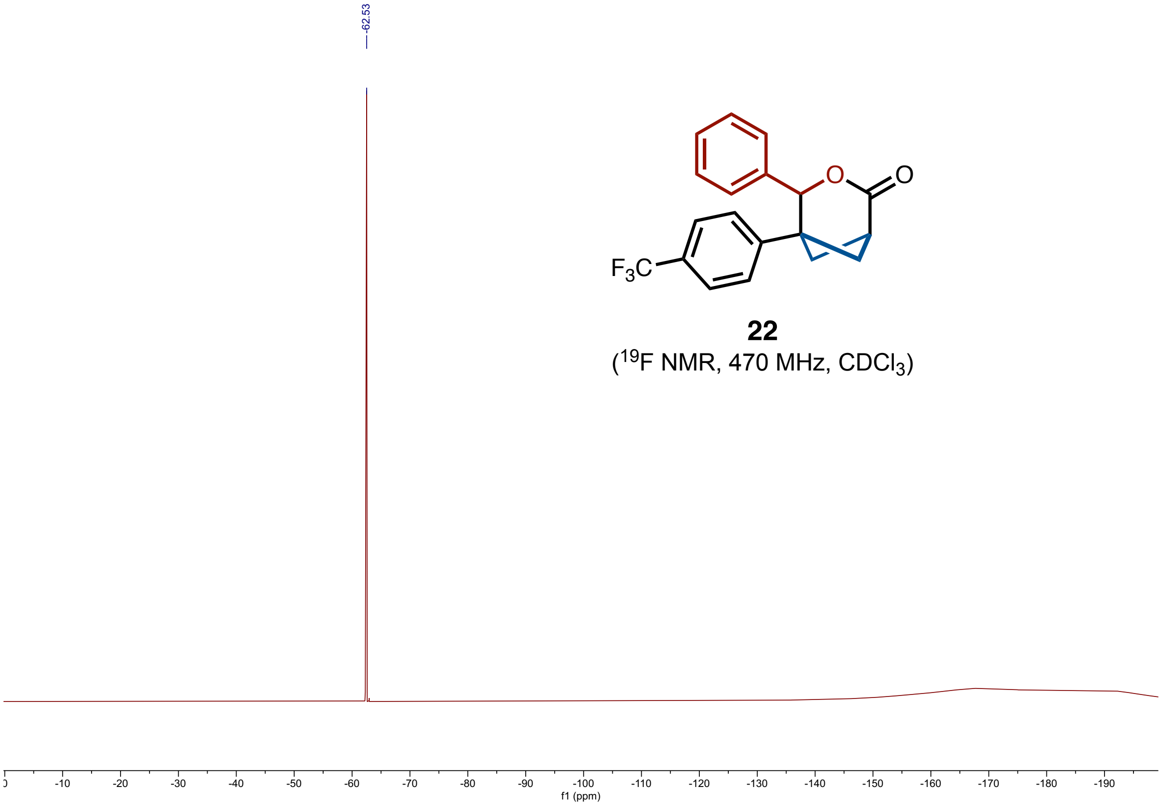

22

(^{19}F NMR, 470 MHz, $CDCl_3$)

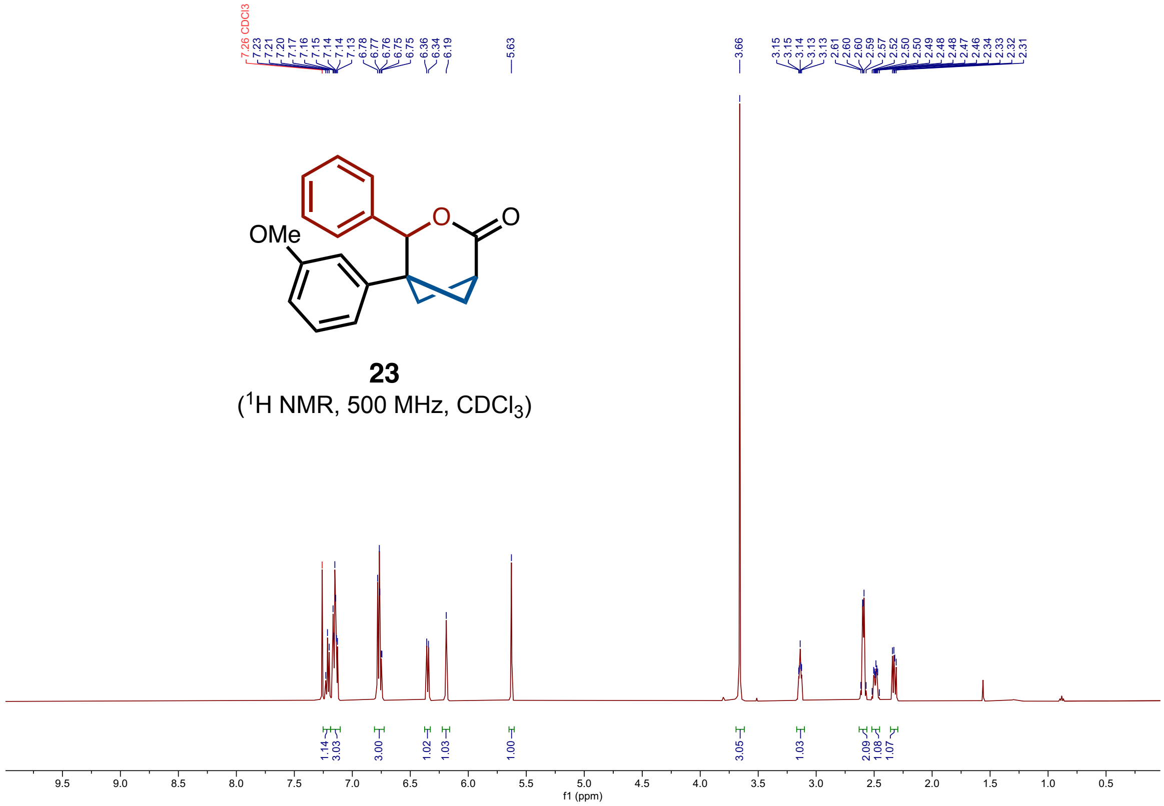

7.26 CDCl3
7.23
7.21
7.20
7.17
7.16
7.15
7.14
7.14
7.13
6.78
6.77
6.76
6.75
6.75
6.36
6.34
6.19
5.63
3.66
3.15
3.15
3.14
3.13
3.13
2.61
2.60
2.60
2.59
2.57
2.52
2.50
2.50
2.49
2.48
2.48
2.47
2.46
2.34
2.33
2.32
2.31
OMe
O
O
23
(1H NMR, 500 MHz, CDCl3)
1.14
3.03
3.00
1.02
1.03
1.00
3.05
1.03
2.09
1.08
1.07
f1 (ppm)

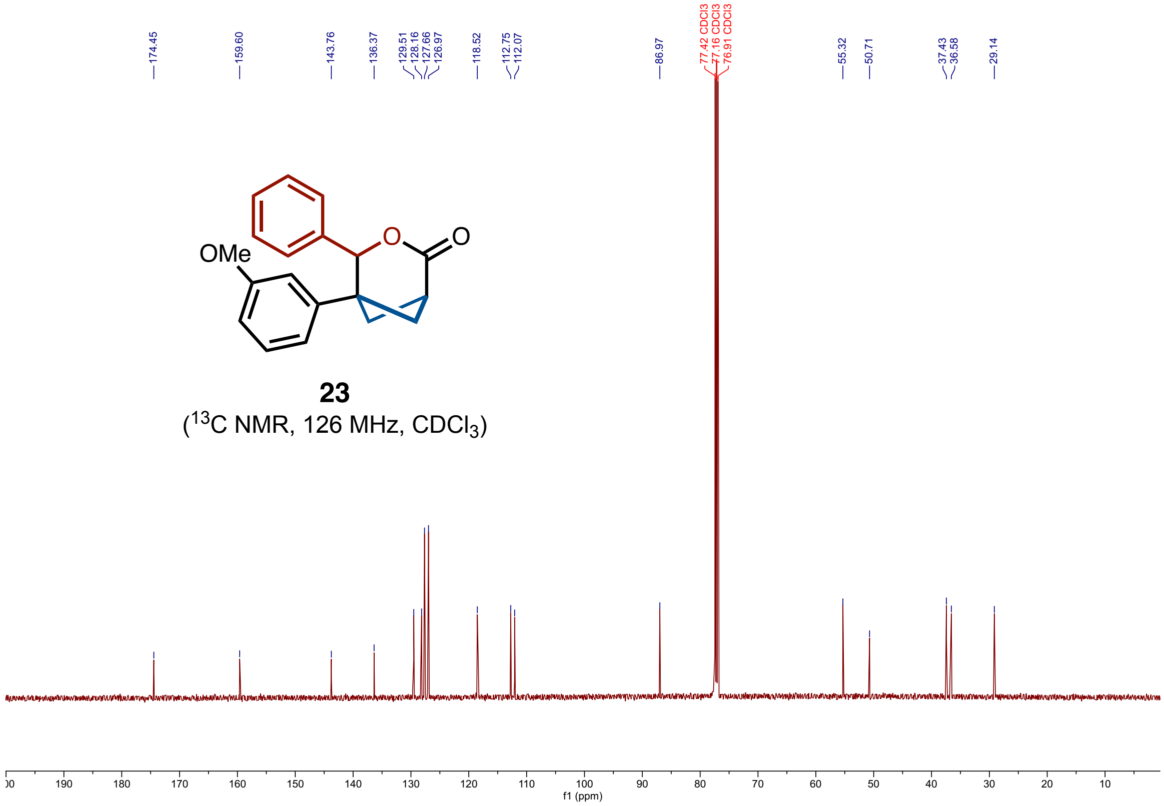

174.45
159.60
143.76
136.37
129.51
128.16
127.66
126.97
118.52
112.75
112.07
86.97
77.42 CDCl3
77.16 CDCl3
76.91 CDCl3
55.32
50.71
37.43
36.58
29.14
OMe
O
O
23
(13C NMR, 126 MHz, CDCl3)
f1 (ppm)

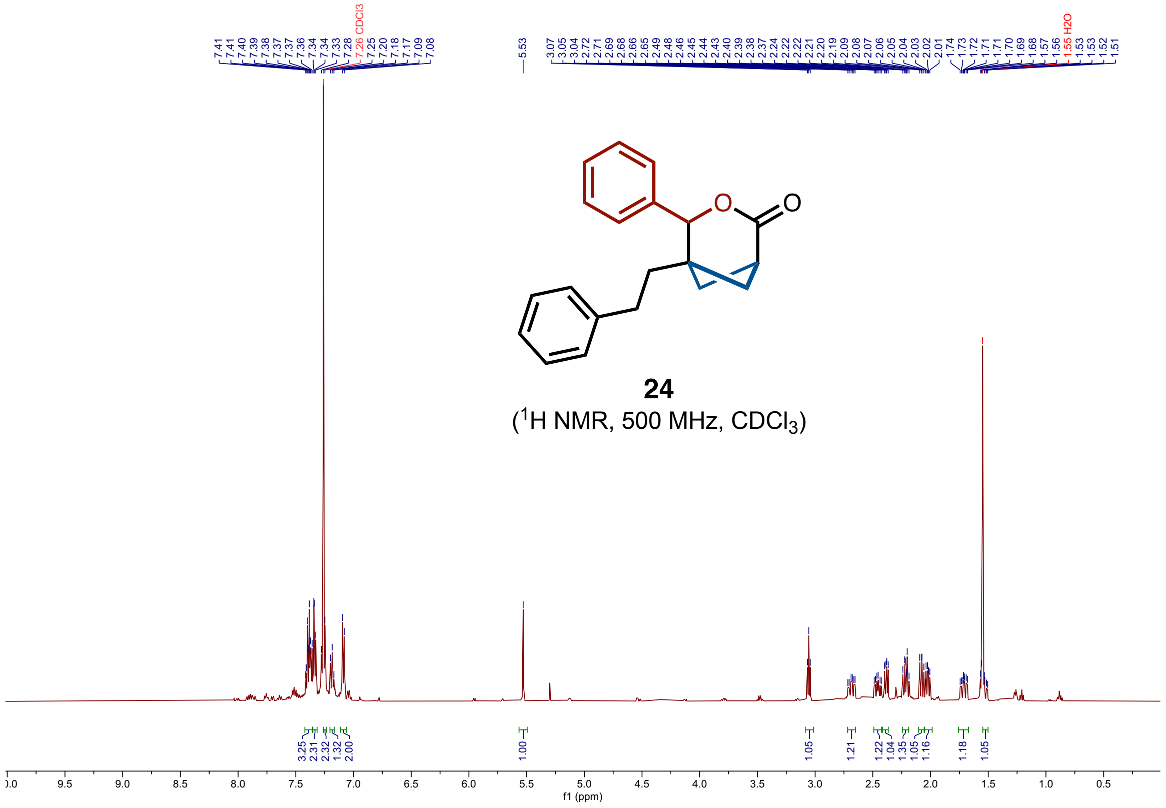

24

(^{1}H NMR, 500 MHz, $CDCl_3$)

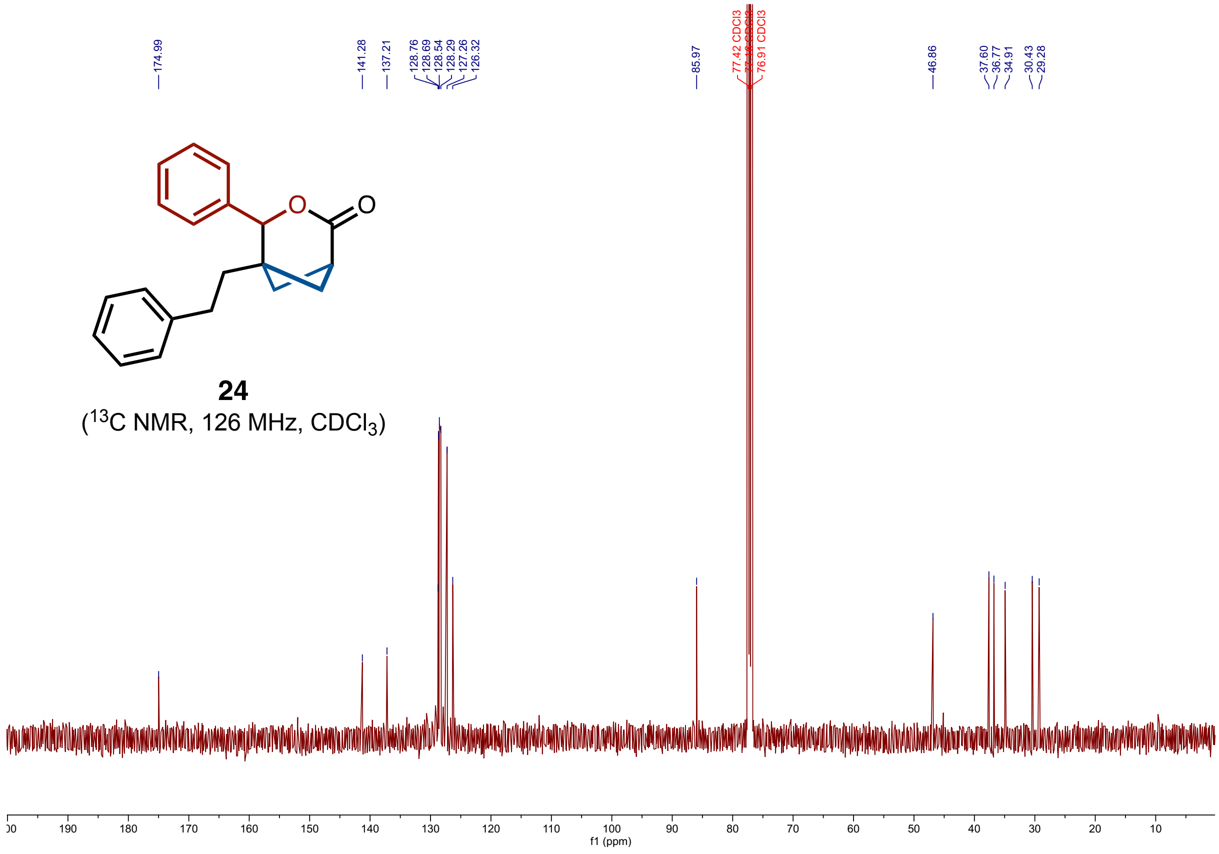

24

(^{13}C NMR, 126 MHz, $CDCl_3$)

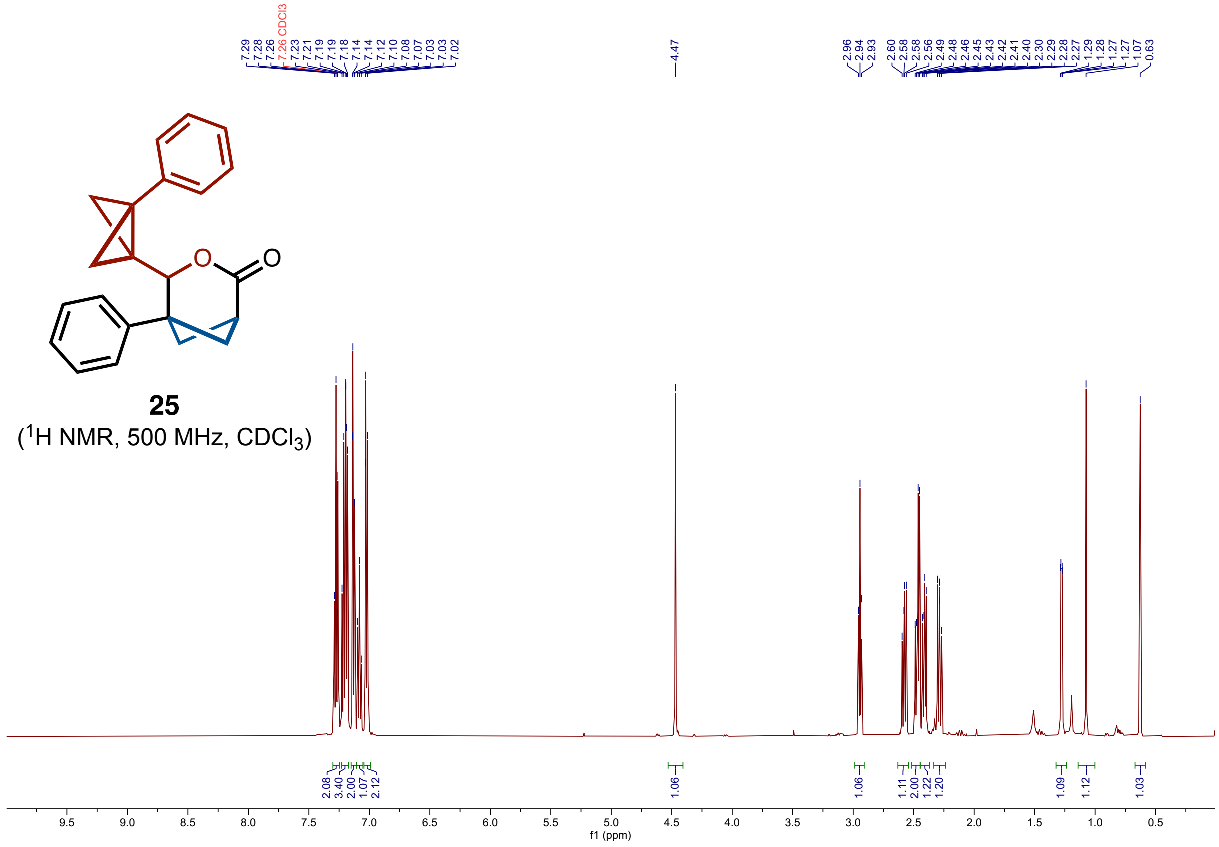

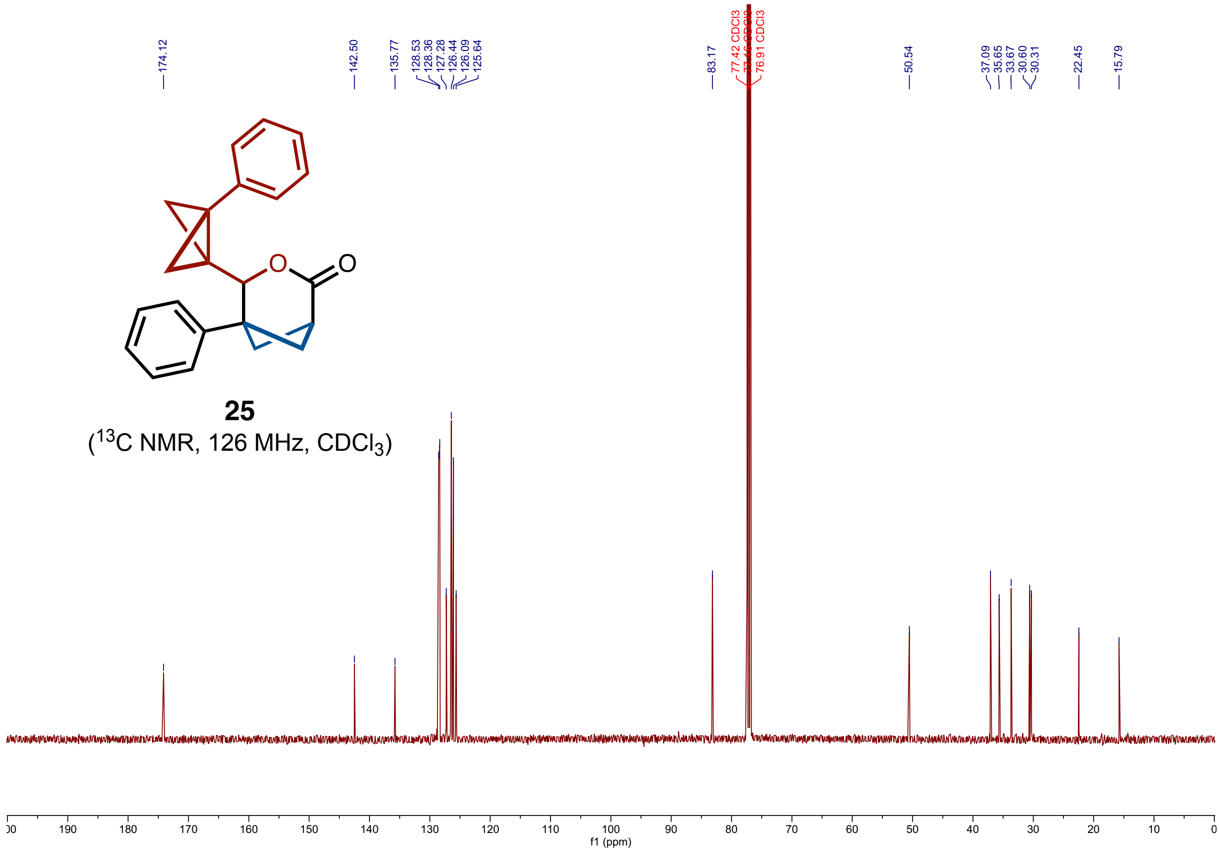

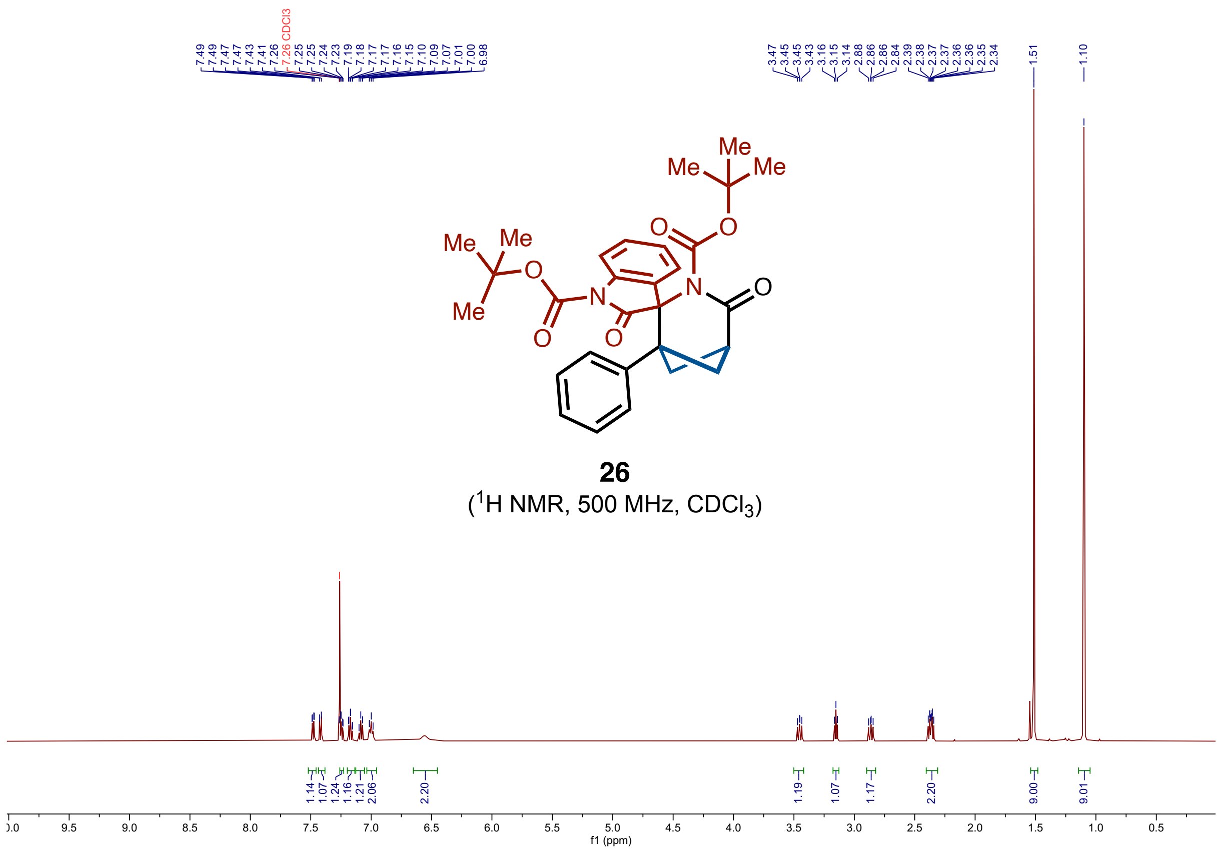

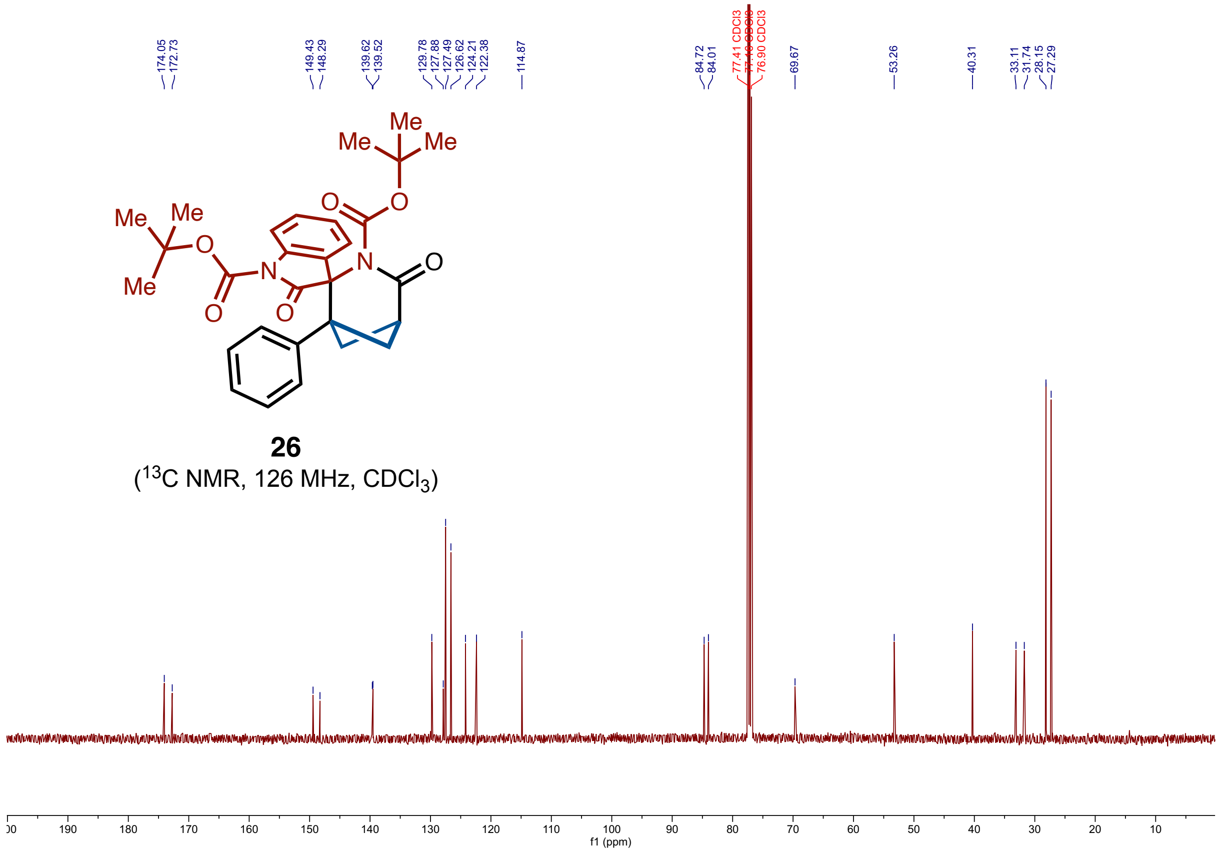

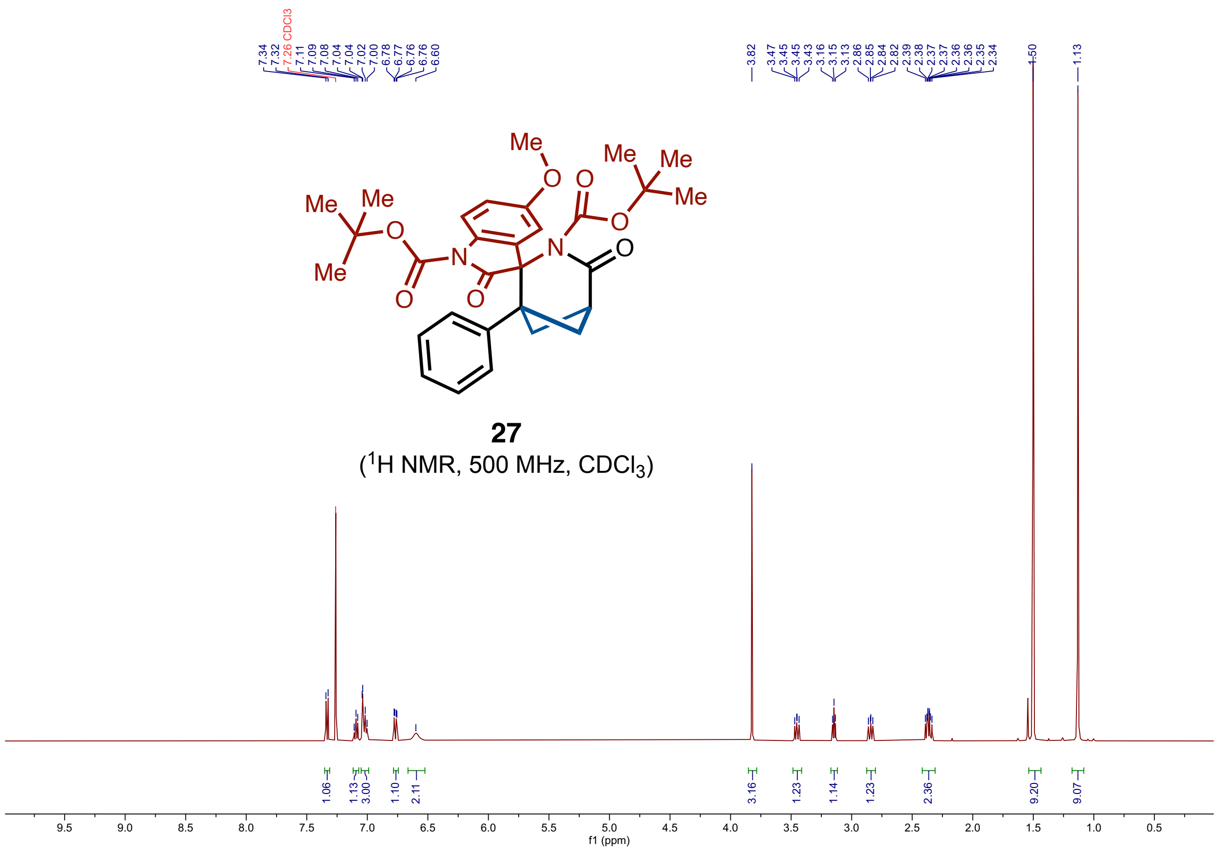

27
(^{1}H NMR, 500 MHz, CDCl$_3$)

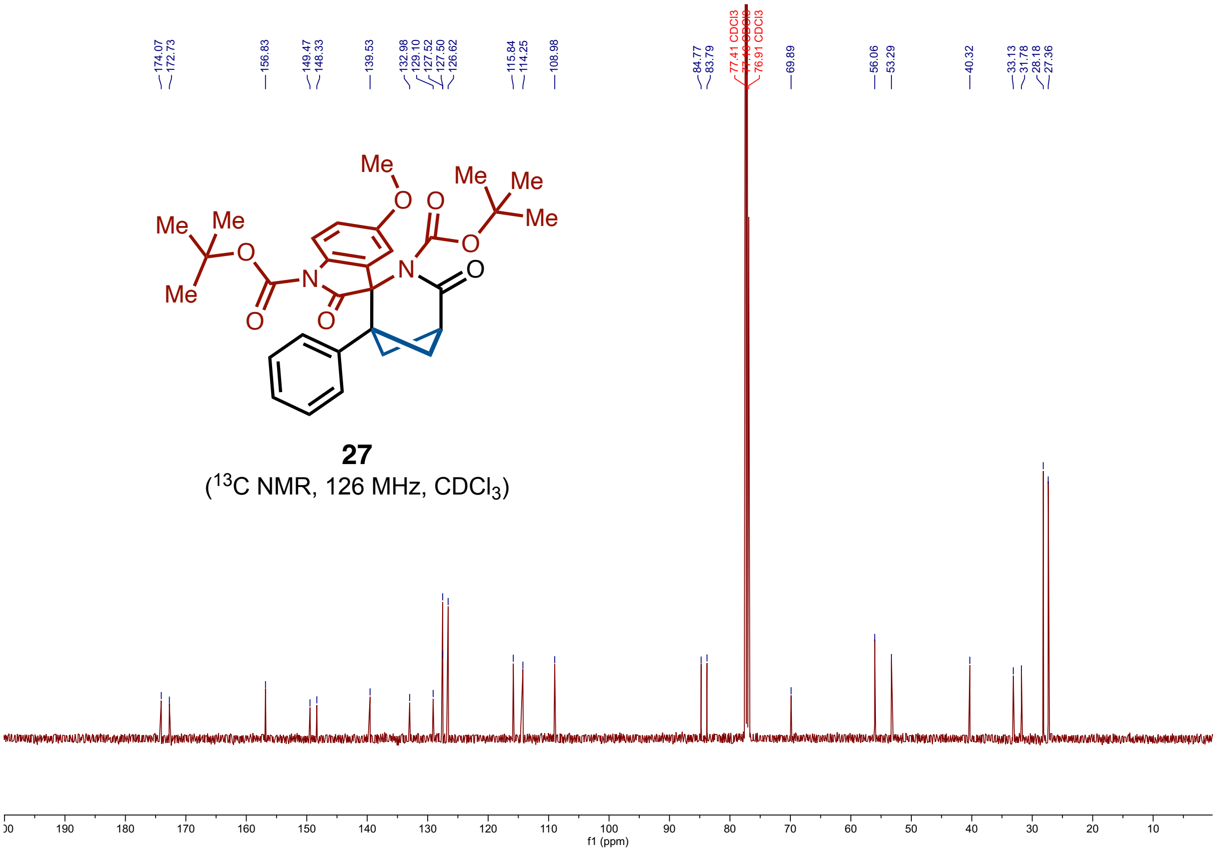

27
(^{13}C NMR, 126 MHz, CDCl$_3$)

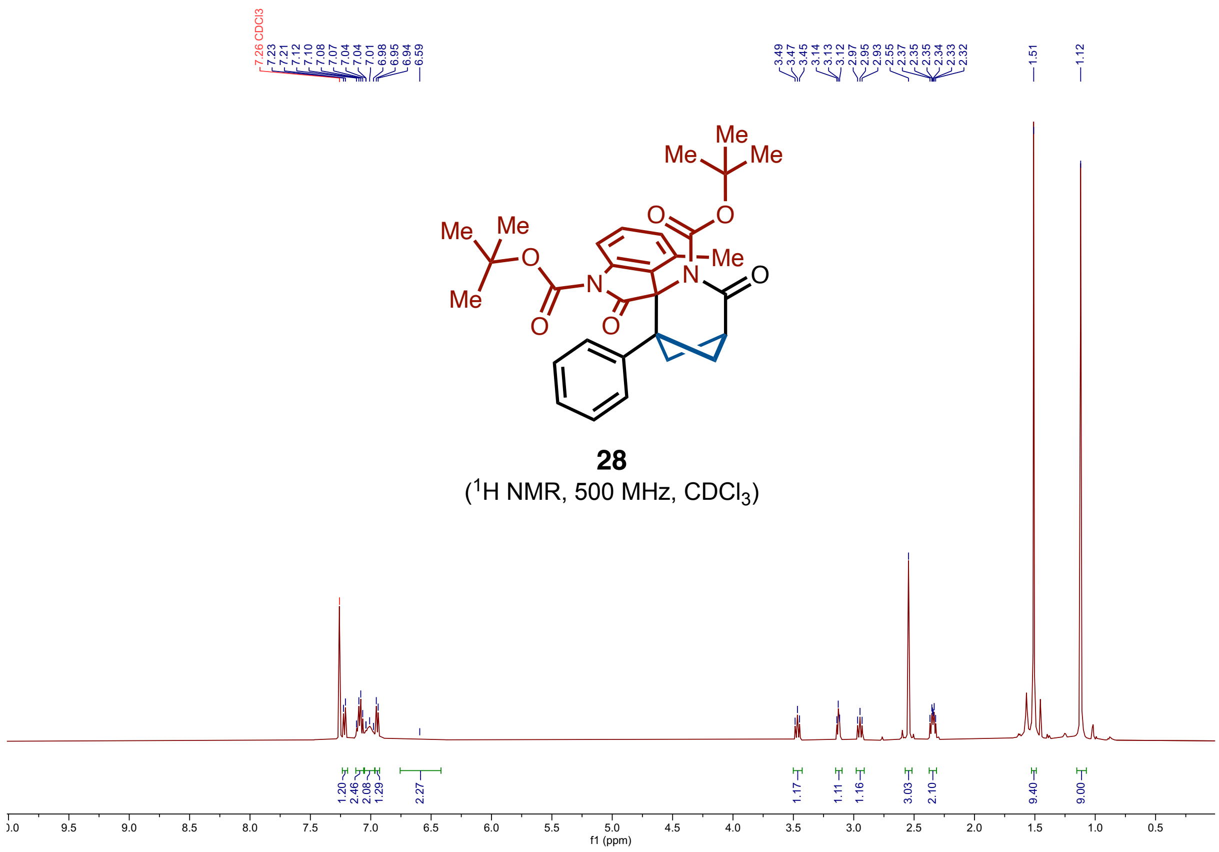

28
(^{1}H NMR, 500 MHz, $CDCl_3$)

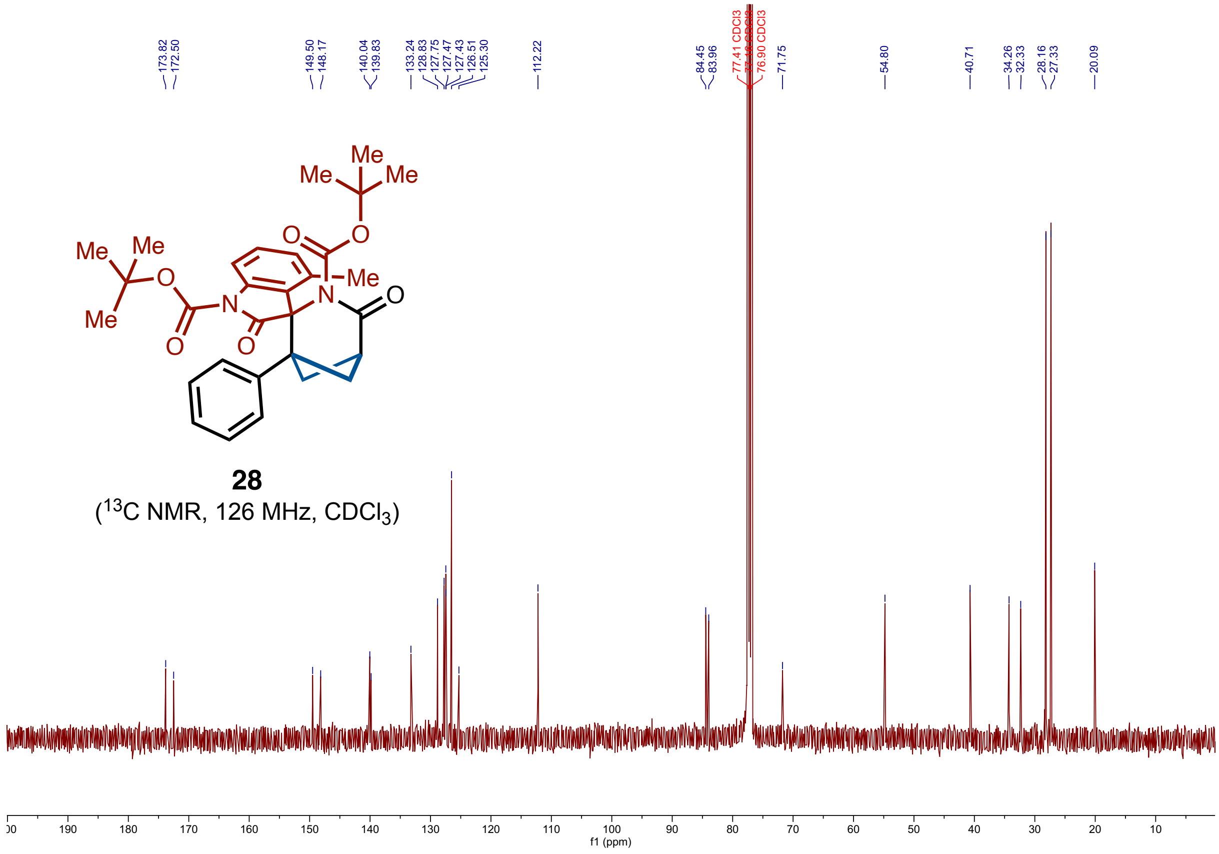

28
(^{13}C NMR, 126 MHz, $CDCl_3$)

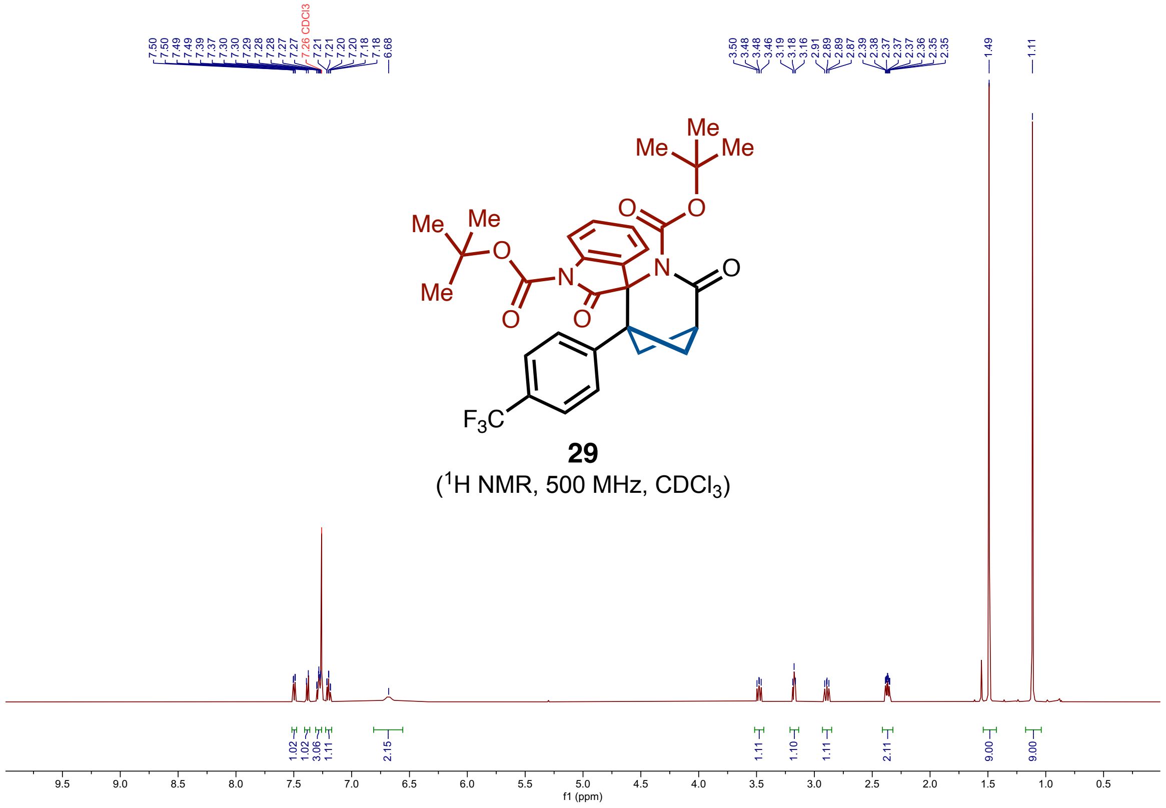
29
(¹H NMR, 500 MHz, CDCl₃)
f1 (ppm)

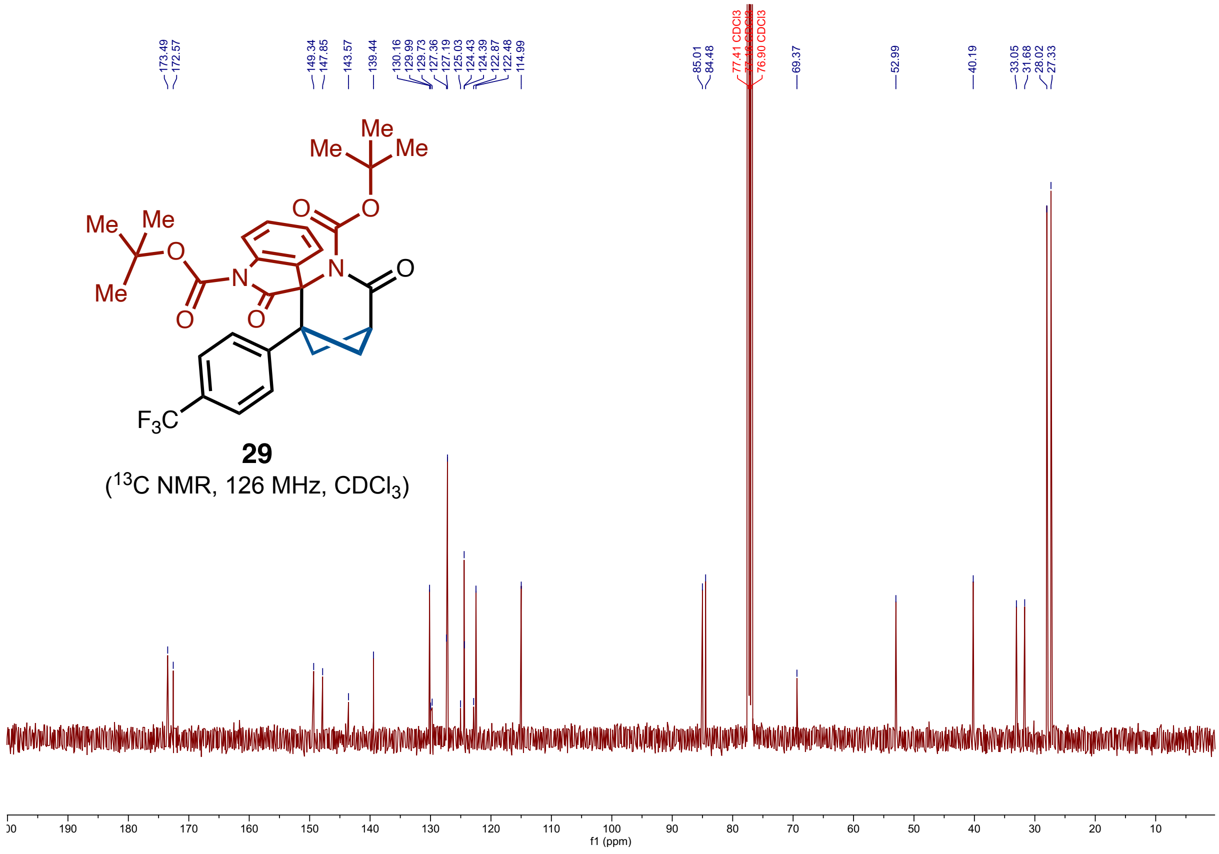
29
(¹³C NMR, 126 MHz, CDCl₃)
f1 (ppm)

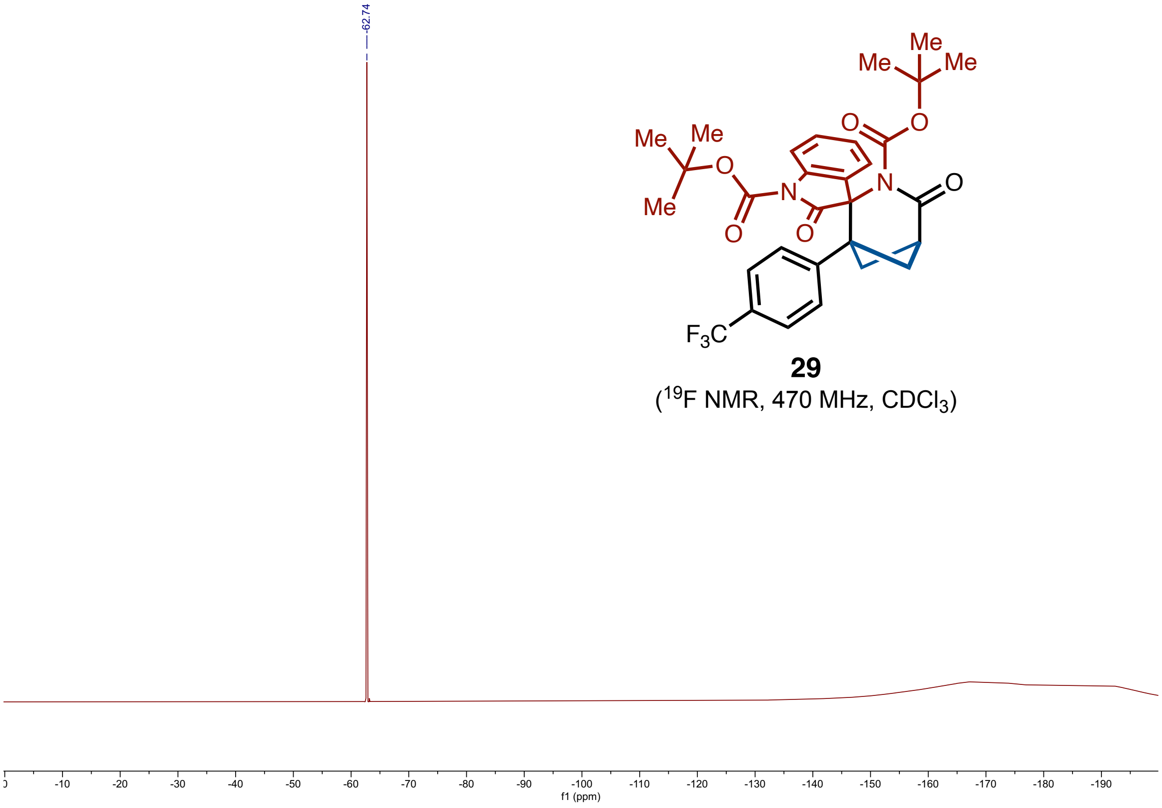

29

(^{19}F NMR, 470 MHz, $CDCl_3$)

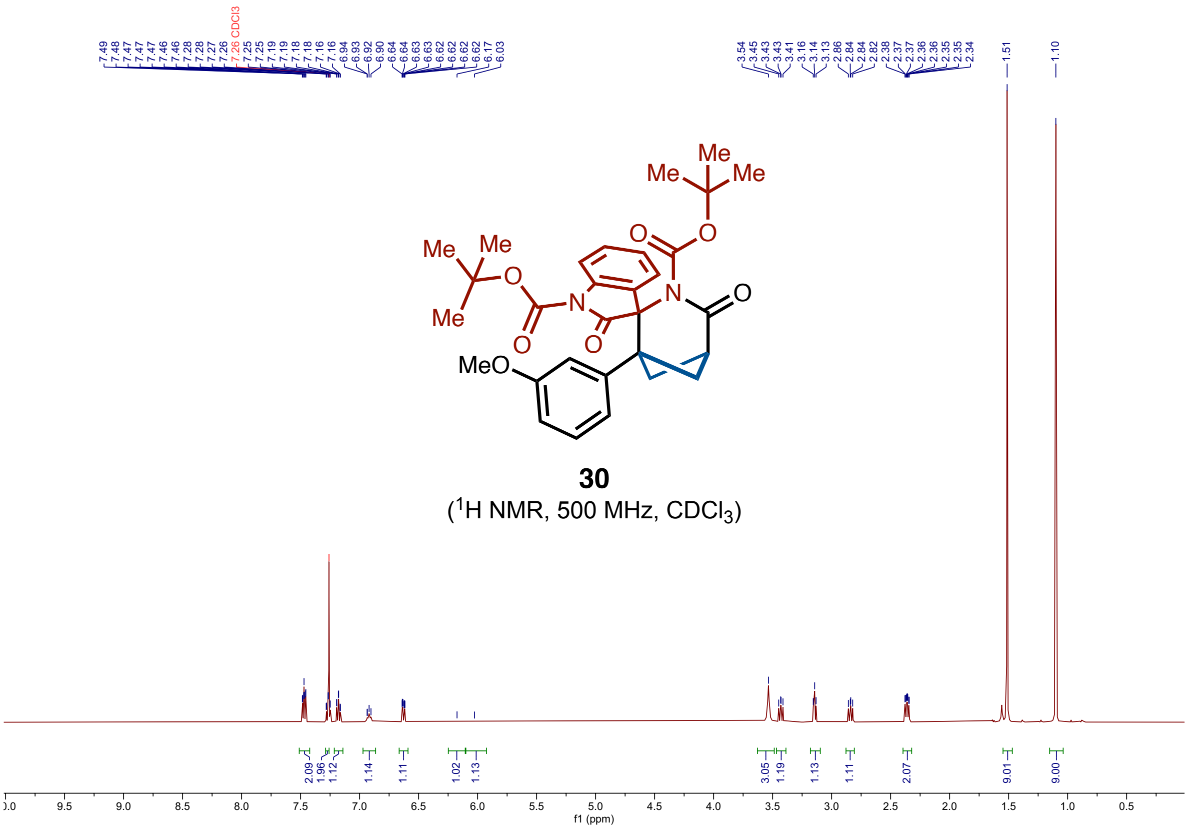
7.49 7.48 7.47 7.47 7.46 7.46 7.28 7.28 7.27 7.26 CDCl3 7.25 7.25 7.19 7.19 7.18 7.16 6.94 6.93 6.92 6.90 6.64 6.63 6.63 6.62 6.62 6.62 6.17 6.03
3.54 3.45 3.43 3.43 3.41 3.16 3.14 3.13 2.86 2.84 2.84 2.82 2.38 2.37 2.37 2.36 2.36 2.35 2.35 2.34 1.51 1.10
Me
Me
Me
Me
Me
Me
O
O
N
N
O
O
O
O
MeO
30
(1H NMR, 500 MHz, CDCl3)
2.09 1.96 1.12 1.14 1.11 1.02 1.13 3.05 1.19 1.13 1.11 2.07 9.01 9.00
f1 (ppm)

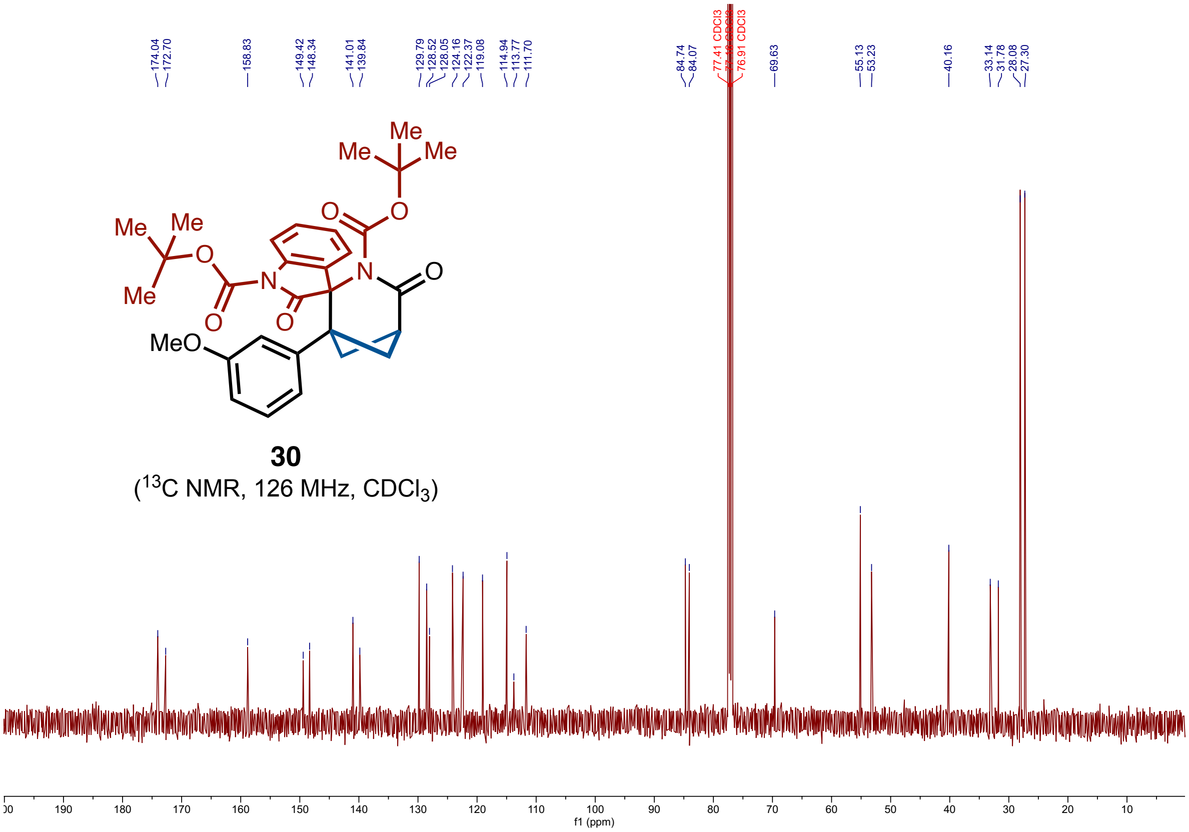
174.04 172.70 158.83 149.42 148.34 141.01 139.84 129.79 128.52 128.05 124.16 122.37 119.08 114.94 113.77 111.70 84.74 84.07 77.41 CDCl3 76.91 CDCl3 69.63 55.13 53.23 40.16 33.14 31.78 28.08 27.30
30
(13C NMR, 126 MHz, CDCl3)
f1 (ppm)

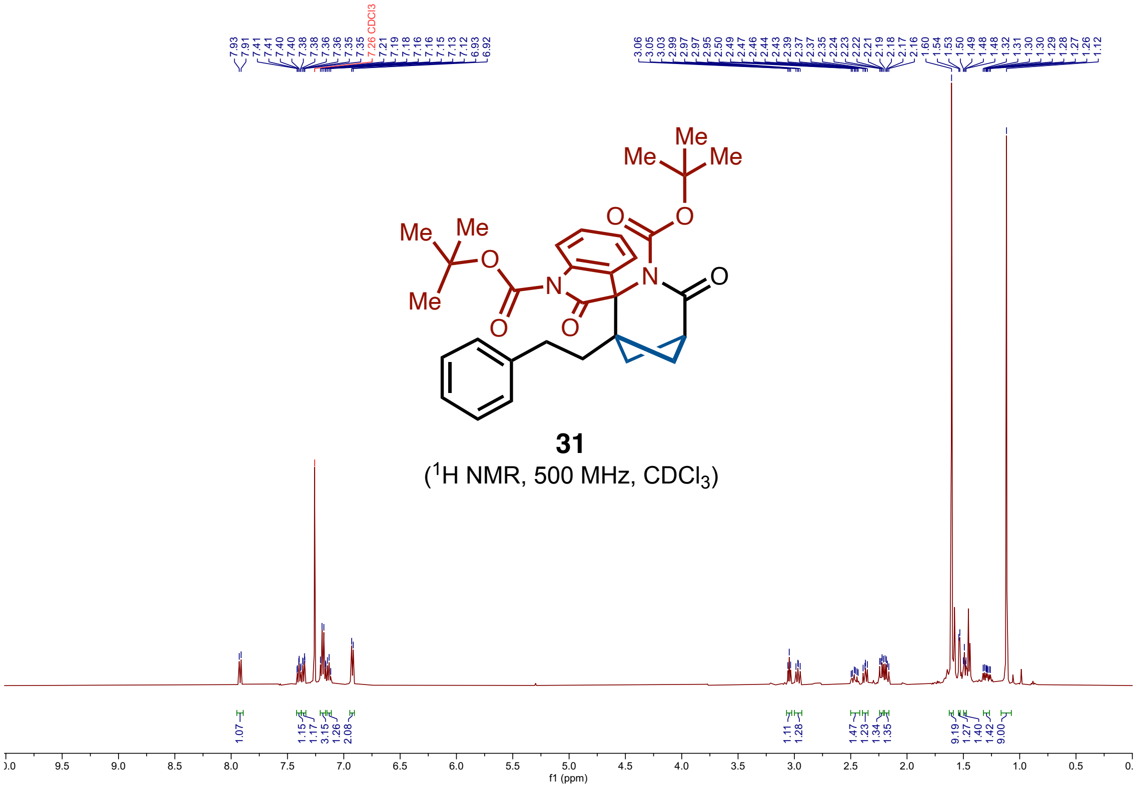

31
(^{1}H NMR, 500 MHz, $CDCl_3$)

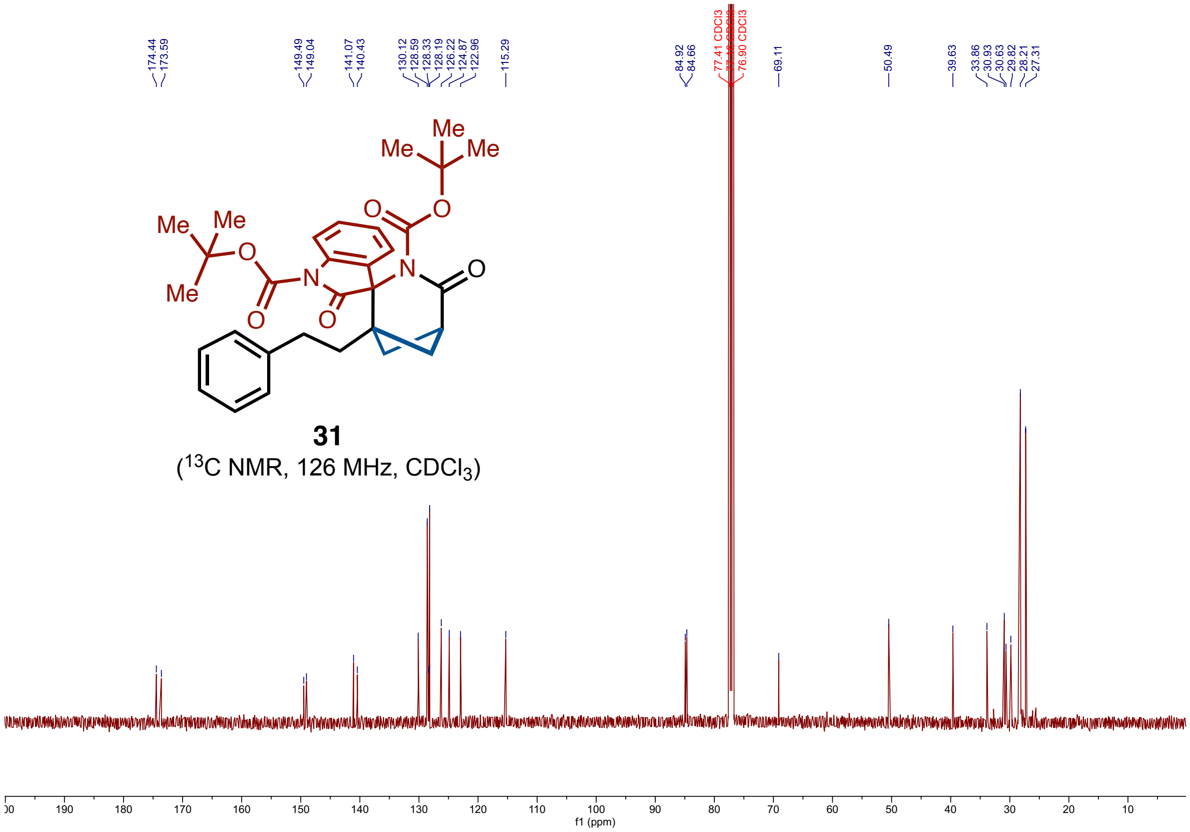

31
(^{13}C NMR, 126 MHz, $CDCl_3$)

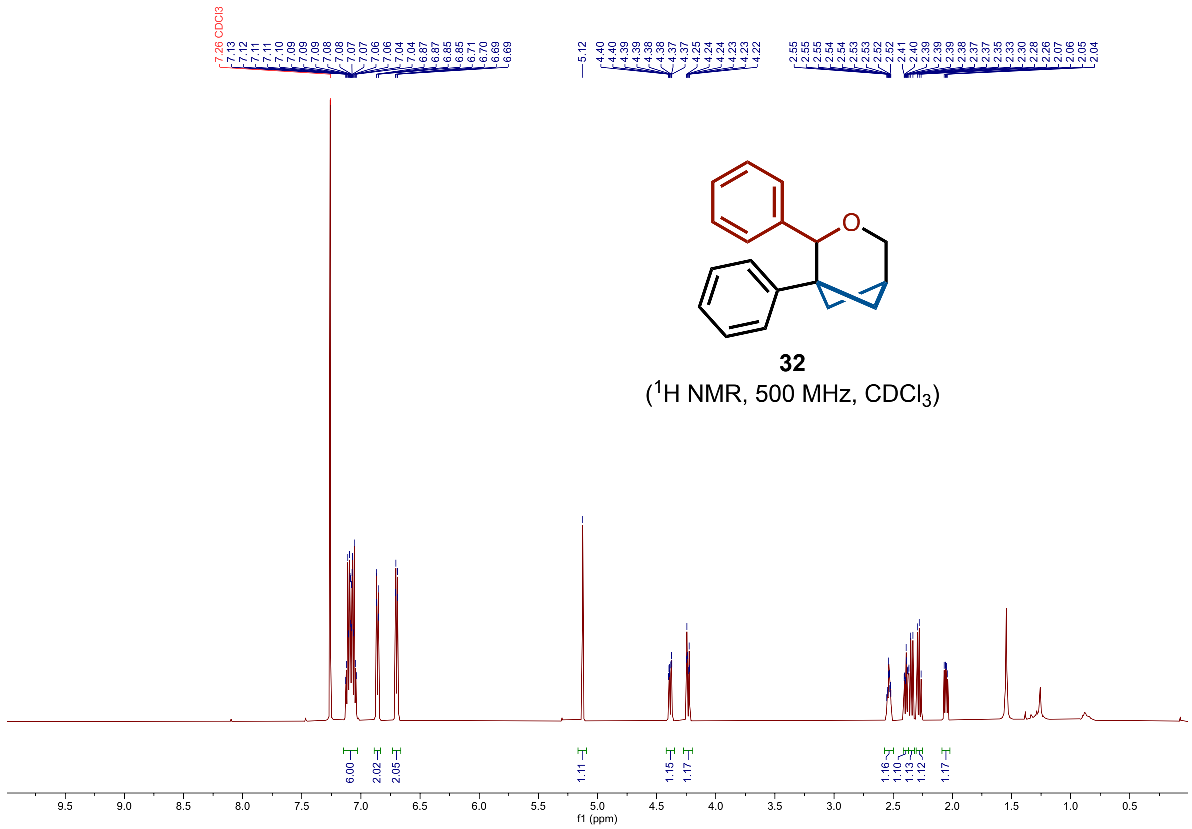
7.26 CDCl3
7.13
7.12
7.11
7.11
7.10
7.09
7.09
7.09
7.08
7.07
7.07
7.06
7.06
7.04
7.04
6.87
6.87
6.85
6.85
6.71
6.70
6.69
6.69
5.12
4.40
4.40
4.39
4.39
4.38
4.38
4.37
4.37
4.25
4.24
4.24
4.23
4.23
4.22
2.55
2.55
2.55
2.54
2.54
2.53
2.53
2.52
2.52
2.41
2.40
2.39
2.39
2.39
2.38
2.37
2.37
2.35
2.33
2.30
2.28
2.26
2.07
2.06
2.05
2.04
O
32
(^{1}H NMR, 500 MHz, CDCl$_3$)
6.00
2.02
2.05
1.11
1.15
1.17
1.16
1.10
1.13
1.12
1.17
f1 (ppm)

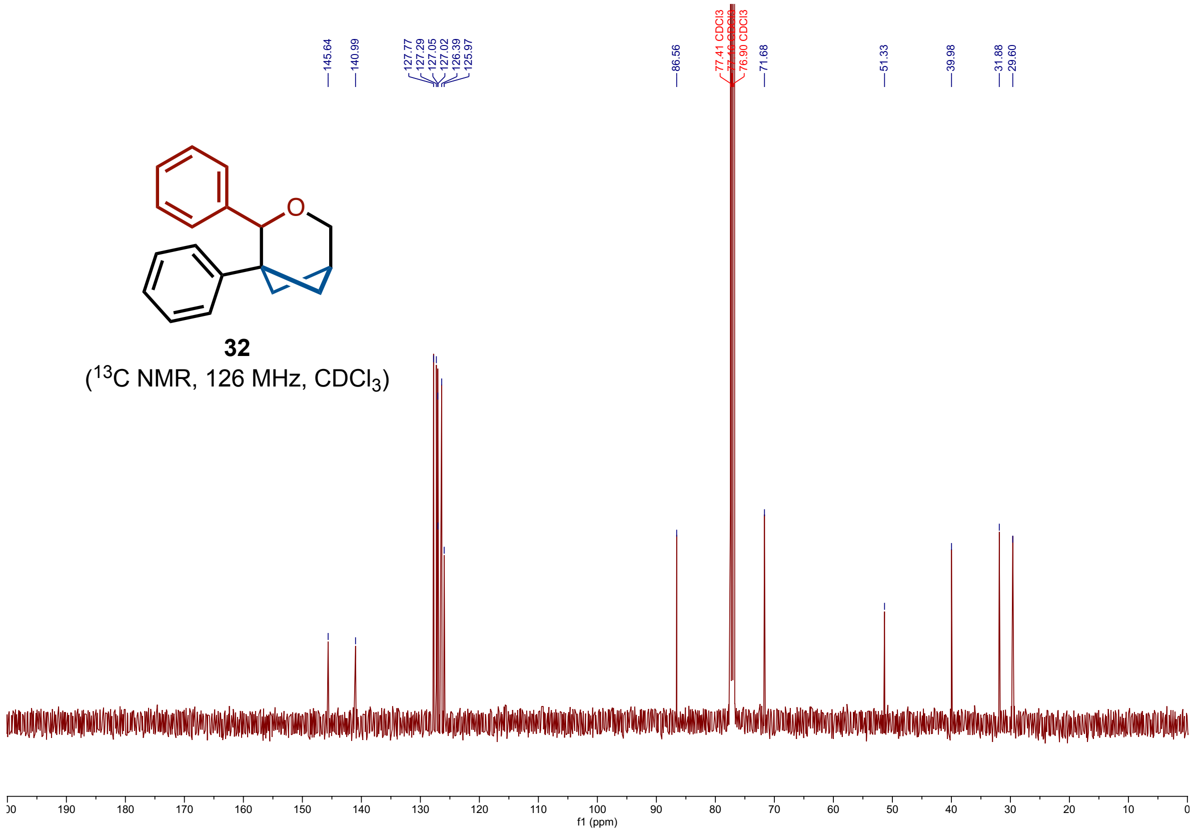
145.64
140.99
127.77
127.29
127.05
127.02
126.39
125.97
86.56
77.41 CDCl3
77.16 CDCl3
76.90 CDCl3
71.68
51.33
39.98
31.88
29.60
O
32
(^{13}C NMR, 126 MHz, CDCl$_3$)
f1 (ppm)

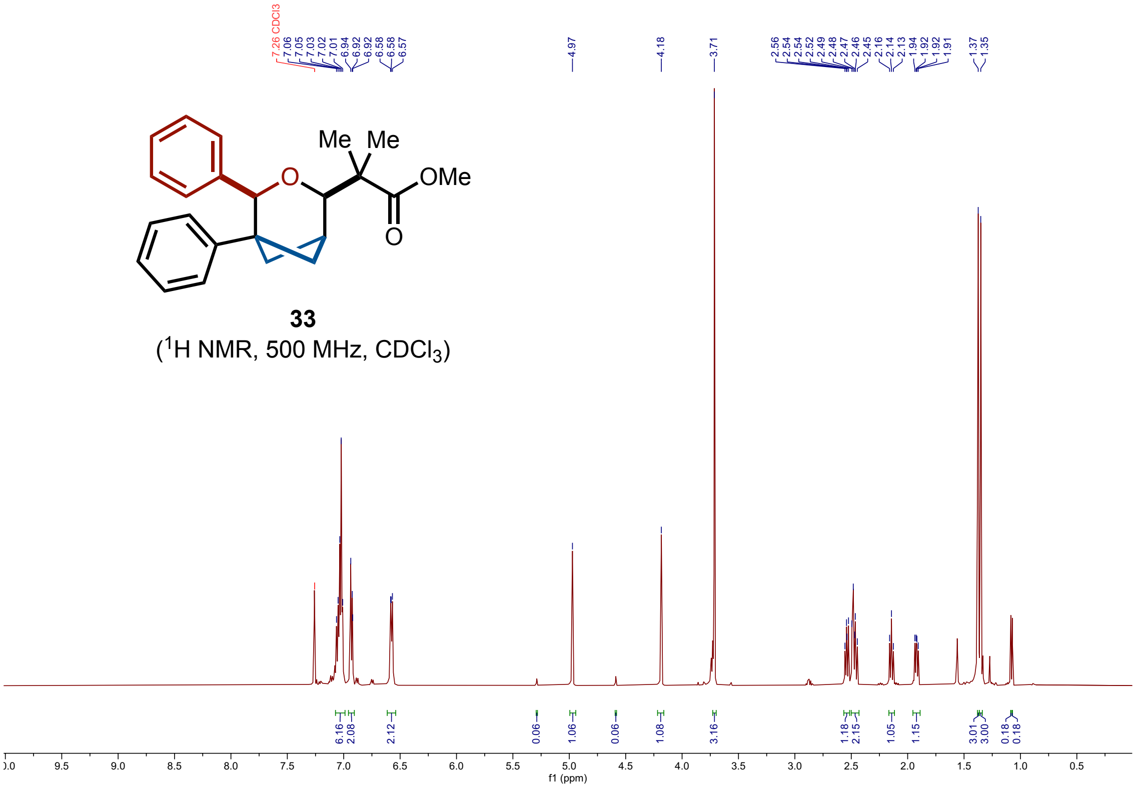

7.26 CDCl3
7.06
7.05
7.03
7.02
7.01
6.94
6.92
6.92
6.58
6.58
6.57
4.97
4.18
3.71
2.56
2.54
2.54
2.52
2.49
2.48
2.47
2.46
2.45
2.16
2.14
2.13
1.94
1.92
1.92
1.91
1.37
1.35
Me
Me
O
OMe
O
33
(¹H NMR, 500 MHz, CDCl₃)
6.16
2.08
2.12
0.06
1.06
0.06
1.08
3.16
1.18
2.15
1.05
1.15
3.01
3.00
0.18
0.18
f1 (ppm)

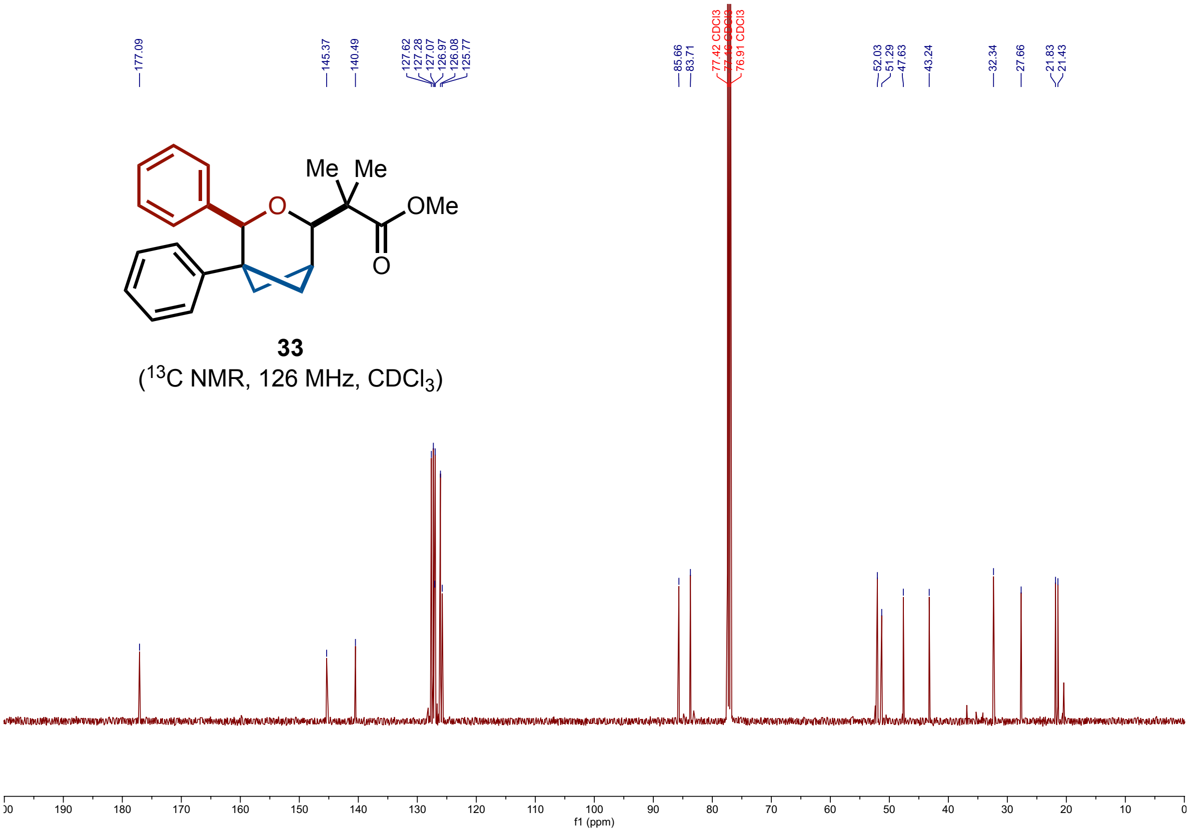

177.09
145.37
140.49
127.62
127.28
127.07
126.97
126.08
125.77
85.66
83.71
77.42 CDCl3
77.16 CDCl3
76.91 CDCl3
52.03
51.29
47.63
43.24
32.34
27.66
21.83
21.43
Me
Me
O
OMe
O
33
(¹³C NMR, 126 MHz, CDCl₃)
f1 (ppm)

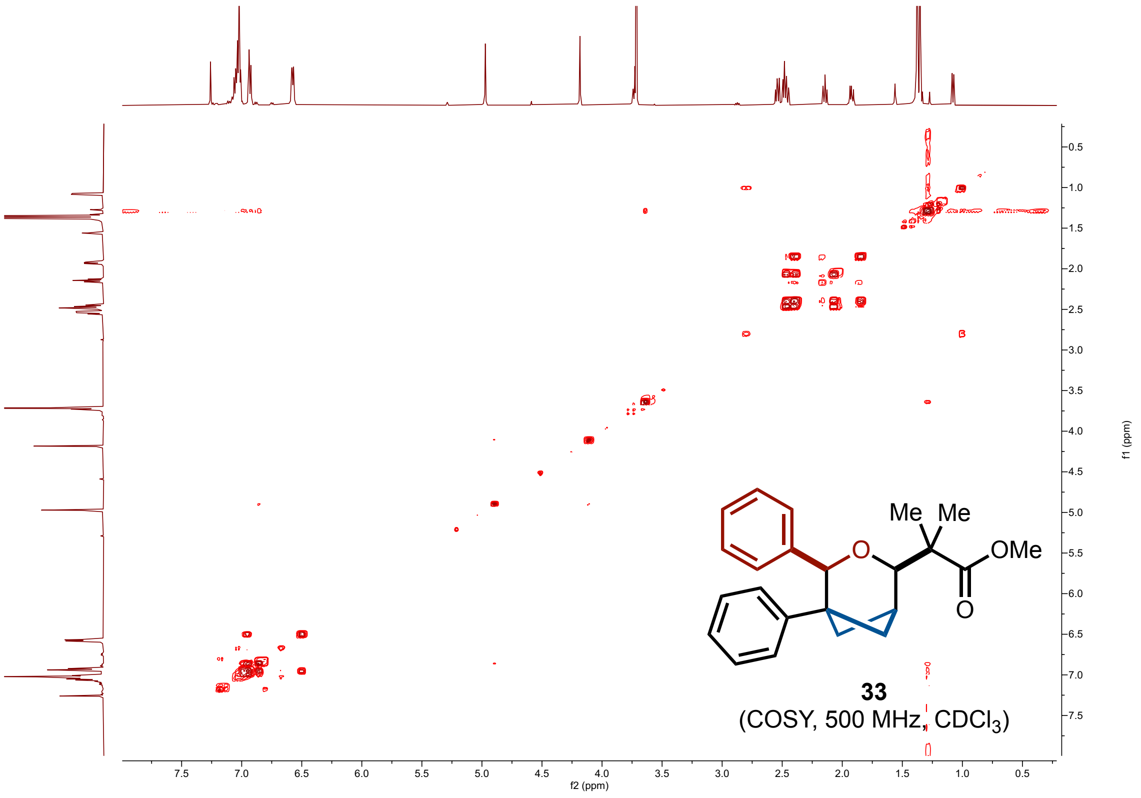
Me Me
O
OMe
O
33
(COSY, 500 MHz, CDCl3)
f2 (ppm)
f1 (ppm)

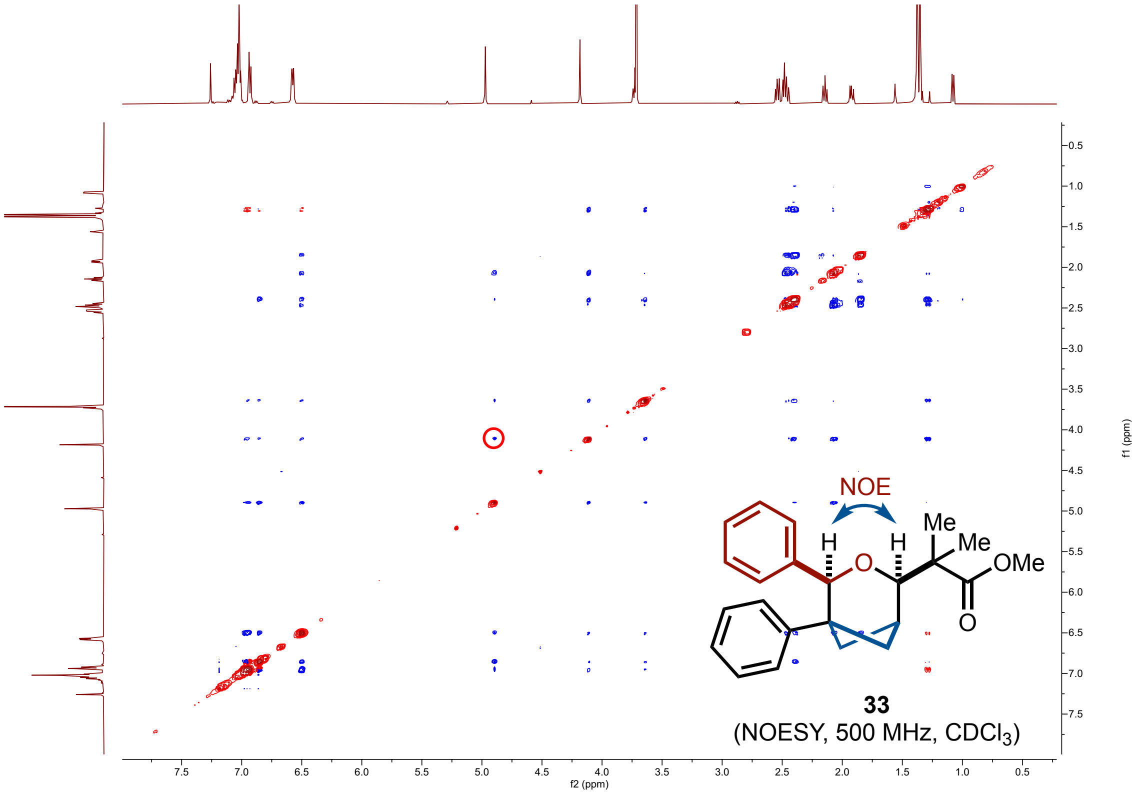
NOE
H
O
H
Me
Me
OMe
O
33
(NOESY, 500 MHz, CDCl3)
f2 (ppm)
f1 (ppm)

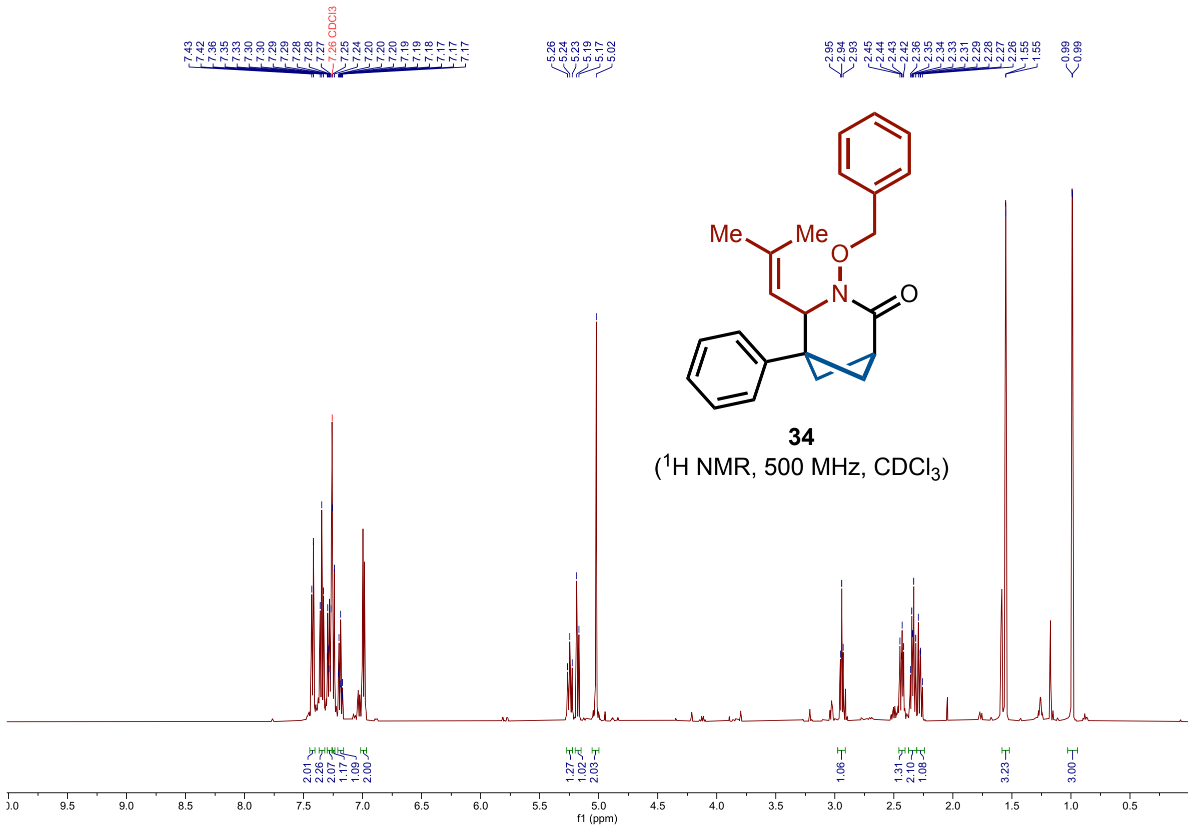
7.43 7.42 7.36 7.35 7.33 7.30 7.30 7.29 7.29 7.28 7.28 7.27 7.26 CDCl3 7.25 7.24 7.20 7.20 7.20 7.20 7.19 7.19 7.18 7.17 7.17 7.17
5.26 5.24 5.23 5.19 5.17 5.02
2.95 2.94 2.93 2.45 2.44 2.43 2.42 2.36 2.35 2.34 2.33 2.31 2.29 2.28 2.27 2.26 1.55 1.55
0.99 0.99
Me
Me
O
N
O
34
(1H NMR, 500 MHz, CDCl3)
2.01 2.26 2.07 1.17 1.09 2.00
1.27 1.02 2.03
1.06
1.31 2.10 1.08
3.23
3.00
f1 (ppm)

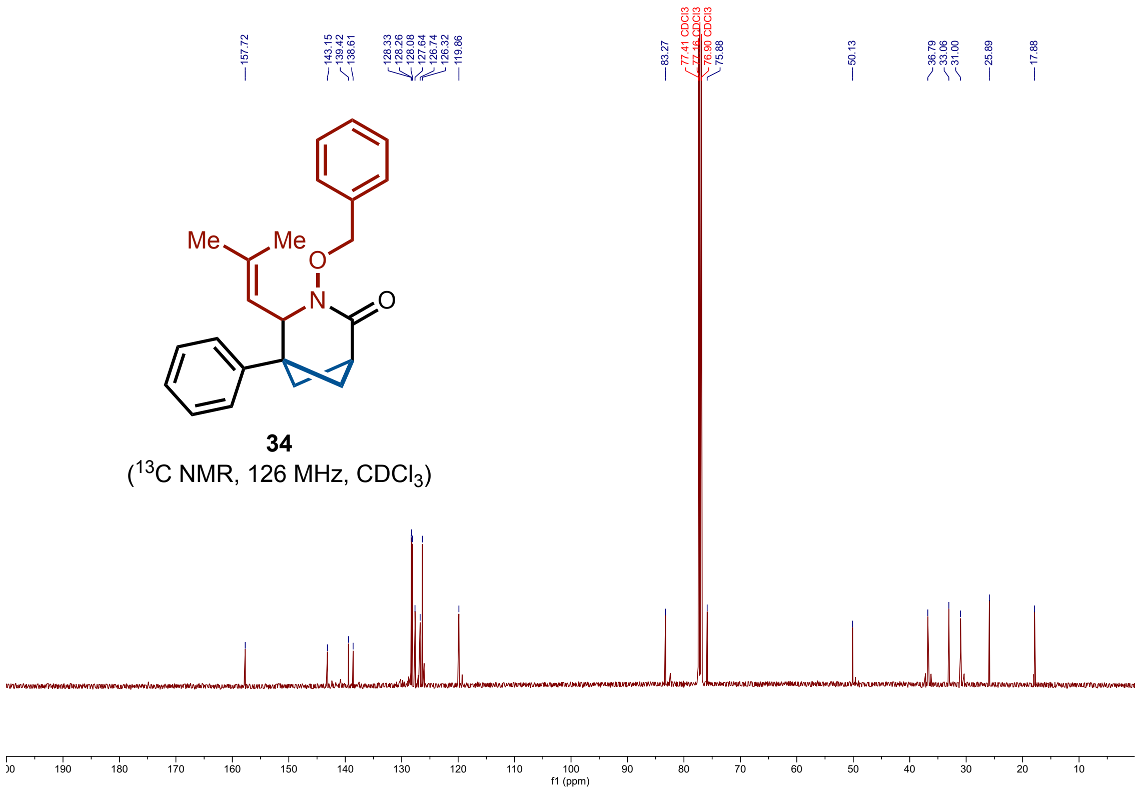
157.72
143.15 139.42 138.61
128.33 128.26 128.08 127.64 126.74 126.32 119.86
83.27
77.41 CDCl3 77.16 CDCl3 76.90 CDCl3 75.88
50.13
36.79 33.06 31.00
25.89
17.88
Me
Me
O
N
O
34
(13C NMR, 126 MHz, CDCl3)
f1 (ppm)

10. References:

[1] Y. Zi, Z.-J. Cai, S.-Y. Wang, S.-J. Ji, *Org. Lett.* **2014**, *16*, 3094-3097.

[2] K.-s. Lee, A. H. Hoveyda, *J. Org. Chem.* **2009**, *74*, 4455-4462.

[3] Y.-C. Chang, M. Martín, K. Bortey, Q. Lefebvre, T. Fessard, C. Salome, R. J. Vázquez, M. K. Brown, *J. Am. Chem. Soc.* **2025**, *147*, 14936-14944.

[4] Dutta, S.; Lee, D.; Ozols, K.; Daniliuc, C. G.; Shintani, R.; Glorius, F. Photoredox-Enabled Dearomative [2π + 2σ] Cycloaddition of Phenols. *J. Am. Chem. Soc.* **2024**, *146*, 2789-2797.

[5] H. Ren, T. Li, J. Xing, Z. Li, Y. Zhang, X. Yu, J. Zheng, *Org. Lett.* **2024**, *26*, 1745-1750.

[6] W. Yan, D. Wang, J. Feng, P. Li, D. Zhao, R. Wang, *Org. Lett.* **2012**, *14*, 2512-2515.

[7] A. Garcia, D. A. L. Otte, W. A. Salamant, J. R. Sanzone, K. A. Woerpel, *Angew. Chem. Int. Ed.* **2015**, *54*, 3061-3064.

[8] S. Bolshakov, J. L. Leighton, *Org. Lett.* **2005**, *7*, 3809-3812.

[9] N. Takeda, Y. Kobori, K. Okamura, M. Yasui, M. Ueda, *Org. Lett.* **2020**, *22*, 9740-9744.

[10] SAINT V8.41 (2024), Bruker AXS, Madison, WI, USA.

[11] L. Krause, R. Herbst-Irmer, G. M. Sheldrick, D. Stalke, *J. Appl. Cryst.,* **2015**, *48*, 3-10.

[12] G. M. Sheldrick, *Acta Cryst*. **2015**, *A71*, 3-8.

[13] G. M. Sheldrick, *Acta Cryst*. **2015**, *C71*, 3-8.
